# Recurrent somatic mutations of FAT family cadherins induce an aggressive phenotype and poor prognosis in anaplastic large cell lymphoma

**DOI:** 10.1038/s41416-024-02881-7

**Published:** 2024-10-30

**Authors:** Matteo Villa, Geeta G. Sharma, Federica Malighetti, Mario Mauri, Giulia Arosio, Nicoletta Cordani, Cosimo Lobello, Hugo Larose, Alessandra Pirola, Deborah D’Aliberti, Luca Massimino, Lucrezia Criscuolo, Lisa Pagani, Clizia Chinello, Cristina Mastini, Diletta Fontana, Silvia Bombelli, Raffaella Meneveri, Federica Lovisa, Lara Mussolin, Andrea Janikova, Šárka Pospíšilová, Suzanne D. Turner, Giorgio Inghirami, Fulvio Magni, Mario Urso, Fabio Pagni, Daniele Ramazzotti, Rocco Piazza, Roberto Chiarle, Carlo Gambacorti-Passerini, Luca Mologni

**Affiliations:** 1 Department of Medicine and Surgery, University of Milano-Bicocca, Monza, Italy; 2 Center of Molecular Medicine, Central European Institute of Technology (CEITEC), Masaryk University, Brno, Czech Republic; 3 Division of Cellular and Molecular Pathology, Department of Pathology, University of Cambridge, Cambridge, UK; 4 Galseq srl, Bresso, Italy; 5 Division of Immunology, Transplantation and Infectious Disease, IRCCS Ospedale San Raffaele, Milano, Italy; 6 Neurogenomics Research Center, Fondazione Human Technopole, Milano, Italy; 7 Maternal and Child Health, Department Pediatric Hematology, Oncology and Stem Cell Transplant Center, University of Padua, Padua, Italy; 8 Pediatric Research Institute “Città della Speranza”, Padua, Italy; 9 Faculty of Medicine, Masaryk University, Brno, Czech Republic; 10 Weill Cornell Medicine, New York, NY USA; 11 Department of Pathology, Fondazione IRCCS San Gerardo dei Tintori, Monza, Italy; 12 Department of Molecular Biotechnology and Health Sciences, University of Torino, Torino, Italy; 13 Department of Pathology, Children’s Hospital and Harvard Medical School, Boston, MA USA; 14 Division of Haematopathology, European Institute of Oncology IRCCS, Milan, Italy; 15 Department of Haematology, Fondazione IRCCS San Gerardo dei Tintori, Monza, Italy

**Keywords:** Oncology, Lymphoma

## Abstract

**Background:**

Anaplastic Large Cell Lymphoma (ALCL) is a rare and aggressive T-cell lymphoma, classified into ALK-positive and ALK-negative subtypes, based on the presence of chromosomal translocations involving the ALK gene. The current standard of treatment for ALCL is polychemotherapy, with a high overall survival rate. However, a subset of patients does not respond to or develops resistance to these therapies, posing a serious challenge for clinicians. Recent targeted treatments such as ALK kinase inhibitors and anti-CD30 antibody-drug conjugates have shown promise but, for a fraction of patients, the prognosis is still unsatisfactory.

**Methods:**

We investigated the genetic landscape of ALK + ALCL by whole-exome sequencing; recurring mutations were characterized in vitro and in vivo using transduced ALCL cellular models.

**Results:**

Recurrent mutations in *FAT* family genes and the transcription factor *RUNX1T1* were found. These mutations induced changes in ALCL cells morphology, growth, and migration, shedding light on potential factors contributing to treatment resistance. In particular, *FAT4* silencing in ALCL cells activated the β-catenin and YAP1 pathways, which play crucial roles in tumor growth, and conferred resistance to chemotherapy. Furthermore, STAT1 and STAT3 were hyper-activated in these cells. Gene expression profiling showed global changes in pathways related to cell adhesion, cytoskeletal organization, and oncogenic signaling. Notably, *FAT* mutations associated with poor outcome in patients.

**Conclusions:**

These findings provide novel insights into the molecular portrait of ALCL, that could help improve treatment strategies and the prognosis for ALCL patients.

## Background

Anaplastic large cell lymphoma (ALCL) is an aggressive CD30 + T-cell lymphoma representing approximately 3% of adult non-Hodgkin lymphomas. ALCL is molecularly divided into anaplastic lymphoma kinase (ALK)-positive and ALK-negative subtypes, depending on the presence of a chromosomal translocation involving the *ALK* gene [1, 2]. *ALK* translocations cause inappropriate high expression and constitutive hyper-activation of the ALK kinase, leading to the activation of downstream signaling, including RAS, JAK/STAT and PI3K pathways [3, 4]. Current frontline therapy is based on polychemotherapy, providing 60–90% overall survival (OS) at 5 years from diagnosis [5–7]. Despite excellent results, there is a fraction of patients that do not respond or develop resistance to therapies, for reasons that are poorly understood. In general, relapsed or refractory patients have a poor prognosis. More recently, targeted therapeutic approaches have included the ALK kinase inhibitor, crizotinib and the drug-conjugate anti-CD30 antibody, brentuximab vedotin [8–10]. Chemoresistant ALK+ patients achieve high response rates to crizotinib, however 30–40% of them quickly relapse, usually within 3 months [10].

While the ALK fusion kinase clearly drives disease, it is not known if and how additional variants (co-mutations) coexisting with the ALK translocation may impact on tumor biology and explain the observed heterogeneity in clinical presentation, morphologic features, and response to treatment. To address this question, we analyzed the whole exome sequence of 27 ALK + ALCL diagnostic samples, and then further extended investigation to ALK-negative patients. This analysis revealed recurrent mutations of *FAT* family genes and *RUNX1T1* in ALCL patients. The consequences of *FAT4* loss of function and the activity of RUNX1T1 mutants were characterized in ALCL cells.

## Methods

### Clinical samples and data

Formalin-fixed paraffin-embedded (FFPE) or fresh frozen tissue from infiltrated lymph nodes, and matched peripheral blood samples were obtained from 32 ALK + ALCL patients in agreement with the Declaration of Helsinki, after written informed consent and ethical approval by local committees from the University Hospital Brno (Czech Republic), Fondazione IRCCS San Gerardo dei Tintori and Istituto di Ricerca Pediatrica Fondazione Città della Speranza (Italy); 16 cases were excluded from WES analysis because of low tumoral fraction (<30% tumor cells) or poor DNA quality. Additional sequencing data from 11 ALK+ and 16 ALK- patients were obtained from Weill Cornell Medical College (USA) and Children’s Cancer and Leukemia Group (UK), from existing datasets [11, 12].

### Whole-exome sequencing

Genomic DNA was isolated as described [13] and DNA quality was assessed using quantitative real-time PCR amplification (qPCR). DNA samples were sent to Galseq (Italy) for library preparation with the SureSelect Human All Exon v6 kit and paired-end sequencing at an average on-target depth of 100x. Raw fastq files were aligned to the reference human genome (GRCh38/hg38) and analyzed by CEQer2 [14]. Variants that were present in >25% of the tumor sample and <10% of control DNA were called, if the coverage of the corresponding position was ≥20x in both tumor and normal samples, and if they did not align within repetitive elements. Synonymous and non-coding substitutions were filtered out. Recurring variants were validated by PCR amplification and Sanger sequencing.

### Drugs, plasmids, cell lines

Crizotinib was purchased from Selleck Chemicals (Houston, TX, USA). Cyclophosphamide, 4-hydroperoxy-cyclophosphamide (4-HC), vincristine sulfate and doxorubicin were purchased from SIGMA-Aldrich (St. Louis, MO, USA). All drugs were dissolved in DMSO at 10 mmol/L stock concentration, aliquoted and stored at –20 °C until used. Cell lines were purchased from ATCC: SUP-M2 cells were used as an ALCL model with NPM::ALK translocation and grown in RPMI medium supplemented with 10% fetal bovine serum (FBS), L-glutamine and antibiotics; the human embryonic kidney cell line HEK-293T was employed for proteomics and gene expression studies and was grown in DMEM + 10% FBS. To obtain stable FAT4-silenced SUP-M2 cells, commercial lentiviral shRNA SMARTvectors targeting human *FAT4* and expressing GFP reporter gene were employed (Dharmacon; Lafayette, CO, USA). A scrambled shRNA vector was used for the control. Each construct was co-transfected with the VSV-G and CMV-8.74 plasmids by lipofection in HEK-293FT packaging cells using JetPrime reagent (Polyplus; Illkirch, France) for virus production. Lentiviral particles were then used for spin infection of SUP-M2 cells. Transduced cells were selected with puromycin (1 µg/ml) and sorted by GFP positivity. Cell clones were then isolated by limiting dilution. Clones #1 and #3 derive from the same bulk population expressing a mixture of three shRNA sequences: TCGATCATCATCTCTTGCC (targeting *FAT4* open reading frame [ORF]), AATACACATACGCCACTGG (targets 3’UTR) and GCGGTGTCTAAGATGACTT (targets ORF); clone #2 derives from an independent transfectant population carrying the 3’UTR targeted shRNA. Wild-type and mutant HA-tagged RUNX1T1 open reading frame sequences (Clone ID: OHu22034C) were purchased from GenScript (Leiden, Netherlands) and subcloned in pCDH-CMV-EF1 at NheI sites. The plasmids were used for standard lipofection in HEK-293T cells and for spin infection in SUP-M2 cells. In both cases, transfected cells were selected with puromycin (1 µg/ml).

### Cell proliferation and clonogenic assays

Ten thousand cells per well were seeded in triplicate in 96-well microplates. For dose-response curves, the cells were treated with vehicle or increasing drug concentrations for 72 h. For time course experiments, the cells were followed for 10 days with dilution on day 5 to avoid saturation. Cell growth and viability were assessed using the CellTiter 96® Aqueous One Solution Cell Proliferation Assay System (Promega, Madison, WI, USA) following manufacturer’s instructions. Absorbance at 490 nm was recorded using the Infinite F200 PRO microplate reader (Tecan). Proliferation curves were generated using GraphPad Prism software (GraphPad Software, Inc.). For soft agar colony assays, 2 × 10^4^ cells were embedded in 0.5% low melting point agar medium (type VII-A, Sigma) and seeded on a 0.5% low melting point agar layer. After 15 days, colonies were counted using the ChemiDoc XRS+ system (Biorad) and ImageJ software.

### Actin rearrangement analysis

The cells were washed twice with PBS and incubated for 10 min at room temperature in 4% paraformaldehyde in 0.12 M sodium phosphate buffer, pH 7.4, then left in PBS overnight at 4 °C. Actin staining was performed for 2 h at room temperature with Alexa Fluor 594 phalloidin (Thermo Fisher Scientific) (1:400 dilution in GDB buffer [0.02 M sodium phosphate buffer, pH 7.4, containing 0.45 M NaCl, 0.2% (w/v) bovine gelatin]), followed by staining with Hoechst 33342 (Thermo Fisher Scientific), coverslips were mounted on glass slides with a 90% (v/v) glycerol/PBS solution. Images were acquired using a Zeiss LSM 710 confocal laser-scanning microscope (Zeiss) using a 63x, 1.4 N/A oil-immersion objective creating a full z-stack for each analyzed cell. Laser intensities and acquisition parameters were held constant throughout each experiment. Confocal microscopy fields were analyzed using a specific macro with ImageJ (https://imagej.nih.gov/ij/) software. Briefly, actin signal was analyzed by measuring the membrane distribution of positive actin staining, comparing the circularity features recorded in shNT *vs* shFAT4 cells and applying a roundness correction as described [15]. All the data obtained were derived from at least 75 fields per experimental condition. Data are expressed as mean ± standard deviation.

### Immunofluorescence

The cells were fixed in 4% paraformaldehyde, washed in PBS and stained with PE-conjugated anti-CD30 antibody (Beckman Coulter, cat# IM2033U), or with anti-β-catenin (BDbiosciences, cat# 610154) or anti-YAP1 (Santa Cruz Biotechnology, cat# sc-376830) primary antibodies in GDB (0.02 M sodium phosphate buffer, pH 7.4, containing 0.45 M NaCl, 0.2% (w/v) bovine gelatin, 1% Titon-x100), followed by staining with Alexa 488-conjugated secondary antibody (Thermo Fisher Scientific) for 1 h. After two washes with PBS and staining with Hoechst 33342 (Thermo Fisher Scientific), coverslips were mounted on glass slides with a 90% (v/v) glycerol/PBS solution. To calculate cell size, confocal microscopy fields were analyzed using a specific macro with ImageJ (https://imagej.nih.gov/ij/) software, using CD30 signal to measure the area of shFAT4 cells normalized to shNT. For β-catenin and YAP1 cellular localization, the relative nuclear/cytoplasmic signal intensity was integrated using ImageJ.

### Migration assay

The migration ability of cells was evaluated using Transwell® Permeable Supports (24-well plates, 8 µm pore size, 6.5 mm insert, Corning). One million SUP-M2 cells were placed in the upper chamber in 100 µl RPMI with 0.1% FBS. The lower chamber was filled with RPMI + 0.1% FBS and 0.1 µg/ml SDF-1α as a chemo-attractant. The number of cells present in the lower chamber after 6 h was counted. To account for possible differences in cell seeding, an MTS assay was run on the cell suspension used for initial upper chamber plating. The experiment was performed 5 times, in triplicate.

### Time-lapse microscopy

Cells were seeded in Petri dishes and imaged for 12 h every 10 min in brightfield with phase contrast. Acquisition parameters and post-processing were held constant throughout each experiment. Images were acquired using an inverted wide-field microscope equipped with temperature and CO_2_ control (Zeiss Cell Observer) with a 20x, 0.8 N/A objective. Images were pre-processed for cell detection and analyzed with ImageJ using the TrackMate plugin. TrackMate employs multiple tracking algorithms to link the detected objects across frames, generate tracks and extract quantitative information from the tracks. The total accumulated distance over 12 h was recorded per each cell. Results were then compared using GraphPad Prism for statistical analysis.

### Western blotting

Cells were lysed in Laemmli buffer supplemented with β-mercaptoethanol and boiled at 95° for 10 min. Denatured samples were loaded onto polyacrylamide gels and transferred to nitrocellulose membrane (Amersham^™^ Protran^®^ 0,45 μm NC, GE Healthcare Life Sciences). After blocking, primary antibodies were added overnight at 4 ˚C. Signal was visualized by chemiluminescence using Westar Nova 2.0 reagents (Cyanagen) and a ChemiDoc XRS+ detection system (Biorad) after adding the appropriate HRP-linked secondary antibody. Primary antibodies used in this study were as follows: β-catenin (BDbiosciences, cat# 610154; dilution (dil) 1:1000), phospho-β-catenin (Ser33/Ser37/Thr41; Cell Signaling Technology [CST], cat# 9561; dil 1:1000), YAP1 (Santa Cruz Biotechnology, cat# sc-376830; dil 1:500), phospho-YAP1 (Ser127; CST, cat# 4911; dil 1:1000), STAT3 (CST, clone D3Z2G, cat# 12640; dil 1:1000), actin (Sigma, cat# A2066; dil 1:2000), GAPDH (AbCam, cat# ab9485; dil 1:500), FLAG-tag (Sigma, clone M2, cat# F3165; dil 1:1000), HA-tag (CST, clone C29F4, cat# 3724; dil 1:1000), PRMT5 (CST, clone D5P2T, cat# 79998; dil 1:1000), DBC1 (CST, cat# 5857; dil 1:1000), FAT4 (Novus biological; cat# NBPI-78381; dil 1:150; used for immunohistochemistry following the protocol recommended by the manufacturer).

### Luciferase assays

For luciferase repression assays, RUNX1T1 was cloned in the pBIND vector (Promega) fused to the Gal4 DNA-binding domain. HEK-293T cells were transiently co-transfected with 0.5 µg pBIND-RUNX1T1 constructs, 0.5 µg pG4-TK-Luc plasmid [16] carrying five Gal4 binding sites upstream of a thymidine kinase promoter and the firefly luciferase gene, and 0.1 µg phRL-CMV plasmid (renilla luciferase) for normalization of transfection efficiency. Twenty-four hours post-transfection, cells were lysed and assayed for firefly and renilla luciferase activity using the Dual-Luciferase® Reporter Assay System (Promega) following manufacturer’s protocol. Luciferase activity was measured using a 1450 MicroBeta TriLux luminometer (Perkin Elmer). The firefly reporter gene signal was normalized to renilla luciferase values to account for differences in transfection efficiency. Transcriptional activity of β-catenin in SUP-M2 cells was measured by transfection of cells with 1 µg TopFlash plasmid, containing 6 copies of a TCF4 binding site upstream of the firefly luciferase reporter, and 0.1 µg phRL-CMV. The firefly luciferase signal was detected as above and normalized to renilla.

### Phospho-array

The phosphorylation profile of 37 intracellular kinases was obtained using the Proteome Profiler Human Phospho-Kinase Array Kit (R&D Systems, Minneapolis, MN, USA) according to protocol. Briefly, pre-spotted membranes were incubated with cell lysates (450 μg total proteins) overnight with shaking. The membranes were then washed, incubated with biotinylated antibody cocktail for 2 h at room temperature, washed, incubated with streptavidin-HRP for 30 min, and washed again. Signal was detected by chemiluminescence as described above.

### In vivo analysis

SUP-M2 cells (shNT and shFAT4 clone #1) were injected subcutaneously in the flank of SCID mice. When tumors reached an average volume of 100 mm^3^, the mice were randomized to receive vehicle (0.5% carboxymethylcellulose + 0.1% Tween80) or crizotinib (30 mg/kg daily, by oral gavage) or CHO (cyclophosphamide 40 mg/kg, vincristine 0.2 mg/kg, doxorubicin 3 mg/kg; every three days, by intraperitoneal injection) as described [17]. Tumors were measured every three days with a caliper and volumes were calculated using the formula: Volume (mm^3^) = d^2^ x D/2 where *d* is the shortest and *D* is the longest diameter of the tumor mass in mm units.

### RNA sequencing

Total RNA was isolated from cells using Trizol reagent. Three independent clones were collected per cell line. Libraries were prepared using TruSeq Stranded mRNA kit (Illumina, Milan, Italy) and sequenced at Genewiz (Azenta Life Sciences, Leipzig, Germany) in paired-end mode (2 × 150 bp) at a depth of 20 million read pairs per sample. Fastq files were aligned to the human reference genome (GRCh38/hg38) using HISAT2. Differential gene expression and statistical analysis were run with DESeq2 [18]. Functional enrichment was performed with WebGestalt [19]. GSEA was performed using the normalized counts for each comparison, with 1000 permutations. FDR < 0.1 was considered significant. z-score normalization was calculated using the formula: z = (x-μ)/σ; where x is the original raw value, μ is the mean, σ is the standard deviation of the mean.

### Co-immunoprecipitation

Twenty million HEK-293T cells expressing HA-RUNX1T1, WT or mutated, were transfected with pcDNA3.1 plasmid expressing the FLAG-tagged protein of interest. After 48 h, the cells were lysed in lysis buffer (25 mM Tris-HCl pH 8, 150 mM NaCl, 1% NP-40, 1 mM EDTA, 2 mM EGTA, 10 mM NaF, 10 mM DTT, and protease inhibitors) and incubated 1.5 h with anti-HA affinity resin (Amintra) with rotation. After 4 washes with lysis buffer, the precipitated immunocomplex was resuspended in Laemmli buffer+β-mercaptoethanol for SDS-PAGE loading.

### Mass spectrometry analysis

Fifty million cells were lysed in lysis buffer and processed as above. The immunoprecipitate was eluted with 0.1 M glycine-HCl pH 2.0 for 10 min and then neutralized with 1 M Tris-HCl pH 8.5. Immunoprecipitated proteins were processed with a suspension trapping system (S-trapTM Micro spin columns; ProtiFi, USA) [20] with minor adjustments. Briefly, samples were incubated with 2% SDS and 20 mM dithiothreitol (45 min at 56 °C) and alkylated with 30 mM iodoacetamide (30 min at room temperature). Then the samples were treated with phosphoric acid (1.2%) and binding buffer (100 mM Tris-HCl, pH 7.1, in 90% aqueous methanol) and loaded on micro-columns for protein trapping and overnight incubation at 37 °C with 3 µg trypsin in 50 mM ammonium bicarbonate. Peptides were eluted by sequential centrifugation with 50 mM ammonium bicarbonate and then with 0.2% formic acid (FA). Finally, hydrophobic peptides were recovered with 50% acetonitrile (ACN) containing 0.2% FA. Pooled elution of each sample was vacuum dried and resuspended in loading buffer (H_2_O:ACN:trifluoroacetic acid (TFA) 98:2:0.1). Peptide content was quantified using a Nanodrop spectrophotometer. For each sample, 1 µg of tryptic peptides was injected in duplicate into a Dionex UltiMate 3000 rapid separation LC nanosystem (Thermo Scientific, USA) coupled with an Impact HDTM UHR-qToF system (Bruker Daltonics, Germany). The samples were loaded into a µ-precolumn (Thermo Scientific; Acclaim PepMap 100, 100 µm × 2 cm, nanoViper, C18, 3 µm) for further desalting and concentration and separated in an analytical nanocolumn (Thermo Scientific, Acclaim PepMap RSLC, 75 µm × 50 cm, nanoViper, C18, 2 µm) with a multistep 240 min gradient of nanopump phase B (H_2_O:ACN:FA 20:80:0.08) at a flow rate of 300 nL/min. The eluted peptides were ionised using a nanoBoosterCaptiveSpray™ (Bruker Daltonics) source using heated nitrogen enriched with ACN. The mass spectrometer was operated in Data Dependent Acquisition mode, with automatic switching between full-scan MS and MS/MS acquisition, as described [21]. To improve mass accuracy, the mass spectrometer was calibrated using a mix of ten standards with a known mass (MMI-L Low Concentration Tuning Mix, Agilent Technologies, Santa Clara, CA, USA) before the sample run sequence. In addition, a specific lock mass (1221.9906 m/z) and a calibration segment (at the first 15 min of the gradient) of 10 mM sodium formate (1% NaOH 1 M and 0.1% FA) cluster solution was used. Raw data were deconvoluted using Compass DataAnalysisTM, v.4.1 (Bruker Daltonics, Germany). The resulting file was processed using Peaks Studio X-Plus (Bioinformatics Solutions Inc., USA); the human SwissProt database (released March 2021) was integrated to the search engine. The parameters were set as follows: trypsin as the enzyme, carbamidomethyl as the fixed modification, oxidation (M) as the variable modification, 20 ppm as the precursor mass tolerances and 0.05 Da for the product ions. A false discovery rate (FDR) ≤ 1% at the peptide level was applied to all the analyses and the proteins were considered identified only if they had at least one unique significant peptide (-10lgP ≥ 20).

## Results

### Whole-exome sequencing of systemic/nodal ALCL patient tumors

**Fig. 1:**
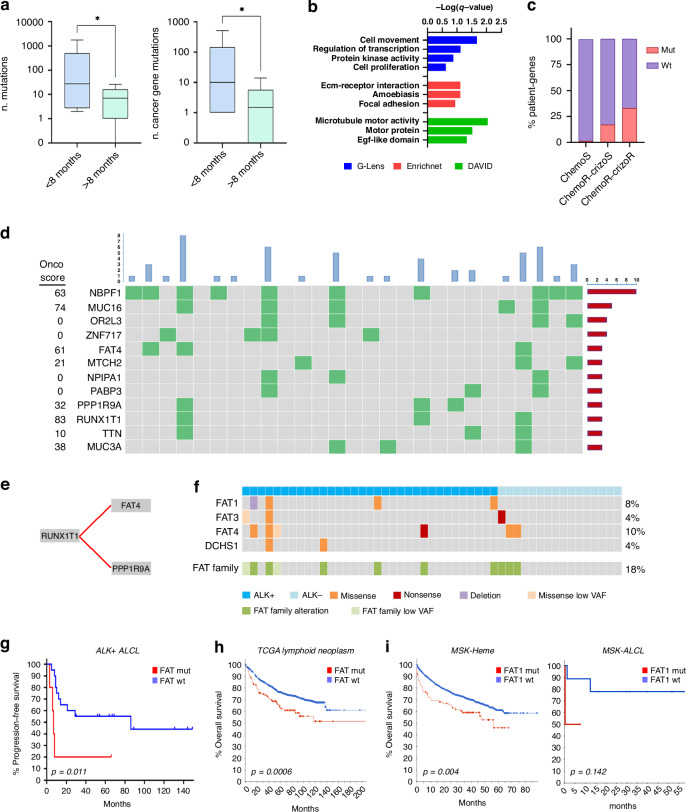
Whole-exome sequencing of ALCL patients. **a** Number of somatic variants (*left*) and cancer-gene mutations (*right*) identified by WES in ALCL patients relapsing early (PFS < 8 months) on first-line chemotherapy and in long responders (PFS > 8 months). Cancer genes were defined as genes with an associated OncoScore >22. **b** Gene ontology enrichment analysis. Significantly enriched terms of the mutated genes, identified by an in-house over-representation algorithm (*blue bars*), Enrichnet (*red bars*) and DAVID (*green bars*). **c** Percent mutated (*red*) *vs* wild-type (*purple*) patient-genes in a panel of 6 cell adhesion-related genes across the cohort of 27 patients, divided in non-relapsed (sensitive, S) and relapsed (R) to chemotherapy and/or crizotinib. **d** Oncoprint showing recurrent somatic mutations ( ≥ 3 occurrences) identified in 27 ALK + ALCL patients by WES. **e** Evolutionary model of repeated evolution inferred by ASCETIC. **f** Chart showing somatic mutations of *FAT* cadherin family genes in 49 ALCL patients. **g** Progression-free survival of ALK + ALCL patients carrying any *FAT* cadherin mutation *vs* non-mutated (WT) patients, in our cohort. **g** Overall survival of patients carrying WT vs mutated *FAT* family genes in TCGA lymphoid tumors. **h** Overall survival of patients from the MSK-Heme project, WT *vs* mutated *FAT1*, in all hematological tumors (*left*) and in the ALCL cohort (*right*). Note that *FAT4* is not represented in the MSK panel. The difference in MSK-ALCL Kaplan-Meier curves is not significant, likely due to the limited number of patients (*n* = 13). Data were retrieved from *cbioportal.org*.

We analyzed whole-exome sequencing (WES) data of lymphoma and matched non-tumoral tissue from 27 ALK + ALCL patients (Supplementary Table S1), using an internally developed pipeline. Mutations showing >25% variant allele frequency (VAF) in the tumor and <10% in the matched healthy sample were called. The analysis confirmed that ALK+ lymphoma carry additional somatic mutations along with the driver translocation event: overall we found a median of 10 (interquartile range (IR) = 2–25) non-synonymous single nucleotide variants (SNVs) per patient (Supplementary Table S2). One hypermutated case displayed >1000 SNVs, among these we noted *MSH2* and *MSH6* somatic substitutions, suggesting mismatch-repair deficiency. Considering only cancer genes (defined as genes with an OncoScore >22 [22]) we observed 4 (IR, 1-10) putative oncogenic variants per patient. Curated clinical data were available for 24 cases: patients who relapsed early (<8 months) on first-line chemotherapy had a higher mutational burden and higher number of cancer genes variants, as reported previously [12, 23, 24] (Fig. 1a). Pathway enrichment analysis identified potential common functions among the mutated genes: a significant overrepresentation of elements related to cell movement was noted using different tools (Fig. 1b). In particular, a *cell adhesion* signature comprising 6 mutated genes (*GPR98*, *ADAM12*, *MMRN1*, *FAT4*, *FAT1*, *FNDC3A*) was associated with sequential failure of both chemotherapy and crizotinib treatments (*p* = 0.003 *vs* all other patients; *p* < 0.0001 *vs* chemo-sensitive patients; Fig. 1c and Supplementary Fig. S1). Interestingly, inspection of germline variations and polymorphisms revealed a strong association of specific HLA-DQB1 alleles with failure of crizotinib therapy (Supplementary Table S3), suggesting a possible effect of the immune system on durable response to this ALK TKI.

To prioritize mutations for further biological validation, we first searched for genes that were recurrently mutated in at least three patients (Fig. 1d). Among these, genes with a low OncoScore, or with a high proportion of variants that are reported in the healthy population (minor allele frequency >1%), were deemed lower priority. The remaining genes were candidates for subsequent analysis. Interestingly, despite the limited number of patients, the ASCETIC framework [25] identified a consistent evolutionary pattern whereby *RUNX1T1* mutations arise early and evolve towards late acquisition of either *FAT4* or *PPP1R9A* mutations (Fig. 1e). No additional regularity was found among the recurrently mutated genes. Unfortunately, it was not possible to assign a temporal ordering with respect to the NPM::ALK rearrangement, as WES is blind to fusions. Cox regularized regression identified *FAT4* mutations as the only feature negatively associated with survival (risk coefficient = 5.65). *FAT4* belongs to the Fat/Dachsous protocadherin family involved in cell adhesion [26]. Of note, another member of the family, *FAT1*, was mutated in two patients (Supplementary Table S2). To expand the analysis, we sequenced all *FAT* family genes by Sanger method in 6 additional samples that were not suitable for WES. In addition, copy number alterations were analyzed in the cohort of 27 WES samples. Finally, available WES data from 16 ALK-negative patients [11] was reanalyzed using our pipeline. In total, we found that the Fat/Dachsous family was altered in 9/49 (18%) ALCL patients (6/33 ALK+ and 3/16 ALK-), including a deep deletion of *FAT1* and mutations of *FAT3* and the FAT ligand *DCHS1* (Fig. 1f). The three ALK-negative cases with *FAT* gene mutations were shown by Crescenzo et al. to carry *JAK1*, *STAT3* and *TP53* driver mutations [11]. By looking at subclonal variants, two more ALK+ patients were found to carry *FAT* genes mutations at low variant allele frequency (VAF, 5% and 6%), which brings the prevalence of *FAT* family alterations to 11/49 (22%) ALCL patients.

In our cohort, ALK+ patients carrying at least one mutated *FAT* family gene had a significantly shorter progression free survival on frontline chemotherapy compared to those with wild-type *FATs*, regardless of treatment regimen (Fig. 1g; median PFS 7 vs 86 months, *p* = 0.011). Interestingly, survival data from The Cancer Genome Atlas (TCGA) indicated a significantly shorter OS for lymphoid cancer patients carrying a mutation in any *FAT*-family gene (Fig. 1h). Similarly, clinical data from the MSK-IMPACT Heme project [27] showed worse survival for *FAT1*-mutated blood cancer patients and *FAT1*-mutated ALCL subgroup compared to non-mutated cases (Fig. 1i), suggesting a general prognostic role of *FAT* genes in hematological cancer.

These results indicate that ALK-rearranged ALCL patients carry additional mutations, frequently related to cell adhesion, and mutations affecting *FAT* proto-cadherins associate with poor survival.

### FAT4 loss induces morphological changes and promotes cell growth and migration

**Fig. 2:**
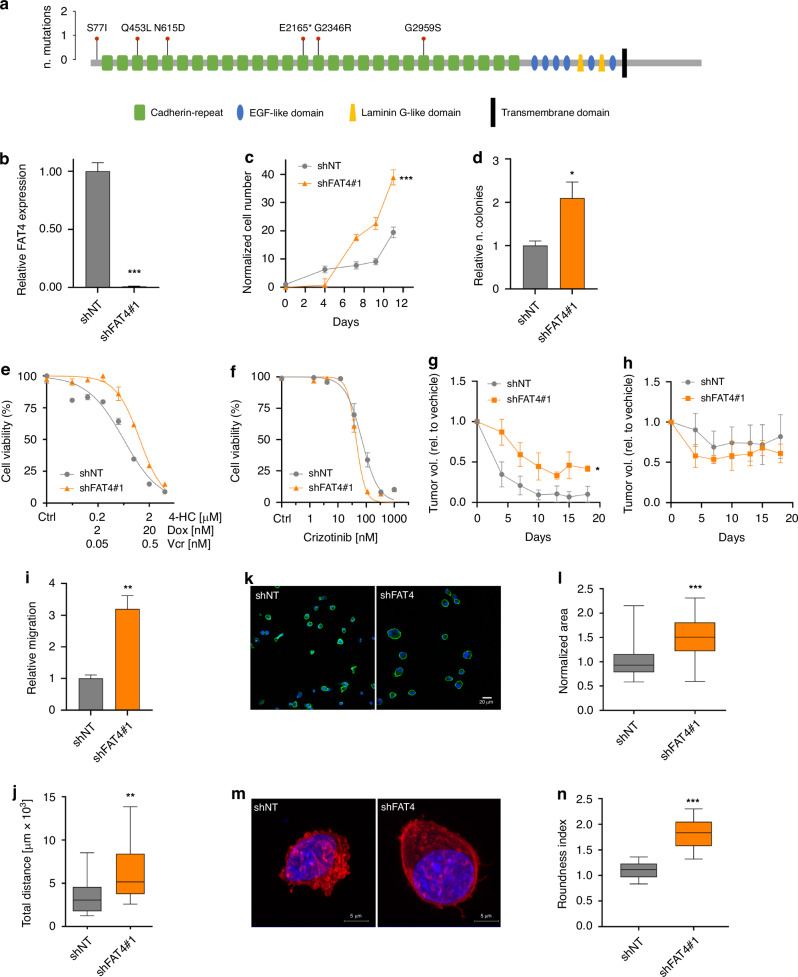
FAT4 mutations in ALCL. **a** Lollipop diagram showing the position of *FAT4* somatic variants. **b** Expression of *FAT4* in SUP-M2 cells expressing non-targeting (shNT) and *FAT4*-targeting (shFAT4, clone #1) shRNA, determined by qPCR. **c** *FAT4* silencing enhances cell proliferation in SUP-M2 cells. **d** Soft agar colony formation ability of control and *FAT4*-silenced cells. Sensitivity of shNT and shFAT4 cells to CHO chemotherapy (**e**) and to crizotinib (**f**) in vitro. Sensitivity of shNT and shFAT4 xenografts to CHO chemotherapy (**g**) and crizotinib (**h**) in SCID mice. Tumor volumes relative to vehicle-treated controls are shown. Migration capability of shNT and shFAT4 cells in transwell (**i**) and time-lapse (**j**) assays. **k** Representative micrographs of shNT and shFAT4 SUP-M2 cells stained with anti-CD30 antibody (green) and with Hoechst (blue) showing larger mean diameter of *FAT4*-silenced cells. **l** Quantification of cell size from images shown in panel (**k**). **m** Representative micrographs of shNT and shFAT4 SUP-M2 cells stained with phalloidin, showing loss of membrane blebbing in shFAT4 cells. **n** Quantification of cell roundness from images shown in panel (**m**) (*n* = 75), where the term ‘roundness’ refers to the absence of surface irregularities. *p < 0.05; **p < 0.01; ***p < 0.001.

Among *FAT* family genes, *FAT4* was most frequently mutated. The observed number of *FAT4* nonsynonymous mutations was more than expected by chance, according to mutation rate analysis [11]. In the expanded cohort, 3 of 5 ALCL patients (3 ALK+ and 2 ALK-) carrying *FAT4* substitutions at high VAF had truncating or damaging variants in highly conserved residues (Supplementary Table S4). All mutations are in the extracellular cadherin repeats (Fig. 2a). One ALK-negative patient carried biallelic *FAT4* mutations. Thus, most of the mutations observed in our patients suggested loss of function, in line with the tumor suppressive role reported in the literature for FAT proteins [28]. Indeed, a search in TCGA database showed that *FAT4* is commonly down-regulated in several cancers (Supplementary Fig. S2A) and lower expression levels of *FAT4* are significantly associated with worse OS in various tumors (Supplementary Fig. S2B). Immunostaining of two wild-type cases from our cohort revealed markedly different FAT4 expression intensities: case 687 showed strong positivity compared to case 2995, which showed a weak signal (Supplementary Fig. S3). From a prognostic point of view, the strongly positive patient 687 had a PFS of 21 months on frontline chemotherapy, whereas low-expressing case 2995 experienced a quick relapse in 7 months, in line with the proposed protective effect of FAT4.

We modeled a loss-of-function scenario by *FAT4* gene silencing in ALCL cells. SUP-M2 cells were chosen as they show the highest *FAT4* expression level among the ALK+ cell lines tested (Supplementary Fig. S4). Stable clones expressing short hairpin RNA (shRNA) against *FAT4* (shFAT4), or a non-targeting shRNA (shNT), were obtained (Fig. 2, clone #1; Supplementary Fig. S5, clones #2 and #3): shFAT4 cells showed near complete suppression of *FAT4* transcript (Fig. 2b). First, we investigated whether loss of *FAT4* had any effect on cell proliferation: *FAT4*-silenced ALCL cells grew significantly faster (*p* = 0.0004) and formed more soft agar colonies (*p* = 0.012) than shNT cells (Fig. 2c, d). We then assessed whether *FAT4* expression affects sensitivity to treatments: shFAT4 cells showed reduced sensitivity to combination chemotherapy (cyclophosphamide, doxorubicine, vincristine [CHO]) [17] while, surprisingly, sensitivity to ALK inhibition by crizotinib was slightly increased (IC_50_ = 44 vs 70 nM; *p* < 0.0001) (Fig. 2e, f). To confirm in vivo these differences in drug sensitivity, shNT and shFAT4 xenografts were grown in SCID mice, that were treated with CHO and crizotinib at suboptimal doses [17]. While a non-significant difference was observed with crizotinib, shFAT4 tumors were markedly less sensitive to CHO treatment compared to shNT (Fig. 2g-h). Next, as FAT cadherins have been involved in the regulation of cell morphology and motility, the ability of shFAT4 cells to migrate was evaluated: a significant increase of migration rate, compared to shNT, was noted in transwell assays (*p* = 0.002; Fig. 2i) and time-lapse imaging (*p* = 0.001; Fig. 2j). Interestingly, shFAT4 cells were on average larger in size compared to shNT cells (*p* < 0.0001; Fig. 2k, l) and showed an altered arrangement of the actin cortex, with less membrane blebs (Fig. 2m, n) consistent with an increased cell spreading and migration speed [29]. Similar results were obtained by two additional shFAT4 clones (Supplementary Fig. S5).

These data indicate that loss of FAT4 modifies the shape and size of ALCL cells and the organization of actin filaments, leading to enhanced cell proliferation and migration. In addition, FAT4 downregulation may reduce the response to chemotherapy in ALCL patients, possibly explaining a worse outcome.

### Down-regulation of FAT4 activates oncogenic signaling and cytoskeleton remodeling program

**Fig. 3:**
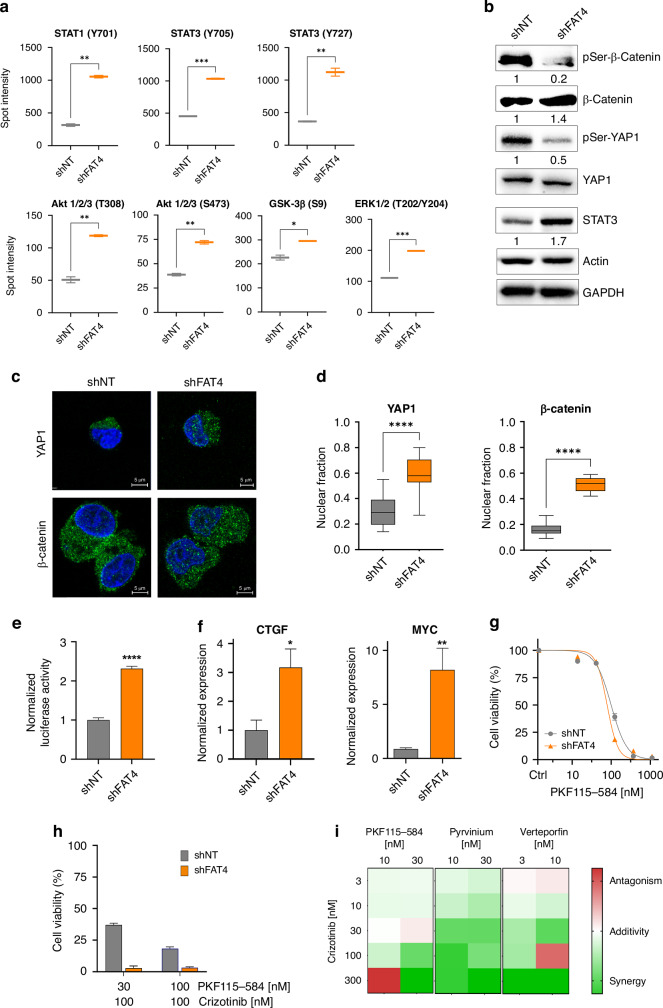
Loss of FAT4 activates YAP1 and β-catenin signaling. **a** Phosphorylation of relevant signaling targets in shFAT4 vs shNT cells, determined by phospho-protein array. **b** Western blot analysis shows simultaneous de-phosphorylation of β-catenin (p-Ser33/37/Thr41) and YAP1 (p-Ser127), and increased STAT3 expression, in shFAT4 cells. **c** Confocal microscopy showing increased β-catenin and YAP1 nuclear localization in shFAT4 cells (green: β-catenin and YAP1; blue: Hoechst). **d** Quantification of the nuclear fraction from the data obtained by confocal microscopy (*n* = 25). **e** TOP-Flash luciferase assay. **f** Expression of the indicated transcripts by qPCR. **g** Sensitivity of shNT and shFAT4 cells to PKF115-584, a blocker of β-catenin/Tcf4 interaction. **h** Effects of crizotinib/PKF115-584 combinations on cells viability. **i** Heatmap of Bliss synergy scores obtained by crizotinib combinations with β-catenin or YAP1 inhibitors, in shFAT4 cells. *p < 0.05; **p < 0.01; ***p < 0.001.

To gain insights into the mechanisms by which *FAT4* silencing affects ALCL cells growth, the activation of various potential oncogenic pathways was studied by phospho-protein array. Activating phosphorylation of STAT1, STAT3, ERK1/2 and AKT, and inhibitory phosphorylation of GSK3β Ser9, were increased, indicating activation of several oncogenic pathways, in shFAT4 cells (Fig. 3a and Supplementary Fig. S6). In line with these data, phosphorylation on Ser33/37/Thr41, which targets β-catenin for degradation, was markedly reduced in shFAT4 cells (Fig. 3b). This was accompanied by a moderate accumulation of total β-catenin. Similarly, the Hippo effector YAP1 was dephosphorylated on Ser127 in shFAT4 cells, indicative of YAP1 nuclear translocation (Fig. 3b). Moreover, STAT3 was upregulated in shFAT4 cells, possibly downstream of β-catenin activation, as previously demonstrated [30]. To confirm involvement of the Hippo and Wnt pathways, the subcellular localization of YAP1 and β-catenin was studied by confocal microscopy: both proteins accumulated in the nucleus, indicating active transcriptional activity, in shFAT4 cells (Fig. 3c, d). These findings were further corroborated by increased activity of the β-catenin-responsive TOPflash reporter and upregulation of two canonical downstream target genes, *CTGF* and *MYC*, in FAT4 knocked down cells (Fig. 3e, f). Next, as shFAT4 cells showed increased β-catenin activity, we asked whether they were sensitive to β-catenin inhibition. Indeed, shFAT4 cells showed higher sensitivity to PKF115-584 [31] (IC_50_ = 79 *vs* 100 nM; *p* = 0.01) and to combined crizotinib/PKF115-584 treatment, compared to shNT cells (Fig. 3g, h). Bliss independence analysis showed that crizotinib interacted synergistically with PKF115-584, as well as with pyrvinium pamoate and with the YAP1 inhibitor verteporfin, to inhibit the growth of shFAT4 cells (Fig. 3i).

**Fig. 4:**
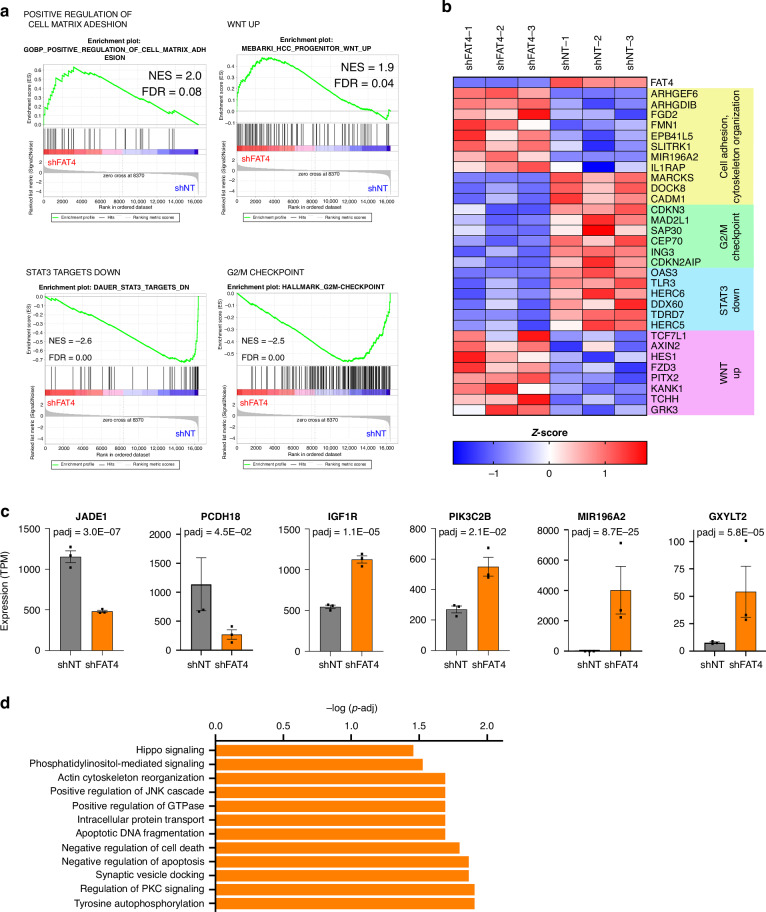
Gene expression profiling of shFAT4 cells. **a** Gene-set enrichment analysis of shFAT4 *vs* shNT cells. NES is the Normalized Enrichment Score; FDR, False Discovery Rate q-value. **b** Heatmap of gene expression signatures from GSEA analysis. **c** Expression of the indicated genes observed by RNA-seq in shFAT4 and shNT cells. **d** Top enriched Gene Ontology (GO) terms in shFAT4, with an adjusted *p* value < 0.05 by Fisher exact test.

Next, transcriptomic analysis of three independent shFAT4 *vs*. three shNT cell clones showed approximately 100 significantly dysregulated genes (Supplementary Fig. S7A). Gene set enrichment analysis (GSEA) identified several top scoring gene sets related to cell adhesion that were significantly enriched in shFAT4 cells (Fig. 4a and Supplementary Fig. S7B), in agreement with the observed modifications of cell morphology, actin filament organization and migration ability of *FAT4*-silenced cells. Indeed, among the most upregulated genes in *FAT4* knockdown cells we found *ARHGEF6*, encoding for the guanine nucleotide exchange factor αPIX that activates Rac1 and Cdc42 GTPases [32–34] and *ARHGDIB*, a negative modulator of the RhoA tumor suppressor [35, 36] (Fig. 4b). Some other genes involved in cytoskeletal organization, such as the actin cross-linking tumor suppressor *MARCKS* [37], were strongly downregulated. In addition, Wnt pathway target genes were upregulated in shFAT4 cells, in line with aberrant activation of β-catenin, while G2/M checkpoint and STAT3-repressed genes [38] were downregulated (Fig. 4a, b). Hypoxia-related gene sets were also found markedly upregulated (Supplementary Fig. S7B). shFAT4 cells displayed altered expression of several additional interesting genes including *IGF-1R* and *PIK3C2B* oncogenes [39, 40], the Wnt negative regulator *JADE-1* [41], proto-cadherin *PCDH18* [42], the Notch1 activator *GXYLT2* [43] and the oncogenic miRNA *MIR196A2* [44], revealing the deployment of an array of diverse intracellular mechanisms that may support lymphoma growth and dissemination (Fig. 4c). Differentially expressed genes were further clustered by gene ontology: tyrosine phosphorylation, regulation of apoptosis, reorganization of actin cytoskeleton and Hippo signaling were among the most significant terms (Fig. 4d).

Altogether, these results suggest that *FAT4* silencing induces significant changes in several processes that are relevant for ALCL biology, among these STAT3 and Wnt/β-catenin pro-tumorigenic signaling, as well as cytoskeletal rearrangement, thus explaining increased proliferation and migration of *FAT4*-silenced cells. Furthermore, our data point to Wnt/β-catenin pathway as a potential therapeutic target in ALCL.

### Mutations in *RUNX1T1* alter transcriptional regulation and enhance cell migration

**Fig. 5:**
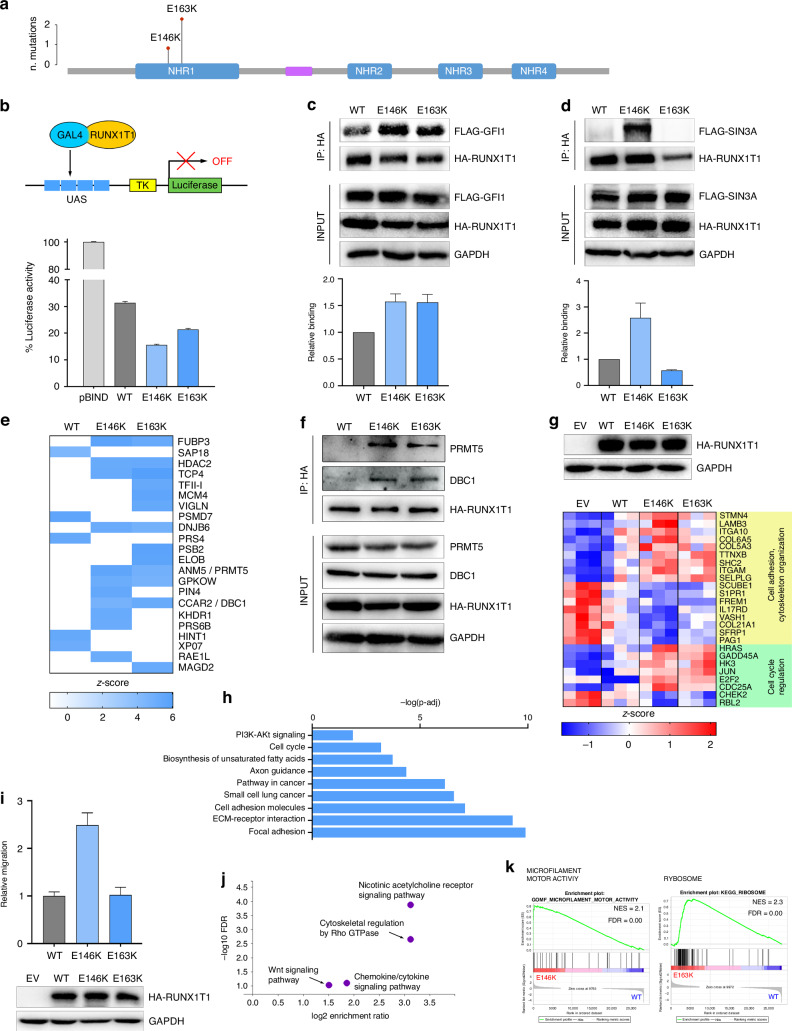
RUNX1T1 mutations in ALCL. **a** Lollipop diagram showing the position of *RUNX1T1* somatic variants. **b** Mutants show enhanced transcriptional repression in luciferase assay. Schematic representation of the experiment is shown on top. **c**, **d** Co-IP experiments showing differential interaction of RUNX1T1 mutants with GFI-1 and SIN3A co-repressors. Densitometry quantification is shown below the bar graphs. **e** Summary heatmap of mass spectrometry analysis results; RUNX1T1 mutants showed an altered interactome compared to the WT protein. **f** Validation of interaction with PRMT5 and DBC1 transcription factors. **g** RNA-seq analysis of HEK-293T cells expressing empty vector (EV), WT, or mutated RUNX1T1 showed significant alteration of cytoskeleton and cell cycle genes expression by the two mutants. Expression of the transfected proteins is shown above the heatmap. **h** GO terms enriched in mutant RUNX1T1 gene expression profiles. **i** Migration of SUP-M2 cells expressing WT or mutant RUNX1T1 in transwell assays. Expression of the transfected proteins is shown below the graph. **j** Volcano plot of significantly over-represented pathways in the transcriptomic profile of SUP-M2 cells expressing RUNX1T1-E146K mutant. **K** Top enriched gene-sets in E146K (left) and E163K (right) mutants *vs* WT RUNX1T1 by GSEA. NES normalized enrichment score, FDR false discovery rate q-value.

The *RUNX1T1* gene, encoding for a transcriptional co-repressor, is involved in hematological disorders via chromosomal rearrangements. *RUNX1T1* was mutated in 3 ALK + ALCL patients, and the mutations were identified as early events according to ASCETIC. The two identified variants (E146K and E163K) are located within the evolutionarily conserved nervy homology region 1 (NHR1), which mediates interaction with several transcriptional factors [45] (Fig. 5a). To evaluate the functional effects of the mutations found in patients, luciferase reporter assays were run using wild-type (WT) and mutated forms of a Gal4-RUNX1T1 fusion protein (Fig. 5b). Both mutants showed enhanced transcriptional repression of the reporter gene, compared to WT protein, suggesting that they may act as gain-of-function mutants (*p* < 0.0001). We then sought to determine whether interaction with known binding partners is affected by the mutations. To this end, HA-tagged RUNX1T1 (WT or mutants) and FLAG-tagged putative interactors were co-expressed in HEK-293 cells and co-immunoprecipitated. RUNX1T1 mutants showed increased binding to the GFI-1 corepressor, while only E146K strongly interacted with Sin3A (Fig. 5c, d). No differences with the WT were found in co-IP experiments with HEB, N-CoR and HDAC1 (data not shown). These results suggested that mutant RUNX1T1 may have an altered interactome that leads to more potent suppression of transcription. To confirm this hypothesis and expand the analysis, a proteomic profiling of RUNX1T1-associated proteins was performed by mass spectrometry: several proteins were found to interact differentially with the two mutants compared to the WT (Fig. 5e). Some of them were chosen for validation by standard co-IP. The arginine methyltransferase PRMT5 and the RNA polymerase II-interacting protein DBC1/CCAR2 were confirmed to interact preferentially with RUNX1T1 mutants (Fig. 5f). *GFI-1*, *Sin3A*, *PRMT5* and *CCAR2* genes were uniformly expressed at high levels also in SUP-M2 cells, thus confirming their potential relevance in lymphoma (Supplementary Fig. S8). Next, RNA-sequencing of stably transfected HEK-293 clones was performed to investigate the effects of mutant RUNX1T1 on gene expression. The results indicated a common dysregulation of several processes linked to cell adhesion and cytoskeleton organization, as well as cell cycle regulation (Fig. 5g, h). Interestingly, the profile of differentially expressed genes indicated that the E146K mutant has a more profound alteration of the transcriptional program: indeed, an increasing distance from empty vector-transfected cells can be seen, following the order Empty > WT > E163K > E146K. Among the top upregulated genes in E146K we noted *ITGA10* (encoding for integrin-α10) and *LAMB3* (laminin beta-3), both associated with migration and invasion in solid tumors [46, 47]. Among down-modulated genes, *S1PR1* and *RBL2* are particularly intriguing: S1PR1 is involved in T cells homing and antagonizes CCR7, whose signaling protects ALCL cells from TKI-induced apoptosis [48]. RBL2 belongs to the retinoblastoma protein family and is repressed by PRMT5 in lymphoma cells [49]. Finally, SUP-M2 cells overexpressing RUNX1T1^E146K^ showed increased motility compared to WT (*p* < 0.0001; Fig. 5i) while no difference was observed in proliferation rate (Supplementary Fig. S9). Transcriptomic analysis of SUP-M2 transfectants showed an over-representation of Wnt and Rho GTPase pathways in the E146K mutant compared to the WT (Fig. 5j) and GSEA again pointed to an alteration of actin cytoskeleton remodeling, confirming previous data (Fig. 5k). Mutant E163K, on the other hand, showed a strong upregulation of ribosomal proteins, suggesting dysregulated ribosome biogenesis in these cells (Fig. 5k), and a down-regulation of STAT3-repressed target genes, similar to shFAT4 cells (Supplementary Fig. S10). Common top differential genes shared by both mutants *vs* WT included a set of positive regulators of migration and invasion that were repressed by the WT form (compared to empty vector-transfected cells) but not by the mutants, suggesting a common loss of repressive function on their promoters. On the contrary, several negative regulators of cell migration were strongly repressed by the mutants but not by WT RUNX1T1, indicating a gain of function in these cases (Supplementary Fig. S11). With the caveat that transfected cells express supraphysiological levels of RUNX1T1, we conclude that a complex change of RUNX1T1 function may be caused by the mutations in SUP-M2 cells. It remains to be seen whether these genes are direct RUNX1T1 targets.

Overall, our data suggest that mutated RUNX1T1 leads to significant changes in gene expression, likely by an altered interaction with nuclear transcription factors, to modify the organization of the cytoskeleton and promote cell migration.

## Discussion

In the past, it was suggested that deregulation of a number of genes may co-exist or even precede chromosomal rearrangement in ALCL [50]. In this study we investigated the presence of somatic mutations, co-occurring with ALK fusions, that may tweak the phenotype of ALK + ALCL. The analysis was further extended to ALK-negative ALCLs which, while lacking ALK rearrangement, often carry other alterations that cause downstream STAT3 activation [11, 13]. *NBPF1* was the most frequently mutated gene in our cohort; however, all the detected variants, albeit confirmed somatic, are frequent polymorphisms in the healthy population (MAF > 1%), suggesting that they likely represent passenger variants with no role in the biology of the lymphoma. In addition, we found recurrent somatic mutations in the protein phosphatase 1 regulatory subunit 9 A (PPP1R9A), in FAT cadherins, and in the RUNX1T1 transcriptional repressor, suggesting their possible involvement in shaping the phenotype of lymphoma cells. We previously investigated the role of phosphatases in ALK + ALCL [51]. *FAT* family genes were altered in 18% of ALCL patients at clonal frequency, 22% when including subclonal mutations. Mutations were damaging or nonsense, in line with a tumor suppressor function. Similarly, *FAT4* and *DCHS1* mutations in Hennekam syndrome and Van Maldergem syndrome patients, two genetic diseases that involve developmental defects in cell migration, are believed to cause a loss of function [52, 53]. Notably, the CCLE database reports missense variants in two ALK + ALCL cell lines, Karpas-299 and SU-DHL-1. A recent preprint describes *FAT4* mutations in 6/49 T-cell lymphomas, including ALCL and PTCL-NOS [54].

FAT cadherins regulate several cellular processes, including the maintenance of planar cell polarity, modulation of Hippo signaling and actin polymerization [26, 28]. Loss of the *fat* gene in Drosophila leads to dramatic tissue overgrowth [55] while human FAT proteins have been involved in cancer, acting as tumor suppressors [56–59]. In particular, *FAT4* is frequently mutated or downregulated in several types of human cancer [60–65]. In lung cancer patients, low expression of *FAT4* associates with short survival, while its overexpression inhibits growth and migration of lung cancer cells and suppresses metastasis [66].

**Fig. 6:**
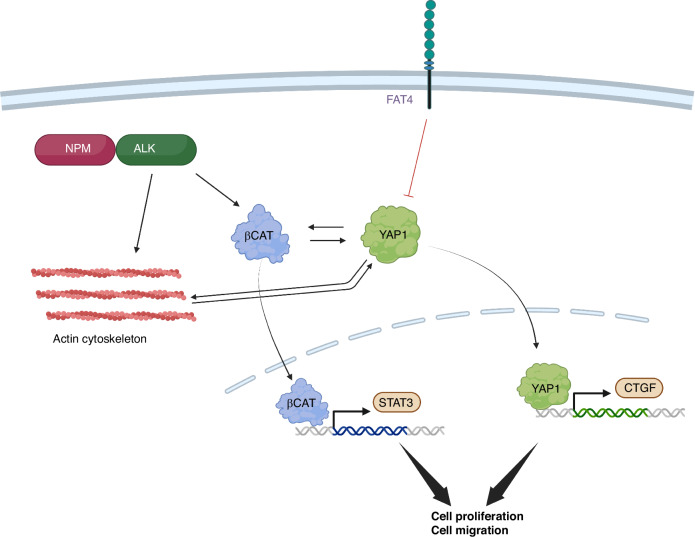
Schematic overview of the findings. NPM::ALK fusion kinase activates β-catenin signaling and induces cytoskeleton remodeling. Loss of FAT4-mediated inhibition activates YAP1, which potentiates both F-actin and β-catenin effects. YAP1 is itself further activated by β-catenin and cell motility, in a positive loop that leads to enhanced cell growth and migration. Created with BioRender.

The FAT4 tumor suppressor has been shown to restrain tumor growth through modulation of Hippo and Wnt/β-catenin pathways [26, 67]. Our results indicate that, in ALCL, loss of FAT4 promotes cell proliferation and migration, primarily by hyper-activation of YAP1 and β-catenin, as well as STAT3, and by modulation of actin dynamics. All these actions are likely interconnected, as YAP1 and β-catenin have been shown to regulate each other reciprocally [68, 69] and β-catenin was shown to increase STAT3 activation [70]. In addition, YAP1 activity is regulated by F-actin cytoskeleton and small GTPase signaling, and in turn regulates mechanical signaling and F-actin remodeling, in a positive feedback loop that promotes cell adhesion and motility [71]. *FAT4* mRNA expression is itself regulated by actin dynamics [72]. Activation of YAP1 and β-catenin may provide ALCL cells with a ‘persister’ phenotype, allowing survival against early treatment, until a fully resistant clone arises [73]. We propose that FAT4 loss may be one mechanism that orchestrates all these changes in some patients, cooperating with ALK fusions in ALK+ cases and with the other main oncogenic drivers in ALK-negative patients (Fig. 6). Interestingly, the observed partial resistance to chemotherapy, while maintaining sensitivity to crizotinib and PKF115-584, suggests that patients with FAT4 mutations may be addressed directly to frontline TKI therapy and that FAT4 loss might be exploited therapeutically in the future, using combined ALK/β-catenin blockade. It is hard to tell why FAT4 loss should alter cells sensitivity to drugs. Resistance to chemotherapy may derive from their ability to activate YAP1 and β-catenin, that have been shown to be protective against various insults [73–75]. The observed regulation of cytoskeletal gene expression is particularly intriguing, as NPM::ALK has been shown to control ALCL cell morphology and motility through Rho GTPases-mediated cytoskeleton remodeling, giving the cells the typical anaplastic appearance [32, 34, 76]. FAT4 mutations may potentiate this effect, contributing to an aggressive phenotype and ensuing diminished sensitivity to therapies. Notably, FAT4 and FAT1 proteins have been found to interact directly with Rho/Rac GTPases in human cells [77]. A ‘cytoskeleton remodeling’ signature was also enriched globally in the entire landscape of somatic co-mutations in patients, suggesting that *FAT* genes are not the only players in this process. Indeed, previous analyses identified a *cell migration/focal adhesion* gene expression profile to be specifically associated with ALK + ALCL [78] and further enriched in relapsed patients [48]. Interestingly, several *ALK* fusions involve cytoskeletal proteins as the N-terminal partner, including *TPM3, TPM4, CLTC1, EML4, LMNA* [4].

*RUNX1T1* is better known as the fusion partner of *RUNX1* in leukemias carrying the t(8;21) translocation [79]. The encoded protein is a transcriptional co-repressor [80, 81] composed of four evolutionarily conserved NHR domains, mediating interactions with several nuclear factors, and a nuclear localization signal [82]. NHR1, also referred to as TAFH, is a hub for interaction with several transcription regulators and is required for transforming ability of *RUNX1/RUNX1T1* fusion in myeloid leukemia [45]. We identified two identical substitutions (Glu to Lys) in 3 ALK+ patients. The change is remarkable, as it causes insertion of a positively charged amino acid in place of a negative one. This may have a marked impact on protein interactions: Glu^163^ belongs to a highly conserved ‘EEF’ tripeptide involved in TAFH-HEB binding [83]. We noted an increased binding of mutants to known RUNX1T1 binding partners such as Sin3A and GFI-1 and identified a differential interaction map that included two transcription regulators that have not previously been associated to RUNX1T1, namely PRMT5 and DBC1. PRMT5 is a histone methyltransferase that suppresses transcription of several tumor suppressors and cell cycle inhibitors and promotes Wnt/β-catenin signaling and invasion [49, 84–87]. Deletion or pharmacological inhibition of Prmt5 improved survival of a mouse *AML1-ETO* leukemia model and inhibited the growth of AML patient-derived xenografts [88]. An augmented repressive potency of RUNX1T1 mutants may be due to increased binding to PRMT5. DBC1/CCAR2 is a multi-function protein involved in several processes, from DNA repair to apoptosis and cell metabolism [89]. DBC1 acts as a negative regulator of BRCA1-dependent transcription, and a positive regulator of β-catenin transcriptional activity, suggesting a pro-tumorigenic role [90, 91]. From our data, we speculate that DBC1 may recruit mutant RUNX1T1 to different transcriptional complexes and redirect its activity to a different set of promoters, leading to an aberrant transcriptional profile. The consequences of this altered activity are visible as modified transcriptional profiles of cells expressing the mutants, once again pointing to cell adhesion and cytoskeleton reorganization, particularly for the E146K mutant cells, which indeed were found to migrate faster than normal. Interestingly, RUNX1T1 was shown to regulate motility and tube forming ability of endothelial cells, supporting its potential involvement in migration and invasion of lymphoma cells [92].

In conclusion, we identified co-mutations that occur alongside the canonical driver alterations in ALCL patients. These mutations may be selected as fine modifiers of the phenotype in some patients, promoting disease progression and resistance to therapy.

## Supplementary information

Supplementary Information

## Data Availability

the data used to support the findings of this study are available from the corresponding author upon request. Raw NGS data are available at NCBI’s Sequence Read Archive, under projects PRJNA1166097 (WES), PRJNA1167917 (RNA-seq of RUNX1T1-transfectants) and PRJNA1168113 (RNA-seq of shFAT4 *vs*. shNT cell clones).
