# Supplementary material for: Recurrent somatic mutations of FAT family cadherins induce an aggressive phenotype and poor prognosis in anaplastic large cell lymphoma
Source: Br J Cancer. 2024 Oct 30;131(11):1781–95. doi: 10.1038/s41416-024-02881-7 (PMC11589140; doi:10.1038/s41416-024-02881-7)
Supplement: Supplementary file 1 — Supplementary Information [file 41416_2024_2881_MOESM1_ESM.pdf]

Supplementary Information

Recurrent somatic mutations of FAT family cadherins induce an aggressive phenotype and poor prognosis in anaplastic large cell lymphoma.

Matteo Villa, Geeta G. Sharma, Federica Malighetti, Mario Mauri, Giulia Arosio, Nicoletta Cordani, Cosimo Lobello, Hugo Larose, Alessandra Pirola, Deborah D'Aliberti, Luca Massimino, Lucrezia Criscuolo, Lisa Pagani, Clizia Chinello, Cristina Mastini, Diletta Fontana, Silvia Bombelli, Raffaella Meneveri, Federica Lovisa, Lara Mussolin, Šárka Pospíšilová, Suzanne D. Turner, Giorgio Inghirami, Fulvio Magni, Mario Urso, Fabio Pagni, Daniele Ramazzotti, Rocco Piazza, Roberto Chiarle, Carlo Gambacorti-Passerini and Luca Mologni

List of Supplementary files:

Supplementary Figure S1 – Mutation matrix of cell adhesion genes

Supplementary Figure S2 – *FAT4* in TCGA tumors

Supplementary Figure S3 – *FAT4* immunostaining on FFPE biopsies

Supplementary Figure S4 – Expression of *FAT4* in ALCL cell lines

Supplementary Figure S5 – Validation of *FAT4* loss in two independent clones

Supplementary Figure S6 – Phospho-array profiling

Supplementary Figure S7 – RNA-seq of shFAT4 vs shNT

Supplementary Figure S8 – Expression of RUNX1T1 interactors in SUP-M2

Supplementary Figure S9 – Cell growth of RUNX1T1 mutants

Supplementary Figure S10 – GSEA analysis of RUNX1T1 mutants

Supplementary Figure S11 – Common DEGs of RUNX1T1 mutants

Supplementary Table S1 – Patients' characteristics

Supplementary Table S2 – List of somatic variants

Supplementary Table S3 – Germline DQB1 variants

Supplementary Table S4 – Functional analysis of FAT variants

Supplementary Figures

GENES

PATIENTS		Pt ID	*GPR98*	*ADAM12*	*MMRN1*	*FAT4*	*FAT1*	*FNDC3A*
chemoR	crizoR	3953	MUT	MUT	WT	MUT	CNL	WT
chemoR	crizoR	10752	WT	WT	MUT	WT	MUT	MUT
chemoR	crizoR	9667	WT	WT	WT	LOW VAF MUT	WT	WT
chemoR	crizoS	687	WT	WT	WT	WT	WT	WT
chemoR	crizoS	I2010	WT	WT	WT	WT	WT	WT
chemoR	crizoS	E2013	WT	WT	WT	WT	WT	WT
chemoR	crizoS	K18	WT	WT	WT	WT	WT	WT
chemoR	crizoS	6548	MUT	WT	MUT	MUT	MUT	MUT
chemoS		2v3	WT	WT	WT	WT	WT	WT
chemoS		75v74	WT	WT	WT	WT	WT	WT
chemoS		B2	WT	WT	WT	WT	WT	WT
chemoS		B3	WT	WT	WT	WT	WT	WT
chemoS		B4	WT	WT	WT	MUT	WT	WT
chemoS		B5	WT	WT	WT	WT	WT	WT
chemoS		B6	WT	WT	WT	WT	WT	WT
chemoS		B7	WT	WT	WT	WT	WT	WT
chemoS		6v7	WT	WT	WT	WT	WT	WT
chemoS		67vs66	WT	WT	WT	WT	WT	WT
chemoS		H4	WT	WT	WT	WT	WT	WT

		TOT	WT	MUT	%m	%wt
chemoR	crizoR	18	10	8	44%	56%
chemoR	crizoS	30	25	5	17%	83%
	chemoS	66	65	1	1,5%	98%

Figure S1. Mutation matrix of cell adhesion genes. The table shows mutated (MUT) and non-mutated (WT) '*cell adhesion'* genes (columns) in each patient's tumor (rows). Patients are grouped according to sensitivity to treatments: chemoS = ongoing response to first-line chemotherapy; chemoR = relapsed on chemotherapy; crizoS = ongoing response to crizotinib; crizoR = relapsed on crizotinib. The small table on the right reports the total number of patient-genes (i.e., cells of the matrix) in each group (TOT), as well as the count and percent of WT and mutated cells.

A

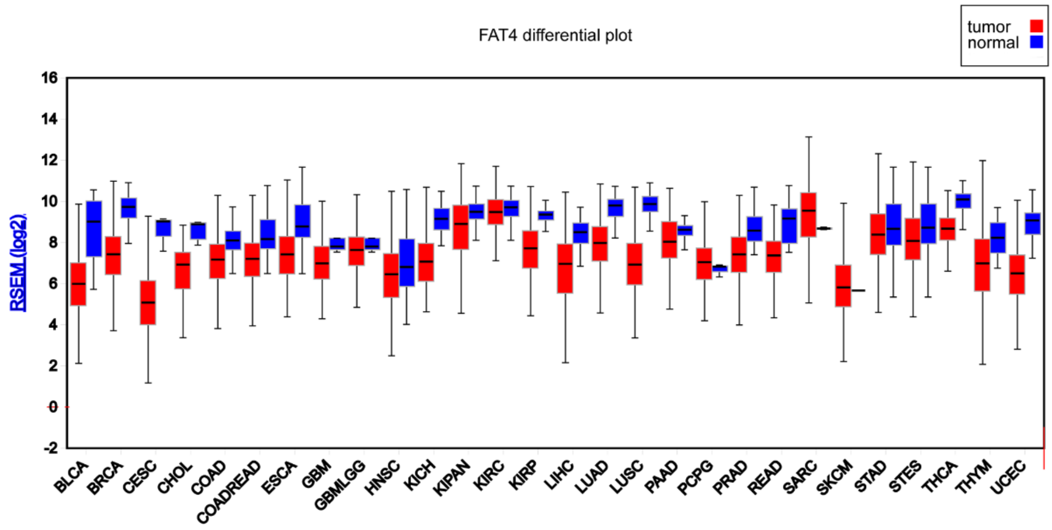

B

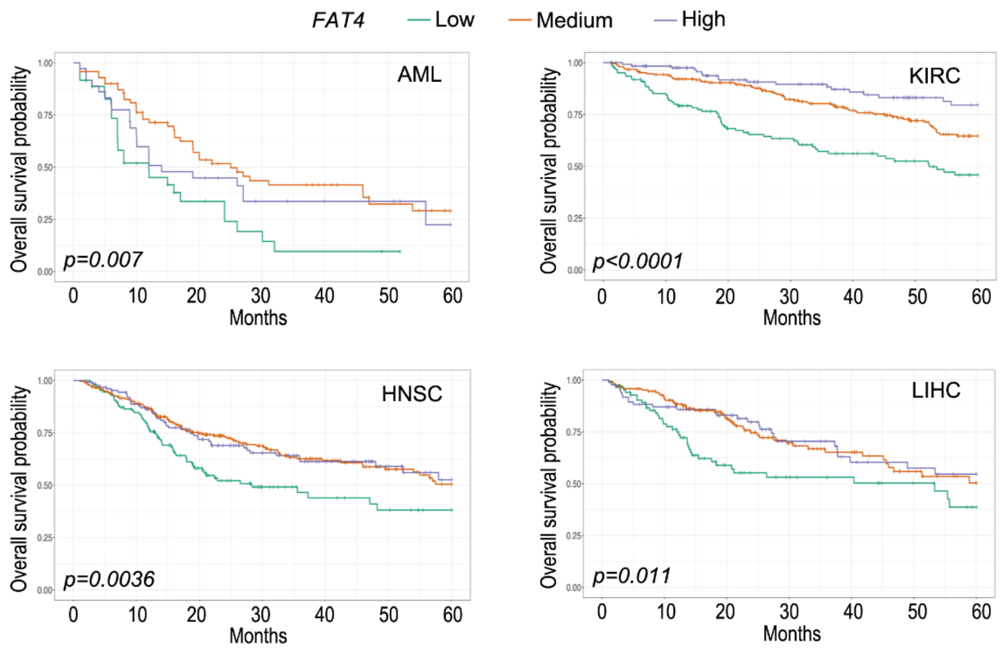

Figure S2. *FAT4* in TCGA tumors. (**A**) Expression of *FAT4* in different tumors and corresponding normal tissues from TCGA database. (**B**) Kaplan-Meier analysis of patients' survival according to *FAT4* expression tertiles (low, medium, high) in acute myeloid leukemia (AML), kidney renal clear cell carcinoma (KIRC), head and neck squamous cell carcinoma (HNSC) and liver hepatocellular carcinoma (LIHC); log-rank p-values are indicated.

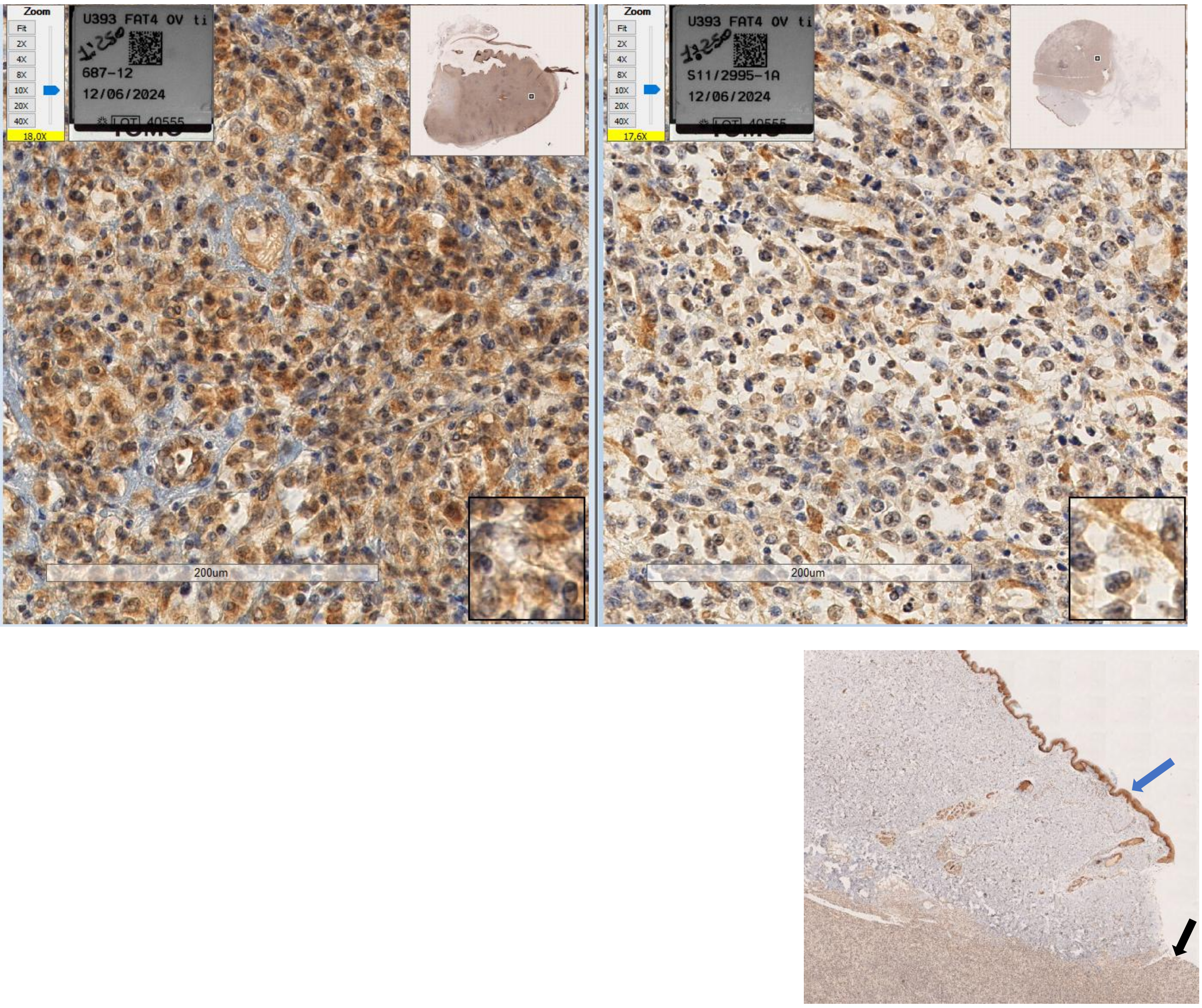

Figure S3. Expression of FAT4 protein in two ALCL patients. *Top*: FAT4 expression was studied by immunohistochemistry in two ALCL cases using a rabbit polyclonal antibody (NBPI-78381; Novus biological, Littleton, CO, USA). Case 687 (*left*) is an ALK+ lymphoma from a cervical lymph node; case 2995 (*right*) is an ALK+ lymphoma in soft tissues. Top-right insets in each image show the entire sample at low magnification. *Bottom*: notably, in 2995, there was a difference in staining between the spinous layer of the skin (blue arrow) and the underlying tumor mass (black arrow). *Note*: patient 2995 was not sequenced by WES due to poor quality of the DNA library.

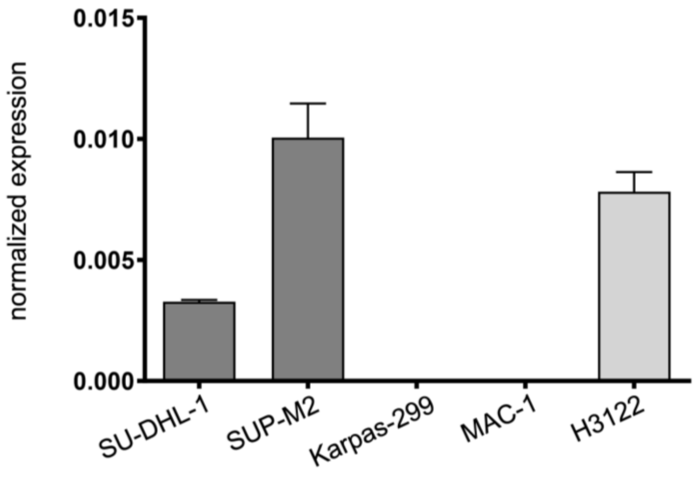

Figure S4. Expression of *FAT4* in ALCL cell lines. Expression of the *FAT4* gene was detected by Taqman qPCR assay in three ALK+ ALCL (SU-DHL-1, SUP-M2, Karpas-299), one ALK- cutaneous ALCL (MAC-1) and one ALK+ lung cancer (H3122) cell lines. Data are normalized to GUS expression.

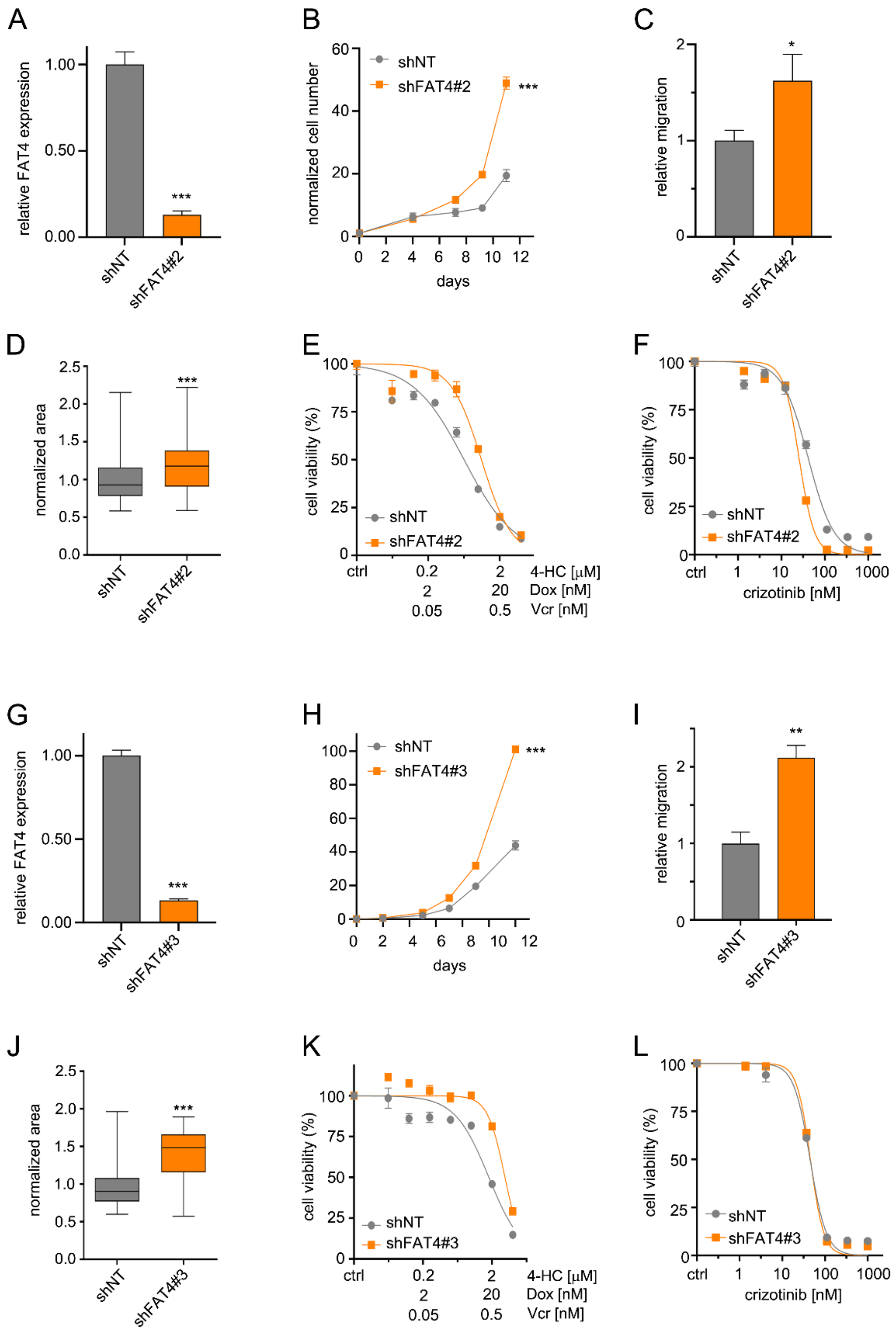

Figure S5. Validation of biological effects of *FAT4* loss in two independent clones. SUP-M2 shFAT4 clone #2 (**A-F**) and clone #3 (**G-L**) were evaluated for expression of *FAT4* (**A**, **G**), cell proliferation (**B**, **H**), migration (**C**, **I**), cell size (**D**, **J**) sensitivity to chemotherapy (**E**, **K**) and to crizotinib (**F**, **L**) in comparison to shNT cells.

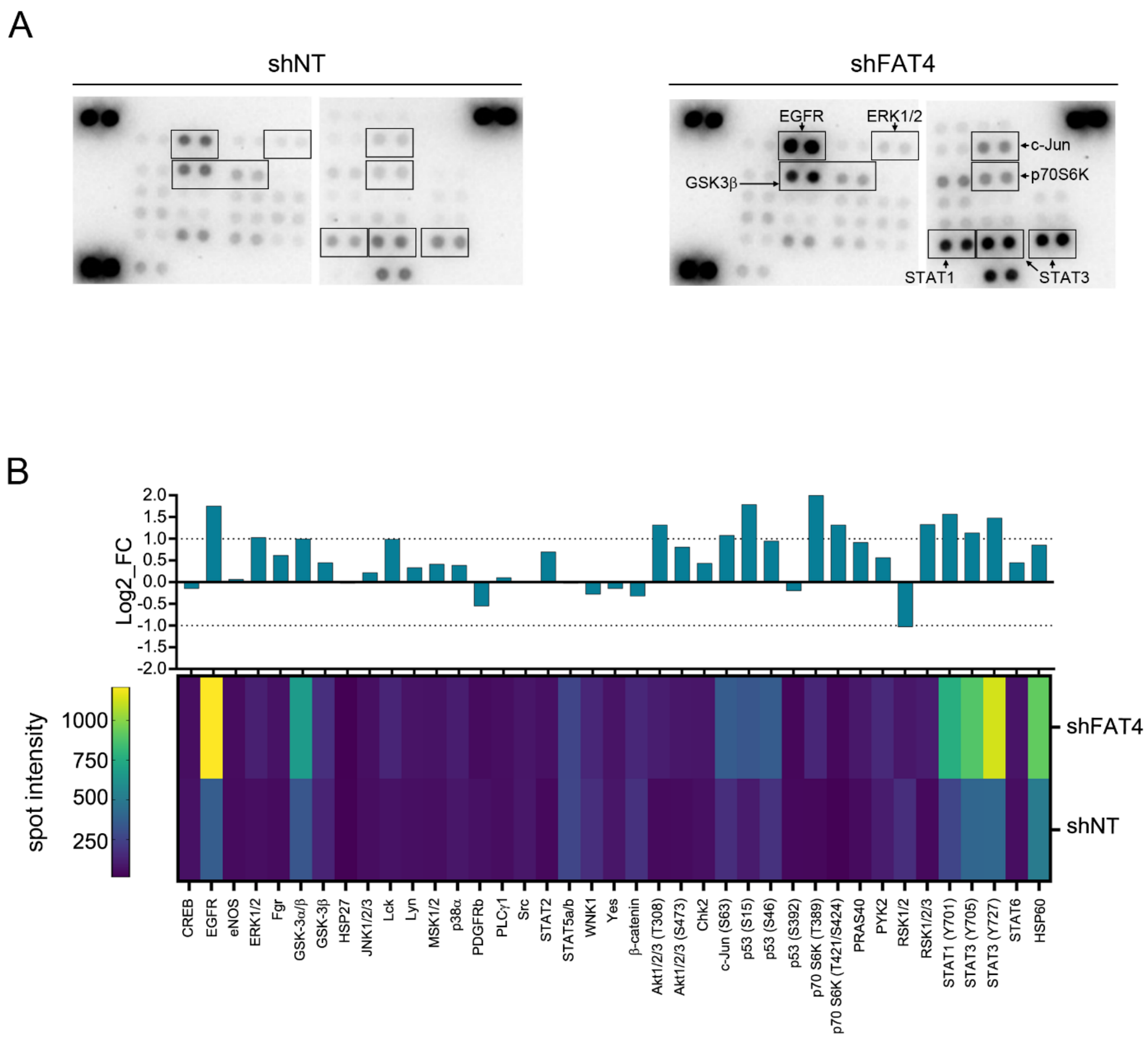

Figure S6. Phospho-array profiling of downstream pathways in shFAT4 cells. (**A**) Hyper-phosphorylation of the indicated proteins in shFAT4 cells shown on phospho-array membranes. The most differential phospho-protein spots are framed and indicated by arrows. Positive (reference) and negative control spots are in the corners of the membranes. (**B**) Heatmap of phospho signal analyzed by spot densitometry. Log_2-transformed fold change of spot intensity in shFAT4 *vs* shNT cells is shown above.

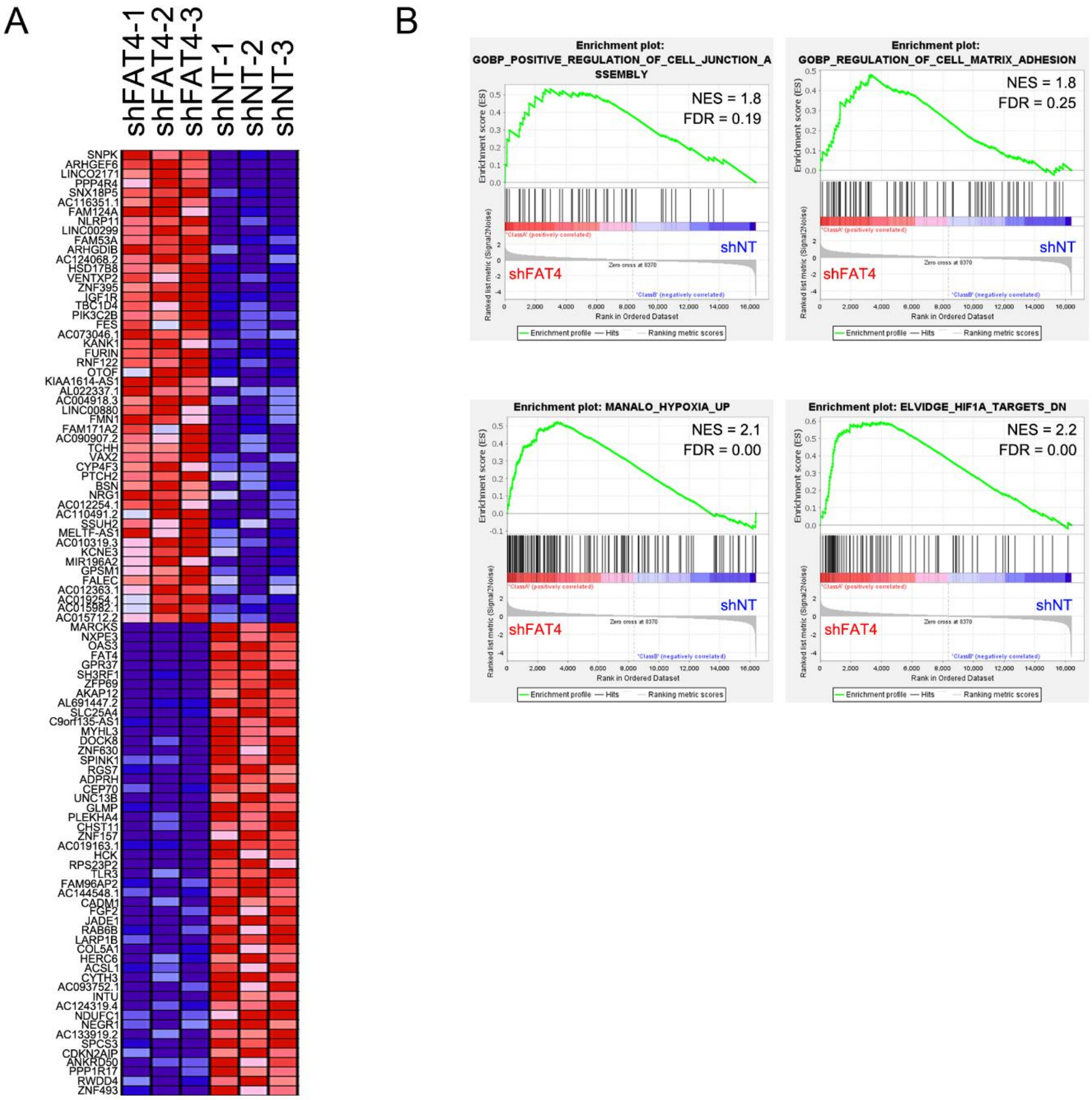

Figure S7. RNA-seq of shFAT4 *vs* shNT. (**A**) Heatmap of top significant differential genes in shFAT4 *vs* shNT RNA-seq profiles. (**B**) Additional enriched gene sets in shFAT4 *vs* shNT cells. NES, Normalized Enrichment Score; FDR, False Discovery Rate q-value.

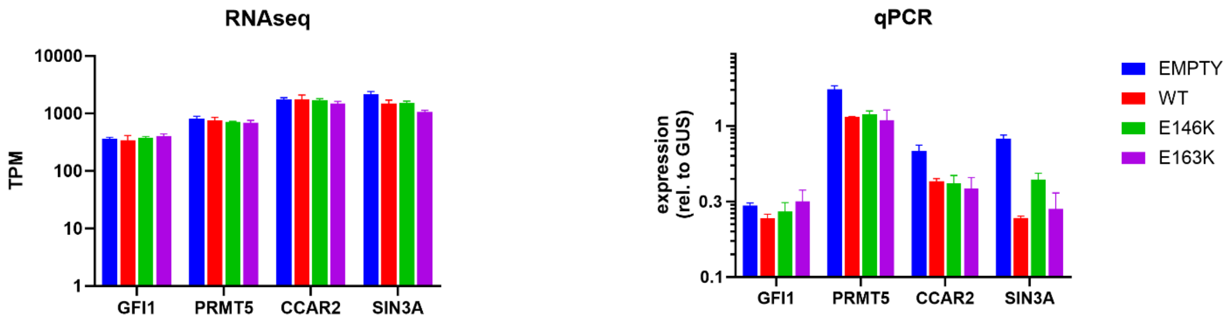

Figure S8. Expression of RUNX1T1 interactors in SUP-M2 transfectants. Expression of the indicated genes is shown, as determined by RNA-sequencing (*left*) and further validated by quantitative real-time PCR using *GUS* as a housekeeping gene (*right*).

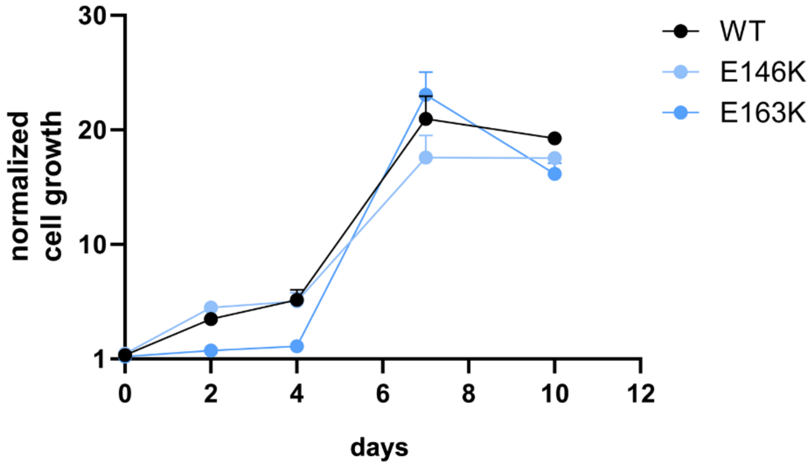

Figure S9. Time-course analysis of RUNX1T1 mutants cell growth. SUP-M2 cells expressing E164K and E163K mutants, or WT RUNX1T1 were followed over 10 days, proliferation was measured by MTS assay and normalized to day 0.

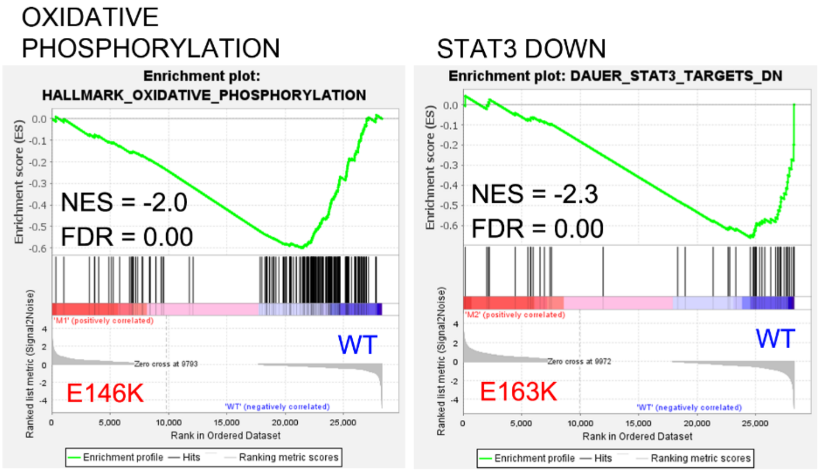

Figure S10. GSEA analysis of RUNX1T1 mutants. Top down-regulated gene sets identified in cells expressing E146K (*left*) and E163K (*right*) RUNX1T1 mutants, compared to WT RUNX1T1-transduced cells. Notably, suppression of oxidative phosphorylation was shown to support tumor cell plasticity and adaptation (Davies A., *Cancer Discov* (2023) 13 (8): 1771–1788).

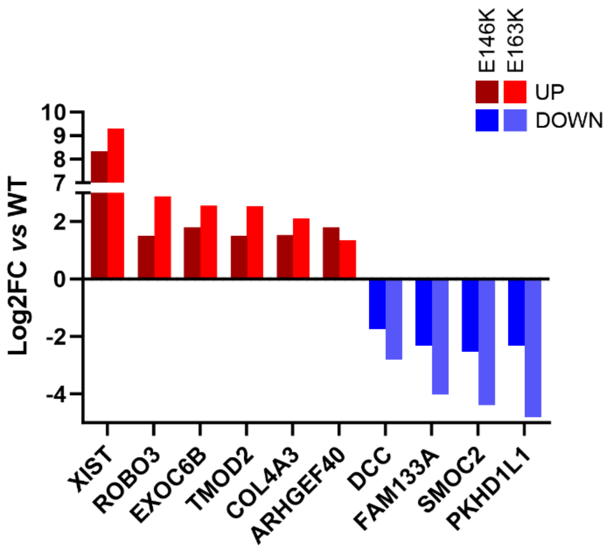

Figure S11. Common top DEGs shared by the two RUNX1T1 mutants *vs* WT in SUP-M2 cells. Up-regulated genes (red bars) are in fact suppressed by the WT but not by the mutants; on the contrary, down-regulated genes (blue bars) are repressed by the mutants.

Supplementary Tables

Supplementary Table S1. Patients' characteristics.

Patient	Sample ID	Type[a]	Age	Gender	Date Diagnosis	Histologic Pattern[b]	1st line[c]	PFS 1st line (months)[d]	2nd line[c]	TKI	PFS crizo (months)[d]	BMT	Death	Last F-up	FAT mut[e]	OS (months)[d]	Number of variants	Number of driver variants
1	687	FFPE	50	F	mar-12	common	COMP	21	GEMOD +BV	CRIZO	11+	N	N	nov-17	*FAT3*	70+	6	1
2	3953	FFPE	35	F	feb-15	common	CHOP	6	DHAP	CRIZO	1	N	Y	ott-15	FAT4	8	25	10
3	E2013	FFPE	16	M	mag-12	common	AIEOP LNH-92	10	CRIZO	CRIZO	57+	N	N	gen-18	-	69+	17	5
4	H6548	FFPE	57	M	ago-16	common	CHOP	7	CRIZO	CRIZO	10+	N	N	feb-18	FAT1,3,4	19+	1722	518
5	9667	FFPE	22	F	gen-10	common	BFM	8	BTZ	CRIZO	2	Y	Y	dic-10	*FAT4*	12	1	0
6	I2010	FFPE	30	F	lug-10	common	VACOP-B	86	BV	CRIZO	12+	Y	N	lug-19	-	110+	2	1
7	K18	FFPE	25	M	ago-17	common	CHOP	9	DHAP	CRIZO	18+	N	N	dic-19	-	28+	79	24
8	H4	FF	8	M	set-01	common	ALCL99	144+	-	-	-	N	N	lug-13	-	144+	14	6
9	B7	FFPE	61	M	mar-11	common	CHOP	89+	-	-	-	N	N	giu-18	-	89+	10	2
10	H5	FF	6	F	gen-02	common/ SC	ALCL99	14	-	-	-	N	N	dic-21	-	242+	11	4
11	GPS 56-57	FF	na	na	na	na	na	na	na	na	-	na	na	na	DCHS1	na	85	25
12	GPS 66-67	FF	22	M	giu-07	na	CHOP	54+	-	-	-	N	N	nov-11	-	54+	0	0
13	B3	FFPE	44	M	ago-07	common	CHOEP	131+	-	-	-	N	N	mag-18	-	131+	55	14
14	GPS 70-71	FF	21	F	nov-97	na	Chemo+ Radio	29	-	-	-	N	N	mag-09	-	140+	0	0
15	GPS 74-75	FF	13	M	mar-11	na	CHOEP	52+	-	-	-	N	N	giu-15	-	52+	1	0
16	GPS 89-90	FF	81	F	feb-05	na	na	na	na	na	-	na	na	na	-	na	2	1

17	GPS 25-26	FF	33	F	ott-10	na	na	na	na	na	-	N	N	na	-	na	12	5
18	10752	FFPE	24	M	ago-16	common	CHOEP+ BV+IEV	3	MTX+ ARAC	CRIZO	3*	Y	N	mag-17	FAT1	9+	30	10
19	GPS 27-28	FF	56	F	ott-06	na	CHOP	12	-	-	-	N	Y	lug-08	-	21	0	0
20	2v3	FF	10	F	lug-10	common/ LH	ALCL99	69+	-	-	-	N	N	feb-16	-	69+	17	7
21	4v5	FF	16	F	feb-09	common	ALCL99	9	na	-	-	N	N	lug-15	-	78+	4	1
22	6v7	FF	8	F	lug-08	common	ALCL99	67+	-	-	-	N	N	feb-14	-	67+	1	0
23	8v9	FF	15	M	mag-10	LH	ALCL99	5	na	-	-	N	N	set-15	-	65+	3	1
24	B4	FFPE	30	F	ott-12	common	CHOP	66+	-	-	-	N	N	mar-18	FAT4	66+	14	6
25	B5	FFPE	46	M	nov-12	LH	CHOP/ HDMTX	64+	-	-	-	N	N	feb-18	-	64+	12	5
26	B6	FFPE	58	M	set-15	common	CHOP	30+	-	-	-	N	N	mar-18	-	30+	26	12
27	B2	FFPE	21	M	mag-06	SC	CHOEP	149+	-	-	-	N	N	lug-18	-	149+	8	3

[a] FF = fresh frozen; FFPE = formalin-fixed paraffin-embedded
[b] SC = small cell; LH = lymphohistiocytic
[c] BV = brentuximab vedotin; IEV = ifosfamide, epirubicin and etoposide; HDMTX = high-dose methotrexate; BFM = Berlin-Frankfurt-Münster protocol; BTZ = bortezomib
[d] in survival data, a plus sign ("+") indicates an ongoing response (PFS) or patient alive (OS) at the data cutoff date (censored)
[e] low VAF variants are indicated in italic
[*] molecular relapse by qPCR

abbreviations:
TKI = tyrosine kinase inhibitor
BMT = bone marrow transplant
PFS = progression-free survival
OS = overall survival

Supplementary Table S2. Somatic variants.

Patient ID	Chr.	Position	Ref	Var	Codon Change	Gene	AA Change	dbSNP	MAF	Coverage (Tumor)	Mut Reads (Tumor)	%Mut (Tumor)	Coverage (Ctrl)	Mut Reads (Ctrl)	%Mut (Ctrl)	Descr. Pvalue	Onco Score
**687**	chr2	97152523-97152524	G	T	GCT->TCT	**ANKRD36**	Ala114Ser; Ala395Ser		na	89	24	27,0	75	6	8,0	0,000	0
**687**	chr2	96023180-96023181	G	A	CGC->TGC	**GPAT2**	Arg511Cys; Arg494Cys; Arg692Cys;	rs201647131	0,053	43	17	39,5	120	11	9,2	0,000	6
**687**	chr1	53327841-53327842	A	C	CTG->CGG	**LRP8**	Leu24Arg		0,000	23	9	39,1	19	0	0,0	0,013	9
**687**	chr1	16565781-16565782	G	A	GCA->GTA	**NBPF1**	Ala972Val	rs773153392	0,119	59	28	47,5	43	0	0,0	0,000	51
**687**	chr3	195673969-195673970	C	A	CGC->CGA; GCA->GAA	**SDHAP2**	Arg193Arg; Ala46Glu	rs7615369	0,121	94	24	25,5	281	21	7,5	0,000	0
**687**	chr5	177672138-177672139	T	A	TAC->TTC	**SIMC1**	Tyr4Phe	rs769410651	0,451	60	16	26,7	73	6	8,2	0,000	0
**3953**	chr10	126101218-126101219	C	T	CGG->CAG	**ADAM12**	Arg258Gln	rs776871759	0,000	66	24	36,4	215	0	0,0	0,000	36
**3953**	chr20	28588756-28588757	T	G	AGA->AGC	**AK057099**	Arg43Ser		0,000	72	23	31,9	77	5	6,5	0,000	0
**3953**	chr11	6395681-6395682	A	G	TGG->CGG; CTG->CCG	**APBB1**	Trp100Arg; Leu425Pro; Leu440Pro;		0,000	114	38	33,3	216	0	0,0	0,000	13
**3953**	chr2	130141239-130141240	G	A	CCC->TCC	**CCDC74B**	Pro303Ser; Pro201Ser; Pro135Ser	rs142452531	0,008	29	12	41,4	106	8	7,5	0,000	0
**3953**	chr13	37104566-37104567	T	+T		**CSNK1A1L**	Lys230	rs769712615	1,000	178	52	29,2	227	0	0,0	0,000	0
**3953**	chr1	78891644-78891645	G	A	ACC->ATC	**ELTD1**	Thr630Ile	rs774129602	0,000	123	32	26,0	220	1	0,5	0,000	38

**3953**	chr4	125449890-125449891	G	A	GGT->AGT	**FAT4**	Gly2959Ser; Gly1257Ser; Gly437Ser		0,000	173	45	26,0	255	1	0,4	0,000	61
**3953**	chr1	47438934-47438935	G	A	GGC->GAC	**FOXD2**	Gly267Asp		0,000	28	8	28,6	38	0	0,0	0,014	44
**3953**	chr5	53483622-53483623	G	A	GGG->AGG	**FST**	Gly133Arg		0,000	73	25	34,3	205	0	0,0	0,000	4
**3953**	chr5	90783289-90783290	T	G	AGT->AGG	**GPR98**	Ser2172Arg; Ser4466Arg; Ser127Arg		0,000	92	25	27,2	172	0	0,0	0,000	7
**3953**	chr12	52573521-52573522	C	T	GCC->ACC	**KRT74**	Ala86Thr		0,000	113	34	30,1	264	0	0,0	0,000	14
**3953**	chr6	69676508-69676509	G	A	CTT->TTT	**LMBRD1**	Leu411Phe; Leu484Phe		0,000	118	33	28,0	175	2	1,1	0,000	3
**3953**	chr11	30411481-30411482	G	T	CCA->ACA	**MPPED2**	Pro291Thr; Pro165Thr		0,000	110	30	27,3	199	0	0,0	0,000	39
**3953**	chr1	16565781-16565782	G	A	GCA->GTA	**NBPF1**	Ala972Val	rs773153392	0,119	50	13	26,0	116	0	0,0	0,000	51
**3953**	chr18	54282979-54282980	G	A	GAT->AAT	**POLI**	Asp314Asn		0,000	122	43	35,3	124	0	0,0	0,000	15
**3953**	chr19	8499464-8499465	G	A	CTC->TTC	**PRAM1**	Leu115Phe		0,000	35	14	40,0	45	0	0,0	0,000	45
**3953**	chr11	45226413-45226414	T	G	TAC->GAC	**PRDM11**	Tyr597Asp		0,000	86	22	25,6	302	0	0,0	0,000	21
**3953**	chr22	39317458-39317459	A	G	TAA->CAA	**RPL3**	*123Gln		0,000	22	6	27,3	68	0	0,0	0,006	20
**3953**	chr16	15084641-15084642	G	A	TCG->TTG	**RRN3**	Ser100Leu; Ser199Leu; Ser169Leu	rs76732059	0,304	59	15	25,4	100	7	7,0	0,000	37

**3953**	chr22	43231763-43231764	C	G	CGC->CCC	**SCUBE1**	Arg319Pro		0,000	160	48	30,0	158	0	0,0	0,000	14
**3953**	chr12	120303849-120303850	C	T	CGG->TGG	**SIRT4**	Arg97Trp	rs201166468	0,000	62	20	32,3	332	0	0,0	0,000	28
**3953**	chr1	117981307-117981308	G	T	GCT->GAT	**SPAG17**	Ala1989Asp		0,000	116	29	25,0	224	0	0,0	0,000	13
**3953**	chr9	14925-14926	C	T	GGA->AGA	**WASH1**	Gly427Arg	rs76748727	0,012	31	9	29,0	106	8	7,5	0,000	0
**3953**	chr1	15942165-15942166	G	A	CAC->TAC	**ZBTB17**	His657Tyr; His746Tyr; His739Tyr;		0,000	210	62	29,5	383	0	0,0	0,000	55
**3953**	chr19	36514847-36514848	T	-G^TATGATTTTTC		**ZNF260**	Thr131		0,000	107	53	49,5	111	0	0,0	0,000	0
**E2013**	chr10	59247617-59247618	A	C	ATT->AGT	**FAM13C**	Ile501Ser; Ile585Ser; Ile487Ser;		0,000	89	23	25,8	245	0	0,0	0,000	0
**E2013**	chr4	78445535-78445536	C	T	CCT->TCT	**FRAS1**	Pro1894Ser; Pro314Ser		0,000	56	19	33,9	270	0	0,0	0,000	15
**E2013**	chr12	31791854-31791855	C	G	TTG->TTC	**H3F3C**	Leu104Phe	rs765335762	0,000	101	30	29,7	443	0	0,0	0,000	0
**E2013**	chr7	75556167-75556168	G	A	TCA->TTA	**HIP1**	Ser562Leu		0,000	39	12	30,8	211	0	0,0	0,000	24
**E2013**	chr10	63206599-63206600	C	T	AGT->AAT	**JMJD1C**	Ser1690Asn	rs763499705	0,000	88	36	40,9	148	0	0,0	0,000	33
**E2013**	chr5	155016221-155016222	G	A	CGG->CAG	**KIF4B**	Arg788Gln	rs370994878	0,000	53	14	26,4	157	0	0,0	0,000	46
**E2013**	chr3	46459777-46459778	C	T	GCC->ACC	**LTF**	Ala29Thr; Ala16Thr	rs1126477	0,339	42	11	26,2	63	0	0,0	0,003	12

**E2013**	chr12	40505148-40505149	G	A	GGA->AGA	**MUC19**	Gly6267Arg	rs76468484	0,001	45	15	33,3	158	10	6,3	0,000	15
**E2013**	chr1	146984875-146984876	T	G	TGT->TGG	**NBPF12**	Cys910Trp		0,000	93	24	25,8	225	15	6,7	0,000	0
**E2013**	chr1	248406858-248406859	G	T	GCC->TCC	**OR2T1**	Ala289Ser		0,000	47	19	40,4	246	0	0,0	0,000	0
**E2013**	chr16	70120576-70120577	A	G	ACG->GCG	**PDPR**	Thr29Ala	rs200469748	0,043	29	9	31,0	231	10	4,3	0,000	12
**E2013**	chr10	93959678-93959679	A	G	ATG->ACG	**PIPSL**	Met573Thr		0,000	68	19	27,9	274	0	0,0	0,000	0
**E2013**	chr10	96750492-96750493	T	G	GTC->GGC	**RPL13A**	Val69Gly	rs772750803	0,000	63	18	28,6	143	4	2,8	0,000	17
**E2013**	chr6	168653222-168653223	G	A	CGC->CAC	**SMOC2**	Arg427His; Arg438His; Arg104His	rs529459047	0,000	36	12	33,3	161	0	0,0	0,000	25
**E2013**	chr15	71728662-71728663	C	T	TCG->TTG	**THSD4**	Ser491Leu; Ser165Leu; Ser131Leu	rs754247111	0,000	49	17	34,7	132	0	0,0	0,000	24
**E2013**	chr3	75738888-75738889	G	A	GCC->GTC	**ZNF717**	Ala245Val	rs111880168	0,500	69	20	29,0	213	19	8,9	0,000	37
**E2013**	chr19	9693737-9693738	T	C	TAC->TGC	**ZNF812**	Tyr66Cys		0,000	54	21	38,9	332	0	0,0	0,000	0
**10752**	chr5	80559459-80559460	G	A	TCA->TTA	**ANKRD34B**	Ser187Leu		0,000	120	34	28,3	102	2	2,0	0,000	0
**10752**	chr16	21250536-21250537	G	A	GGC->GAC	**ANKS4B**	Gly324Asp		0,000	196	63	32,1	144	2	1,4	0,000	12
**10752**	chr20	51671174-51671175	G	A	CGC->TGC	**ATP9A**	Arg374Cys; Arg238Cys	rs756587295	0,000	144	52	36,1	192	2	1,0	0,000	10

**10752**	chr22	16591334-16591335	G	A	CCC->TCC	**CCT8L2**	Pro406Ser		0,000	182	72	39,6	291	1	0,3	0,000	0
**10752**	chr19	44703408-44703409	A	C	GAA->GCA	**CEACAM16**	Glu33Ala		0,000	299	109	36,5	131	1	0,8	0,000	17
**10752**	chr5	98872528-98872529	G	A	CCA->TCA	**CHD1**	Pro1200Ser		0,000	127	50	39,4	86	0	0,0	-2,000	24
**10752**	chr1	111320418-111320419	G	A	GCC->ACC	**CHIA**	Ala301Thr; Ala462Thr; Ala354Thr		0,000	173	68	39,3	185	2	1,1	0,000	19
**10752**	chr14	53058493-53058494	G	A	CAT->TAT	**DDHD1**	His55Tyr; His241Tyr; His659Tyr;		0,000	71	25	35,2	56	2	3,6	0,000	12
**10752**	chr17	78429255-78429256	A	C	TAT->TAG	**DNAH17**	Tyr375*; Tyr4090*		0,000	172	61	35,5	134	3	2,2	0,000	0
**10752**	chr8	143865269-143865270	C	T	GTC->ATC	**EPPK1**	Val2662Ile		0,000	39	11	28,2	21	0	0,0	0,042	30
**10752**	chr1	166070582-166070583	C	T	TGG->TGA	**FAM78B**	Trp148*		0,000	197	76	38,6	251	3	1,2	0,000	0
**10752**	chr4	186628626-186628627	T	A	AAC->ATC	**FAT1**	Asn1487Ile		0,000	177	63	35,6	99	0	0,0	0,000	39
**10752**	chr19	7870998-7870999	A	G	TCT->CCT	**FLJ22184**	Ser749Pro	rs12974877	0,250	76	19	25,0	41	2	4,9	0,000	0
**10752**	chr13	49175461-49175462	C	T	TCC->TTC	**FNDC3A**	Ser484Phe; Ser410Phe; Ser428Phe		0,000	125	56	44,8	92	0	0,0	0,000	28
**10752**	chr12	131084580-131084581	C	T	ACG->ATG	**GPR133**	Thr530Met; Thr562Met; Thr49Met	rs544054620	0,000	489	149	30,5	253	3	1,2	0,000	5
**10752**	chr2	196319802-196319803	C	T	GAC->AAC	**HECW2**	Asp7Asn; Asp363Asn	rs761027745	0,000	181	81	44,8	222	7	3,2	0,000	37

**10752**	chrX	105267472-105267473	G	A	GGA->GAA	**IL1RAPL2**	Gly210Glu		0,000	50	31	62,0	42	1	2,4	0,000	6
**10752**	chr4	89935786-89935787	A	-T^AG		**MMRN1**	Arg703; Arg445		0,000	224	75	33,5	139	1	0,7	0,000	20
**10752**	chr2	233819379-233819380	A	C	ACT->CCT	**MROH2A**	Thr1093Pro		0,000	211	80	37,9	112	1	0,9	0,000	0
**10752**	chr11	1103986-1103987	C	T	CTC->TTC	**MUC2**	Leu3140Phe		0,000	393	162	41,2	170	1	0,6	0,000	60
**10752**	chr17	10395231-10395232	C	T	ATG->ATA	**MYH8**	Met1621Ile		0,000	73	33	45,2	65	1	1,5	0,000	21
**10752**	chr1	16565781-16565782	G	A	GCA->GTA	**NBPF1**	Ala972Val	rs773153392	0,119	65	28	43,1	18	0	0,0	0,004	51
**10752**	chr1	161984095-161984096	G	A	CCC->CTC	**OLFML2B**	Pro94Leu; Pro611Leu; Pro612Leu		0,000	619	183	29,6	299	0	0,0	0,000	0
**10752**	chr7	95250103-95250104	G	A	GAA->AAA	**PPP1R9A**	Glu749Lys; Glu727Lys		0,000	104	37	35,6	64	1	1,6	0,000	28
**10752**	chr6	13696812-13696813	C	G	GTA->CTA	**RANBP9**	Val219Leu		0,000	117	51	43,6	73	0	0,0	-2,000	14
**10752**	chr8	92011072-92011073	C	T	GAA->AAA	**RUNX1T1**	Glu163Lys		0,000	229	89	38,9	187	3	1,6	0,000	83
**10752**	chr4	53145559-53145560	G	A	TCC->TTC	**SCFD2**	Ser445Phe		0,000	129	43	33,3	101	0	0,0	0,000	25
**10752**	chr9	84280009-84280010	C	T	GGG->GAG	**SLC28A3**	Gly529Glu; Gly598Glu		0,000	119	40	33,6	135	1	0,7	0,000	41
**10752**	chr16	22134367-22134368	G	A	GGC->GAC	**VWA3A**	Gly698Asp; Gly690Asp		0,000	107	50	46,7	181	3	1,7	0,000	0

**10752**	chr19	44388030-44388031	G	A	CAT->TAT	**ZNF285**	His79Tyr; His72Tyr		0,000	97	28	28,9	100	2	2,0	0,000	0
**9667**	chr5	179644956-179644957	C	G	GAC->CAC	**C5orf60**	Asp22His	rs7731123	0,251	68	19	27,9	250	12	4,8	0,000	0
**I2010**	chr1	16565781-16565782	G	A	GCA->GTA	**NBPF1**	Ala972Val	rs773153392	0,119	51	26	51,0	25	0	0,0	0,000	63
**I2010**	chrX	7843768-7843769	G	C	AGT->ACT	**VCX**	Ser125Thr	rs149395826	0,001	27	9	33,3	19	0	0,0	0,031	11
**K18**	chr15	88868311-88868312	G	A	GGG->GAG	**ACAN**	Gly2348Glu		0,000	98	25	25,5	117	0	0,0	0,000	10
**K18**	chr15	79293873-79293874	G	A	GGC->GAC	**ANKRD34C**	Gly197Asp		0,000	77	31	40,3	178	0	0,0	0,000	0
**K18**	chr18	12125747-12125748	A	T	AAA->TAA	**ANKRD62**	Lys643*		0,000	62	26	41,9	93	0	0,0	0,000	0
**K18**	chr19	1469937-1469938	G	A	GAG->AAG	**APC2**	Glu2213Lys		0,000	40	17	42,5	49	0	0,0	0,000	59
**K18**	chr2	231345070-231345071	C	T	CAC->TAC	**ARMC9**	His659Tyr		0,000	90	32	35,6	123	0	0,0	0,000	0
**K18**	chr1	23441716-23441717	C	T	GGC->AGC	**ASAP3**	Gly229Ser;<br>Gly220Ser		0,000	69	31	44,9	156	0	0,0	0,000	42
**K18**	chr7	70790336-70790337	G	A	GAC->AAC	**AUTS2**	Asp1040Asn;<br>Asp1017Asn;<br>Asp1041Asn;		0,000	208	73	35,1	504	0	0,0	0,000	19
**K18**	chr3	161086048-161086049	G	A	CCA->TCA	**B3GALNT1**	Pro236Ser		0,000	77	28	36,4	92	0	0,0	0,000	17
**K18**	chr1	92139359-92139360	G	T	GTA->TTA	**BTBD8**	Val255Leu		0,000	51	24	47,1	70	0	0,0	0,000	0

**K18**	chr22	37182677-37182678	G	A	CCC->CTC	**C1QTNF6**	Pro116Leu		0,000	216	106	49,1	290	0	0,0	0,000	42
**K18**	chr12	1878335-1878336	G	A	TCC->TCT	**CACNA2D4**	Ser502Ser; Ser566Ser; Ser451Ser;		0,000	107	42	39,3	207	0	0,0	0,000	4
**K18**	chr19	8305131-8305132	G	A	CCC->CTC	**CD320**	Pro56Leu		0,000	122	53	43,4	108	0	0,0	0,000	26
**K18**	chr22	37568406-37568407	C	A	CCT->ACT	**CDC42EP1**	Pro255Thr	rs77417880	0,067	28	7	25,0	20	0	0,0	0,116	19
**K18**	chr1	159873127-159873128	C	T	GAG->AAG	**CFAP45**	Glu465Lys; Glu380Lys		0,000	74	29	39,2	96	0	0,0	0,000	0
**K18**	chr3	74424847-74424848	C	A	GGA->TGA	**CNTN3**	Gly151*		0,000	77	33	42,9	66	0	0,0	0,000	21
**K18**	chr1	86125892-86125893	C	T	GGA->GAA	**COL24A1**	Gly148Glu		0,000	56	24	42,9	85	0	0,0	0,000	23
**K18**	chr2	227008272-227008273	G	T	AGC->AGA	**COL4A4**	Ser1518Arg		0,000	133	41	30,8	220	0	0,0	0,000	6
**K18**	chr8	3307755-3307756	G	A	CCG->TCG	**CSMD1**	Pro1298Ser; Pro1297Ser		0,000	70	26	37,1	142	0	0,0	0,000	35
**K18**	chr15	45139563-45139564	G	A	GAC->AAC	**DUOX1**	Asp452Asn; Asp98Asn		0,000	81	32	39,5	90	0	0,0	-2,000	21
**K18**	chr15	45099841-45099842	C	T	GGC->AGC	**DUOX2**	Gly1079Ser		0,000	150	59	39,3	298	0	0,0	0,000	20
**K18**	chr6	73519452-73519453	C	T	GGT->AGT	**EEF1A1**	Gly70Ser		0,000	102	48	47,1	92	0	0,0	0,000	40
**K18**	chr17	7575153-7575154	G	A	GGG->AGG	**EIF4A1**	Gly45Arg; Gly81Arg; Gly79Arg;		0,000	46	19	41,3	70	0	0,0	0,000	51

**K18**	chr22	32493254-32493255	G	T	AGG->ATG	**FBXO7**	Arg373Met; Arg294Met; Arg259Met	rs751440649	0,000	152	54	35,5	113	0	0,0	0,000	17
**K18**	chr12	49653248-49653249	C	A	GAG->TAG	**FMNL3**	Glu434*; Glu383*		0,000	136	58	42,7	163	0	0,0	0,000	36
**K18**	chr11	72221645-72221646	G	A	GAA->AAA	**FOLR2**	Glu214Lys; Glu218Lys;		0,000	134	58	43,3	167	0	0,0	0,000	33
**K18**	chr7	151148890-151148891	G	A	CGC->TGC	**GBX1**	Arg264Cys		0,000	80	28	35,0	136	0	0,0	0,000	15
**K18**	chr2	96023180-96023181	G	A	CGC->TGC	**GPAT2**	Arg692Cys; Arg621Cys	rs201647131	0,053	81	21	25,9	94	4	4,3	0,000	17
**K18**	chr11	5226662-5226663	C	T	GCT->ACT	**HBB**	Ala77Thr		0,000	59	15	25,4	200	0	0,0	0,000	11
**K18**	chr1	39626586-39626587	G	A	CCA->TCA	**HEYL**	Pro303Ser		0,000	102	40	39,2	163	0	0,0	0,000	13
**K18**	chr6	32814423-32814424	G	A	ACC->ATC	**HLA-DOB**	Thr180Ile		0,000	72	24	33,3	105	0	0,0	0,000	21
**K18**	chr3	184058504-184058505	G	A	GGG->GAG	**HTR3C**	Gly213Glu	rs141394478	0,000	60	26	43,3	110	0	0,0	0,000	20
**K18**	chr9	24543316-24543317	G	A	TCG->TTG	**IZUMO3**	Ser205Leu; Ser211Leu		0,000	46	18	39,1	37	0	0,0	0,000	0
**K18**	chr8	132129550-132129551	C	T	CGG->CAG	**KCNQ3**	Arg668Gln; Arg657Gln; Arg765Gln;	rs201328910	0,000	241	103	42,7	399	0	0,0	0,000	4
**K18**	chr12	10451073-10451074	T	G	AAA->ACA	**KLRC1**	Lys28Thr		0,000	192	91	47,4	151	0	0,0	0,000	35
**K18**	chr22	21049070-21049071	C	T	GCG->GTG	**LRRC74B**	Ala179Val	rs201821840	0,053	89	23	25,8	128	11	8,6	0,000	0

**K18**	chr10	73426296-73426297	C	T	GGG->AGG	**MSS51**	Gly195Arg	rs771172523	0,000	65	17	26,2	122	0	0,0	0,000	2
**K18**	chr2	190060323-190060324	G	A	CCC->CTC	**MSTN**	Pro162Leu		0,000	36	9	25,0	61	0	0,0	0,008	8
**K18**	chr17	8490509-8490510	C	T	GTG->ATG	**MYH10**	Val1572Met; Val1550Met; Val1541Met	rs200997387	0,000	101	29	28,7	202	0	0,0	0,000	20
**K18**	chr3	108398736-108398737	T	A	GAG->GTG	**MYH15**	Glu1698Val		0,000	92	38	41,3	122	0	0,0	0,000	11
**K18**	chr3	175755415-175755416	T	A	TAT->TAA	**NAALADL2**	Tyr729*		0,000	78	38	48,7	133	0	0,0	0,000	26
**K18**	chr2	15276927-15276928	C	A	TGT->TTT	**NBAS**	Cys1771Phe	rs750497674	0,000	87	41	47,1	90	0	0,0	-2,000	12
**K18**	chr13	35196095-35196096	A	T	GAA->GAT	**NBEA**	Glu1720Asp; Glu1721Asp; Glu1717Asp		0,000	52	27	51,9	69	0	0,0	0,000	22
**K18**	chr14	75105972-75105973	C	T	GAG->AAG	**NEK9**	Glu518Lys		0,000	49	13	26,5	68	0	0,0	0,000	37
**K18**	chr1	115838107-115838108	G	A	CGC->TGC	**NHLH2**	Arg89Cys		0,000	165	47	28,5	116	0	0,0	0,000	24
**K18**	chr17	5532974-5532975	G	A	TCC->TTC	**NLRP1**	Ser1052Phe; Ser1048Phe; Ser1018Phe		0,000	87	40	46,0	174	0	0,0	0,000	21
**K18**	chr10	82358762-82358763	G	A	CGA->CAA	**NRG3**	Arg283Gln; Arg62Gln; Arg87Gln;	rs749884304	0,000	87	37	42,5	142	0	0,0	0,000	26
**K18**	chr12	5494722-5494723	G	A	GGA->GAA	**NTF3**	Gly170Glu; Gly183Glu		0,000	157	59	37,6	240	0	0,0	0,000	10
**K18**	chr1	248488657-248488658	C	T	CGA->TGA	**OR2T5**	Arg24*	rs76332972	0,115	53	14	26,4	127	8	6,3	0,000	0

**K18**	chr1	248488721-248488722	G	A	GGA->GAA	**OR2T5**	Gly45Glu	rs139516854	0,262	49	14	28,6	122	8	6,6	0,000	0
**K18**	chr14	22770886-22770887	C	T	CCC->CTC	**OXA1L**	Pro290Leu; Pro306Leu; Pro366Leu		0,000	70	27	38,6	126	0	0,0	0,000	7
**K18**	chr11	74267181-74267182	C	T	GGG->GAG	**P4HA3**	Gly565Glu		0,000	83	28	33,7	138	0	0,0	0,000	32
**K18**	chr1	17276778-17276779	C	A	TTC->TTA	**PADI3**	Phe486Leu		0,000	145	48	33,1	238	0	0,0	0,000	8
**K18**	chr3	130733898-130733899	C	T	GAA->AAA	**PIK3R4**	Glu367Lys	rs139640851	0,000	147	62	42,2	181	0	0,0	0,000	47
**K18**	chr15	42153317-42153318	G	A	ATC->ATT	**PLA2G4F**	Ile172Ile; Ser109Leu		0,000	70	21	30,0	147	0	0,0	0,000	0
**K18**	chr2	43710071-43710072	G	T	CAG->CAT	**PLEKHH2**	Gln683His		0,000	31	9	29,0	42	0	0,0	0,007	0
**K18**	chr19	55239486-55239487	G	C	CAG->GAG	**PPP6R1**	Gln557Glu		0,000	117	41	35,0	224	0	0,0	0,000	0
**K18**	chr19	53906371-53906372	C	T	ACC->ATC	**PRKCG**	Thr607Ile		0,000	101	44	43,6	177	0	0,0	0,000	14
**K18**	chr12	130450215-130450216	A	G	TGT->CGT	**RIMBP2**	Cys196Arg; Cys172Arg; Cys189Arg		0,000	51	24	47,1	118	0	0,0	0,000	0
**K18**	chr6	37376997-37376998	C	G	CTC->GTC	**RNF8**	Leu401Val		0,000	37	19	51,4	39	0	0,0	0,000	66
**K18**	chrX	154400836-154400837	C	T	CGG->TGG	**RPL10**	Arg159Trp; Arg210Trp; Gly155Gly;	rs979369776	0,000	63	17	27,0	52	2	3,8	0,000	28
**K18**	chr1	15661233-15661234	G	A	GAT->AAT	**RSC1A1**	Asp456Asn	rs759773926	0,000	79	31	39,2	90	0	0,0	0,000	10

**K18**	chr1	237784007-237784008	C	T	GCC->GTC	**RYR2**	Ala4099Val		0,000	196	85	43,4	219	0	0,0	0,000	3
**K18**	chr15	33818680-33818681	G	A	AGG->AAG	**RYR3**	Arg3568Lys; Arg3563Lys; Arg3567Lys;		0,000	37	15	40,5	45	0	0,0	0,000	3
**K18**	chr15	90225097-90225098	C	T	CCC->CTC	**SEMA4B**	Pro442Leu		0,000	135	51	37,8	126	0	0,0	0,000	48
**K18**	chr20	45208423-45208424	G	A	AGC->AAC	**SEMG1**	Ser376Asn	rs970575286	0,000	22	9	40,9	53	0	0,0	0,000	15
**K18**	chr14	36734541-36734542	G	A	CCT->TCT	**SLC25A21**	Pro79Ser	rs772207743	0,000	62	35	56,5	72	0	0,0	0,000	20
**K18**	chr19	6430154-6430155	A	C	TCC->GCC	**SLC25A41**	Ser124Ala; Thr156Thr		0,000	35	13	37,1	80	0	0,0	0,000	0
**K18**	chr1	149913637-149913638	C	T	GGA->GAA	**SV2A**	Gly68Glu		0,000	92	46	50,0	165	0	0,0	0,000	6
**K18**	chr1	43419899-43419900	C	T	TCC->TTC	**SZT2**	Ser349Phe		0,000	76	39	51,3	139	0	0,0	0,000	16
**K18**	chr5	9629623-9629624	C	T	GTA->ATA	**TAS2R1**	Val137Ile; Val97Ile		0,000	71	23	32,4	120	0	0,0	0,000	3
**K18**	chr2	101059524-101059525	C	T	GAA->AAA	**TBC1D8**	Glu85Lys; Glu100Lys		0,000	57	30	52,6	61	0	0,0	0,000	0
**K18**	chr16	1975078-1975079	G	A	GAC->AAC	**TBL3**	Asp92Asn; Asp206Asn	rs147896871	0,001	96	38	39,6	177	1	0,6	0,000	15
**K18**	chr11	78672103-78672104	C	T	GAC->AAC	**TENM4**	Asp1908Asn	rs189247153	0,000	87	39	44,8	180	0	0,0	0,000	11
**K18**	chr1	45345132-45345133	C	T	GAA->AAA	**TESK2**	Glu446Lys; Glu475Lys		0,000	159	77	48,4	424	0	0,0	0,000	26

**K18**	chr9	117714594-117714595	C	T	CCA->TCA	**TLR4**	Pro783Ser; Pro823Ser		0,000	56	20	35,7	126	0	0,0	0,000	31
**K18**	chr1	155688372-155688373	G	A	TCC->TTC	**YY1AP1**	Ser31Phe	rs769104797	0,000	153	70	45,8	138	0	0,0	0,000	71
**K18**	chrX	71241016-71241017	G	A	CCC->TCC	**ZMYM3**	Pro1248Ser; Pro1338Ser; Pro1340Ser;		0,000	94	75	79,8	165	0	0,0	0,000	41
**K18**	chr20	45952474-45952475	G	A	GCC->GTC	**ZNF335**	Ala954Val		0,000	104	54	51,9	138	0	0,0	0,000	15
**K18**	chr20	59193441-59193442	G	A	TGG->TAG	**ZNF831**	Trp808*		0,000	119	51	42,9	390	0	0,0	0,000	0
**K18**	chr19	58368949-58368950	G	A	TCC->TTC	**ZNF837**	Ser128Phe		0,000	79	32	40,5	152	0	0,0	0,000	0
**GPS 56-57**	chr11	17412703-17412704	C	-G^C		**ABCC8**	Ala840; Ala841; Ala830		0,000	35	9	25,7	33	0	0,0	0,046	6
**GPS 56-57**	chr19	44066207-44066208	A	C	CAG->CCG	**AC084219.2**	Gln237Pro		0,000	38	14	36,8	35	0	0,0	0,002	0
**GPS 56-57**	chr1	78920339-78920340	A	T	ATA->AAA	**ADGRL4**	Ile435Lys		0,000	43	15	34,9	49	0	0,0	0,000	0
**GPS 56-57**	chr1	48634575-48634576	G	A	CGT->TGT	**AGBL4**	Arg290Cys	rs748307842	0,000	26	7	26,9	27	0	0,0	0,024	0
**GPS 56-57**	chr6	24533637-24533638	C	T	CTT->TTT	**ALDH5A1**	Leu512Phe; Leu484Phe; Leu525Phe		0,000	26	11	42,3	43	1	2,3	0,000	7
**GPS 56-57**	chr2	73451600-73451601	C	T	CCA->TCA	**ALMS1**	Pro1692Ser; Pro1650Ser	rs764039432	0,000	138	44	31,9	133	1	0,8	0,000	7
**GPS 56-57**	chr12	5612745-5612746	C	T	GGG->GAG	**ANO2**	Gly671Glu; Gly667Glu		0,000	39	14	35,9	46	0	0,0	0,000	14

**GPS 56-57**	chr16	88810138-88810139	C	A	GAG->TAG	**APRT**	Glu111*; Glu84*		0,000	30	13	43,3	43	1	2,3	0,000	24
**GPS 56-57**	chr3	154122601-154122602	C	T	CCT->TCT	**ARHGEF26**	Pro204Ser		0,000	39	18	46,2	35	0	0,0	0,000	0
**GPS 56-57**	chr15	72544838-72544839	G	A	GAG->AAG	**ARIH1**	Glu155Lys		0,000	29	12	41,4	35	0	0,0	0,001	25
**GPS 56-57**	chr13	51975131-51975132	C	T	GAA->AAA	**ATP7B**	Glu30Lys		0,000	53	17	32,1	60	0	0,0	0,000	17
**GPS 56-57**	chr11	102350556-102350557	G	A	GAT->AAT	**BIRC2**	Asp77Asn; Asp186Asn; Asp235Asn;		0,000	80	35	43,8	63	1	1,6	0,000	69
**GPS 56-57**	chrX	50309250-50309251	C	T	TCC->TTC	**CCNB3**	Ser361Phe		0,000	40	10	25,0	62	1	1,6	0,000	59
**GPS 56-57**	chr19	51483215-51483216	G	T	ATG->ATT	**CEACAM18**	Met291Ile		0,000	34	12	35,3	65	1	1,5	0,000	0
**GPS 56-57**	chr3	150972622-150972623	C	T	GGG->GAG	**CLRN1**	Gly29Glu		0,000	115	42	36,5	111	2	1,8	0,000	1
**GPS 56-57**	chr12	22058592-22058593	T	G	TTA->GTA	**CMAS**	Leu196Val; Leu37Val		0,000	61	21	34,4	90	1	1,1	0,000	13
**GPS 56-57**	chr6	70156336-70156337	C	T	CCT->TCT	**COL19A1**	Pro736Ser	rs149314833	0,000	28	8	28,6	46	0	0,0	0,005	4
**GPS 56-57**	chr12	38724876-38724877	C	T	GGA->GAA	**CPNE8**	Gly274Glu; Gly262Glu		0,000	23	8	34,8	44	0	0,0	0,002	26
**GPS 56-57**	chr10	123757313-123757314	C	T	GAA->AAA	**CPXM2**	Glu606Lys; Glu102Lys	rs747279699	0,000	53	23	43,4	63	0	0,0	0,000	11
**GPS 56-57**	chr7	117734971-117734972	C	T	GAT->AAT	**CTTNBP2**	Asp1273Asn	rs754336610	0,000	22	6	27,3	26	0	0,0	0,038	16

**GPS 56-57**	chr11	6623220-6623221	G	A	CAC->TAC	**DCHS1**	His2819Tyr		0,000	24	8	33,3	24	0	0,0	0,023	26
**GPS 56-57**	chr7	21681650-21681651	G	A	GTG->ATG	**DNAH11**	Val1817Met; Val1812Met	rs376798792	0,000	37	14	37,8	38	1	2,6	0,000	1
**GPS 56-57**	chr1	225097120-225097121	C	T	CTT->TTT	**DNAH14**	Leu1193Phe		0,000	37	15	40,5	38	0	0,0	0,000	0
**GPS 56-57**	chr5	147395684-147395685	C	T	GAT->AAT	**DPYSL3**	Asp500Asn; Asp614Asn	rs772681423	0,000	30	8	26,7	25	0	0,0	0,031	38
**GPS 56-57**	chr4	36294860-36294861	C	T	CAA->TAA	**DTHD1**	Gln199*; Gln404*; Gln364*		0,000	88	36	40,9	82	0	0,0	0,000	0
**GPS 56-57**	chr6	93246828-93246829	G	C	CCA->GCA	**EPHA7**	Pro897Ala		0,000	29	13	44,8	45	2	4,4	0,000	33
**GPS 56-57**	chr9	136717627-136717628	G	A	GTC->ATC	**FAM69B**	Val39Ile	rs752961190	0,000	21	10	47,6	15	0	0,0	0,011	0
**GPS 56-57**	chr19	8141773-8141774	A	T	TTC->ATC	**FBN3**	Phe270Ile		0,000	26	12	46,2	34	1	2,9	0,000	36
**GPS 56-57**	chr5	177090764-177090765	G	A	GAT->AAT	**FGFR4**	Asp126Asn	rs370322847	0,000	70	22	31,4	82	2	2,4	0,000	49
**GPS 56-57**	chr4	143697983-143697984	C	T	GGG->AGG	**FREM3**	Gly898Arg	rs878960888	0,000	42	17	40,5	51	1	2,0	0,000	0
**GPS 56-57**	chr6	89178780-89178781	T	G	ATT->CTT	**GABRR1**	Ile477Leu; Ile460Leu; Ile390Leu		0,000	43	15	34,9	38	0	0,0	0,001	9
**GPS 56-57**	chr6	55331731-55331732	G	A	GAA->AAA	**GFRAL**	Glu14Lys		0,000	40	20	50,0	38	0	0,0	0,000	39
**GPS 56-57**	chr3	190857874-190857875	C	T	GAA->AAA	**GMNC**	Glu98Lys		0,000	42	13	31,0	48	0	0,0	0,001	14

**GPS 56-57**	chr21	29689812-29689813	A	C	GAT->GAG	**GRIK1**	Asp153Glu		0,000	23	11	47,8	46	0	0,0	0,000	8
**GPS 56-57**	chr4	155713463-155713464	T	C	TCA->CCA	**GUCY1A3**	Ser485Pro; Ser227Pro		0,000	87	32	36,8	111	1	0,9	0,000	21
**GPS 56-57**	chr1	185980998-185980999	G	A	GGA->GAA	**HMCN1**	Gly863Glu		0,000	42	12	28,6	51	0	0,0	0,004	21
**GPS 56-57**	chr10	89307162-89307163	C	T	CAG->TAG	**IFIT2**	Gln403*		0,000	41	15	36,6	22	0	0,0	0,006	35
**GPS 56-57**	chr7	86909936-86909937	C	T	GGG->GAG	**KIAA1324L**	Gly745Glu; Gly674Glu; Gly578Glu		0,000	41	15	36,6	51	0	0,0	0,000	0
**GPS 56-57**	chr12	57569303-57569304	G	A	GAC->AAC	**KIF5A**	Asp290Asn; Asp201Asn		0,000	48	15	31,3	38	0	0,0	0,003	7
**GPS 56-57**	chr4	54658056-54658057	C	G	CTC->GTC	**KIT**	Leu15Val		0,000	39	15	38,5	40	0	0,0	0,000	31
**GPS 56-57**	chr3	42688968-42688969	C	T	CAT->TAT	**KLHL40**	His508Tyr		0,000	22	8	36,4	29	0	0,0	0,003	0
**GPS 56-57**	chr12	52679861-52679862	G	T	CAG->AAG	**KRT1**	Gln163Lys		0,000	38	15	39,5	66	0	0,0	0,000	29
**GPS 56-57**	chr9	136740918-136740919	A	G	GTG->GCG	**LCN10**	Val118Ala; Val131Ala		0,000	20	12	60,0	19	0	0,0	0,000	0
**GPS 56-57**	chrX	10567282-10567283	C	T	GCA->ACA	**MID1**	Ala89Thr		0,000	63	24	38,1	77	1	1,3	0,000	12
**GPS 56-57**	chr4	3253225-3253226	G	A	GAT->AAT	**MSANTD1**	Asp101Asn; Asp114Asn		0,000	38	10	26,3	36	0	0,0	0,025	0
**GPS 56-57**	chr11	47618845-47618846	T	C	AAA->AGA	**MTCH2**	Lys300Arg	rs73465610	0,005	71	18	25,4	64	5	7,8	0,000	21

**GPS 56-57**	chr11	95838138-95838139	T	A	TTA->TTT	**MTMR2**	Leu516Phe; Leu444Phe		0,000	30	17	56,7	30	0	0,0	0,000	10
**GPS 56-57**	chr6	31029206-31029207	C	T	TCC->TTC	**MUC22**	Ser1259Phe		0,000	233	81	34,8	215	2	0,9	0,000	0
**GPS 56-57**	chr20	34754856-34754857	G	A	CGC->TGC	**NCOA6**	Arg514Cys	rs532522078	0,000	25	11	44,0	27	0	0,0	0,001	35
**GPS 56-57**	chr2	151694523-151694524	C	T	GAT->AAT	**NEB**	Asp594Asn	rs768295694	0,000	75	33	44,0	82	0	0,0	0,000	8
**GPS 56-57**	chr12	55027186-55027187	G	A	GAG->AAG	**NEUROD4**	Glu250Lys		0,000	115	37	32,2	114	1	0,9	0,000	9
**GPS 56-57**	chr19	54933664-54933665	G	T	ACA->AAA	**NLRP7**	Thr849Lys; Thr821Lys		0,000	29	9	31,0	34	0	0,0	0,017	57
**GPS 56-57**	chr1	154095002-154095003	A	C	TGG->GGG	**NUP210L**	Trp707Gly		0,000	97	33	34,0	106	0	0,0	0,000	0
**GPS 56-57**	chr1	247712436-247712437	A	C	TTA->GTA	**OR6F1**	Leu107Val		0,000	42	17	40,5	40	2	5,0	0,000	0
**GPS 56-57**	chr5	140869913-140869914	G	A	GAA->AAA	**PCDHA11**	Glu271Lys	rs782742972	0,000	56	18	32,1	66	1	1,5	0,000	0
**GPS 56-57**	chr11	14835070-14835071	G	T	GGT->TGT	**PDE3B**	Gly766Cys; Gly715Cys		0,000	23	12	52,2	21	0	0,0	0,001	15
**GPS 56-57**	chr6	51904009-51904010	C	T	GAG->AAG	**PKHD1**	Glu2281Lys		0,000	93	38	40,9	93	0	0,0	0,000	14
**GPS 56-57**	chr1	155294497-155294498	G	A	CAC->TAC	**PKLR**	His286Tyr; His317Tyr		0,000	37	18	48,7	42	1	2,4	0,000	19
**GPS 56-57**	chr1	973526-973527	C	T	CCA->TCA	**PLEKHN1**	Pro441Ser; Pro406Ser; Pro493Ser	rs376427746	0,000	34	14	41,2	46	1	2,2	0,000	0

**GPS 56-57**	chr15	74035297-74035298	C	T	CCC->TCC; CCC->TCC	**PML**	Pro613Ser; Pro565Ser; Ala626Val		0,000	30	9	30,0	32	0	0,0	0,020	59
**GPS 56-57**	chr21	45267526-45267527	G	A	CCC->CTC	**POFUT2**	Pro400Leu		0,000	43	14	32,6	65	0	0,0	0,000	17
**GPS 56-57**	chr6	149505498-149505499	C	T	AGA->AAA; GAA->AAA	**PPIL4**	Arg478Lys; Glu111Lys		0,000	75	27	36,0	74	0	0,0	0,000	0
**GPS 56-57**	chr7	122010639-122010640	G	A	GAA->AAA	**PTPRZ1**	Glu532Lys		0,000	68	24	35,3	68	0	0,0	0,000	34
**GPS 56-57**	chr18	23026213-23026214	C	T	CCA->TCA; CCA->TCA	**RBBP8**	Pro890Ser; Leu857Leu; Pro895Ser;		0,000	41	14	34,2	51	0	0,0	0,000	66
**GPS 56-57**	chr13	24793140-24793141	G	C	AAG->AAC	**RNF17**	Lys345Asn		0,000	98	29	29,6	102	2	2,0	0,000	25
**GPS 56-57**	chr1	237623824-237623825	C	T	CGT->TGT	**RYR2**	Arg1993Cys	rs767406030	0,000	63	20	31,8	61	1	1,6	0,000	3
**GPS 56-57**	chr11	22359486-22359487	G	A	GGA->GAA	**SLC17A6**	Gly178Glu		0,000	34	15	44,1	45	1	2,2	0,000	2
**GPS 56-57**	chr1	75300665-75300666	A	C	TGC->GGC	**SLC44A5**	Cys41Gly		0,000	56	20	35,7	68	0	0,0	0,000	0
**GPS 56-57**	chr3	27436532-27436533	T	-C^T		**SLC4A7**	Glu135; Glu139; Glu144; Glu148		0,000	66	27	40,9	73	3	4,1	0,000	27
**GPS 56-57**	chr8	17544561-17544562	A	T	GAA->GTA	**SLC7A2**	Glu163Val; Glu203Val		0,000	30	8	26,7	25	0	0,0	0,031	22
**GPS 56-57**	chr3	165188750-165188751	C	T	GAA->AAA	**SLITRK3**	Glu694Lys		0,000	35	14	40,0	26	0	0,0	0,001	21
**GPS 56-57**	chrX	153783239-153783240	C	T	CAG->TAG	**SRPK3**	Gln255*; Gln322*; Gln213*		0,000	30	17	56,7	32	0	0,0	0,000	0

**GPS 56-57**	chr12	94581934-94581935	C	T	GAG->AAG	**TMCC3**	Glu228Lys; Glu197Lys		0,000	71	32	45,1	70	0	0,0	0,000	0
**GPS 56-57**	chr6	32072083-32072084	G	C	ATC->ATG	**TNXB**	Ile1632Met; Ile1719Met		0,000	33	9	27,3	38	0	0,0	0,019	18
**GPS 56-57**	chr17	7675084-7675085	C	A	TGC->TTC	**TP53**	Cys176Phe; Cys137Phe; Cys44Phe;		0,000	21	9	42,9	32	0	0,0	0,004	91
**GPS 56-57**	chr14	22003251-22003252	A	G	ATA->ATG	**TRAV18**	Ile14Met		0,000	24	16	66,7	37	0	0,0	0,000	0
**GPS 56-57**	chr12	71132712-71132713	C	T	GGA->AGA	**TSPAN8**	Gly186Arg		0,000	54	19	35,2	64	0	0,0	0,000	54
**GPS 56-57**	chr2	209929932-209929933	C	T	CGA->TGA	**UNC80**	Arg1886*; Arg1891*		0,000	41	16	39,0	57	0	0,0	0,000	7
**GPS 56-57**	chrX	108077135-108077136	A	G	ATT->GTT	**VSIG1**	Ile307Val; Ile343Val	rs983567597	0,000	57	27	47,4	62	0	0,0	0,000	70
**GPS 56-57**	chr14	100354456-100354457	T	A	ATT->TTT	**WARS**	Ile137Phe; Ile178Phe		0,000	52	17	32,7	62	0	0,0	0,000	5
**GPS 56-57**	chr1	241679557-241679558	T	A	ATT->AAT	**WDR64**	Ile186Asn		0,000	40	12	30,0	51	0	0,0	0,003	0
**GPS 56-57**	chrX	153347249-153347250	C	T	CGG->TGG	**ZNF275**	Arg136Trp; Arg189Trp		0,000	45	15	33,3	46	0	0,0	0,000	0
**GPS 56-57**	chr19	53577557-53577558	G	A	GGG->GAG	**ZNF331**	Gly333Glu		0,000	45	18	40,0	47	0	0,0	0,000	63
**GPS 56-57**	chr19	57606250-57606251	T	G	AGT->AGG	**ZNF530**	Ser242Arg		0,000	63	20	31,8	63	1	1,6	0,000	0
**GPS 56-57**	chrX	85270145-85270146	G	T	GCT->TCT	**ZNF711**	Ala370Ser; Ala416Ser		0,000	26	17	65,4	38	2	5,3	0,000	28

**GPS 66-67**	--	--	--	--	--	--	--	--	--	--	--	--	--	--	--	--	--
**GPS 70-71**	--	--	--	--	--	--	--	--	--	--	--	--	--	--	--	--	--
**GPS 74-75**	chr3	75738359-75738360	C	A	AAG->AAT	**ZNF717**	Lys371Asn	rs79432557	0,500	75	20	26,7	82	8	9,8	0,000	0
**GPS 89-90**	chr7	100954960-100954961	A	C	AAC->ACC	**MUC3A**	Asn1061Thr		0,000	34	15	44,12	13	0	0	0,036	40
**GPS 89-90**	chr16	1786794-1786795	C	A	ATC->ATA; CCC->ACC	**NUBP2**	Ile58Ile; Pro65Thr; Ile146Ile	rs2235648	0,549	28	13	46,43	12	0	0	0,030	11
**GPS 25-26**	chr1	33013298-33013299	T	A	TAC->TTC	**AK2**	Tyr201Phe; Tyr159Phe	rs113711467	0,010	46	14	30,43	60	3	5	0,00000349	8
**GPS 25-26**	chr5	35957321-35957322	T	A	AAG->ATG	**UGT3A1**	Lys314Met; Lys280Met		0,000	24	8	33,33	52	0	0	0,00315901	14
**GPS 25-26**	chr5	130184583-130184584	A	G	CAG->CGG	**CHSY3**	Gln481Arg		0,000	58	24	41,38	82	0	0	0	0
**GPS 25-26**	chr5	148414182-148414183	G	A	GTA->ATA	**FBXO38**	Val381Ile		0,000	20	13	65	32	0	0	0,00000886	0
**GPS 25-26**	chr6	38842768-38842769	A	C	AAT->CAT	**DNAH8**	Asn1571His; Asn1354His		0,000	33	14	42,42	59	0	0	0,00002019	0
**GPS 25-26**	chr17	53824675-53824676	G	A	AGG->AAG	**KIF2B**	Arg548Lys		0,000	46	19	41,3	85	0	0	0	44
**GPS 25-26**	chr6	106105597-106105598	C	T	CAG->TAG	**PRDM1**	Gln444*; Gln480*; Gln443*;		0,000	103	77	74,76	114	0	0	0,00000001	41
**GPS 25-26**	chr12	62801931-62801932	C	T	GGA->AGA	**PPM1H**	Gly214Arg		0,000	22	9	40,91	24	0	0	0,00356838	36

**GPS 25-26**	chr12	113945877-113945878	C	T	GAT->AAT	**RBM19**	Asp526Asn	rs200306327	0,000	45	19	42,22	56	0	0	0,00001445	19
**GPS 25-26**	chr7	84129181-84129182	C	T	GTG->ATG	**SEMA3A**	Val92Met		0,000	47	20	42,55	82	0	0	0	20
**GPS 25-26**	chr17	76946408-76946409	G	A	GAG->AAG	**MGAT5B**	Glu626Lys; Glu628Lys; Glu637Lys	rs199775293	0,000	30	17	56,67	36	0	0	0,00000504	30
**GPS 25-26**	chr19	54818385-54818386	C	T	CGG->TGG	**KIR3DL1**	Arg48Trp	rs756691092	0,000	27	15	55,56	29	0	0	0,00001405	29
**GPS 27-28**	--	--	--	--	--	--	--	--	--	--	--	--	--	--	--	--	--
**2v3**	chr4	76369598-76369599	C	T	GAG->AAG	**CCDC158**	Glu392Lys		0,000	22	6	27,3	63	0	0,00	0,008	0
**2v3**	chr9	39078874-39078875	C	T	GGC->GAC	**CNTNAP3**	Gly1163Asp; Gly1082Asp	rs75217636	1,000	41	12	29,3	108	6	5,56	0,000	12
**2v3**	chr13	36125959-36125960	G	A	CGT->TGT	**DCLK1**	Arg60Cys		0,000	28	10	35,7	70	2	2,86	0,000	61
**2v3**	chr16	66723799-66723800	A	C	AAT->AAG	**DYNC1LI2**	Asn467Lys; Asn390Lys		0,000	31	10	32,3	47	1	2,13	0,000	39
**2v3**	chr9	34724745-34724746	C	T	GAA->AAA	**FAM205A**	Glu832Lys		0,000	38	20	52,6	100	1	1,00	0,000	0
**2v3**	chr14	99668245-99668246	G	A	GGG->GAG	**HHIPL1**	Gly558Glu		0,000	34	11	32,4	57	0	0,00	0,002	0
**2v3**	chr6	26196967-26196968	C	T	GAG->AAG	**HIST1H3D**	Glu95Lys		0,000	61	19	31,2	121	0	0,00	0,000	0
**2v3**	chr2	8778903-8778904	T	C	GAT->GGT	**KIDINS220**	Asp870Gly; Asp869Gly		0,000	38	10	26,3	93	0	0,00	0,000	31

**2v3**	chr7	152182067-152182068	G	A	TCG->TTG	**KMT2C**	Ser1931Leu	rs200297010	0,000	25	14	56,0	68	0	0,00	0,000	68
**2v3**	chr7	107986063-107986064	G	A	CCT->TCT	**LAMB1**	Pro236Ser; Pro212Ser		0,000	85	35	41,2	127	1	0,79	0,000	26
**2v3**	chr3	174279510-174279511	G	A	GCC->ACC	**NLGN1**	Ala504Thr		0,000	48	17	35,4	74	0	0,00	0,000	6
**2v3**	chr7	94911140-94911141	T	G	CTG->CGG	**PPP1R9A**	Leu343Arg		0,000	108	40	37,0	211	0	0,00	0,000	32
**2v3**	chr4	81433886-81433887	C	T	CGG->CAG	**RASGEF1B**	Arg425Gln; Arg426Gln; Arg384Gln	rs755507736	0,000	31	8	25,8	86	0	0,00	0,000	18
**2v3**	chr16	19180399-19180400	C	A	AGC->AGA	**SYT17**	Ser3Arg; Ser64Arg; Ser60Arg		0,000	22	8	36,4	34	0	0,00	0,009	6
**2v3**	chr11	79069870-79069871	G	A	TCG->TTG	**TENM4**	Ser25Leu		0,000	27	10	37,0	60	0	0,00	0,000	13
**4v5**	chr1	161548423-161548424	T	C	ATC->GTC	**FCGR3A**	Ile142Val; Ile106Val; Ile105Val	rs148181339	0,299	56	20	35,7	51	4	7,84	0,000	45
**4v5**	chrX	136251294-136251295	G	A	CGG->TGG	**MAP7D3**	Arg22Trp		0,000	37	10	27,0	40	0	0,00	0,011	0
**4v5**	chr13	25097528-25097529	A	T	AAG->ATG	**PABPC3**	Lys444Met	rs75484271	0,010	64	16	25,0	101	7	6,93	0,000	0
**4v5**	chr2	178671993-178671994	T	C	GAA->GGA	**TTN**	Glu10434Gly; Glu11735Gly; Glu11361Gly		0,000	35	9	25,7	40	0	0,00	0,019	10
**6v7**	chr9	39078874-39078875	C	T	GGC->GAC	**CNTNAP3**	Gly1163Asp; Gly1082Asp	rs75217636	1,000	44	11	25,0	98	4	4,08	0,000	11
**8v9**	chr19	8904687-8904688	T	A	ACC->TCC	**MUC16**	Thr12742Ser	rs202219452	0,000	53	14	26,4	79	7	8,86	0,000	74

**8v9**	chr2	137450919-137450920	G	C	TGC->TCC	**THSD7B**	Cys1012Ser; Cys981Ser		0,000	46	12	26,1	48	0	0,00	0,003	0
**B2**	chr15	82345128-82345129	C	T	CGT->CAT	**GOLGA6L10**	Arg244His	rs202051302	0,001	36	13	36,1	16	0	0,00	0,040	0
**B2**	chr6	31271164-31271165	G	A	GCG->GTG	**HLA-C**	Ala176Val	rs2308590	0,274	59	24	40,7	80	2	2,50	0,000	22
**B2**	chrX	141906244-141906245	G	C	GTG->CTG	**MAGEC1**	Val281Leu	rs176044	0,183	24	18	75,0	62	3	4,84	0,000	83
**B2**	chr11	47622718-47622719	C	T	TGG->TGA	**MTCH2**	Trp269*; Trp260*		na	56	17	30,4	129	6	4,65	0,000	21
**B2**	chr1	16565781-16565782	G	A	GCA->GTA	**NBPF1**	Ala972Val	rs773153392	0,119	35	12	34,3	66	0	0,00	0,000	63
**B2**	chr1	248061266-248061267	G	T	GGC->TGC	**OR2L3**	Gly196Cys	rs75239130	0,478	645	187	29,0	541	47	8,69	0,000	0
**B2**	chr2	107859471-107859472	C	T	CTT->TTT	**RGPD4**	Leu518Phe	rs143215949	0,001	260	74	28,5	377	36	9,55	0,000	0
**B2**	chr16	28592474-28592475	T	C	CAA->CGA	**SULT1A2**	Gln155Arg		0,000	204	53	26,0	517	16	3,09	0,000	34
**B3**	chr17	68924866-68924867	C	T	GAA->AAA	**ABCA8**	Glu426Lys; Glu57Lys		0,000	59	21	35,6	127	0	0,00	0,000	40
**B3**	chr1	146344576-146344577	G	C	CGT->GGT	**AC243756.1**	Arg47Gly		0,000	28	9	32,1	164	0	0,00	0,000	0
**B3**	chr2	235799373-235799374	A	C	AAT->ACT	**AGAP1**	Asn270Thr; Asn535Thr		0,000	52	13	25,0	237	1	0,42	0,000	39
**B3**	chr11	67299998-67299999	G	A	GTG->ATG	**ANKRD13D**	Val230Met; Val317Met		0,000	38	18	47,4	83	0	0,00	0,000	0

**B3**	chr2	111130160-111130161	C	G	CTG->GTG	**BCL2L11**	Leu52Val		0,000	37	10	27,0	26	0	0,00	0,020	61
**B3**	chr5	173163766-173163767	A	G	AAG->AGG	**BNIP1**	Lys144Arg; Lys221Arg; Lys187Arg;		0,000	50	15	30,0	100	0	0,00	0,000	30
**B3**	chr2	158250907-158250908	C	T	CGT->CAT	**CCDC148**	Arg381His; Arg372His	rs768078869	0,000	40	18	45,0	57	0	0,00	0,000	0
**B3**	chr5	42799718-42799719	C	T	CGC->TGC	**CCDC152**	Arg179Cys; Arg235Cys		na	24	6	25,0	46	0	0,00	0,017	0
**B3**	chr15	75689743-75689744	C	T	GAG->AAG	**CSPG4**	Glu441Lys	rs79463888	0,155	67	18	26,9	274	18	6,57	0,000	65
**B3**	chr22	45328016-45328017	G	A	CGG->CAG	**FAM118A**	Arg159Gln	rs62001863	0,006	28	13	46,4	148	8	5,41	0,000	0
**B3**	chr15	82345023-82345024	C	T	CGT->CAT	**GOLGA6L10**	Arg245His; Arg279His		0,000	43	20	46,5	23	1	4,35	0,000	0
**B3**	chr14	106874999-106875000	G	C	AGC->AGG	**IGHV7-81**	Ser6Arg		0,000	22	6	27,3	114	2	1,75	0,000	0
**B3**	chr7	100993132-100993133	G	A	CGA->CAA	**MUC12**	Arg1000Gln; Arg857Gln		0,000	25	7	28,0	75	0	0,00	0,005	43
**B3**	chr19	8917565-8917566	T	C	AAC->GAC	**MUC16**	Asn12184Asp	rs199790313	0,000	36	10	27,8	120	0	0,00	0,000	74
**B3**	chr7	100959051-100959052	G	A	GGC->AGC	**MUC3A**	Gly2425Ser	rs75842043	0,031	149	38	25,5	138	1	0,72	0,000	40
**B3**	chr11	1017954-1017955	C	T	GTT->ATT	**MUC6**	Val1616Ile	rs113926343	0,045	29	14	48,3	47	3	6,38	0,000	70
**B3**	chr17	18141102-18141103	G	A	GGA->AGA	**MYO15A**	Gly1831Arg		0,000	91	32	35,2	258	0	0,00	0,000	5

**B3**	chr1	16565697-16565698	A	T	CTG->CAG	**NBPF1**	Leu1000Gln	rs779418724	0,036	38	10	26,3	167	0	0,00	0,000	64
**B3**	chr1	144423921-144423922	T	C	AAA->GAA	**NBPF15**	Lys573Glu		0,000	52	13	25,0	32	0	0,00	0,039	0
**B3**	chr1	149080124-149080125	A	G	TTT->TCT	**NBPF9**	Phe69Ser		0,000	24	6	25,0	115	1	0,87	0,000	0
**B3**	chr16	14951776-14951777	T	C	TGT->CGT	**NPIPA1**	Cys269Arg	rs146043318	0,001	472	134	28,4	554	0	0,00	0,000	0
**B3**	chr16	29382391-29382392	G	C	CCG->CGG	**NPIPB11**	Pro847Arg		0,000	40	16	40,0	150	0	0,00	0,000	0
**B3**	chr16	28456863-28456864	A	G	TGT->CGT	**NPIPB7**	Cys269Arg		0,000	102	30	29,4	15	0	0,00	0,000	0
**B3**	chr1	248061266-248061267	G	T	GGC->TGC	**OR2L3**	Gly196Cys	rs75239130	0,478	294	81	27,6	600	49	8,17	0,000	0
**B3**	chr1	13420982-13420983	C	T	CGT->TGT	**PRAMEF20**	Arg385Cys		na	50	14	28,0	292	19	6,51	0,000	0
**B3**	chr12	11267572-11267573	C	G	GAA->CAA	**PRB3**	Glu226Gln		0,000	56	20	35,7	78	3	3,85	0,000	7
**B3**	chr4	16016217-16016218	A	T	CTT->CAT	**PROM1**	Leu342His;<br>Leu333His		0,000	50	28	56,0	86	0	0,00	0,000	64
**B3**	chr2	107859471-107859472	C	T	CTT->TTT	**RGPD4**	Leu518Phe	rs143215949	0,001	149	46	30,9	353	28	7,93	0,000	0
**B3**	chr5	236562-236563	G	A	GCA->ACA	**SDHA**	Ala418Thr;<br>Ala466Thr;<br>Ala321Thr	rs111387770	0,038	159	43	27,0	538	39	7,25	0,000	38
**B3**	chr19	51265805-51265806	G	A	GTG->ATG	**SIGLECL1**	Val112Met;<br>Val18Met	rs747602773	0,000	209	55	26,3	426	0	0,00	0,000	0

**B3**	chr2	241501514-241501515	C	G	AGC->ACC	**STK25**	Ser75Thr		0,000	75	29	38,7	381	0	0,00	0,000	27
**B3**	chr16	11684192-11684193	G	A	CCT->TCT	**TXNDC11**	Pro736Ser; Pro763Ser		0,000	56	16	28,6	61	0	0,00	0,000	0
**B3**	chr19	21726680-21726681	T	C	AAA->GAA	**ZNF100**	Lys542Glu	rs774320913	0,000	55	16	29,1	41	0	0,00	0,003	0
**B3**	chr7	64978323-64978324	A	G	CTC->CCC	**ZNF117**	Leu416Pro		0,000	41	17	41,5	49	0	0,00	0,000	0
**B3**	chr19	21973606-21973607	T	G	AAT->ACT	**ZNF208**	Asn476Thr	rs145224729	0,001	38	10	26,3	73	0	0,00	0,000	47
**B3**	chr19	34943973-34943974	T	A	AGT->AGA	**ZNF30**	Ser337Arg; Ser336Arg		0,000	23	6	26,1	85	0	0,00	0,000	0
**B3**	chr19	21057505-21057506	T	C	TTC->CTC	**ZNF430**	Phe400Leu		0,000	24	10	41,7	34	0	0,00	0,002	0
**B3**	chr16	30397953-30397954	G	A	CGC->CAC	**ZNF48**	Arg235His; Arg112His		na	23	6	26,1	307	1	0,33	0,000	0
**B3**	chr19	22664540-22664541	G	A	AGC->AAC	**ZNF492**	Ser291Asn		0,000	23	6	26,1	102	0	0,00	0,000	0
**B3**	chr4	86446-86447	A	G	AAA->GAA	**ZNF595**	Lys315Glu; Lys132Glu; Lys283Glu		0,000	47	14	29,8	52	0	0,00	0,003	0
**B3**	chr19	20624096-20624097	C	T	AGA->AAA	**ZNF626**	Arg538Lys		0,000	23	6	26,1	38	0	0,00	0,044	0
**B3**	chr19	22180205-22180206	C	G	CGC->CCC	**ZNF676**	Arg504Pro	rs202153135	0,040	35	9	25,7	82	4	4,88	0,000	0
**B3**	chr19	23744686-23744687	T	C	GAC->GGC	**ZNF681**	Asp288Gly		0,000	42	13	31,0	55	0	0,00	0,002	0

**B3**	chr19	21293446-21293447	T	C	AAA->GAA	**ZNF708**	Lys427Glu; Lys507Glu	rs577764108	0,001	28	9	32,1	29	0	0,00	0,005	0
**B3**	chr19	23223391-23223392	T	C	AAA->GAA	**ZNF724P**	Lys285Glu		0,000	21	11	52,4	31	0	0,00	0,000	0
**B3**	chr7	64077907-64077908	A	G	AAA->GAA	**ZNF727**	Lys287Glu	rs572800871	0,000	40	11	27,5	58	0	0,00	0,003	0
**B3**	chr19	22316213-22316214	A	G	AAG->GAG	**ZNF729**	Lys933Glu		na	23	8	34,8	86	0	0,00	0,000	0
**B3**	chr19	23146400-23146401	G	A	GAA->AAA	**ZNF730**	Glu453Lys		na	27	7	25,9	48	0	0,00	0,009	0
**B3**	chr4	272288-272289	C	T	GCT->ACT	**ZNF732**	Ala190Thr		0,000	34	9	26,5	39	0	0,00	0,019	0
**B3**	chr19	53456067-53456068	G	C	GCT->CCT	**ZNF761**	Ala521Pro	rs750870904	0,000	83	24	28,9	139	0	0,00	0,000	0
**B3**	chr19	53408852-53408853	G	C	AGT->ACT	**ZNF765**	Ser433Thr	rs763139884	0,000	38	12	31,6	83	0	0,00	0,000	0
**B3**	chr19	40074644-40074645	T	G	CAA->CAC	**ZNF780A**	Gln565His; Gln599His; Gln600His	rs200594600	0,001	36	10	27,8	91	0	0,00	0,000	0
**B3**	chr19	52613352-52613353	A	T	GAT->GAA	**ZNF83**	Asp404Glu		0,000	25	7	28,0	45	0	0,00	0,008	0
**B3**	chr19	36748791-36748792	T	G	ATT->CTT	**ZNF850**	Ile718Leu; Ile750Leu		na	24	6	25,0	60	0	0,00	0,014	0
**B3**	chr19	23361966-23361967	C	T	GCT->ACT	**ZNF91**	Ala338Thr; Ala306Thr		0,000	45	13	28,9	67	1	1,49	0,000	27
**B4**	chr4	125415455-125415456	G	T	GAA->TAA	**FAT4**	Glu2165*; Glu463*		0,000	55	17	30,9	75	0	0,00	0,000	61

**B4**	chr1	240472379-240472380	A	G	GAA->GGA	**FMN2**	Glu1690Gly; Glu166Gly; Glu10Gly		0,000	57	15	26,3	40	0	0,00	0,007	30
**B4**	chr2	27462772-27462773	A	T	TTT->TTA	**IFT172**	Phe681Leu		0,000	55	14	25,5	53	0	0,00	0,007	17
**B4**	chr21	44551225-44551226	G	A	TCG->TTG	**KRTAP10-2**	Ser78Leu		0,000	387	97	25,1	192	12	6,25	0,000	0
**B4**	chr6	112136187-112136188	C	T	GGT->AGT	**LAMA4**	Gly1117Ser; Gly1110Ser	rs2032567	0,772	60	18	30,0	54	5	9,26	0,000	29
**B4**	chr18	36205160-36205161	G	A	GGA->GAA	**MOCOS**	Gly368Glu		0,000	106	30	28,3	165	0	0,00	0,000	15
**B4**	chr11	47622704-47622705	T	C	AAA->AGA	**MTCH2**	Lys274Arg		na	48	12	25,0	93	2	2,15	0,000	21
**B4**	chr7	100958136-100958137	C	A	CCC->ACC	**MUC3A**	Pro2120Thr		0,000	72	18	25,0	43	2	4,65	0,000	38
**B4**	chr17	47946704-47946705	C	T	CCC->TCC	**PNPO**	Pro194Ser; Pro237Ser		0,000	290	108	37,2	361	0	0,00	0,000	6
**B4**	chr8	92014610-92014611	C	T	GAA->AAA	**RUNX1T1**	Glu109Lys; Glu119Lys; Glu146Lys;		0,000	260	117	45,0	196	0	0,00	0,000	83
**B4**	chr17	63941603-63941604	G	A	CCG->TCG	**SCN4A**	Pro1560Ser		0,000	464	117	25,2	487	0	0,00	0,000	2
**B4**	chr11	20630734-20630735	T	C	TTT->TCT	**SLC6A5**	Phe515Ser		0,000	163	46	28,2	230	0	0,00	0,000	2
**B4**	chr3	98788415-98788416	G	A	GCA->ACA	**ST3GAL6**	Ala119Thr; Ala237Thr; Ala290Thr;		0,000	35	11	31,4	55	0	0,00	0,002	33
**B4**	chr2	178553059-178553060	C	T	CGA->CAA	**TTN**	Arg27379Gln; Arg21074Gln; Arg21007Gln;	rs762912526	0,000	71	20	28,2	88	0	0,00	0,000	10

**B5**	chr10	45847138-45847139	C	T	CGG->CAG	**AGAP4**	Arg70Gln		0,000	31	11	35,5	89	3	3,37	0,000	0
**B5**	chrX	108179378-108179379	C	T	GAT->AAT	**COL4A6**	Asp731Asn; Asp748Asn; Asp732Asn		0,000	21	9	42,9	60	0	0,00	0,000	48
**B5**	chr11	49186714-49186715	G	A	CGG->TGG	**FOLH1**	Arg190Trp; Arg175Trp		na	32	10	31,3	61	1	1,64	0,000	50
**B5**	chr10	133625510-133625511	T	C	GAT->GGT	**FRG2B**	Asp143Gly; Asp142Gly	rs75470891	0,009	81	22	27,2	453	24	5,30	0,000	0
**B5**	chr4	144001282-144001283	T	G	AAA->CAA; GAA->GCA	**GYPB**	Lys41Gln; Glu13Ala	rs201662569	0,265	41	13	31,7	64	2	3,13	0,000	2
**B5**	chr19	8889492-8889493	C	T	GGC->AGC	**MUC16**	Gly13530Ser		0,000	260	72	27,7	353	31	8,78	0,000	74
**B5**	chr1	16565781-16565782	G	A	GCA->GTA	**NBPF1**	Ala972Val	rs773153392	0,119	86	57	66,3	42	1	2,38	0,000	63
**B5**	chr16	14951776-14951777	T	C	TGT->CGT	**NPIPA1**	Cys269Arg	rs146043318	0,001	262	68	26,0	249	0	0,00	0,000	0
**B5**	chr1	248061266-248061267	G	T	GGC->TGC	**OR2L3**	Gly196Cys	rs75239130	0,478	337	105	31,2	506	29	5,73	0,000	0
**B5**	chr13	25096712-25096713	A	G	CAA->CGA	**PABPC3**	Gln172Arg	rs75475407	0,004	20	8	40,0	115	7	6,09	0,000	0
**B5**	chrX	154400836-154400837	C	T	CGG->TGG	**RPL10**	Arg159Trp; Arg210Trp;	rs979369776	0,000	22	11	50,0	73	2	2,74	0,000	22
**B5**	chr1	228401040-228401041	G	A	CAG->TAG	**TRIM11**	Gln95*; Gln220*		0,000	32	9	28,1	38	0	0,00	0,018	42
**B6**	chr8	24339521-24339522	G	A	GGG->AGG	**ADAM28**	Gly542Arg	rs191658345	0,000	104	27	26,0	80	0	0,00	0,000	47

**B6**	chr16	49378476-49378477	G	A	GGA->GAA	**C16orf78**	Gly93Glu		0,000	144	36	25,0	128	0	0,00	0,000	0
**B6**	chr12	2597269-2597270	C	T	ACC->ATC	**CACNA1C**	Thr970Ile; Thr945Ile		0,000	56	14	25,0	37	0	0,00	0,022	6
**B6**	chr19	38367230-38367231	G	A	GAA->AAA	**CATSPERG**	Glu897Lys; Glu857Lys		0,000	204	64	31,4	98	0	0,00	0,000	0
**B6**	chr1	6130319-6130320	C	T	GCC->ACC	**CHD5**	Ala1091Thr; Ala40Thr		0,000	468	117	25,0	219	0	0,00	0,000	63
**B6**	chr17	50199305-50199306	G	A	CGA->TGA	**COL1A1**	Arg131*; Arg149*		0,000	148	44	29,7	48	0	0,00	0,000	18
**B6**	chr13	42215641-42215642	C	T	TCT->TTT	**DGKH**	Ser1063Phe; Ser927Phe		0,000	120	37	30,8	133	0	0,00	0,000	9
**B6**	chrX	66599814-66599815	G	A	TCT->TTT	**EDA2R**	Ser209Phe; Ser188Phe		0,000	139	70	50,4	152	0	0,00	0,000	40
**B6**	chr14	73976331-73976332	G	A	CAG->TAG	**ENTPD5**	Gln212*		0,000	168	43	25,6	244	0	0,00	0,000	55
**B6**	chr5	128334780-128334781	C	T	GGA->GAA	**FBN2**	Gly196Glu; Gly1345Glu; Gly1313Glu;		0,000	96	32	33,3	71	0	0,00	0,000	14
**B6**	chr11	67451817-67451818	G	A	CGG->TGG	**GPR152**	Arg303Trp	rs147242558	0,000	1471	391	26,6	418	1	0,24	0,000	0
**B6**	chr12	52949299-52949300	G	C	GGT->CGT	**KRT18**	Gly43Arg		0,000	31	15	48,4	10	0	0,00	0,007	40
**B6**	chr2	140485426-140485427	G	A	TCC->TTC	**LRP1B**	Ser3114Phe		0,000	102	28	27,5	129	0	0,00	0,000	54
**B6**	chr3	169115694-169115695	G	A	TCA->TTA	**MECOM**	Ser603Leu; Ser538Leu; Ser539Leu;		0,000	178	60	33,7	152	0	0,00	0,000	77

**B6**	chr1	16565781-16565782	G	A	GCA->GTA	**NBPF1**	Ala972Val	rs773153392	0,119	71	25	35,2	40	0	0,00	0,000	63
**B6**	chr1	228365517-228365518	C	A	CAG->AAG	**OBSCN**	Gln6341Lys; Gln7298Lys		0,000	798	308	38,6	227	1	0,44	0,000	20
**B6**	chr6	154091407-154091408	G	A	CGA->CAA	**OPRM1**	Arg460Gln; Arg286Gln; Arg267Gln;	rs201516315	0,000	100	29	29,0	62	0	0,00	0,000	12
**B6**	chr17	7502094-7502095	G	A	GAC->AAC	**POLR2A**	Asp849Asn		0,000	332	89	26,8	169	0	0,00	0,000	44
**B6**	chr4	75876434-75876435	C	T	CGG->CAG	**PPEF2**	Arg391Gln	rs137955024	0,000	388	98	25,3	179	1	0,56	0,000	0
**B6**	chr18	22993246-22993247	C	T	CCA->TCA	**RBBP8**	Pro474Ser		0,000	76	21	27,6	81	0	0,00	0,000	66
**B6**	chr13	98176458-98176459	G	A	CGC->TGC	**RNF113B**	Arg260Cys		0,000	868	223	25,7	364	0	0,00	0,000	0
**B6**	chr1	154966413-154966414	C	+C		**SHC1**	Val363; Val134; Val253; Val116	rs746556543	1,000	448	122	27,2	220	1	0,45	0,000	36
**B6**	chr4	76739280-76739281	G	A	GAG->AAG	**SHROOM3**	Glu370Lys		0,000	608	157	25,8	263	0	0,00	0,000	11
**B6**	chrX	30854948-30854949	G	A	TCA->TTA	**TAB3**	Ser239Leu	rs150176434	0,001	150	87	58,0	208	0	0,00	0,000	49
**B6**	chr1	19700875-19700876	C	T	GGA->GAA	**TMCO4**	Gly425Glu		0,000	297	75	25,3	168	0	0,00	0,000	0
**B6**	chr4	84821341-84821342	A	T	TTT->ATT	**WDFY3**	Phe445Ile		0,000	95	25	26,3	81	0	0,00	0,000	21
**B7**	chr2	95944650-95944651	C	A	TTG->TTT	**ANKRD36C**	Leu489Phe		0,000	42	12	28,6	64	2	3,13	0,000	0

**B7**	chr15	20534992-20534993	T	C	AAG->GAG	**GOLGA6L6**	Lys481Glu	rs756554667	0,002	70	26	37,1	35	2	5,71	0,000	0
**B7**	chr14	106235208-106235209	G	T	TCC->TAC	**IGHV3-21**	Ser69Tyr		0,000	124	41	33,1	266	20	7,52	0,000	0
**B7**	chr19	8908636-8908637	C	G	GGA->GCA	**MUC16**	Gly12525Ala		na	136	43	31,6	212	11	5,19	0,000	74
**B7**	chr1	16565781-16565782	G	A	GCA->GTA	**NBPF1**	Ala972Val	rs773153392	0,119	78	20	25,6	120	0	0,00	0,000	63
**B7**	chr1	120807626-120807627	C	G	CTC->GTC	**NBPF26**	Leu66Val		0,000	73	24	32,9	105	7	6,67	0,000	0
**B7**	chr16	14951776-14951777	T	C	TGT->CGT	**NPIPA1**	Cys269Arg	rs146043318	0,001	24	14	58,3	5	0	0,00	0,042	0
**B7**	chr1	248061266-248061267	G	T	GGC->TGC	**OR2L3**	Gly196Cys	rs75239130	0,478	569	148	26,0	474	40	8,44	0,000	0
**B7**	chr13	25097528-25097529	A	T	AAG->ATG	**PABPC3**	Lys444Met	rs75484271	0,010	42	11	26,2	185	14	7,57	0,000	0
**B7**	chr3	75738766-75738767	C	T	GTT->ATT	**ZNF717**	Val236Ile	rs78153004	0,250	57	16	28,1	71	4	5,63	0,000	0
**H4**	chr17	76468845-76468846	G	T	GCC->TCC	**AANAT**	Ala79Ser; Ala34Ser; Ala72Ser		0,000	180	67	37,2	145	0	0,00	nd	6
**H4**	chr6	30571512-30571513	C	T	CCG->CTG	**ABCF1**	Pro9Leu	rs767397757	0,000	75	24	32,0	43	0	0,00	nd	35
**H4**	chr11	74701644-74701645	A	T	GAT->GAA	**CHRDL2**	Asp374Glu		0,000	23	7	30,4	81	0	0,00	nd	25
**H4**	chr2	77518606-77518607	G	T	TCA->TAA	**LRRTM4**	Ser422*; Ser421*		0,000	77	23	29,9	164	0	0,00	nd	15

**H4**	chr1	113619803-113619804	A	T	CAG->CTG	**MAGI3**	Gln407Leu; Gln382Leu		0,000	32	10	31,3	76	0	0,00	nd	47
**H4**	chr1	46594875-46594876	G	A	GCA->GTA	**MKNK1**	Ala23Val		0,000	36	9	25,0	41	0	0,00	nd	55
**H4**	chrX	105945970-105945971	C	T	CGG->TGG	**NRK**	Arg1387Trp	rs372868547	0,000	40	15	37,5	97	0	0,00	nd	28
**H4**	chr5	35068264-35068265	G	A	CCG->CTG	**PRLR**	Pro269Leu; Pro168Leu	rs754974807	0,000	50	14	28,0	150	0	0,00	nd	32
**H4**	chr11	70487390-70487391	T	C	AGT->GGT	**SHANK2**	Ser380Gly; Ser372Gly; Ser968Gly		0,000	123	33	26,8	449	0	0,00	nd	11
**H4**	chr20	17968360-17968361	T	A	CAG->CTG	**SNX5**	Gln22Leu		0,000	36	10	27,8	42	0	0,00	nd	14
**H4**	chr3	48620842-48620843	C	A	TGA->TTA	**TMEM89**	*160Leu		0,000	109	34	31,2	526	0	0,00	nd	0
**H4**	chr15	43092031-43092032	G	C	CGC->GGC	**UBR1**	Arg44Gly		0,000	22	6	27,3	24	0	0,00	nd	11
**H4**	chr7	141719210-141719211	G	A	CGC->CAC	**WEE2**	Arg242His	rs752743808	0,000	24	9	37,5	42	0	0,00	nd	17
**H4**	chr3	75737532-75737533	G	T	ACA->AAA	**ZNF717**	Thr647Lys	rs201345045	0,407	25	7	28,0	48	3	6,25	nd	0
**H5**	chr22	17500721-17500722	G	T	GAG->TAG	**CECR2**	Glu50*; Glu213*; Glu191*		0,000	47	4	8,5	37	0	0,00	nd	14
**H5**	chr2	70816486-70816487	T	A	TTA->TTT	**CLEC4F**	Leu298Phe		0,000	236	24	10,2	250	0	0,00	nd	11
**H5**	chr13	110183001-110183002	C	T	GGC->AGC	**COL4A1**	Gly696Ser	rs587780588	na	66	7	10,6	84	0	0,00	nd	18

**H5**	chr17	64506287-64506288	G	C	NonCoding	**DDX5**	NonCoding		0,000	161	13	8,1	159	0	0,00	nd	50
**H5**	chr5	107861781-107861782	T	C	AGG->GGG	**FBXL17**	Arg444Gly; Arg284Gly; Arg682Gly		0,000	507	48	9,5	538	0	0,00	nd	0
**H5**	chrX	49332762-49332763	A	G	TAT->TGT	**GAGE13**	Tyr9Cys		0,000	32	16	50,0	25	0	0,00	nd	0
**H5**	chr4	16502712-16502713	G	A	CCG->CTG	**LDB2**	Pro349Leu; Pro351Leu	rs751472745	0,000	442	42	9,5	489	0	0,00	nd	23
**H5**	chr3	111193235-111193236	G	C	GAG->CAG	**NECTIN3**	Glu441Gln		0,000	137	16	11,7	143	0	0,00	nd	0
**H5**	chr11	59422569-59422570	G	T	TGC->TGA	**OR5A2**	Cys128*	rs370169877	0,000	287	30	10,5	276	1	0,36	nd	0
**H5**	chr15	90961670-90961671	C	T	CGT->TGT	**RCCD1**	Arg345Cys; Arg343Cys	rs760490428	0,000	305	26	8,5	387	0	0,00	nd	23
**H5**	chrX	124056161-124056162	G	T	GAA->TAA	**STAG2**	Glu411*		0,000	413	48	11,6	342	3	0,88	nd	66
**H6548**	chr17	76468753-76468754	C	T	ACG->ATG	**AANAT**	Thr48Met; Thr3Met	rs61739395	0,008	140	35	25,0	138	0	0,0	0,000	5
**H6548**	chr17	81181216-81181217	C	G	CAG->GAG	**AATK-AS1**	Gln226Glu	rs7209980	0,113	189	69	36,5	198	0	0,0	0,000	0
**H6548**	chr11	111456535-111456536	C	A	TGC->TGA	**AB231705**	Cys190*	rs12270679	0,059	63	30	47,6	108	0	0,0	0,000	0
**H6548**	chr9	104858585-104858586	C	T	AGG->AAG	**ABCA1**	Arg219Lys	rs2230806	0,334	44	14	31,8	75	0	0,0	0,000	13
**H6548**	chr17	69150007-69150008	G	A	CGG->TGG	**ABCA10**	Arg477Trp; Arg1485Trp	rs141260911	0,005	43	14	32,6	83	0	0,0	0,000	28

**H6548**	chr7	48278341-48278342	T	G	ATA->AGA	**ABCA13**	Ile2383Arg; Ile64Arg	rs200243325	0,002	98	27	27,6	149	0	0,0	0,000	30
**H6548**	chr9	137022799-137022800	C	T	CGG->CAG	**ABCA2**	Arg44Gln; Arg113Gln; Arg143Gln	rs759220923	0,000	246	83	33,7	109	0	0,0	0,000	38
**H6548**	chr17	69105576-69105577	C	T	ATG->ATA	**ABCA6**	Met875Ile	rs7212506	0,872	26	10	38,5	57	0	0,0	0,000	31
**H6548**	chr17	68989850-68989851	T	G	AAA->ACA	**ABCA9**	Lys1268Thr; Lys1306Thr	rs2302294	0,567	64	17	26,6	96	0	0,0	0,000	31
**H6548**	chr7	151033878-151033879	C	G	CTG->GTG	**ABCB8**	Leu161Val; Leu141Val; Leu124Val	rs117793104	0,007	132	44	33,3	217	1	0,5	0,000	31
**H6548**	chr16	48231865-48231866	C	T	CGT->CAT	**ABCC11**	Arg19His	rs16945988	0,094	61	18	29,5	137	0	0,0	0,000	44
**H6548**	chr17	50683691-50683692	G	A	CGC->CAC	**ABCC3**	Arg1297His; Arg51His	rs11568591	0,036	229	70	30,6	283	0	0,0	0,000	50
**H6548**	chr2	43837950-43837951	G	A	CGC->TGC	**ABCG5**	Arg50Cys	rs6756629	0,064	42	11	26,2	114	0	0,0	0,000	9
**H6548**	chr20	25302330-25302331	C	T	GCA->ACA	**ABHD12**	Ala349Thr	rs746748	0,042	61	22	36,1	83	0	0,0	0,000	8
**H6548**	chr3	100839571-100839572	T	C	ACT->GCT	**ABI3BP**	Thr648Ala	rs9841585	0,253	77	22	28,6	103	0	0,0	0,000	38
**H6548**	chr12	120738180-120738181	T	C	CTG->CCG	**ACADS**	Leu202Pro	rs555404	0,579	139	63	45,3	205	0	0,0	0,000	6
**H6548**	chr11	44067801-44067802	G	A	GAT->AAT	**ACCS**	Asp59Asn	rs33952257	0,186	60	17	28,3	147	0	0,0	0,000	59
**H6548**	chr19	6312278-6312279	T	C	ATG->GTG	**ACER1**	Met74Val	rs72981971	0,158	72	20	27,8	201	0	0,0	0,000	25

**H6548**	chr7	100893175-100893176	G	T	CAC->AAC	**ACHE**	His353Asn; His414Asn	rs1799805	0,042	167	50	29,9	220	2	0,9	0,000	14
**H6548**	chr2	110841380-110841381	C	T	ACG->ATG	**ACOXL**	Thr78Met; Thr255Met	rs1554005	0,206	69	19	27,5	108	0	0,0	0,000	39
**H6548**	chr19	50794567-50794568	G	A	GCC->ACC	**ACPT**	Ala325Thr	rs55716643	0,305	97	26	26,8	195	1	0,5	0,000	8
**H6548**	chr22	50744826-50744827	A	G	ATG->GTG	**ACR**	Met296Val	rs5771002	0,732	100	25	25,0	133	0	0,0	0,000	17
**H6548**	chr9	108862461-108862462	C	A	ACG->AAG	**ACTL7A**	Thr47Lys		0,000	90	24	26,7	227	0	0,0	0,000	26
**H6548**	chr11	66560623-66560624	C	T	CGA->TGA	**ACTN3**	Arg620*; Arg577*	rs1815739	0,450	106	30	28,3	166	0	0,0	0,000	2
**H6548**	chr16	84195163-84195164	G	C	GGG->CGG	**ADAD2**	Gly307Arg; Gly235Arg	rs11149631	0,261	385	120	31,2	297	0	0,0	0,000	0
**H6548**	chr14	70470719-70470720	C	T	AGA->AAA	**ADAM20P1**	Arg166Lys	rs45447692	0,022	92	27	29,4	164	0	0,0	0,000	0
**H6548**	chr14	70457884-70457885	T	G	TTT->TGT	**ADAM21**	Phe129Cys	rs72735759	0,246	56	21	37,5	139	0	0,0	0,000	28
**H6548**	chr7	88125599-88125600	G	A	GTA->ATA	**ADAM22**	Val206Ile; Val207Ile; Val259Ile	rs17255978	0,065	60	16	26,7	95	0	0,0	0,000	21
**H6548**	chr20	3671117-3671118	C	T	GTC->ATC	**ADAM33**	Val206Ile; Val710Ile; Val684Ile	rs3918396	0,100	173	50	28,9	99	0	0,0	0,000	10
**H6548**	chr8	39473573-39473574	T	C	AGA->GGA	**ADAM3A**	Arg166Gly; Arg215Gly	rs7845028	0,370	25	17	68,0	26	0	0,0	0,000	19
**H6548**	chr11	130462561-130462562	T	C	TAC->CAC	**ADAMTS15**	Tyr442His	rs116897071	0,020	309	90	29,1	356	0	0,0	0,000	42

**H6548**	chr15	100281370-100281371	G	A	TCG->TTG	**ADAMTS17**	Ser216Leu	rs7496668	0,428	171	99	57,9	118	0	0,0	0,000	13
**H6548**	chr11	130405853-130405854	T	C	ACA->GCA	**ADAMTS8**	Thr273Ala; Thr792Ala	rs11222085	0,142	161	63	39,1	381	0	0,0	0,000	44
**H6548**	chr7	140694786-140694787	C	T	CCA->CTA	**ADCK2**	Pro622Leu	rs1046515	0,106	84	23	27,4	167	0	0,0	0,000	0
**H6548**	chr16	3983434-3983435	T	C	ATA->ATG	**ADCY9**	Ile772Met	rs2230739	0,351	113	44	38,9	226	0	0,0	0,000	19
**H6548**	chr10	114044276-114044277	A	G	AGC->GGC	**ADRB1**	Ser49Gly	rs1801252	0,291	229	70	30,6	132	0	0,0	0,000	4
**H6548**	chr2	99594179-99594180	T	C	AAT->AGT	**AFF3**	Asn494Ser; Asn519Ser; Asn647Ser	rs1047265	0,182	208	120	57,7	343	0	0,0	0,000	28
**H6548**	chr4	73455259-73455260	C	T	CGT->TGT	**AFP**	Arg604Cys; Arg378Cys	rs555650673	0,000	68	19	27,9	86	0	0,0	0,000	34
**H6548**	chr14	104949919-104949920	G	T	GCC->GAC	**AHNAK2**	Ala1744Asp; Ala1844Asp	rs201728587	0,009	429	119	27,7	725	1	0,1	0,000	53
**H6548**	chr5	427906-427907	C	T	GCG->GTG	**AHRR**	Ala292Val; Ala120Val; Ala274Val;	rs183151904	0,001	189	54	28,6	192	1	0,5	0,000	35
**H6548**	chr1	26344117-26344118	C	T	CGC->CAC	**AIM1L**	Arg847His	rs34370465	0,211	115	34	29,6	274	0	0,0	0,000	0
**H6548**	chr12	132552778-132552779	G	A	GGC->AGC	**AK096230**	Gly227Ser	rs113109626	0,006	259	81	31,3	163	0	0,0	0,000	0
**H6548**	chr16	84194698-84194699	C	G	GTA->CTA	**AK123582**	Val20Leu	rs11864916	0,287	43	19	44,2	77	0	0,0	0,000	0
**H6548**	chr16	629952-629953	C	T	ACC->ATC	**AK128777**	Thr152Ile	rs17139376	0,208	69	22	31,9	81	0	0,0	0,000	0

**H6548**	chr9	132878212-132878213	G	A	CCC->TCC	**AK8**	Pro15Ser		0,000	204	65	31,9	209	0	0,0	0,000	18
**H6548**	chr15	85743555-85743556	C	T	CCC->CTC	**AKAP13**	Pro2708Leu; Pro2712Leu; Pro1329Leu;	rs61734378	0,054	76	23	30,3	101	0	0,0	0,000	44
**H6548**	chr14	32824324-32824325	T	A	TTC->TAC	**AKAP6**	Phe2171Tyr	rs4647899	0,289	112	45	40,2	234	0	0,0	0,000	10
**H6548**	chr1	108852635-108852636	G	T	ACG->AAG	**AKNAD1**	Thr10Lys	rs41279674	0,001	85	31	36,5	149	0	0,0	0,000	0
**H6548**	chr10	5096554-5096555	A	G	GAA->GGA	**AKR1C3**	Glu77Gly; Glu54Gly	rs11551177	0,050	61	26	42,6	110	0	0,0	0,000	59
**H6548**	chr4	99649843-99649844	T	C	CAG->CGG	**AL832378**	Gln19Arg	rs1491242	0,101	143	44	30,8	194	0	0,0	0,000	0
**H6548**	chr3	105540016-105540017	A	G	AAT->AGT	**ALCAM**	Asn258Ser; Asn207Ser	rs1044240	0,140	95	51	53,7	131	0	0,0	0,000	46
**H6548**	chr15	58279201-58279202	A	T	ATG->AAG	**ALDH1A2**	Met1Lys	rs72739147	0,137	85	33	38,8	153	0	0,0	0,000	22
**H6548**	chr5	126595257-126595258	C	T	GCG->ACG	**ALDH7A1**	Ala8Thr	rs144272515	0,028	76	36	47,4	78	0	0,0	0,000	13
**H6548**	chr11	78114273-78114274	T	C	AAT->AGT	**ALG8**	Asn222Ser	rs665278	0,197	74	22	29,7	123	0	0,0	0,000	4
**H6548**	chr2	73489975-73489976	G	C	GAT->CAT	**ALMS1**	Asp2060His; Asp2673His; Asp2631His	rs2017116	0,141	69	22	31,9	176	0	0,0	0,000	7
**H6548**	chr4	112423990-112423991	A	G	AAT->GAT	**ALPK1**	Asn175Asp; Asn97Asp	rs6533616	0,076	58	17	29,3	117	0	0,0	0,000	34
**H6548**	chr18	58537699-58537700	T	G	AAA->AAC	**ALPK2**	Lys160Asn; Lys829Asn	rs3809973	0,466	104	32	30,8	161	0	0,0	0,000	34

**H6548**	chr5	34007994-34007995	C	T	GTG->ATG	**AMACR**	Val9Met	rs3195676	0,417	294	81	27,6	210	0	0,0	0,000	84
**H6548**	chr19	2249384-2249385	C	T	GCT->GTT	**AMH**	Ala18Val	rs61736578	0,002	186	76	40,9	155	0	0,0	0,000	22
**H6548**	chr11	118198036-118198037	T	C	ATA->ATG	**AMICA1**	Ile132Met; Ile283Met; Ile312Met;	rs2298831	0,180	115	45	39,1	211	0	0,0	0,000	0
**H6548**	chr4	70518819-70518820	C	T	CGG->TGG	**AMTN**	Arg15Trp	rs35286445	0,127	82	27	32,9	129	0	0,0	0,000	13
**H6548**	chr15	64943579-64943580	A	G	AAG->GAG	**ANKDD1A**	Lys355Glu; Lys264Glu	rs34988193	0,314	110	36	32,7	139	0	0,0	0,000	0
**H6548**	chr17	56457272-56457273	G	A	GTT->ATT	**ANKFN1**	Val445Ile	rs10852985	0,743	27	19	70,4	62	0	0,0	0,000	0
**H6548**	chr19	17282720-17282721	T	A	CTG->CAG	**ANKLE1**	Leu148Gln; Leu137Gln; Leu116Gln	rs8108174	0,506	197	50	25,4	107	0	0,0	0,000	53
**H6548**	chr2	240524035-240524036	C	A	GTG->TTG	**ANKMY1**	Val561Leu; Val472Leu; Val331Leu;	rs3821348	0,172	351	208	59,3	335	0	0,0	0,000	0
**H6548**	chr10	37216263-37216264	C	T	CGT->TGT	**ANKRD30A**	Arg929Cys	rs1200875	0,198	61	32	52,5	120	0	0,0	0,000	49
**H6548**	chr2	197126016-197126017	A	C	ATT->ATG	**ANKRD44**	Ile69Met; Ile94Met; Ile22Met	rs35338671	0,043	87	23	26,4	164	0	0,0	0,000	0
**H6548**	chr2	70978677-70978678	G	-C^G		**ANKRD53**	Ala11		0,000	106	29	27,4	42	0	0,0	0,000	0
**H6548**	chr5	56111714-56111715	C	T	GTG->ATG	**ANKRD55**	Val57Met; Val345Met	rs321776	0,241	52	24	46,2	104	0	0,0	0,000	10
**H6548**	chr16	21250536-21250537	G	A	GGC->GAC	**ANKS4B**	Gly324Asp		0,000	144	36	25,0	206	0	0,0	0,000	0

**H6548**	chr11	70161663-70161664	T	C	TTC->TCC	**ANO1**	Phe550Ser; Phe608Ser; Phe317Ser	rs2186797	0,037	117	33	28,2	162	0	0,0	0,000	51
**H6548**	chr4	164197643-164197644	G	A	GCC->GTC	**ANP32C**	Ala23Val	rs2288674	0,419	35	9	25,7	81	0	0,0	0,000	0
**H6548**	chr19	2118692-2118693	C	T	GGG->AGG	**AP3D1**	Gly269Arg; Gly541Arg	rs34569645	0,125	172	48	27,9	161	0	0,0	0,000	9
**H6548**	chr1	113896328-113896329	A	G	TTG->TCG	**AP4B1**	Leu7Ser; Leu274Ser; Leu480Ser;	rs1217401	0,321	76	35	46,1	142	0	0,0	0,000	0
**H6548**	chr19	3752875-3752876	A	G	TGC->CGC	**APBA3**	Cys134Arg; Cys376Arg	rs8102086	0,562	449	132	29,4	361	1	0,3	0,000	37
**H6548**	chr2	68502859-68502860	A	G	ATT->GTT	**APLF**	Ile76Val; Ile100Val	rs11902811	0,081	58	20	34,5	107	0	0,0	0,000	50
**H6548**	chr2	21007032-21007033	T	C	AGC->GGC	**APOB**	Ser3279Gly	rs12720854	0,005	115	29	25,2	164	0	0,0	0,000	7
**H6548**	chr22	39102032-39102033	G	C	GAG->GAC	**APOBEC3H**	Glu178Asp	rs139302	0,480	109	32	29,4	116	0	0,0	0,000	35
**H6548**	chr17	66226045-66226046	C	T	AGT->AAT	**APOH**	Ser107Asn	rs1801692	0,040	93	36	38,7	147	0	0,0	0,000	10
**H6548**	chr22	36191462-36191463	C	T	CGT->CAT	**APOL4**	Arg220His; Arg223His	rs2227168	0,553	157	60	38,2	298	0	0,0	0,000	0
**H6548**	chr22	35726676-35726677	C	A	AGC->AGA	**APOL5**	Ser203Arg	rs73155887	0,009	64	22	34,4	166	0	0,0	0,000	0
**H6548**	chr3	57269655-57269656	A	G	GAA->GGA	**APPL1**	Glu700Gly	rs11544593	0,127	95	25	26,3	151	0	0,0	0,000	24
**H6548**	chr2	240682769-240682770	C	T	CGG->CAG	**AQP12B**	Arg23Gln	rs4081909	0,170	607	313	51,6	364	0	0,0	0,000	0

**H6548**	chr5	115962654-115962655	G	A	CGC->CAC	**AQPEP**	Arg13His	rs780442231	0,000	187	56	30,0	86	0	0,0	-2,000	0
**H6548**	chr11	47166859-47166860	C	T	AGC->AAC	**ARFGAP2**	Ser142Asn; Ser411Asn; Ser383Asn;	rs3740691	0,328	106	38	35,9	213	0	0,0	0,000	18
**H6548**	chr16	24939523-24939524	C	T	GCT->ACT	**ARHGAP17**	Ala46Thr; Ala249Thr; Ala522Thr	rs145791095	0,008	135	41	30,4	143	0	0,0	0,000	21
**H6548**	chr2	68822808-68822809	T	C	ATG->ACG	**ARHGAP25**	Met557Thr; Met517Thr; Met549Thr;	rs10177248	0,314	91	26	28,6	139	0	0,0	0,000	25
**H6548**	chr17	45395458-45395459	G	C	CAC->CAG	**ARHGAP27**	His521Gln; His548Gln; His889Gln	rs117139057	0,140	303	79	26,1	117	0	0,0	0,000	0
**H6548**	chr22	44862452-44862453	C	G	CCT->CGT	**ARHGAP8**	Pro597Arg; Pro440Arg; Pro418Arg;	rs9614957	0,087	141	84	59,6	240	0	0,0	0,000	45
**H6548**	chr19	41896715-41896716	C	T	CCT->CTT	**ARHGEF1**	Pro452Leu	rs79725225	0,047	59	43	72,9	52	0	0,0	0,000	34
**H6548**	chr1	156937322-156937323	T	C	AGC->GGC	**ARHGEF11**	Ser832Gly; Ser1456Gly; Ser1416Gly	rs868188	0,395	113	30	26,6	172	0	0,0	0,000	30
**H6548**	chr1	16208150-16208151	C	G	GGC->CGC	**ARHGEF19**	Gly163Arg	rs221058	0,269	256	82	32,0	187	0	0,0	0,000	0
**H6548**	chr12	57616379-57616380	A	G	CAG->CGG	**ARHGEF25**	Gln545Arg; Gln400Arg; Gln506Arg	rs1564374	0,626	79	20	25,3	145	0	0,0	0,000	0
**H6548**	chr5	73852655-73852656	G	A	AGA->AAA	**ARHGEF28**	Arg585Lys; Arg272Lys	rs2973566	0,238	53	17	32,1	123	0	0,0	0,000	30
**H6548**	chr5	149628903-149628904	C	A	CCC->ACC	**ARHGEF37**	Pro586Thr	rs3733662	0,163	43	22	51,2	70	0	0,0	0,000	0
**H6548**	chr11	13356909-13356910	C	T	GCG->GTG	**ARNTL**	Ala10Val	rs62620045	0,054	134	40	29,9	256	0	0,0	0,000	15

**H6548**	chr3	9829345-9829346	T	G	ATG->AGG	**ARPC4-TTLL3**	Met563Arg; Met146Arg; Met234Arg;	rs2290305	0,596	36	17	47,2	63	0	0,0	0,000	0
**H6548**	chr12	14840504-14840505	C	T	GAC->AAC	**ART4**	Asp265Asn	rs11276	0,347	80	38	47,5	120	0	0,0	0,000	19
**H6548**	chr22	29793652-29793653	C	T	CGG->CAG	**ASCC2**	Arg571Gln; Arg495Gln	rs61736786	0,053	85	23	27,1	137	0	0,0	0,000	0
**H6548**	chr6	100848512-100848513	G	A	CTT->TTT	**ASCC3**	Leu146Phe; Leu48Phe	rs9390698	0,360	72	37	51,4	158	0	0,0	0,000	32
**H6548**	chr16	29901481-29901482	G	+GGT		**ASPHD1**	Gly171	rs140411458	0,333	615	242	39,4	284	0	0,0	0,000	0
**H6548**	chr17	81996667-81996668	T	A	CTG->CAG	**ASPSCR1**	Leu252Gln; Leu175Gln	rs8074498	0,492	240	81	33,8	143	0	0,0	0,000	68
**H6548**	chr9	116686699-116686700	C	T	GAA->AAA	**ASTN2**	Glu36Lys	rs10983304	0,024	49	14	28,6	89	0	0,0	0,000	9
**H6548**	chr1	1490669-1490670	A	C	CAG->CCG	**ATAD3B**	Gln538Pro; Gln492Pro; Gln168Pro	rs138009989	0,013	262	74	28,2	218	0	0,0	0,000	44
**H6548**	chr22	39521509-39521510	A	C	CAG->CCG	**ATF4**	Gln22Pro	rs4894	0,305	42	23	54,8	66	2	3,0	0,000	37
**H6548**	chr1	161781950-161781951	A	G	ATG->GTG	**ATF6**	Met67Val	rs1058405	0,233	90	24	26,7	160	0	0,0	0,000	29
**H6548**	chr3	11358544-11358545	T	C	GTC->GCC	**ATG7**	Val471Ala; Val432Ala	rs36117895	0,033	60	15	25,0	173	0	0,0	0,000	38
**H6548**	chr2	85754637-85754638	T	C	CTG->CCG	**ATOH8**	Leu150Pro	rs17851881	0,602	233	127	54,5	98	1	1,0	0,000	15
**H6548**	chr4	47576953-47576954	G	A	AGA->AAA	**ATP10D**	Arg1183Lys; Arg431Lys	rs16851681	0,271	99	49	49,5	186	0	0,0	0,000	12

**H6548**	chr1	16986247-16986248	C	T	CCG->CCA; CCG->CCA	**ATP13A2**	Pro1172Pro; Pro1167Pro; Ala1072Thr	rs3170740	0,498	370	178	48,1	169	0	0,0	0,000	5
**H6548**	chr17	3947698-3947699	C	T	GCC->ACC	**ATP2A3**	Ala263Thr	rs753190807	0,000	815	205	25,2	341	0	0,0	0,000	25
**H6548**	chr1	111449472-111449473	C	T	CCT->TCT	**ATP5F1**	Pro125Ser	rs1264899	0,448	107	28	26,2	161	0	0,0	0,000	21
**H6548**	chr2	70936042-70936043	C	T	ACC->ATC	**ATP6V1B1**	Thr30Ile	rs17720303	0,179	96	24	25,0	85	0	0,0	-2,000	4
**H6548**	chr13	51970668-51970669	C	G	GTG->CTG	**ATP7B**	Val345Leu; Val456Leu; Val424Leu	rs1801244	0,432	116	34	29,3	268	0	0,0	0,000	17
**H6548**	chr19	1806667-1806668	C	T	GCC->ACC	**ATP8B3**	Ala160Thr; Ala213Thr	rs45574836	0,094	209	65	31,1	171	1	0,6	0,000	0
**H6548**	chrX	77682470-77682471	C	G	GAG->CAG	**ATRX**	Glu929Gln; Glu891Gln; Glu714Gln;	rs3088074	0,601	58	29	50,0	74	0	0,0	0,000	60
**H6548**	chr16	28826365-28826366	G	A	GTT->ATT	**ATXN2L**	Val198Ile	rs117987062	0,008	97	28	28,9	168	0	0,0	0,000	0
**H6548**	chr7	105613787-105613788	C	T	CGG->CAG	**ATXN7L1**	Arg509Gln; Arg633Gln	rs150948758	0,006	371	100	27,0	342	0	0,0	0,000	0
**H6548**	chr1	25835821-25835822	T	G	AAG->ACG	**AUNIP**	Lys82Thr	rs34449716	0,041	76	22	29,0	183	0	0,0	0,000	0
**H6548**	chr15	34003095-34003096	C	G	GAG->GAC	**AVEN**	Glu127Asp	rs202188583	0,000	58	17	29,3	81	0	0,0	0,000	11
**H6548**	chr1	206116319-206116320	C	G	GGC->CGC	**AVPR1B**	Gly191Arg	rs33990840	0,947	156	53	34,0	235	0	0,0	0,000	8,03
**H6548**	chr20	5316004-5316005	T	G	TGT->GGT	**AX746654**	Cys141Gly	rs910973	0,228	165	70	42,4	189	1	0,5	0,000	0

**H6548**	chr5	79991007-79991008	C	G	GAG->GAC	**AX746944**	Glu77Asp	rs34727832	0,196	376	117	31,1	299	0	0,0	0,000	0
**H6548**	chr15	32718534-32718535	G	A	CTC->TTC	**AX747968**	Leu2Phe	rs2293581	0,316	277	96	34,7	123	0	0,0	0,000	0
**H6548**	chr12	122204633-122204634	C	G	CCT->GCT	**B3GNT4**	Pro6Ala	rs7136356	0,373	99	31	31,3	88	0	0,0	-2,000	0
**H6548**	chr6	89951522-89951523	C	T	GCC->ACC	**BACH2**	Ala195Thr	rs143586122	0,002	152	39	25,7	251	0	0,0	0,000	42
**H6548**	chr6	31642908-31642909	A	G	TCT->CCT	**BAG6**	Ser619Pro; Ser625Pro	rs1052486	0,531	134	60	44,8	193	0	0,0	0,000	34
**H6548**	chr17	81445620-81445621	G	A	GCC->ACC	**BAHCC1**	Ala1035Thr; Ala234Thr	rs368906845	0,003	177	49	27,7	110	0	0,0	0,000	0
**H6548**	chr16	88033110-88033111	C	T	CCG->TCG	**BANP**	Pro253Ser; Pro317Ser; Pro323Ser;	rs74992447	0,150	415	235	56,6	285	0	0,0	0,000	44
**H6548**	chr2	214752453-214752454	C	G	TGC->TCC	**BARD1**	Cys557Ser; Cys123Ser; Cys413Ser;	rs28997576	0,015	51	13	25,5	85	0	0,0	0,000	84
**H6548**	chr11	129436752-129436753	T	C	TCC->CCC	**BARX2**	Ser64Pro	rs142822705	0,012	85	31	36,5	72	0	0,0	0,000	22
**H6548**	chr1	212699731-212699732	C	T	GTC->ATC	**BATF3**	Val11Ile	rs2202683	0,219	39	10	25,6	24	0	0,0	0,040	41
**H6548**	chr7	151807411-151807412	C	T	ACG->ATG	**BC040865**	Thr61Met	rs12703159	0,202	77	20	26,0	134	0	0,0	0,000	0
**H6548**	chr17	4616866-4616867	G	T	TTG->TTT	**BC043554**	Leu57Phe		0,000	53	19	35,9	98	0	0,0	0,000	0
**H6548**	chr19	44813446-44813447	T	A	ATG->AAG	**BCAM**	Met204Lys	rs28399656	0,009	486	137	28,2	249	0	0,0	0,000	39

**H6548**	chr22	17727153-17727154	C	T	CCT->TCT	**BCL2L13**	Pro360Ser; Pro198Ser; Pro384Ser;	rs9306198	0,092	77	22	28,6	172	0	0,0	0,000	37
**H6548**	chr16	81268088-81268089	A	T	AGA->AGT	**BCMO1**	Arg267Ser; Arg198Ser	rs12934922	0,352	79	28	35,4	157	0	0,0	0,000	9
**H6548**	chr11	112179333-112179334	C	G	CGG->GGG	**BCO2**	Arg49Gly; Arg15Gly	rs35361223	0,039	110	31	28,2	179	0	0,0	0,000	13
**H6548**	chr14	96204870-96204871	G	-G^GGTGGGGAC		**BDKRB2**	Gly18	rs71103505	0,586	92	27	29,4	121	0	0,0	0,000	15
**H6548**	chr11	61963080-61963081	T	C	TCG->CCG	**BEST1**	Ser271Pro; Ser583Pro	rs17185413	0,172	98	40	40,8	211	0	0,0	0,000	6
**H6548**	chr17	78215996-78215997	A	C	ACC->CCC	**BIRC5**	Thr85Pro; Thr110Pro	rs146071276	0,000	68	19	27,9	87	0	0,0	0,000	84
**H6548**	chr13	102875651-102875652	G	C	GAT->CAT	**BIVM-ERCC5**	Asp1558His	rs17655	0,281	106	34	32,1	156	0	0,0	0,000	0
**H6548**	chr20	6778467-6778468	A	T	AGA->AGT	**BMP2**	Arg190Ser	rs235768	0,678	55	26	47,3	106	0	0,0	0,000	15
**H6548**	chr4	78865701-78865702	G	A	GGT->AGT	**BMP2K**	Gly405Ser	rs2288255	0,062	79	20	25,3	100	0	0,0	0,000	40
**H6548**	chr20	33008665-33008666	A	T	AAG->ATG	**BPIFB2**	Lys31Met	rs6088066	0,247	244	74	30,3	234	0	0,0	0,000	0
**H6548**	chr20	33064485-33064486	G	A	GTG->ATG	**BPIFB3**	Val228Met	rs4911290	0,425	69	18	26,1	212	0	0,0	0,000	0
**H6548**	chr20	33086039-33086040	A	G	ATC->GTC	**BPIFB4**	Ile268Val	rs2070325	0,412	46	12	26,1	110	0	0,0	0,000	0
**H6548**	chr22	32435723-32435724	C	G	GTC->CTC	**BPIFC**	Val302Leu; Val116Leu; Val26Leu	rs5994570	0,083	52	15	28,9	95	1	1,1	0,000	0

**H6548**	chr17	67911161-67911162	A	G	AAG->AGG	**BPTF**	Lys1219Arg; Lys1093Arg	rs34038380	0,014	117	30	25,6	149	0	0,0	0,000	55
**H6548**	chr22	49794204-49794205	C	T	GCT->ACT	**BRD1**	Ala325Thr; Ala779Thr; Ala730Thr	rs35331092	0,067	231	164	71,0	160	0	0,0	0,000	23
**H6548**	chr17	74357605-74357606	G	T	GCG->GAG	**BTBD17**	Ala163Glu	rs11547807	0,142	273	69	25,3	117	0	0,0	0,000	0
**H6548**	chr4	74794306-74794307	A	C	TGC->GGC	**BTC**	Cys7Gly	rs28549760	0,246	139	37	26,6	86	0	0,0	-2,000	33
**H6548**	chr5	181005746-181005747	C	G	CAG->GAG	**BTNL3**	Gln426Glu; Gln211Glu	rs59220426	0,071	72	21	29,2	222	0	0,0	0,000	0
**H6548**	chr5	180950017-180950018	C	A	ACA->AAA	**BTNL8**	Thr326Lys; Thr201Lys; Thr210Lys;	rs7703365	0,181	82	32	39,0	241	0	0,0	0,000	39
**H6548**	chr10	49325417-49325418	T	A	TTC->TAC	**C10orf71**	Phe958Tyr	rs10857470	0,125	150	44	29,3	323	0	0,0	0,000	0
**H6548**	chr12	14823483-14823484	T	-C^TA		**C12orf60**	Thr183	rs139293175	0,159	53	18	34,0	81	0	0,0	0,000	0
**H6548**	chr12	27081980-27081981	G	A	CCG->CTG	**C12orf71**	Pro168Leu	rs61741737	0,199	71	25	35,2	89	0	0,0	0,000	0
**H6548**	chr15	75207653-75207654	T	G	TCA->GCA	**C15orf39**	Ser536Ala; Ser295Ala	rs28509789	0,319	134	42	31,3	227	0	0,0	0,000	0
**H6548**	chr19	50798198-50798199	G	A	CGC->TGC	**C19orf48**	Arg84Cys	rs4802741	0,312	119	32	26,9	226	0	0,0	0,000	0
**H6548**	chr19	13774669-13774670	A	G	AAG->AGG	**C19orf53**	Lys39Arg	rs10104	0,319	203	69	34,0	386	0	0,0	0,000	0
**H6548**	chr19	40742103-40742104	C	T	GTC->ATC	**C19orf54**	Val86Ile	rs3745213	0,133	127	42	33,1	185	0	0,0	0,000	0

**H6548**	chr19	3543398-3543399	C	T	CCG->CTG	**C19orf71**	Pro83Leu	rs12608919	0,344	106	33	31,1	49	0	0,0	0,000	0
**H6548**	chrX	120626773-120626774	A	T	GAT->GAA	**C1GALT1C1**	Asp131Glu	rs17261572	0,195	45	24	53,3	92	0	0,0	0,000	44
**H6548**	chr2	119156898-119156899	C	T	GGG->GAG	**C1QL2**	Gly256Glu		0,000	135	56	41,5	128	0	0,0	0,000	12
**H6548**	chr20	276085-276086	T	A	ATT->TTT	**C20orf96**	Ile305Phe; Ile304Phe; Ile252Phe	rs3827147	0,604	47	16	34,0	139	0	0,0	0,000	0
**H6548**	chr22	31938034-31938035	T	A	CAT->CTT	**C22orf24**	His11Leu	rs1984388	0,169	102	29	28,4	204	0	0,0	0,000	0
**H6548**	chr2	27581564-27581565	T	C	TCC->CCC	**C2orf16**	Ser1665Pro	rs28381983	0,578	73	36	49,3	149	6	4,0	0,000	0
**H6548**	chr4	86887866-86887867	C	G	GTG->CTG	**C4orf36**	Val83Leu	rs72613147	0,175	54	25	46,3	140	0	0,0	0,000	0
**H6548**	chr4	158973158-158973159	A	G	TAC->CAC	**C4orf45**	Tyr73His	rs62351583	0,029	75	19	25,3	162	0	0,0	0,000	0
**H6548**	chr6	42106367-42106368	A	G	CTC->CCC	**C6orf132**	Leu515Pro	rs9394866	0,501	130	34	26,2	237	0	0,0	0,000	0
**H6548**	chr6	31112116-31112117	A	G	GTG->GCG	**C6orf15**	Val81Ala	rs2233977	0,196	95	26	27,4	223	0	0,0	0,000	0
**H6548**	chr6	4122014-4122015	C	A	AAC->AAA	**C6orf201**	Asn117Lys	rs643232	0,322	81	22	27,2	163	0	0,0	0,000	0
**H6548**	chr1	56875053-56875054	C	A	CAA->AAA	**C8A**	Gln93Lys	rs652785	0,367	62	16	25,8	117	0	0,0	0,000	6
**H6548**	chr8	143043191-143043192	T	C	CTG->CCG	**C8orf31**	Leu39Pro	rs11136300	0,672	210	97	46,2	245	0	0,0	0,000	0

**H6548**	chr8	13567595-13567596	G	T	TGT->TTT	**C8orf48**	Cys202Phe	rs752134623	-1,000	76	21	27,6	137	0	0,0	0,000	0
**H6548**	chr8	93134426-93134427	G	T	GCA->GAA	**C8orf87**	Ala58Glu	rs13267247	0,257	65	32	49,2	113	1	0,9	0,000	0
**H6548**	chr9	93335386-93335387	C	T	GGA->AGA	**C9orf129**	Gly118Arg	rs62572859	0,135	328	97	29,6	583	0	0,0	0,000	0
**H6548**	chr9	129613152-129613153	C	T	CGA->CAA	**C9orf50**	Arg381Gln;<br>Arg380Gln	rs2302779	0,191	96	30	31,3	112	0	0,0	0,000	0
**H6548**	chr9	138023326-138023327	G	T	GCG->TCG	**CACNA1B**	Ala463Ser;<br>Ala862Ser;<br>Ala72Ser	rs7873074	0,330	169	49	29,0	64	0	0,0	-2,000	8
**H6548**	chr16	1219094-1219095	C	T	CGC->TGC	**CACNA1H**	Arg2005Cys;<br>Arg1999Cys;<br>Arg700Cys;	rs72552056	0,076	134	40	29,9	99	0	0,0	0,000	14
**H6548**	chr22	39679394-39679395	G	A	GGG->AGG	**CACNA1I**	Gly1782Arg;<br>Gly1747Arg;<br>Gly1697Arg;	rs2294369	0,166	200	53	26,5	63	0	0,0	-2,000	9
**H6548**	chr19	3612820-3612821	G	A	GGT->AGT	**CACTIN-AS1**	Gly183Ser	rs62130601	0,109	317	108	34,1	300	0	0,0	0,000	0
**H6548**	chr12	121274273-121274274	T	A	ACC->TCC	**CAMKK2**	Thr85Ser	rs3817190	0,396	494	145	29,4	267	0	0,0	0,000	42
**H6548**	chr11	65206564-65206565	C	T	CGC->TGC	**CAPN1**	Arg486Cys;<br>Arg227Cys	rs780976578	0,000	282	80	28,4	332	1	0,3	0,000	0
**H6548**	chr1	223545288-223545289	G	A	ACG->ATG	**CAPN8**	Thr592Met	rs61823553	0,490	97	44	45,4	128	0	0,0	0,000	25
**H6548**	chr22	37510301-37510302	C	-G^CTCCTT		**CARD10**	Glu273	rs113275238	0,315	89	26	29,2	123	0	0,0	0,000	56
**H6548**	chr17	80205093-80205094	C	T	CGG->TGG	**CARD14**	Arg820Trp	rs11652075	0,422	137	35	25,6	53	0	0,0	-2,000	23

**H6548**	chr19	48234506-48234507	A	G	TTT->TCT; CTT->CTC	**CARD8**	Phe15Ser; Leu82Leu; Leu32Leu	rs2288876	0,316	50	20	40,0	100	0	0,0	0,000	61
**H6548**	chr15	40621978-40621979	T	C	ATG->ACG	**CASC5**	Met422Thr; Met572Thr; Met598Thr	rs11858113	0,391	92	29	31,5	107	0	0,0	0,000	29
**H6548**	chr11	105008930-105008931	T	A	AAA->AAT	**CASP5**	Lys32Asn; Lys19Asn	rs45483102	0,029	88	24	27,3	132	0	0,0	0,000	29
**H6548**	chr19	38337319-38337320	T	C	CTC->CCC	**CATSPERG**	Leu29Pro	rs2302182	0,170	189	62	32,8	284	0	0,0	0,000	0
**H6548**	chr18	68874768-68874769	G	T	TGT->TTT	**CCDC102B**	Cys346Phe	rs745894	0,177	75	21	28,0	115	0	0,0	0,000	0
**H6548**	chr19	15023113-15023114	C	A	CCC->ACC	**CCDC105**	Pro499Thr	rs8112667	0,338	325	122	37,5	128	0	0,0	0,000	0
**H6548**	chr22	21634309-21634310	G	A	GGA->AGA	**CCDC116**	Gly121Arg	rs371513	0,149	349	113	32,4	297	1	0,3	0,000	0
**H6548**	chr13	43837295-43837296	A	G	ATT->ACT	**CCDC122**	Ile269Thr	rs9567280	0,053	85	25	29,4	146	0	0,0	0,000	0
**H6548**	chr3	42749945-42749946	C	T	GGC->AGC	**CCDC13**	Gly206Ser	rs575899223	0,003	31	9	29,0	90	0	0,0	0,000	0
**H6548**	chr17	81672677-81672678	C	T	CGG->TGG	**CCDC137**	Arg282Trp	rs11546631	0,107	89	26	29,2	72	0	0,0	0,000	0
**H6548**	chr2	158340258-158340259	T	C	ATG->GTG	**CCDC148**	Met157Val; Met71Val; Met104Val	rs12620556	0,127	103	32	31,1	177	0	0,0	0,000	0
**H6548**	chr11	119193197-119193198	C	T	CGC->CAC	**CCDC153**	Arg101His	rs2301574	0,233	43	11	25,6	139	0	0,0	0,000	0
**H6548**	chr16	1434471-1434472	A	G	CCT->CCC; CTG->CCG	**CCDC154**	Pro79Pro; Leu647Pro	rs7192331	0,954	163	81	49,7	117	0	0,0	0,000	0

**H6548**	chr8	143708092-143708093	T	C	AAG->AGG	**CCDC166**	Lys6Arg		0,000	66	22	33,3	25	0	0,0	0,003	0
**H6548**	chr13	102733183-102733184	C	T	CGA->CAA	**CCDC168**	Arg5838Gln	rs17592438	0,159	99	26	26,3	196	0	0,0	0,000	0
**H6548**	chr6	151573204-151573205	C	T	GCT->GTT	**CCDC170**	Ala269Val	rs12205837	0,091	61	24	39,3	85	0	0,0	0,000	61
**H6548**	chr9	15591373-15591374	T	A	TCA->ACA	**CCDC171**	Ser121Thr; Ser28Thr	rs4741510	0,404	62	37	59,7	106	0	0,0	0,000	0
**H6548**	chr2	169654140-169654141	C	T	AGA->AAA	**CCDC173**	Arg298Lys	rs61741435	0,048	83	21	25,3	111	0	0,0	0,000	0
**H6548**	chr9	97343499-97343500	C	G	TCC->TGC	**CCDC180**	Ser856Cys; Ser995Cys; Ser813Cys	rs2061634	0,269	116	35	30,2	214	0	0,0	0,000	0
**H6548**	chr16	3029683-3029684	T	C	CAG->CGG	**CCDC64B**	Gln66Arg; Gln273Arg	rs2244494	0,335	176	49	27,8	127	0	0,0	0,000	0
**H6548**	chr3	56615983-56615984	G	A	GAA->AAA	**CCDC66**	Glu592Lys; Glu228Lys; Glu558Lys	rs4681904	0,037	68	21	30,9	124	1	0,8	0,000	26
**H6548**	chr11	32603000-32603001	A	T	ATA->AAA	**CCDC73**	Ile1017Lys	rs199847743	0,001	55	15	27,3	81	0	0,0	0,000	0
**H6548**	chr2	131533338-131533339	G	A	GAG->AAG	**CCDC74A**	Glu360Lys; Glu294Lys; Glu357Lys	rs140033985	0,012	316	100	31,7	344	1	0,3	0,000	0
**H6548**	chr16	724691-724692	A	G	TGG->CGG	**CCDC78**	Trp252Arg	rs2071950	0,608	258	106	41,1	191	1	0,5	0,000	0
**H6548**	chr2	56193160-56193161	C	T	CGG->TGG	**CCDC85A**	Arg321Trp	rs75743618	0,068	235	76	32,3	205	0	0,0	0,000	0
**H6548**	chr11	64357467-64357468	T	G	TGG->GGG	**CCDC88B**	Trp707Gly; Trp218Gly	rs600377	0,355	169	119	70,4	166	0	0,0	0,000	0

**H6548**	chr19	47271514-47271515	T	C	CTG->CCG	**CCDC9**	Leu478Pro	rs888836	0,501	111	51	46,0	66	0	0,0	-2,000	0
**H6548**	chr12	28450178-28450179	A	G	ATG->GTG	**CCDC91**	Met231Val; Met261Val; Met63Val	rs1133028	0,053	62	16	25,8	79	0	0,0	0,000	0
**H6548**	chr12	123942758-123942759	T	A	AGT->TGT	**CCDC92**	Ser53Cys; Ser70Cys	rs11057401	0,279	76	19	25,0	145	0	0,0	0,000	21
**H6548**	chr2	118014106-118014107	G	-T^GCCGCCGCC		**CCDC93**	Gly19	rs761915740	-1,000	166	62	37,4	90	0	0,0	-2,000	0
**H6548**	chr19	38911814-38911815	C	G	GAG->GAC	**CCER2**	Glu33Asp	rs76973734	0,209	247	80	32,4	164	0	0,0	0,000	0
**H6548**	chr16	57415774-57415775	G	A	GTG->ATG	**CCL17**	Val67Met; Val96Met	rs34379253	0,025	55	14	25,5	94	0	0,0	0,000	30
**H6548**	chr19	43354425-43354426	G	A	GGC->GAC	**CD177**	Gly138Asp	rs77156507	0,138	125	45	36,0	124	0	0,0	0,000	37
**H6548**	chr1	158330049-158330050	C	T	GGA->AGA	**CD1B**	Gly137Arg	rs35841099	0,013	52	15	28,9	83	0	0,0	0,000	18
**H6548**	chr16	30353390-30353391	G	A	ACC->ATC	**CD2BP2**	Thr262Ile	rs34391305	0,011	183	56	30,6	295	0	0,0	0,000	11
**H6548**	chr17	74613949-74613950	C	T	GGG->AGG	**CD300E**	Gly158Arg	rs1878061	0,328	67	17	25,4	164	0	0,0	0,000	22
**H6548**	chr17	74695123-74695124	G	T	ACG->AAG	**CD300LF**	Thr285Lys; Thr282Lys; Thr297Lys	rs34303409	0,050	115	33	28,7	223	0	0,0	0,000	13
**H6548**	chr19	51225384-51225385	A	G	AGG->GGG	**CD33**	Arg69Gly	rs2455069	0,357	75	31	41,3	193	0	0,0	0,000	69
**H6548**	chr11	61008713-61008714	C	T	ACG->ATG	**CD6**	Thr217Met	rs11230562	0,186	767	193	25,2	425	0	0,0	0,000	25

**H6548**	chr3	119537172-119537173	G	A	CAT->TAT	**CD80**	His222Tyr	rs2229098	0,007	77	27	35,1	176	0	0,0	0,000	39
**H6548**	chr11	44618717-44618718	A	G	ATC->GTC	**CD82**	Ile241Val; Ile216Val	rs1139971	0,640	184	97	52,7	213	0	0,0	0,000	72
**H6548**	chr1	20589207-20589208	A	C	AAG->CAG	**CDA**	Lys27Gln	rs2072671	0,272	51	16	31,4	134	0	0,0	0,000	22
**H6548**	chr22	37568406-37568407	C	A	CCT->ACT	**CDC42EP1**	Pro255Thr	rs77417880	0,067	24	7	29,2	27	0	0,0	0,019	19
**H6548**	chr8	25507317-25507318	A	T	AGA->AGT	**CDCA2**	Arg884Ser; Arg869Ser; Arg547Ser	rs3829009	0,204	78	23	29,5	141	0	0,0	0,000	65
**H6548**	chr16	64991814-64991815	G	A	ACG->ATG	**CDH11**	Thr255Met; Thr129Met	rs35195	0,269	123	57	46,3	215	0	0,0	0,000	52
**H6548**	chr16	89179481-89179482	G	T	GCG->TCG	**CDH15**	Ala37Ser	rs2287359	0,115	211	61	28,9	185	0	0,0	0,000	27
**H6548**	chr7	105974959-105974960	G	A	GTG->ATG	**CDHR3**	Val55Met; Val42Met	rs35008315	0,187	59	16	27,1	89	0	0,0	0,000	10
**H6548**	chr17	47981704-47981705	T	C	TCA->CCA	**CDK5RAP3**	Ser308Pro	rs2737	0,244	66	17	25,8	135	0	0,0	0,000	63
**H6548**	chr6	21065217-21065218	C	T	CCA->CTA	**CDKAL1**	Pro409Leu; Pro339Leu; Pro5Leu	rs77152992	0,066	152	45	29,6	245	0	0,0	0,000	11
**H6548**	chr12	12718164-12718165	T	G	GTC->GGC	**CDKN1B**	Val109Gly	rs2066827	0,278	196	61	31,1	173	0	0,0	0,000	77
**H6548**	chr6	4715781-4715782	A	G	ACA->GCA	**CDYL**	Thr2Ala	rs3812179	0,214	73	25	34,3	133	0	0,0	0,000	19
**H6548**	chr19	44703408-44703409	A	C	GAA->GCA	**CEACAM16**	Glu33Ala		0,000	163	42	25,8	174	0	0,0	0,000	0

**H6548**	chr19	44524078-44524079	T	C	ATT->GTT	**CEACAM20**	Ile127Val	rs35443082	0,112	111	41	36,9	225	0	0,0	0,000	50
**H6548**	chr19	41761980-41761981	T	G	GTC->GGC	**CEACAM6**	Val239Gly	rs11548735	0,539	143	67	46,9	264	0	0,0	0,000	67
**H6548**	chr14	23118628-23118629	G	T	CTG->ATG	**CEBPE**	Leu155Met	rs141903485	0,007	387	120	31,0	228	1	0,4	0,000	56
**H6548**	chr2	37201783-37201784	T	-G^TTT		**CEBPZ**	Thr1049	rs148813970	0,053	43	11	25,6	88	0	0,0	0,000	0
**H6548**	chr22	17188415-17188416	T	C	CAT->CGT	**CECR1**	His94Arg; His215Arg; His335Arg;	rs2231495	0,348	90	25	27,8	191	0	0,0	0,000	26
**H6548**	chr1	15482376-15482377	G	A	GAC->AAC	**CELA2B**	Asp114Asn	rs3766160	0,273	93	25	26,9	268	0	0,0	0,000	0
**H6548**	chr1	214640438-214640439	A	G	ATG->GTG	**CENPF**	Met701Val	rs3795524	0,082	69	18	26,1	126	0	0,0	0,000	68
**H6548**	chr14	80784910-80784911	T	C	CAT->CGT	**CEP128**	His732Arg; His759Arg	rs327463	0,383	94	53	56,4	186	0	0,0	0,000	0
**H6548**	chr17	81196777-81196778	C	T	GCG->ACG	**CEP131**	Ala38Thr; Ala608Thr; Ala611Thr	rs4613097	0,089	276	73	26,5	116	0	0,0	0,000	44
**H6548**	chr11	117363500-117363501	T	C	TAT->CAT	**CEP164**	Tyr254His	rs750854769	0,000	56	15	26,8	143	0	0,0	0,000	38
**H6548**	chr18	13100451-13100452	A	G	AAA->GAA	**CEP192**	Lys2271Glu; Lys1796Glu; Lys693Glu;	rs3737379	0,048	75	25	33,3	143	0	0,0	0,000	49
**H6548**	chr10	93519748-93519749	A	T	CAT->CTT	**CEP55**	His378Leu	rs2293277	0,698	76	31	40,8	115	0	0,0	0,000	68
**H6548**	chr12	94375992-94375993	G	A	CGG->TGG	**CEP83**	Arg276Trp	rs74340001	0,052	49	14	28,6	67	0	0,0	0,000	0

**H6548**	chr19	32879177-32879178	T	A	CAT->CTT	**CEP89**	His532Leu; His779Leu	rs745960	0,262	48	14	29,2	88	0	0,0	0,000	0
**H6548**	chr2	181603867-181603868	T	C	ATA->ATG	**CERKL**	Ile150Met	rs780561754	0,000	77	30	39,0	134	0	0,0	0,000	9
**H6548**	chr19	8261981-8261982	C	T	GCG->GTG	**CERS4**	Ala353Val; Ala189Val; Ala265Val	rs17160348	0,159	155	40	25,8	126	1	0,8	0,000	38
**H6548**	chr1	196959057-196959058	A	G	TAT->TGT	**CFHR2**	Tyr264Cys; Tyr140Cys	rs41310132	0,014	104	27	26,0	155	0	0,0	0,000	0
**H6548**	chr3	126733093-126733094	G	T	GCT->TCT	**CHCHD6**	Ala95Ser	rs2272487	0,220	86	41	47,7	198	0	0,0	0,000	0
**H6548**	chr1	147242776-147242777	G	C	CGA->CCA	**CHD1L**	Arg25Pro	rs11588753	0,188	223	64	28,7	123	0	0,0	0,000	70
**H6548**	chr16	53324526-53324527	A	G	ACA->GCA	**CHD9**	Thr2760Ala; Thr2776Ala; Thr2761Ala;	rs3743771	0,256	71	35	49,3	100	0	0,0	0,000	39
**H6548**	chr1	111312272-111312273	G	A	GAC->AAC	**CHIA**	Asp47Asn	rs41282494	0,107	114	29	25,4	133	0	0,0	0,000	20
**H6548**	chr11	870445-870446	G	A	GCC->GTC	**CHID1**	Ala399Val; Ala363Val; Ala338Val;	rs6682	0,548	40	18	45,0	53	0	0,0	0,000	17
**H6548**	chr3	319809-319810	G	A	GTA->ATA	**CHL1**	Val12Ile	rs142251617	0,018	62	17	27,4	78	0	0,0	0,000	19
**H6548**	chr16	75529431-75529432	G	A	ACG->ATG	**CHST5**	Thr318Met; Thr324Met	rs3826107	0,167	618	320	51,8	480	0	0,0	0,000	24
**H6548**	chr15	101178033-101178034	C	G	AGA->ACA	**CHSY1**	Arg316Thr; Arg588Thr	rs62621400	0,063	137	36	26,3	157	0	0,0	0,000	20
**H6548**	chr16	10906990-10906991	G	C	GGC->GCC	**CIITA**	Gly500Ala; Gly452Ala; Gly501Ala	rs4774	0,292	465	136	29,3	290	0	0,0	0,000	41

**H6548**	chr12	106239790-106239791	C	T	GCC->ACC	**CKAP4**	Ala348Thr	rs3088113	0,256	176	47	26,7	152	0	0,0	0,000	74
**H6548**	chr22	19524401-19524402	G	A	CAG->TAG	**CLDN5**	Gln37*	rs885985	0,537	237	137	57,8	107	0	0,0	0,000	26
**H6548**	chr16	3015594-3015595	T	C	ATC->GTC	**CLDN6**	Ile143Val	rs2257295	0,359	199	69	34,7	278	0	0,0	0,000	47
**H6548**	chr12	9680927-9680928	C	G	CTG->GTG	**CLEC2D**	Leu23Val	rs3764022	0,319	54	34	63,0	123	0	0,0	0,000	35
**H6548**	chr3	45035630-45035631	G	A	GGC->AGC	**CLEC3B**	Gly106Ser	rs13963	0,488	405	117	28,9	282	0	0,0	0,000	35
**H6548**	chr2	70816328-70816329	C	T	CGT->CAT	**CLEC4F**	Arg351His	rs722896	0,332	84	24	28,6	148	0	0,0	0,000	11
**H6548**	chr12	9733111-9733112	T	+TAAGT		**CLECL1**	Leu51	rs71045297	0,507	179	47	26,3	204	0	0,0	0,000	9
**H6548**	chr2	55209797-55209798	G	A	GCT->GTT	**CLHC1**	Ala178Val; Ala56Val	rs9677948	0,208	67	21	31,3	133	0	0,0	0,000	0
**H6548**	chr2	29160389-29160390	G	T	CGC->CTC	**CLIP4**	Arg486Leu	rs3100246	0,096	82	24	29,3	159	2	1,3	0,000	39
**H6548**	chr12	92424705-92424706	C	T	TCC->TTC	**CLLU1**	Ser9Phe	rs12580153	0,235	69	19	27,5	129	0	0,0	0,000	78
**H6548**	chr4	10584946-10584947	G	A	CCT->CTT	**CLNK**	Pro31Leu	rs61759824	0,043	105	32	30,5	171	0	0,0	0,000	25
**H6548**	chr3	100167817-100167818	G	A	GTC->ATC	**CMSS1**	Val166Ile; Val148Ile	rs11537816	0,169	72	20	27,8	129	0	0,0	0,000	0
**H6548**	chr16	71284673-71284674	T	C	AAT->AGT	**CMTR2**	Asn416Ser	rs3803704	0,280	67	25	37,3	141	0	0,0	0,000	0

**H6548**	chr18	74508847-74508848	T	C	TAT->CAT	**CNDP2**	Tyr126His	rs2278161	0,226	95	35	36,8	168	0	0,0	0,000	19
**H6548**	chr4	47943277-47943278	C	T	GAT->AAT	**CNGA1**	Asp187Asn; Asp118Asn	rs28642966	0,180	49	15	30,6	104	0	0,0	0,000	0
**H6548**	chr3	32727696-32727697	A	T	ACG->TCG	**CNOT10**	Thr120Ser; Thr348Ser; Thr347Ser;	rs11558687	0,021	44	15	34,1	112	0	0,0	0,000	0
**H6548**	chr11	99819554-99819555	T	G	TCT->GCT	**CNTN5**	Ser23Ala	rs10790978	0,497	49	20	40,8	60	0	0,0	0,000	9
**H6548**	chr7	147121153-147121154	G	C	TTG->TTC	**CNTNAP2**	Leu310Phe		0,000	71	23	32,4	107	0	0,0	0,000	14
**H6548**	chr9	41991720-41991721	G	A	CTT->TTT	**CNTNAP3B**	Leu408Phe	rs3739623	0,341	152	47	30,9	396	0	0,0	0,000	0
**H6548**	chr2	164842036-164842037	G	A	CCG->CTG	**COBLL1**	Pro3Leu	rs2279982	0,214	115	33	28,7	132	0	0,0	0,000	36
**H6548**	chr13	39655819-39655820	T	A	TGC->AGC	**COG6**	Cys32Ser	rs3812883	0,512	160	49	30,6	107	0	0,0	0,000	16
**H6548**	chr1	102879758-102879759	C	T	CGC->CAC	**COL11A1**	Arg929His; Arg1733His; Arg1745His;	rs140250347	0,000	90	31	34,4	225	0	0,0	0,000	28
**H6548**	chr6	33185750-33185751	C	T	GAG->AAG	**COL11A2**	Glu276Lys	rs9277934	0,304	47	12	25,5	52	0	0,0	0,010	33
**H6548**	chr9	98986073-98986074	A	G	ATG->GTG	**COL15A1**	Met204Val	rs2075663	0,476	55	15	27,3	121	0	0,0	0,000	21
**H6548**	chr6	56064600-56064601	T	C	GAA->GAG; AGA->GGA	**COL21A1**	Glu74Glu; Arg102Gly; Arg90Gly;		0,000	111	33	29,7	153	0	0,0	0,000	27
**H6548**	chr7	101557446-101557447	G	A	GCC->ACC	**COL26A1**	Ala415Thr; Ala413Thr	rs17393069	0,375	67	25	37,3	151	0	0,0	0,000	0

**H6548**	chr9	114267533-114267534	C	T	CTT->TTT	**COL27A1**	Leu1160Phe	rs373214163	0,000	105	37	35,2	192	0	0,0	0,000	4
**H6548**	chr2	227298736-227298737	C	A	GAC->GAA	**COL4A3**	Asp1269Glu	rs57611801	0,044	65	20	30,8	130	0	0,0	0,000	8
**H6548**	chr9	134750807-134750808	G	A	GGC->AGC	**COL5A1**	Gly530Ser	rs61735045	0,035	199	73	36,7	281	0	0,0	0,000	10
**H6548**	chr19	9977594-9977595	C	G	CGG->CCG	**COL5A3**	Arg1042Pro	rs2161468	0,379	98	29	29,6	177	0	0,0	0,000	24
**H6548**	chr2	237381336-237381337	G	C	ACT->AGT	**COL6A3**	Thr492Ser; Thr286Ser; Thr85Ser	rs113897824	0,007	96	24	25,0	157	0	0,0	0,000	22
**H6548**	chr3	15175195-15175196	A	T	TAT->AAT	**COL6A4P1**	Tyr278Asn	rs11718863	0,229	112	30	26,8	193	0	0,0	0,000	0
**H6548**	chr3	130471871-130471872	G	A	GCA->ACA	**COL6A5**	Ala2589Thr	rs73868680	0,157	145	51	35,2	201	0	0,0	0,000	4
**H6548**	chr1	184037292-184037293	C	A	CGC->CTC	**COLGALT2**	Arg22Leu	rs114087663	0,006	157	44	28,0	97	0	0,0	0,000	0
**H6548**	chr3	149750742-149750743	T	G	ATT->CTT	**COMMD2**	Ile113Leu	rs9843784	0,349	54	22	40,7	81	0	0,0	0,000	0
**H6548**	chr19	17011498-17011499	T	C	ACC->GCC	**CPAMD8**	Thr198Ala	rs200785414	0,001	155	51	32,9	224	0	0,0	0,000	0
**H6548**	chr10	92240045-92240046	G	A	TCG->TTG	**CPEB3**	Ser102Leu		0,000	461	158	34,3	341	0	0,0	0,000	14
**H6548**	chr3	9704753-9704754	G	C	GTG->CTG	**CPNE9**	Val39Leu	rs201517941	0,004	120	50	41,7	85	0	0,0	-2,000	0
**H6548**	chr16	12665108-12665109	T	C	AAA->AGA	**CPPED1**	Lys241Arg; Lys99Arg	rs1713480	0,850	65	22	33,9	161	0	0,0	0,000	0

**H6548**	chr8	96835133-96835134	G	C	GCT->CCT	**CPQ**	Ala199Pro		0,000	60	15	25,0	96	0	0,0	0,000	11
**H6548**	chr7	29120987-29120988	C	T	CGC->CAC	**CPVL**	Arg25His	rs34219043	0,148	46	14	30,4	88	0	0,0	0,000	8
**H6548**	chr1	207552815-207552816	G	T	CAG->CAT	**CR1**	Gln572His; Gln1022His	rs200082366	0,156	24	9	37,5	50	0	0,0	0,000	32
**H6548**	chr1	207708211-207708212	A	G	ATC->GTC	**CR1L**	Ile455Val	rs6683902	0,612	77	44	57,1	181	0	0,0	0,000	4
**H6548**	chr1	207473116-207473117	G	A	AGT->AAT	**CR2**	Ser124Asn; Ser639Asn	rs17615	0,270	44	11	25,0	111	0	0,0	0,000	36
**H6548**	chr14	105488367-105488368	C	T	GCA->GTA	**CRIP1**	Ala58Val	rs55633823	0,174	117	31	26,5	101	1	1,0	0,000	40
**H6548**	chr4	5887389-5887390	T	A	CAT->CTT	**CRMP1**	His7Leu	rs28550375	0,410	103	80	77,7	102	0	0,0	0,000	34
**H6548**	chr20	20052722-20052723	C	G	GGG->CGG	**CRNKL1**	Gly35Arg	rs7508949	0,386	169	82	48,5	151	0	0,0	0,000	0
**H6548**	chr10	97865561-97865562	C	T	GAG->AAG	**CRTAC1**	Glu658Lys	rs56007204	0,149	215	82	38,1	143	0	0,0	0,000	11
**H6548**	chr19	18765447-18765448	G	A	GTC->ATC	**CRTC1**	Val327Ile; Val311Ile; Val176Ile	rs36070283	0,068	60	15	25,0	118	0	0,0	0,000	50
**H6548**	chr11	111910322-111910323	A	C	TGT->GGT	**CRYAB**	Cys110Gly	rs11603779	0,268	52	14	26,9	127	0	0,0	0,000	20
**H6548**	chr2	218993157-218993158	G	A	CCG->TCG	**CRYBA2**	Pro7Ser	rs141631259	0,025	190	54	28,4	79	0	0,0	-2,000	4
**H6548**	chr21	33624711-33624712	C	T	GCT->ACT	**CRYZL1**	Ala39Thr; Ala63Thr	rs13050238	0,017	56	17	30,4	97	0	0,0	0,000	0

**H6548**	chr10	43183347-43183348	C	T	CCT->TCT	**CSGALNACT2**	Pro479Ser	rs2435381	0,229	136	43	31,6	251	0	0,0	0,000	26
**H6548**	chr8	3493717-3493718	C	G	AAG->AAC	**CSMD1**	Lys451Asn	rs201131145	0,000	135	34	25,2	210	0	0,0	0,000	34
**H6548**	chr1	33572613-33572614	T	C	ATG->GTG	**CSMD2**	Met2552Val; Met2554Val	rs2641962	0,500	70	20	28,6	110	0	0,0	0,000	31
**H6548**	chr6	31669956-31669957	A	T	GAC->GTC	**CSNK2B**	Asp89Val; Asp113Val	rs5872	0,325	98	29	29,6	128	0	0,0	0,000	23
**H6548**	chr15	75676993-75676994	C	T	CGG->CAG	**CSPG4**	Arg1842Gln	rs77237106	0,125	134	42	31,3	108	0	0,0	0,000	65
**H6548**	chr8	67161901-67161902	G	A	CGT->CAT	**CSPP1**	Arg872His; Arg907His; Arg527His	rs16933182	0,078	86	25	29,1	100	0	0,0	0,000	38
**H6548**	chr20	23440463-23440464	G	A	AGG->AAG	**CSTL1**	Arg66Lys	rs17757442	0,098	104	27	26,0	220	0	0,0	0,000	0
**H6548**	chrX	154653250-154653251	C	G	GAG->CAG	**CTAG2**	Glu89Gln	rs17328091	0,561	176	85	48,3	103	0	0,0	0,000	80
**H6548**	chr18	22415767-22415768	T	C	ATA->GTA	**CTAGE1**	Ile682Val	rs9946136	0,205	137	37	27,0	216	0	0,0	0,000	76
**H6548**	chr13	49892774-49892775	G	A	GCT->ACT	**CTAGE10P**	Ala729Thr	rs34448316	0,169	144	38	26,4	229	0	0,0	0,000	0
**H6548**	chr6	131709419-131709420	G	A	ACG->ATG	**CTAGE9**	Thr533Met	rs200750273	0,305	68	17	25,0	165	0	0,0	0,000	0
**H6548**	chr1	84574386-84574387	C	A	CGC->CTC	**CTBS**	Arg10Leu	rs115347546	0,311	115	54	47,0	48	0	0,0	0,000	4
**H6548**	chr9	108965389-108965390	C	G	GAA->CAA	**CTNNAL1**	Glu179Gln; Glu527Gln	rs7021366	0,258	70	19	27,1	128	0	0,0	0,000	36

**H6548**	chr17	3660668-3660669	C	G	CCA->GCA	**CTNS**	Pro380Ala; Pro272Ala	rs2873624	0,251	201	73	36,3	150	0	0,0	0,000	5
**H6548**	chr20	45891598-45891599	C	-C^CTG		**CTSA**	Leu29; Leu11	rs3080212	0,648	147	53	36,1	94	0	0,0	0,000	10
**H6548**	chr1	150755062-150755063	G	A	CGG->TGG	**CTSS**	Arg113Trp	rs2230061	0,377	139	48	34,5	205	0	0,0	0,000	17
**H6548**	chr16	88715205-88715206	G	C	CGG->CGC;	**CTU2**	Arg501Arg;	rs1058158	0,256	260	89	34,2	212	0	0,0	0,000	7
**H6548**	chr6	43046737-43046738	C	A	TGC->TTC	**CUL7**	Cys33Phe	rs73733790	0,039	151	44	29,1	295	0	0,0	0,000	38
**H6548**	chr6	33418175-33418176	A	G	TCG->CCG	**CUTA**	Ser4Pro	rs1705003	0,150	153	41	26,8	260	0	0,0	0,000	10
**H6548**	chr2	179950684-179950685	G	A	GCG->GTG	**CWC22**	Ala178Val; Ala656Val	rs17778270	0,024	54	20	37,0	100	0	0,0	0,000	8
**H6548**	chr17	38802748-38802749	G	A	CGG->TGG	**CWC25**	Arg309Trp; Arg372Trp	rs139189793	0,009	87	23	26,4	137	0	0,0	0,000	0
**H6548**	chr2	218060695-218060696	A	T	ATA->AAA	**CXCR2P1**	Ile101Lys	rs3181379	0,014	118	33	28,0	352	0	0,0	0,000	0
**H6548**	chrX	45191865-45191866	C	T	AGA->AAA	**CXorf36**	Arg128Lys	rs1132201	0,412	52	37	71,2	87	0	0,0	0,000	0
**H6548**	chr2	38070995-38070996	T	C	AAC->AGC	**CYP1B1**	Asn453Ser	rs1800440	0,153	91	26	28,6	159	0	0,0	0,000	47
**H6548**	chr19	15650070-15650071	C	A	GCC->GAC	**CYP4F3**	Ala269Asp; Ala120Asp	rs1805040	0,134	138	59	42,8	243	0	0,0	0,000	21
**H6548**	chr19	15615748-15615749	G	T	GGC->TGC; GGC->TGC	**CYP4F8**	Gly45Cys; Thr6Thr	rs73001907	0,168	190	49	25,8	190	0	0,0	0,000	13

**H6548**	chr4	186191886-186191887	C	G	CTT->GTT	**CYP4V2**	Leu22Val	rs1055138	0,521	244	113	46,3	125	0	0,0	0,000	5
**H6548**	chr2	241751259-241751260	G	A	GTC->ATC	**D2HGDH**	Val338Ile; Val204Ile; Val37Ile	rs1106639	0,248	118	35	29,7	156	1	0,6	0,000	36
**H6548**	chr9	133657151-133657152	C	T	CGC->TGC	**DBH**	Arg549Cys	rs6271	0,044	154	53	34,4	167	0	0,0	0,000	6
**H6548**	chr2	119367481-119367482	C	T	GCC->GTC	**DBI**	Ala30Val; Ala49Val	rs3731607	0,197	298	93	31,2	260	0	0,0	0,000	6
**H6548**	chr1	168044965-168044966	T	A	CTT->CAT	**DCAF6**	Leu666His; Leu635His; Leu609His;	rs41271647	0,069	62	23	37,1	92	0	0,0	0,000	35
**H6548**	chr11	6627192-6627193	G	A	ACG->ATG	**DCHS1**	Thr1949Met	rs4758443	0,354	137	37	27,0	266	0	0,0	0,000	26
**H6548**	chr4	154320582-154320583	C	T	GCA->ACA	**DCHS2**	Ala1151Thr	rs79535970	0,037	94	24	25,5	160	0	0,0	0,000	14
**H6548**	chr10	14932905-14932906	T	C	CAT->CGT	**DCLRE1C**	His243Arg; His123Arg; His128Arg	rs12768894	0,155	87	22	25,3	183	0	0,0	0,000	42
**H6548**	chr14	53058493-53058494	G	A	CAT->TAT	**DDHD1**	His55Tyr; His241Tyr; His659Tyr;		0,000	33	10	30,3	58	0	0,0	0,002	12
**H6548**	chr1	13417-13418	G	+GAGA		**DDX11L1**	Glu61	rs777038595	0,174	50	17	34,0	68	0	0,0	0,000	0
**H6548**	chr12	9419126-9419127	C	T	GTC->ATC	**DDX12P**	Val63Ile	rs573319575	0,003	25	7	28,0	58	0	0,0	0,007	0
**H6548**	chr1	111766708-111766709	T	C	ATA->ACA	**DDX20**	Ile762Thr; Ile524Thr; Ile370Thr	rs85276	0,129	96	29	30,2	153	0	0,0	0,000	32
**H6548**	chr12	123613218-123613219	A	G	AAT->AGT	**DDX55**	Asn117Ser; Asn264Ser	rs11057306	0,120	70	22	31,4	158	0	0,0	0,000	0

**H6548**	chr4	168276145-168276146	C	T	GTG->ATG	**DDX60**	Val672Met	rs550625	0,091	51	15	29,4	86	0	0,0	0,000	32
**H6548**	chr8	7836511-7836512	A	G	ATT->GTT	**DEFB104A**	Ile10Val	rs2680507	0,450	20	6	30,0	71	0	0,0	0,007	0
**H6548**	chr20	158934-158935	C	A	CGT->AGT	**DEFB127**	Arg71Ser	rs16995685	0,359	116	68	58,6	178	0	0,0	0,000	0
**H6548**	chr1	197511855-197511856	C	T	GGT->AGT	**DENND1B**	Gly563Ser	rs10494755	0,063	72	19	26,4	152	0	0,0	0,000	23
**H6548**	chr12	31495891-31495892	C	T	AGA->AAA	**DENND5B**	Arg87Lys; Arg52Lys; Arg74Lys	rs4930979	0,453	89	28	31,5	115	0	0,0	0,000	0
**H6548**	chr8	120049639-120049640	G	A	AGC->AAC	**DEPTOR**	Ser389Asn; Ser288Asn	rs4871827	0,319	120	31	25,8	203	0	0,0	0,000	53
**H6548**	chr22	20086482-20086483	A	G	ATA->GTA	**DGCR8**	Ile174Val	rs35987994	0,054	83	21	25,3	123	0	0,0	0,000	30
**H6548**	chr17	56834969-56834970	A	G	AAG->GAG	**DGKE**	Lys59Glu		0,000	260	85	32,7	289	0	0,0	0,000	11
**H6548**	chr3	186288805-186288806	G	A	CGG->TGG	**DGKG**	Arg150Trp	rs137931929	0,000	111	29	26,1	170	0	0,0	0,000	18
**H6548**	chr1	26460135-26460136	G	A	GTG->ATG	**DHDDS**	Val253Met; Val214Met; Val219Met;	rs3816539	0,319	67	18	26,9	96	0	0,0	0,000	4
**H6548**	chr5	80654583-80654584	C	T	TGG->TAG	**DHFR**	Trp127*	rs10168	0,265	51	22	43,1	49	0	0,0	0,000	25
**H6548**	chr13	51791217-51791218	G	T	CAA->AAA	**DHRS12**	Gln105Lys; Gln56Lys	rs61729904	0,092	45	13	28,9	78	0	0,0	0,000	0
**H6548**	chr20	62905585-62905586	G	A	TCG->TTG	**DIDO1**	Ser535Leu	rs2295000	0,204	49	19	38,8	74	0	0,0	0,000	56

**H6548**	chr14	80203236-80203237	T	C	ACA->GCA	**DIO2**	Thr92Ala	rs225014	0,437	82	21	25,6	137	0	0,0	0,000	8
**H6548**	chr13	72775220-72775221	G	C	ACA->AGA	**DIS3**	Thr296Arg; Thr326Arg	rs7332388	0,402	78	37	47,4	108	0	0,0	0,000	32
**H6548**	chr1	231694548-231694549	G	A	CGG->CAG	**DISC1**	Arg219Gln; Arg264Gln; Arg253Gln	rs3738401	0,290	195	99	50,8	248	0	0,0	0,000	7
**H6548**	chr15	40367669-40367670	G	A	GTG->ATG	**DISP2**	Val520Met	rs138998520	0,003	186	64	34,4	203	0	0,0	0,000	18
**H6548**	chr19	49364655-49364656	G	A	GCT->ACT	**DKKL1**	Ala29Thr	rs919364	0,268	74	41	55,4	103	0	0,0	0,000	26
**H6548**	chr19	39504070-39504071	T	C	CTG->CCG	**DLL3**	Leu218Pro	rs1110627	0,571	68	30	44,1	61	0	0,0	0,000	21
**H6548**	chr17	49969598-49969599	A	G	AAT->AGT	**DLX4**	Asn44Ser	rs61749026	0,046	237	95	40,1	289	0	0,0	0,000	46
**H6548**	chrX	31478232-31478233	C	T	CGG->CAG	**DMD**	Arg208Gln; Arg477Gln; Arg1593Gln;	rs1800280	0,904	82	43	52,4	111	0	0,0	0,000	4
**H6548**	chr3	52383974-52383975	G	A	GTG->ATG	**DNAH1**	Val2756Met	rs61749019	0,008	105	30	28,6	128	0	0,0	0,000	5
**H6548**	chr12	123789926-123789927	A	G	ATT->GTT	**DNAH10**	Ile480Val	rs10846559	0,734	67	34	50,8	161	0	0,0	0,000	28
**H6548**	chr3	57446217-57446218	T	C	AAT->AGT	**DNAH12**	Asn1308Ser	rs62622492	0,065	74	22	29,7	106	0	0,0	0,000	0
**H6548**	chr1	225265280-225265281	A	G	AAT->AGT	**DNAH14**	Asn2435Ser	rs76767146	0,051	113	35	31,0	131	0	0,0	0,000	0
**H6548**	chr5	13902067-13902068	A	C	TTG->TGG	**DNAH5**	Leu572Trp	rs137878131	0,001	58	20	34,5	56	0	0,0	0,000	8

**H6548**	chr2	195957279-195957280	A	G	ATA->ACA	**DNAH7**	Ile1020Thr	rs62623378	0,089	57	27	47,4	145	2	1,4	0,000	11
**H6548**	chr6	38805516-38805517	G	A	GGA->GAA	**DNAH8**	Gly1024Glu; Gly807Glu	rs874808	0,459	81	23	28,4	116	0	0,0	0,000	0
**H6548**	chr9	34500822-34500823	G	A	GTC->ATC	**DNAI1**	Val335Ile; Val339Ile	rs11793196	0,157	80	20	25,0	178	0	0,0	0,000	1
**H6548**	chr7	73683323-73683324	C	T	GGA->AGA	**DNAJC30**	Gly34Arg	rs1128349	0,382	190	74	39,0	258	0	0,0	0,000	0
**H6548**	chr11	6498411-6498412	G	A	CGA->CAA	**DNHD1**	Arg66Gln	rs11604149	0,458	82	40	48,8	166	0	0,0	0,000	0
**H6548**	chr12	32707367-32707368	G	A	GGG->GGA; GGG->GGA	**DNM1L**	Gly97Gly; Gly84Gly; Gly29Glu	rs2272238	0,133	34	9	26,5	65	0	0,0	0,003	25
**H6548**	chr2	224806183-224806184	G	C	ATC->ATG	**DOCK10**	Ile115Met; Ile1246Met; Ile1252Met	rs4674941	0,169	97	53	54,6	229	0	0,0	0,000	47
**H6548**	chr9	334336-334337	A	G	AAT->AGT	**DOCK8**	Asn413Ser; Asn345Ser	rs10970979	0,247	44	14	31,8	78	0	0,0	0,000	33
**H6548**	chr5	177504592-177504593	G	C	CCC->CGC	**DOK3**	Pro294Arg	rs61749657	0,071	368	105	28,5	129	0	0,0	0,000	33
**H6548**	chr6	83129132-83129133	G	A	GTA->ATA	**DOPEY1**	Val647Ile; Val656Ile	rs147987838	0,002	135	39	28,9	179	0	0,0	0,000	0
**H6548**	chr11	119097047-119097048	T	C	ATC->GTC	**DPAGT1**	Ile393Val; Ile286Val; Ile312Val;	rs643788	0,422	89	29	32,6	149	0	0,0	0,000	23
**H6548**	chr6	30953112-30953113	G	A	CGG->CAG	**DPCR1**	Arg1393Gln	rs2240804	0,294	64	20	31,3	197	0	0,0	0,000	45
**H6548**	chr17	2036937-2036938	A	G	AAA->AGA	**DPH1**	Lys226Arg; Lys210Arg; Lys86Arg;	rs1131600	0,152	95	34	35,8	171	0	0,0	0,000	42

**H6548**	chr2	115746094-115746095	G	A	GTG->ATG	**DPP10**	Val288Met; Val238Met; Val284Met;	rs36044503	0,084	97	34	35,1	170	0	0,0	0,000	10
**H6548**	chr12	63668242-63668243	A	C	TCC->GCC	**DPY19L2**	Ser51Ala	rs10878073	0,293	161	49	30,4	269	0	0,0	0,000	7
**H6548**	chr7	103210278-103210279	T	A	AAC->ATC	**DPY19L2P2**	Asn333Ile	rs17136078	0,176	38	14	36,8	99	0	0,0	0,000	0
**H6548**	chr18	31546144-31546145	T	G	GTA->GGA	**DSG2**	Val920Gly	rs142841727	0,003	113	32	28,3	198	0	0,0	0,000	35
**H6548**	chr6	56508737-56508738	C	T	GAC->AAC	**DST**	Asp3721Asn; Asp4047Asn; Asp4087Asn;	rs41271862	0,039	105	27	25,7	246	0	0,0	0,000	7
**H6548**	chr10	75043808-75043809	T	G	AGC->CGC	**DUPD1**	Ser137Arg	rs16931938	0,152	181	53	29,3	127	0	0,0	0,000	0
**H6548**	chr10	75108140-75108141	T	C	AAG->AGG	**DUSP13**	Lys73Arg	rs7912300	0,355	59	24	40,7	79	0	0,0	0,000	28
**H6548**	chr20	31861521-31861522	C	G	GTG->CTG	**DUSP15**	Val94Leu; Val197Leu	rs947310	0,346	289	100	34,6	98	0	0,0	0,000	0
**H6548**	chr1	167127744-167127745	A	G	AAG->GAG	**DUSP27**	Lys872Glu	rs138472448	0,003	70	20	28,6	122	2	1,6	0,000	32
**H6548**	chr2	206663115-206663116	G	T	CAG->AAG	**DYTN**	Gln474Lys	rs2115591	0,365	107	52	48,6	225	0	0,0	0,000	0
**H6548**	chr3	138088416-138088417	G	A	CGG->TGG	**DZIP1L**	Arg321Trp	rs2724693	0,635	74	19	25,7	94	0	0,0	0,000	24
**H6548**	chr10	22209538-22209539	C	T	GGA->AGA	**EBLN1**	Gly149Arg	rs838759	0,290	124	32	25,8	235	0	0,0	0,000	0
**H6548**	chr6	4116027-4116028	G	A	GCC->GTC	**ECI2**	Ala314Val; Ala172Val; Ala344Val;	rs7166	0,302	35	10	28,6	76	0	0,0	0,000	14

**H6548**	chr9	92522699-92522700	T	G	CAG->CCG	**ECM2**	Gln56Pro	rs10120210	0,626	74	42	56,8	122	0	0,0	0,000	18
**H6548**	chr1	184694402-184694403	A	C	ATT->AGT	**EDEM3**	Ile820Ser; Ile836Ser	rs9425343	0,390	79	29	36,7	114	0	0,0	0,000	26
**H6548**	chr6	12296021-12296022	G	T	AAG->AAT	**EDN1**	Lys197Asn; Lys198Asn	rs5370	0,235	109	28	25,7	164	0	0,0	0,000	16
**H6548**	chr8	143589009-143589010	C	T	GTG->ATG	**EEF1D**	Val358Met; Val408Met	rs746820089	0,000	170	47	27,7	77	0	0,0	-2,000	32
**H6548**	chr17	47370465-47370466	A	G	ATA->GTA	**EFCAB13**	Ile35Val; Ile183Val; Ile279Val;	rs55853213	0,061	53	18	34,0	114	0	0,0	0,000	0
**H6548**	chr8	132003280-132003281	A	G	ATA->GTA	**EFR3A**	Ile786Val	rs147579193	0,004	102	31	30,4	150	0	0,0	0,000	10
**H6548**	chr15	82220087-82220088	C	G	GAG->GAC	**EFTUD1**	Glu478Asp; Glu427Asp	rs2292189	0,726	69	29	42,0	90	0	0,0	0,000	0
**H6548**	chr7	55161561-55161562	G	A	AGG->AAG	**EGFR**	Arg521Lys; Arg476Lys; Arg468Lys	rs2227983	0,287	207	64	30,9	219	0	0,0	0,000	69
**H6548**	chr3	185192688-185192689	C	T	CGA->CAA	**EHHADH**	Arg474Gln; Arg570Gln	rs151323332	0,000	145	37	25,5	225	0	0,0	0,000	14
**H6548**	chr6	31896769-31896770	G	T	ACC->AAC	**EHMT2**	Thr112Asn; Thr55Asn	rs7887	0,382	150	66	44,0	184	0	0,0	0,000	52
**H6548**	chr19	39540063-39540064	C	T	GCA->ACA	**EID2**	Ala6Thr	rs7252027	0,244	99	51	51,5	77	0	0,0	-2,000	0
**H6548**	chr15	39967646-39967647	A	C	ATT->CTT	**EIF2AK4**	Ile441Leu; Ile170Leu	rs2291627	0,153	111	41	36,9	183	0	0,0	0,000	29
**H6548**	chr22	31471916-31471917	C	T	GAA->AAA	**EIF4ENIF1**	Glu33Lys	rs11544091	0,004	69	22	31,9	112	0	0,0	0,000	36

**H6548**	chr15	43776870-43776871	G	C	CAG->GAG	**ELL3**	Gln11Glu	rs2277531	0,150	294	80	27,2	169	0	0,0	0,000	68
**H6548**	chr7	74060494-74060495	G	C	GGG->CGG	**ELN**	Gly587Arg; Gly552Arg; Gly500Arg;	rs17855988	0,070	76	21	27,6	154	0	0,0	0,000	30
**H6548**	chr20	41361973-41361974	C	T	AGC->AAC	**EMILIN3**	Ser532Asn	rs2235592	0,246	307	225	73,3	337	0	0,0	0,000	55
**H6548**	chr11	62602408-62602409	G	A	CAA->TAA	**EML3**	Gln855*; Gln883*	rs35156678	0,261	249	75	30,1	240	0	0,0	0,000	0
**H6548**	chr19	6904125-6904126	C	T	GCA->GTA	**EMR1**	Ala298Val; Ala246Val; Ala157Val	rs370094	0,327	56	23	41,1	128	0	0,0	0,000	19
**H6548**	chr7	155458737-155458738	C	T	CTC->TTC	**EN2**	Leu121Phe	rs3735653	0,412	169	46	27,2	58	0	0,0	-2,000	22
**H6548**	chr17	79082788-79082789	C	A	CCC->CAC	**ENGASE**	Pro23His	rs74001528	0,148	237	78	32,9	176	0	0,0	0,000	1
**H6548**	chr6	46140014-46140015	C	G	CAC->CAG	**ENPP4**	His144Gln	rs7451713	0,515	89	48	53,9	163	0	0,0	0,000	0
**H6548**	chr6	46168057-46168058	T	C	ATT->GTT	**ENPP5**	Ile69Val	rs34432940	0,006	52	16	30,8	157	0	0,0	0,000	10
**H6548**	chr2	47373966-47373967	T	C	ATG->ACG	**EPCAM**	Met115Thr	rs1126497	0,526	97	51	52,6	131	0	0,0	0,000	85
**H6548**	chr18	45928889-45928890	C	G	GAG->GAC	**EPG5**	Glu844Asp	rs3744999	0,057	79	21	26,6	104	0	0,0	0,000	9
**H6548**	chr6	93410920-93410921	T	C	ATA->GTA	**EPHA7**	Ile138Val	rs2278107	0,050	67	34	50,8	112	0	0,0	0,000	30
**H6548**	chr7	100818627-100818628	A	G	TCT->TCC; TCT->TCC	**EPHB4**	Ser345Ser; Ser438Ser; Leu411Pro	rs144173	0,604	165	45	27,3	290	0	0,0	0,000	45

**H6548**	chr8	143868185-143868186	T	C	ATC->GTC	**EPPK1**	Ile1690Val	rs113992087	0,000	579	163	28,2	379	0	0,0	0,000	28
**H6548**	chr19	16361939-16361940	C	T	GAG->AAG	**EPS15L1**	Glu809Lys; Glu712Lys	rs144312610	0,000	97	27	27,8	117	0	0,0	0,000	0
**H6548**	chr1	109757818-109757819	G	A	CAC->TAC	**EPS8L3**	His293Tyr; His294Tyr; His260Tyr;	rs3818562	0,484	138	68	49,3	172	0	0,0	0,000	0
**H6548**	chr2	211424175-211424176	C	T	GTT->ATT	**ERBB4**	Val949Ile; Val939Ile	rs376298364	0,000	51	14	27,5	77	0	0,0	0,000	51
**H6548**	chr12	1480846-1480847	C	T	CCG->CTG	**ERC1**	Pro1073Leu	rs11613546	0,304	76	23	30,3	101	0	0,0	0,000	30
**H6548**	chr3	56080832-56080833	T	C	AAT->AGT	**ERC2**	Asn542Ser; Asn13Ser	rs12488237	0,062	103	32	31,1	204	0	0,0	0,000	13
**H6548**	chr10	49470670-49470671	T	C	ATG->GTG	**ERCC6**	Met257Val; Met467Val; Met465Val;	rs2228526	0,217	71	26	36,6	136	0	0,0	0,000	37
**H6548**	chr16	20798943-20798944	T	C	ATA->GTA	**ERI2**	Ile286Val; Ile193Val; Ile58Val	rs16970675	0,071	75	24	32,0	138	0	0,0	0,000	0
**H6548**	chr13	45596473-45596474	C	T	GAG->AAG	**ERICH6B**	Glu178Lys	rs3014939	0,372	76	22	29,0	78	0	0,0	0,000	0
**H6548**	chr10	100152306-100152307	T	C	ATT->GTT	**ERLIN1**	Ile291Val; Ile207Val	rs2862954	0,327	82	38	46,3	164	0	0,0	0,000	10
**H6548**	chr12	53268839-53268840	C	A	GCC->GAC	**ESPL1**	Ala25Asp	rs6580942	0,781	106	55	51,9	164	0	0,0	0,000	51
**H6548**	chr1	6448811-6448812	C	T	CGC->TGC	**ESPN**	Arg546Cys	rs138198721	0,218	111	34	30,6	50	0	0,0	0,000	8
**H6548**	chr2	67404698-67404699	G	A	GAA->AAA	**ETAA1**	Glu673Lys	rs61740794	0,453	78	31	39,7	181	0	0,0	0,000	52

**H6548**	chr15	76286420-76286421	G	A	ACA->ATA	**ETFA**	Thr171Ile; Thr122Ile	rs1801591	0,076	76	27	35,5	74	0	0,0	0,000	5
**H6548**	chr4	5708461-5708462	G	A	CTC->TTC	**EVC2**	Leu18Phe	rs6820907	0,183	55	26	47,3	29	0	0,0	0,000	2
**H6548**	chr1	92607670-92607671	C	A	CAG->CAT	**EVI5**	Gln612His; Gln623His	rs11808092	0,196	29	13	44,8	68	0	0,0	0,000	35
**H6548**	chr14	103100497-103100498	C	G	GAC->GAG	**EXOC3L4**	Asp93Glu	rs2297066	0,241	143	52	36,4	133	0	0,0	0,000	0
**H6548**	chr6	6174632-6174633	G	A	CCG->CTG	**F13A1**	Pro565Leu; Pro502Leu	rs5982	0,217	98	27	27,6	188	0	0,0	0,000	13
**H6548**	chr1	169542316-169542317	T	C	AAG->GAG	**F5**	Lys925Glu	rs6032	0,273	101	29	28,7	202	0	0,0	0,000	8
**H6548**	chr1	31369472-31369473	T	C	AAA->AGA	**FABP3**	Lys53Arg	rs2228194	0,027	78	25	32,1	184	0	0,0	0,000	15
**H6548**	chr4	186157711-186157712	C	T	CCG->CTG	**FAM149A**	Pro241Leu	rs2276922	0,243	123	34	27,6	72	0	0,0	-2,000	0
**H6548**	chr1	54609778-54609779	G	A	GCG->GTG	**FAM151A**	Ala416Val	rs1368883	0,407	131	42	32,1	169	1	0,6	0,000	0
**H6548**	chr15	82282537-82282538	T	C	TGG->CGG	**FAM154B**	Trp225Arg; Trp210Arg	rs11630197	0,253	33	9	27,3	87	0	0,0	0,000	0
**H6548**	chr4	151577917-151577918	G	A	GCT->ACT	**FAM160A1**	Ala192Thr	rs868550454	-1,000	123	37	30,1	201	0	0,0	0,000	0
**H6548**	chr8	22098596-22098597	A	G	ACC->GCC	**FAM160B2**	Thr315Ala	rs35497596	0,456	151	109	72,2	185	0	0,0	0,000	0
**H6548**	chr2	61840297-61840298	T	C	ATT->GTT	**FAM161A**	Ile236Val; Ile46Val; Ile127Val	rs17513722	0,181	71	18	25,4	89	0	0,0	0,000	3

**H6548**	chr14	73950128-73950129	G	T	GAC->GAA	**FAM161B**	Asp29Glu	rs111833521	0,004	217	63	29,0	532	0	0,0	0,000	0
**H6548**	chr6	116765214-116765215	C	A	CAG->CAT	**FAM162B**	Gln71His	rs654128	0,116	270	83	30,7	329	0	0,0	0,000	0
**H6548**	chr8	11444091-11444092	G	C	ACT->AGT	**FAM167A**	Thr107Ser	rs3021512	0,146	293	107	36,5	241	1	0,4	0,000	12
**H6548**	chr5	119634264-119634265	C	T	CCC->TCC	**FAM170A**	Pro173Ser; Pro126Ser	rs328694	0,447	107	35	32,7	247	0	0,0	0,000	0
**H6548**	chr2	29017193-29017194	A	G	CAG->CGG	**FAM179A**	Gln362Arg	rs11127202	0,221	69	18	26,1	124	0	0,0	0,000	0
**H6548**	chr4	17705825-17705826	C	T	GGC->AGC	**FAM184B**	Gly366Ser	rs61741460	0,035	66	20	30,3	178	0	0,0	0,000	0
**H6548**	chr7	102749249-102749250	C	T	CTC->TTC	**FAM185A**	Leu15Phe	rs141352868	0,106	436	155	35,6	440	0	0,0	0,000	0
**H6548**	chr9	34834136-34834137	A	G	CTG->CCG	**FAM205B**	Leu449Pro	rs146266330	0,020	83	28	33,7	347	0	0,0	0,000	0
**H6548**	chr10	5762567-5762568	G	A	AGT->AAT	**FAM208B**	Ser2404Asn; Ser1248Asn	rs2797501	0,858	21	8	38,1	45	0	0,0	0,001	0
**H6548**	chr6	4068898-4068899	T	C	ATG->GTG	**FAM217A**	Met442Val; Met379Val	rs10485172	0,098	107	34	31,8	178	0	0,0	0,000	0
**H6548**	chr10	122850510-122850511	G	A	CCT->CTT	**FAM24B**	Pro2Leu	rs1891110	0,527	52	13	25,0	91	0	0,0	0,000	0
**H6548**	chr6	116515831-116515832	C	T	ACG->ATG	**FAM26E**	Thr258Met	rs200089126	0,000	104	26	25,0	214	0	0,0	0,000	0
**H6548**	chr6	116463633-116463634	G	A	GAA->AAA	**FAM26F**	Glu293Lys; Glu121Lys	rs11544160	0,058	68	18	26,5	75	0	0,0	0,000	0

**H6548**	chr10	87170491-87170492	A	T	AGT->TGT	**FAM35A**	Ser550Cys	rs11202365	0,343	43	13	30,2	40	0	0,0	0,004	45
**H6548**	chr6	81752010-81752011	C	-A^CCGCCGAAGTCGCCG		**FAM46A**	Gly63; Gly44	rs771239762	0,097	133	58	43,6	94	0	0,0	-2,000	39
**H6548**	chrX	80443093-80443094	C	G	GAC->GAG	**FAM46D**	Asp185Glu	rs1113265	0,550	46	31	67,4	111	0	0,0	0,000	0
**H6548**	chrX	37011180-37011181	A	C	AAT->ACT	**FAM47C**	Asn924Thr	rs1995914	0,217	61	45	73,8	104	0	0,0	0,000	0
**H6548**	chr4	76271714-76271715	G	A	GAG->AAG	**FAM47E**	Glu175Lys; Glu105Lys; Glu273Lys;	rs3733250	0,376	80	41	51,3	128	0	0,0	0,000	0
**H6548**	chr8	143739866-143739867	G	T	GGG->TGG	**FAM83H-AS1**	Gly32Trp	rs7831467	0,258	222	70	31,5	128	0	0,0	0,000	0
**H6548**	chr8	12427740-12427741	G	A	CGG->TGG	**FAM86B2**	Arg270Trp; Arg79Trp; Arg42Trp	rs199873615	0,199	26	8	30,8	29	0	0,0	0,009	0
**H6548**	chr16	89771677-89771678	C	A	ATG->ATT	**FANCA**	Met717Ile	rs1131660	0,022	76	24	31,6	129	0	0,0	0,000	65
**H6548**	chr10	125980284-125980285	G	C	AGG->AGC	**FANK1**	Arg40Ser; Arg46Ser	rs17153882	0,024	106	28	26,4	239	0	0,0	0,000	5
**H6548**	chr13	98440743-98440744	C	T	CGC->TGC	**FARP1**	Arg933Cys; Arg902Cys	rs61749894	0,006	315	89	28,3	179	0	0,0	0,000	14
**H6548**	chr20	3147931-3147932	A	C	ATA->AGA	**FASTKD5**	Ile380Arg	rs41304800	0,075	81	21	25,9	159	0	0,0	0,000	0
**H6548**	chr4	186709435-186709436	G	A	GCG->GTG	**FAT1**	Ala131Val	rs3733415	0,183	61	16	26,2	192	0	0,0	0,000	53
**H6548**	chr5	151566874-151566875	A	G	TTT->TCT	**FAT2**	Phe686Ser	rs9324700	0,466	105	32	30,5	166	0	0,0	0,000	31

**H6548**	chr11	92857281-92857282	A	G	AGT->GGT	**FAT3**	Ser3812Gly; Ser252Gly	rs4753069	0,818	127	33	26,0	191	0	0,0	0,000	54
**H6548**	chr4	125317768-125317769	A	T	CAG->CTG	**FAT4**	Gln453Leu	rs6847454	0,423	179	45	25,1	441	0	0,0	0,000	62
**H6548**	chr17	75919721-75919722	G	A	GCG->GTG	**FBF1**	Ala671Val; Ala680Val; Ala681Val	rs113062332	0,022	169	58	34,3	118	0	0,0	0,000	31
**H6548**	chr3	13571435-13571436	A	G	AGC->GGC	**FBLN2**	Ser387Gly; Ser361Gly	rs3732666	0,304	402	126	31,3	282	0	0,0	0,000	43
**H6548**	chr5	128393156-128393157	C	G	CAG->CAC	**FBN2**	Gln481His; Gln448His		0,000	108	30	27,8	191	0	0,0	0,000	14
**H6548**	chr19	8123798-8123799	G	C	CGA->GGA	**FBN3**	Arg981Gly		0,000	84	30	35,7	84	0	0,0	0,000	36
**H6548**	chr8	144356874-144356875	C	G	GGG->GCG	**FBXL6**	Gly265Ala; Gly271Ala; Gly32Ala	rs61746974	0,054	208	67	32,2	238	0	0,0	0,000	0
**H6548**	chr2	229996802-229996803	G	T	TTG->TTT	**FBXO36**	Leu86Phe; Leu55Phe	rs1035834	0,212	48	17	35,4	73	0	0,0	0,000	0
**H6548**	chr17	6780364-6780365	A	G	TAT->TGT	**FBXO39**	Tyr166Cys	rs16956264	0,146	59	18	30,5	114	0	0,0	0,000	0
**H6548**	chr19	45713383-45713384	G	T	CCT->ACT	**FBXO46**	Pro38Thr	rs11537711	0,126	210	60	28,6	165	0	0,0	0,000	0
**H6548**	chr22	32475377-32475378	G	A	GGG->GAG	**FBXO7**	Gly6Glu	rs9621461	0,088	244	62	25,4	191	0	0,0	0,000	17
**H6548**	chr17	18749756-18749757	G	A	GAA->AAA	**FBXW10**	Glu236Lys	rs9895749	0,359	162	71	43,8	339	0	0,0	0,000	0
**H6548**	chr5	73052444-73052445	A	G	ATG->GTG	**FCHO2**	Met371Val; Met338Val	rs185435	0,249	73	20	27,4	104	0	0,0	0,000	7

**H6548**	chr9	134887179-134887180	C	T	ACG->ATG	**FCN2**	Thr236Met; Thr198Met	rs17549193	0,262	39	12	30,8	93	0	0,0	0,000	5
**H6548**	chr1	157768647-157768648	T	A	ACT->TCT	**FCRL2**	Thr217Ser	rs148798001	0,001	67	19	28,4	146	0	0,0	0,000	50
**H6548**	chr8	11802852-11802853	T	C	CTT->CTC	**FDFT1**	Leu7Leu;	rs1047643	0,159	271	77	28,4	251	2	0,8	0,000	22
**H6548**	chr5	108946207-108946208	C	G	CTG->GTG	**FER**	Leu439Val; Leu264Val; Leu70Val	rs2229086	0,059	100	26	26,0	166	0	0,0	0,000	14
**H6548**	chr20	35560230-35560231	G	A	CGG->TGG	**FER1L4**	Arg103Trp	rs224424	0,608	276	168	60,9	275	0	0,0	0,000	61
**H6548**	chr19	35352182-35352183	G	A	CGC->CAC	**FFAR1**	Arg211His	rs2301151	0,829	445	196	44,0	219	0	0,0	0,000	14
**H6548**	chr4	154586437-154586438	T	C	ACT->GCT	**FGA**	Thr331Ala	rs6050	0,289	133	43	32,3	269	0	0,0	0,000	6
**H6548**	chr6	37008860-37008861	G	C	CAG->CAC	**FGD2**	Gln32His	rs831510	0,297	63	16	25,4	119	0	0,0	0,000	0
**H6548**	chr19	48758110-48758111	T	C	CTC->CCC	**FGF21**	Leu174Pro	rs739320	0,733	149	79	53,0	77	1	1,3	0,000	11
**H6548**	chr12	4370382-4370383	G	A	ACG->ATG	**FGF23**	Thr239Met	rs7955866	0,125	267	74	27,7	157	0	0,0	0,000	14
**H6548**	chr10	91908352-91908353	T	A	GAG->GTG	**FGFBP3**	Glu206Val	rs1107947	0,192	249	80	32,1	214	0	0,0	0,000	0
**H6548**	chr5	177089629-177089630	G	A	GTC->ATC	**FGFR4**	Val10Ile	rs1966265	0,240	114	29	25,4	252	1	0,4	0,000	49
**H6548**	chr4	1025222-1025223	C	T	CCA->CTA	**FGFRL1**	Pro464Leu	rs4647932	0,058	267	78	29,2	175	0	0,0	0,000	27

**H6548**	chr8	17874451-17874452	G	A	CCA->CTA	**FGL1**	Pro105Leu; Pro155Leu	rs2653406	0,116	65	17	26,2	139	0	0,0	0,000	48
**H6548**	chr1	15289642-15289643	G	A	CGC->CAC	**FHAD1**	Arg182His	rs486557	0,284	202	54	26,7	215	0	0,0	0,000	0
**H6548**	chr11	65887921-65887922	G	C	TCT->TGT	**FIBP**	Ser99Cys	rs641018	0,235	197	64	32,5	236	0	0,0	0,000	42
**H6548**	chr6	109786313-109786314	T	C	GTG->GCG	**FIG4**	Val654Ala; Val377Ala	rs9885672	0,282	43	16	37,2	104	1	1,0	0,000	5
**H6548**	chr20	44311052-44311053	C	T	ATG->ATA	**FITM2**	Met32Ile	rs148377517	0,018	264	84	31,8	187	1	0,5	0,000	7
**H6548**	chr1	152358763-152358764	G	A	CTT->TTT	**FLG2**	Leu41Phe	rs3818831	0,253	71	18	25,4	106	0	0,0	0,000	10
**H6548**	chr20	29416808-29416809	C	G	CAG->CAC	**FLJ20518**	Gln30His		0,000	34	10	29,4	42	0	0,0	0,004	0
**H6548**	chr19	7870829-7870830	G	A	ACG->ATG	**FLJ22184**	Thr805Met	rs12984133	0,253	232	59	25,4	218	0	0,0	0,000	0
**H6548**	chr5	180630292-180630293	T	C	AAC->GAC	**FLT4**	Asn149Asp	rs34221241	0,074	173	76	43,9	202	0	0,0	0,000	42
**H6548**	chr1	171205391-171205392	A	G	GAG->GGG	**FMO2**	Glu94Gly; Glu314Gly	rs2020863	0,134	61	37	60,7	134	0	0,0	0,000	7
**H6548**	chr11	47767111-47767112	G	-C^GGTGGT		**FNBP4**	Thr59	rs67450550	0,461	254	86	33,9	99	0	0,0	0,000	28
**H6548**	chr13	49175461-49175462	C	T	TCC->TTC	**FNDC3A**	Ser484Phe; Ser410Phe; Ser428Phe		0,000	54	15	27,8	149	1	0,7	0,000	0
**H6548**	chr1	108718886-108718887	A	G	ATT->GTT	**FNDC7**	Ile146Val	rs17553619	0,076	96	27	28,1	193	0	0,0	0,000	0

**H6548**	chr9	20953049-20953050	A	G	ACA->GCA	**FOCAD**	Thr1373Ala; Thr809Ala	rs3206852	0,381	82	29	35,4	129	0	0,0	0,000	0
**H6548**	chr14	37591440-37591441	C	T	AGC->AAC	**FOXA1**	Ser415Asn; Ser448Asn	rs33984772	0,068	209	65	31,1	328	0	0,0	0,000	61
**H6548**	chr16	86568826-86568827	T	C	TGC->CGC	**FOXC2**	Cys498Arg	rs61753346	0,001	142	41	28,9	160	0	0,0	0,000	35
**H6548**	chr9	117505-117506	G	A	CCG->CTG	**FOXD4**	Pro205Leu	rs2492215	0,043	141	38	27,0	61	0	0,0	-2,000	30
**H6548**	chr16	86579536-86579537	G	A	GGC->AGC	**FOXL1**	Gly272Ser	rs62051072	0,033	334	104	31,1	223	0	0,0	0,000	40
**H6548**	chr17	28524583-28524584	C	T	CGC->TGC	**FOXN1**	Arg69Cys	rs2071587	0,102	251	65	25,9	177	1	0,6	0,000	32
**H6548**	chr8	23573893-23573894	A	G	ACT->GCT	**FP15737**	Thr94Ala	rs3174040	0,255	81	26	32,1	197	0	0,0	0,000	0
**H6548**	chr4	78066002-78066003	A	G	GAT->GGT	**FRAS1**	Asp32Gly	rs4859905	0,460	82	47	57,3	138	0	0,0	0,000	18
**H6548**	chr13	38856176-38856177	C	T	ACC->ATC	**FREM2**	Thr2326Ile; Thr412Ile	rs9548509	0,741	141	71	50,4	292	0	0,0	0,000	13
**H6548**	chr6	116003978-116003979	C	T	GGA->AGA	**FRK**	Gly122Arg	rs3756772	0,456	62	22	35,5	76	0	0,0	0,000	41
**H6548**	chr6	168062943-168062944	G	A	CGT->TGT	**FRMD1**	Arg29Cys; Arg45Cys; Arg186Cys;	rs902393	0,072	172	59	34,3	139	0	0,0	0,000	0
**H6548**	chr13	32202478-32202479	T	A	TGT->AGT	**FRY**	Cys1324Ser	rs61750791	0,073	78	42	53,9	114	0	0,0	0,000	11
**H6548**	chr14	44506402-44506403	G	T	CAC->CAA	**FSCB**	His195Gln	rs3809429	0,189	94	28	29,8	198	0	0,0	0,000	0

**H6548**	chr19	4306313-4306314	T	C	CGT->CGC; TAC->CAC	**FSD1**	Arg63Arg; Tyr60His; Arg76Arg	rs4807578	0,621	25	14	56,0	109	0	0,0	0,000	19
**H6548**	chr2	48963901-48963902	C	T	GCT->ACT	**FSHR**	Ala307Thr; Ala281Thr; Ala245Thr	rs6165	0,543	44	14	31,8	88	0	0,0	0,000	15
**H6548**	chr15	39617850-39617851	C	G	GGC->GCC	**FSIP1**	Gly528Ala	rs16969386	0,142	85	22	25,9	173	0	0,0	0,000	61
**H6548**	chr2	185813905-185813906	C	T	ACT->ATT	**FSIP2**	Thr6819Ile	rs17826666	0,074	159	42	26,4	196	0	0,0	0,000	21
**H6548**	chr21	46137307-46137308	G	A	GGC->GGT; GGC->GGT	**FTCD**	Gly490Gly; Arg476Cys; Gly359Gly	rs10432965	0,090	137	49	35,8	226	0	0,0	0,000	7
**H6548**	chrX	37043315-37043316	C	T	GGG->AGG	**FTH1P18**	Gly125Arg	rs28675922	0,307	206	178	86,4	130	0	0,0	0,000	0
**H6548**	chr19	5843772-5843773	A	T	ATA->AAA	**FUT3**	Ile356Lys	rs3894326	0,087	211	77	36,5	163	0	0,0	0,000	30
**H6548**	chr14	65669444-65669445	C	A	ACA->AAA	**FUT8**	Thr267Lys; Thr104Lys; Thr138Lys	rs35949016	0,171	102	30	29,4	215	0	0,0	0,000	42
**H6548**	chr7	1534546-1534547	C	T	CGG->CAG	**FW339998**	Arg66Gln	rs145176413	0,014	175	53	30,3	222	0	0,0	0,000	0
**H6548**	chr3	45936447-45936448	A	G	ATG->ACG	**FYCO1**	Met1347Thr; Met1367Thr	rs781176882	0,000	95	26	27,4	141	0	0,0	0,000	33
**H6548**	chr8	103325138-103325139	A	C	ATG->CTG	**FZD6**	Met345Leu; Met313Leu; Met40Leu;	rs3808553	0,462	130	41	31,5	224	0	0,0	0,000	35
**H6548**	chr17	80113241-80113242	G	A	GAG->AAG	**GAA**	Glu689Lys	rs1800309	0,087	163	59	36,2	85	0	0,0	-2,000	11
**H6548**	chrX	152652813-152652814	A	T	ATT->TTT	**GABRQ**	Ile478Phe	rs3810651	0,470	70	36	51,4	158	0	0,0	0,000	8

**H6548**	chr6	89217242-89217243	T	C	CAC->CGC	**GABRR1**	His27Arg	rs1186902	0,260	66	32	48,5	135	0	0,0	0,000	9
**H6548**	chrX	49323275-49323276	G	A	CGG->CAG	**GAGE12J**	Arg28Gln	rs7064530	0,461	137	115	83,9	109	0	0,0	0,000	0
**H6548**	chr14	87976367-87976368	C	T	GAT->AAT	**GALC**	Asp192Asn; Asp248Asn; Asp225Asn;	rs34362748	0,123	102	30	29,4	112	0	0,0	0,000	7
**H6548**	chr7	151987170-151987171	A	G	GAA->GGA	**GALNTL5**	Glu72Gly; Glu183Gly	rs61729490	0,190	42	23	54,8	90	0	0,0	0,000	0
**H6548**	chr15	42287750-42287751	G	A	GAA->AAA	**GANC**	Glu88Lys	rs145853612	0,008	125	40	32,0	149	0	0,0	0,000	22
**H6548**	chr17	35745535-35745536	G	A	GCC->GTC	**GAS2L2**	Ala654Val	rs3744374	0,211	145	59	40,7	405	2	0,5	0,000	0
**H6548**	chr1	89186418-89186419	A	T	TAC->AAC	**GBP4**	Tyr541Asn	rs655260	0,428	75	19	25,3	163	0	0,0	0,000	25
**H6548**	chr1	89171894-89171895	G	A	ACT->ATT	**GBP7**	Thr14Ile	rs676913	0,698	192	56	29,2	378	1	0,3	0,000	0
**H6548**	chr19	18386330-18386331	T	A	TCC->ACC	**GDF15**	Ser48Thr	rs1059369	0,264	150	46	30,7	150	0	0,0	0,000	47
**H6548**	chr12	7689990-7689991	C	G	GTG->CTG	**GDF3**	Val328Leu	rs2302516	0,034	67	18	26,9	156	0	0,0	0,000	23
**H6548**	chr11	77243787-77243788	G	A	CAT->TAT	**GDPD4**	His383Tyr	rs11237146	0,296	70	39	55,7	134	0	0,0	0,000	0
**H6548**	chr17	745991-745992	C	T	CGA->CAA	**GEMIN4**	Arg684Gln	rs3744741	0,200	63	19	30,2	105	0	0,0	0,000	40
**H6548**	chr19	45678133-45678134	G	C	GAG->CAG	**GIPR**	Glu354Gln; Glu318Gln	rs1800437	0,200	89	38	42,7	108	0	0,0	0,000	12

**H6548**	chr1	34794600-34794601	G	A	GTA->ATA	**GJA4**	Val130Ile; Val206Ile	rs41266431	0,095	570	175	30,7	392	1	0,3	0,000	10
**H6548**	chr15	69255998-69255999	A	G	ATG->GTG	**GLCE**	Met65Val	rs12440300	0,021	60	20	33,3	139	0	0,0	0,000	31
**H6548**	chr16	4337422-4337423	A	G	ACG->GCG	**GLIS2**	Thr492Ala	rs8057701	0,061	311	80	25,7	120	0	0,0	0,000	49
**H6548**	chr9	4285985-4285986	G	C	CCC->CGC	**GLIS3**	Pro147Arg	rs10758591	0,442	95	24	25,3	163	0	0,0	0,000	16
**H6548**	chr2	190921009-190921010	T	-C^TT		**GLS**	Leu3	rs770407339	0,000	25	12	48,0	40	1	2,5	0,000	13
**H6548**	chr12	56471553-56471554	A	G	CTC->CCC	**GLS2**	Leu544Pro; Leu316Pro; Leu305Pro;	rs2657879	0,165	78	43	55,1	171	0	0,0	0,000	56
**H6548**	chrX	121049175-121049176	T	G	TCG->GCG	**GLUD2**	Ser498Ala	rs9697983	0,028	52	13	25,0	74	0	0,0	0,000	11
**H6548**	chr9	77647696-77647697	G	A	CGC->TGC	**GNA14**	Arg33Cys	rs138686336	0,006	275	74	26,9	97	0	0,0	0,000	23
**H6548**	chr9	124899365-124899366	G	A	ACG->ATG	**GOLGA1**	Thr425Met	rs634710	0,412	123	35	28,5	187	0	0,0	0,000	0
**H6548**	chr3	37355186-37355187	G	A	ATG->ATA	**GOLGA4**	Met2221Ile; Met2236Ile; Met2102Ile	rs148417077	0,002	148	53	35,8	168	0	0,0	0,000	15
**H6548**	chr15	34528067-34528068	G	A	GCT->GTT	**GOLGA8B**	Ala345Val; Ala344Val; Ala488Val	rs142225671	0,651	36	25	69,4	17	0	0,0	0,000	0
**H6548**	chr15	30145489-30145490	C	G	CAG->GAG	**GOLGA8K**	Gln538Glu	rs201978730	0,148	41	23	56,1	48	0	0,0	0,000	0
**H6548**	chr15	23364402-23364403	G	T	GAA->TAA	**GOLGA8S**	Glu261*	rs140059935	0,125	113	65	57,5	146	0	0,0	0,000	0

**H6548**	chr3	121692408-121692409	G	A	CTT->TTT	**GOLGB1**	Leu2314Phe; Leu2319Phe; Leu2280Phe;	rs769851315	0,000	79	20	25,3	161	0	0,0	0,000	24
**H6548**	chr19	55015004-55015005	G	C	CTC->CTG; CCC->GCC	**GP6**	Leu312Leu; Pro314Ala; Leu294Leu	rs2304166	0,536	177	67	37,9	112	0	0,0	0,000	53
**H6548**	chr10	112180570-112180571	T	C	ATC->GTC	**GPAM**	Ile43Val	rs2792751	0,745	60	22	36,7	115	0	0,0	0,000	12
**H6548**	chr2	240462463-240462464	G	A	CGG->CAG	**GPC1**	Arg200Gln	rs761559309	0,000	356	93	26,1	140	0	0,0	0,000	33
**H6548**	chr2	27629050-27629051	G	A	AGG->AAG	**GPN1**	Arg12Lys	rs3749147	0,224	85	38	44,7	252	0	0,0	0,000	25
**H6548**	chrX	137030547-137030548	A	G	CTC->CCC	**GPR101**	Leu376Pro	rs5931046	0,174	86	29	33,7	151	0	0,0	0,000	61
**H6548**	chr6	47679011-47679012	T	C	TCC->CCC	**GPR111**	Ser117Pro; Ser49Pro	rs13212023	0,135	68	24	35,3	135	0	0,0	0,000	0
**H6548**	chr6	46859501-46859502	C	T	GTC->ATC	**GPR116**	Val356Ile; Val230Ile; Val801Ile;	rs9395218	0,250	38	11	29,0	49	0	0,0	0,002	24
**H6548**	chr3	100649701-100649702	A	G	AAA->AGA	**GPR128**	Lys425Arg; Lys126Arg	rs61730367	0,093	63	16	25,4	102	0	0,0	0,000	0
**H6548**	chr1	9105625-9105626	G	A	CGC->TGC	**GPR157**	Arg218Cys	rs72637739	0,219	300	108	36,0	250	0	0,0	0,000	0
**H6548**	chr10	25395982-25395983	C	A	CCT->ACT	**GPR158**	Pro361Thr	rs145500194	0,001	50	13	26,0	125	0	0,0	0,000	20
**H6548**	chr12	12662102-12662103	C	T	GTT->ATT	**GPR19**	Val116Ile	rs41276680	0,015	110	28	25,5	183	0	0,0	0,000	28
**H6548**	chr1	202123203-202123204	C	G	CCC->GCC	**GPR37L1**	Pro81Ala	rs3795594	0,145	152	55	36,2	192	0	0,0	0,000	10

**H6548**	chr2	53854653-53854654	C	A	GAT->TAT	**GPR75**	Asp35Tyr	rs543604312	0,000	162	41	25,3	200	0	0,0	0,000	12
**H6548**	chr5	90756471-90756472	G	A	GAG->AAG	**GPR98**	Glu1573Lys; Glu3867Lys; Glu1467Lys	rs10062026	0,344	54	19	35,2	110	0	0,0	0,000	6
**H6548**	chr1	108904127-108904128	G	A	GGG->AGG	**GPSM2**	Gly356Arg	rs61754640	0,022	41	17	41,5	56	0	0,0	0,000	24
**H6548**	chr19	34996220-34996221	G	A	GCC->ACC	**GRAMD1A**	Ala67Thr	rs67018685	0,185	81	21	25,9	89	0	0,0	0,000	0
**H6548**	chr21	29553702-29553703	G	A	GCT->GTT	**GRIK1**	Ala870Val	rs363503	0,005	42	12	28,6	99	1	1,0	0,000	7
**H6548**	chr6	146434187-146434188	T	C	TCC->CCC	**GRM1**	Ser993Pro	rs6923492	0,534	214	80	37,4	184	0	0,0	0,000	40
**H6548**	chr4	7068028-7068029	C	T	GCG->ACG	**GRPEL1**	Ala2Thr	rs760261432	0,000	265	80	30,2	203	0	0,0	0,000	23
**H6548**	chr19	48451163-48451164	G	A	CGG->CAG	**GRWD1**	Arg319Gln	rs2302951	0,180	168	56	33,3	171	0	0,0	0,000	45
**H6548**	chr17	39966432-39966433	G	A	GAG->AAG	**GSDMA**	Glu130Lys	rs7212944	0,326	44	11	25,0	97	0	0,0	0,000	63
**H6548**	chr8	129748603-129748604	A	G	ATG->ACG	**GSDMC**	Met475Thr	rs4144738	0,556	100	25	25,0	195	0	0,0	0,000	63
**H6548**	chr17	3724545-3724546	G	A	GGC->GAC	**GSG2**	Gly204Asp	rs220462	0,305	215	59	27,4	209	0	0,0	0,000	30
**H6548**	chr16	11915673-11915674	C	-C^CCGCCGCCG		**GSPT1**	Gly16	rs749531224	0,057	207	58	28,0	134	0	0,0	0,000	57
**H6548**	chr1	109737078-109737079	C	T	GTA->ATA	**GSTM3**	Val224Ile; Val221Ile	rs7483	0,342	33	12	36,4	61	0	0,0	0,000	56

**H6548**	chr10	104267300-104267301	G	A	GAA->AAA	**GSTO1**	Glu180Lys; Glu208Lys; Glu175Lys	rs11509438	0,038	92	26	28,3	113	0	0,0	0,000	33
**H6548**	chr16	27463588-27463589	A	G	TTC->TCC	**GTF3C1**	Phe1959Ser; Phe1934Ser	rs12919017	0,119	137	41	29,9	176	0	0,0	0,000	0
**H6548**	chr9	132670607-132670608	G	A	GCC->ACC	**GTF3C4**	Ala4Thr	rs143172300	0,012	137	37	27,0	61	0	0,0	-2,000	0
**H6548**	chr14	21888370-21888371	T	C	TTC->TCC	**GU122926**	Phe71Ser	rs10483261	0,514	81	39	48,2	158	0	0,0	0,000	0
**H6548**	chr3	108920536-108920537	T	C	ATG->GTG	**GUCA1C**	Met85Val	rs6804162	0,342	40	12	30,0	83	0	0,0	0,000	0
**H6548**	chr11	76704164-76704165	G	A	CCC->TCC	**GUCY2EP**	Pro511Ser	rs10899257	0,187	64	17	26,6	104	0	0,0	0,000	0
**H6548**	chr11	6718761-6718762	G	A	CAG->TAG	**GVINP1**	Gln426*	rs141319423	0,002	58	21	36,2	141	0	0,0	0,000	0
**H6548**	chrY	12406888-12406889	T	G	CAG->CCG	**GYG2P1**	Gln58Pro	rs200150033	0,017	43	18	41,9	89	0	0,0	0,000	0
**H6548**	chr20	23365206-23365207	A	C	CAG->CCG	**GZF1**	Gln275Pro	rs6048760	0,538	186	105	56,5	211	0	0,0	0,000	0
**H6548**	chr14	24631075-24631076	A	G	TAC->CAC	**GZMB**	Tyr247His; Tyr235His	rs2236338	0,248	126	36	28,6	164	0	0,0	0,000	39
**H6548**	chr12	48330000-48330001	C	T	TCC->TTC	**H1FNT**	Ser237Phe	rs2291483	0,219	156	48	30,8	94	0	0,0	0,000	0
**H6548**	chrX	104040192-104040193	C	T	CAG->TAG	**H2BFM**	Gln73*	rs2301384	0,438	65	45	69,2	83	0	0,0	0,000	0
**H6548**	chr17	41734462-41734463	A	T	TCC->ACC	**HAP1**	Ser58Thr	rs4796603	0,792	186	78	41,9	180	0	0,0	0,000	16

**H6548**	chr9	19058922-19058923	G	A	GCT->GTT	**HAUS6**	Ala479Val; Ala615Val; Ala580Val	rs62622380	0,121	29	9	31,0	79	0	0,0	0,000	0
**H6548**	chr6	135037428-135037429	T	G	GAA->GCA	**HBS1L**	Glu343Ala	rs7742542	0,504	54	34	63,0	84	0	0,0	0,000	14
**H6548**	chr22	50245985-50245986	T	C	CAG->CGG	**HDAC10**	Gln586Arg; Gln566Arg; Gln379Arg	rs11553697	0,041	204	84	41,2	111	0	0,0	0,000	58
**H6548**	chr1	236587408-236587409	C	T	GGA->AGA	**HEATR1**	Gly570Arg	rs56242514	0,021	69	20	29,0	120	0	0,0	0,000	0
**H6548**	chr17	60078702-60078703	G	A	GCC->GTC	**HEATR6**	Ala71Val	rs3744376	0,079	165	43	26,1	128	0	0,0	0,000	0
**H6548**	chr4	83429585-83429586	T	C	AAG->AGG	**HELQ**	Lys819Arg; Lys752Arg	rs75238520	0,004	28	8	28,6	68	0	0,0	0,002	56
**H6548**	chr4	185019797-185019798	C	G	CTG->GTG	**HELT**	Leu62Val; Leu6Val; Leu147Val	rs1078461	0,110	147	53	36,1	118	0	0,0	0,000	22
**H6548**	chr20	63562091-63562092	G	A	ACG->ATG	**HELZ2**	Thr1601Met; Thr2170Met	rs3810481	0,186	263	78	29,7	222	0	0,0	0,000	0
**H6548**	chr4	88656062-88656063	A	T	AAG->ATG	**HERC3**	Lys366Met	rs2972040	0,336	37	11	29,7	105	0	0,0	0,000	38
**H6548**	chr17	82433807-82433808	A	G	ATC->GTC	**HEXDC**	Ile145Val	rs4789773	0,449	183	93	50,8	200	0	0,0	0,000	0
**H6548**	chr8	132079890-132079891	G	A	CCC->CTC	**HHLA1**	Pro108Leu; Pro251Leu	rs2403730	0,293	143	61	42,7	295	0	0,0	0,000	0
**H6548**	chr3	108362369-108362370	C	A	AAC->AAA	**HHLA2**	Asn280Lys; Asn344Lys	rs3792332	0,067	86	23	26,7	171	1	0,6	0,000	71
**H6548**	chr11	119134421-119134422	C	T	GCC->GTC	**HINFP**	Ala493Val; Ala246Val	rs100803	0,415	96	40	41,7	220	0	0,0	0,000	45

**H6548**	chr6	26107561-26107562	G	T	CAG->AAG	**HIST1H1T**	Gln178Lys	rs198845	0,328	90	32	35,6	164	0	0,0	0,000	0
**H6548**	chr6	12120354-12120355	C	T	ACG->ATG	**HIVEP1**	Thr187Met	rs2228209	0,296	132	39	29,6	169	0	0,0	0,000	25
**H6548**	chr1	41584694-41584695	C	T	GTC->ATC	**HIVEP3**	Val35Ile	rs2146315	0,274	151	47	31,1	307	0	0,0	0,000	22
**H6548**	chr6	29943462-29943463	T	A	TTG->TAG	**HLA-A**	Leu180*; Leu59*; Leu152*		0,000	273	75	27,5	300	0	0,0	0,000	0
**H6548**	chr6	31356869-31356870	T	C	GAC->GGC	**HLA-B**	Asp54Gly; Asp26Gly	rs9266183	0,047	200	92	46,0	177	0	0,0	0,000	0
**H6548**	chr6	32661986-32661987	C	T	AGC->AAC	**HLA-DQB1**	Ser169Asn; Ser214Asn		0,000	134	54	40,3	265	0	0,0	0,000	0
**H6548**	chr6	32584353-32584354	C	G	AGG->ACG	**HLA-DRB1**	Arg42Thr	rs1136759	-1,000	49	37	75,5	42	3	7,1	0,000	0
**H6548**	chr6	32521966-32521967	G	T	GCG->GAG	**HLA-DRB5**	Ala103Glu	rs1059598	0,030	189	49	25,9	29	0	0,0	-2,000	0
**H6548**	chr6	29828598-29828599	C	A	CTC->ATC	**HLA-G**	Leu134Ile; Leu139Ile; Leu106Ile	rs12722477	0,141	255	83	32,6	287	0	0,0	0,000	0
**H6548**	chr6	29888085-29888086	G	A	GAG->AAG	**HLA-H**	Glu34Lys	rs200854025	0,000	131	64	48,9	227	0	0,0	0,000	0
**H6548**	chr1	186132406-186132407	A	G	CAA->CGA	**HMCN1**	Gln4437Arg; Gln6Arg	rs10911825	0,412	40	11	27,5	90	0	0,0	0,000	21
**H6548**	chr13	30462504-30462505	C	G	ATG->ATC	**HMGB1**	Met168Ile	rs3742305	0,220	160	47	29,4	254	0	0,0	0,000	35
**H6548**	chr1	13115966-13115967	C	T	CGC->CAC	**HNRNPCP5**	Arg145His	rs28713604	0,249	66	18	27,3	198	0	0,0	0,000	0

**H6548**	chr19	12766149-12766150	G	C	CAC->CAG	**HOOK2**	His488Gln	rs897804	0,331	112	74	66,1	84	0	0,0	-2,000	26
**H6548**	chr1	150706556-150706557	G	A	ACA->ATA	**HORMAD1**	Thr267Ile; Thr260Ile; Thr187Ile	rs1336900	0,400	127	32	25,2	251	0	0,0	0,000	43
**H6548**	chr2	176107439-176107440	G	T	GCT->TCT	**HOXD11**	Ala29Ser	rs149509317	0,003	426	144	33,8	431	0	0,0	0,000	17
**H6548**	chr22	26466074-26466075	G	A	CGA->TGA	**HPS4**	Arg92*; Arg246*	rs3747129	0,282	96	29	30,2	200	0	0,0	0,000	8
**H6548**	chr8	22127431-22127432	C	T	GGT->GAT	**HR**	Gly337Asp	rs12675375	0,320	211	167	79,2	197	0	0,0	0,000	24
**H6548**	chr18	24477005-24477006	A	G	CAT->CGT	**HRH4**	His206Arg; His118Arg	rs11662595	0,082	87	22	25,3	210	0	0,0	0,000	14
**H6548**	chr1	152213664-152213665	C	T	CGC->CAC	**HRNR**	Arg2655His	rs41266128	0,142	140	37	26,4	127	2	1,6	0,000	24
**H6548**	chr13	96832575-96832576	A	G	AAA->AGA	**HS6ST3**	Lys265Arg	rs9516771	0,056	66	18	27,3	182	0	0,0	0,000	16
**H6548**	chr11	43855147-43855148	C	T	TCG->TTG	**HSD17B12**	Ser280Leu; Ser122Leu	rs11555762	0,334	81	22	27,2	102	0	0,0	0,000	38
**H6548**	chr1	162797842-162797843	C	T	ACC->ATC	**HSD17B7**	Thr125Ile	rs143124291	0,000	151	39	25,8	301	0	0,0	0,000	23
**H6548**	chr1	119511591-119511592	A	G	ATC->GTC	**HSD3B1**	Ile79Val; Ile81Val	rs6201	0,052	99	31	31,3	188	0	0,0	0,000	29
**H6548**	chr16	30987820-30987821	A	G	ACC->GCC; CAA->CAG	**HSD3B7**	Thr250Ala; Gln195Gln	rs9938550	0,572	305	232	76,1	280	0	0,0	0,000	9
**H6548**	chr10	14842605-14842606	C	G	CCA->GCA	**HSPA14**	Pro108Ala	rs142727275	0,002	74	21	28,4	170	0	0,0	0,000	16

**H6548**	chr6	31810168-31810169	C	T	GAG->AAG	**HSPA1L**	Glu602Lys	rs2075800	0,326	115	52	45,2	131	0	0,0	0,000	26
**H6548**	chr1	21852750-21852751	C	T	GGC->AGC	**HSPG2**	Gly2225Ser; Gly2226Ser	rs35669711	0,050	148	39	26,4	128	0	0,0	0,000	21
**H6548**	chr11	113932305-113932306	A	C	TAC->TCC	**HTR3B**	Tyr129Ser; Tyr118Ser	rs1176744	0,338	78	21	26,9	184	0	0,0	0,000	12
**H6548**	chr3	50320437-50320438	A	C	TCA->GCA	**HYAL2**	Ser18Ala	rs709210	0,682	176	118	67,1	168	1	0,6	0,000	44
**H6548**	chr1	43453741-43453742	A	G	TCC->CCC	**HYI**	Ser18Pro	rs6692611	0,685	85	24	28,2	52	0	0,0	0,000	3
**H6548**	chr19	10333926-10333927	C	G	AGC->ACC	**ICAM3**	Ser525Thr; Ser448Thr	rs2230399	0,080	187	47	25,1	356	0	0,0	0,000	25
**H6548**	chr20	31605573-31605574	A	G	AAC->GAC	**ID1**	Asn63Asp	rs1802548	0,023	377	131	34,8	243	0	0,0	0,000	51
**H6548**	chr4	1002079-1002080	C	T	AAC->AAT; CGA->TGA	**IDUA**	Asn297Asn; Arg284*	rs114806891	0,080	368	97	26,4	334	1	0,3	0,000	7
**H6548**	chr1	181089481-181089482	C	G	CGC->GGC	**IER5**	Arg194Gly	rs1416829	0,476	105	57	54,3	46	0	0,0	0,000	40
**H6548**	chr1	159054877-159054878	A	T	ACT->TCT	**IFI16**	Thr723Ser	rs6940	0,184	118	30	25,4	125	0	0,0	0,000	36
**H6548**	chr19	39298474-39298475	A	G	AAC->GAC	**IFNL1**	Asn188Asp	rs30461	0,141	281	80	28,5	440	1	0,2	0,000	26
**H6548**	chr19	46230151-46230152	G	A	GGA->AGA	**IGFL1**	Gly24Arg	rs75105417	0,062	28	13	46,4	48	0	0,0	0,000	31
**H6548**	chr3	151448749-151448750	G	T	CCT->ACT	**IGSF10**	Pro411Thr		0,000	46	13	28,3	124	0	0,0	0,000	0

**H6548**	chr3	118902652-118902653	C	T	AGC->AAC	**IGSF11**	Ser388Asn; Ser360Asn; Ser364Asn;	rs34908332	0,066	97	27	27,8	255	0	0,0	0,000	39
**H6548**	chr11	18716781-18716782	C	T	GAG->AAG	**IGSF22**	Glu398Lys	rs61886891	0,180	103	26	25,2	189	0	0,0	0,000	0
**H6548**	chr21	39793534-39793535	C	A	GAC->GAA	**IGSF5**	Asp350Glu	rs2837225	0,368	164	75	45,7	296	0	0,0	0,000	0
**H6548**	chr11	133920599-133920600	C	A	CGG->CTG	**IGSF9B**	Arg1042Leu		0,000	359	133	37,1	311	0	0,0	0,000	25
**H6548**	chr9	108889339-108889340	A	T	TGT->AGT	**IKBKAP**	Cys76Ser; Cys723Ser; Cys1072Ser;	rs3204145	0,200	101	26	25,7	177	1	0,6	0,000	21
**H6548**	chr22	49998058-49998059	G	A	CCG->CTG	**IL17REL**	Pro262Leu	rs142430606	0,011	173	60	34,7	133	0	0,0	0,000	14
**H6548**	chr2	102339007-102339008	C	A	GCA->GAA	**IL1RL1**	Ala78Glu	rs1041973	0,232	134	37	27,6	166	0	0,0	0,000	16
**H6548**	chr1	67168128-67168129	G	T	CAG->CAT	**IL23R**	Gln3His	rs1884444	0,529	42	11	26,2	79	0	0,0	0,000	18
**H6548**	chr22	37128578-37128579	G	T	GAC->GAA	**IL2RB**	Asp391Glu	rs228942	0,185	165	47	28,5	253	0	0,0	0,000	31
**H6548**	chr16	70660096-70660097	C	A	TAC->TAA	**IL34**	Tyr213*; Tyr212*	rs4985556	0,088	152	53	34,9	113	0	0,0	0,000	27
**H6548**	chr5	55956102-55956103	G	C	CTA->GTA	**IL6ST**	Leu108Val; Leu397Val	rs2228043	0,124	68	17	25,0	87	0	0,0	0,000	53
**H6548**	chr2	120346326-120346327	T	G	TCC->GCC	**INHBB**	Ser47Ala	rs11900747	0,250	60	16	26,7	46	0	0,0	0,003	26
**H6548**	chr2	206046503-206046504	G	A	GCG->GTG	**INO80D**	Ala358Val	rs2909111	0,387	189	50	26,5	122	0	0,0	0,000	0

**H6548**	chr1	37864326-37864327	C	G	GAT->CAT	**INPP5B**	Asp707His; Asp871His	rs41311191	0,126	55	15	27,3	107	0	0,0	0,000	5
**H6548**	chr22	31125417-31125418	G	A	GCA->ACA	**INPP5J**	Ala227Thr; Ala160Thr	rs2240432	0,379	123	46	37,4	131	0	0,0	0,000	71
**H6548**	chr9	100292658-100292659	G	A	GGA->GAA	**INVS**	Gly705Glu; Gly801Glu; Gly475Glu	rs76868679	0,027	122	35	28,7	181	0	0,0	0,000	12
**H6548**	chr3	51778935-51778936	C	T	CGG->CAG	**IQCF6**	Arg4Gln	rs11130296	0,192	211	64	30,3	272	0	0,0	0,000	0
**H6548**	chr3	197932288-197932289	C	G	GAT->CAT	**IQCG**	Asp79His; Asp177His	rs67877771	0,195	42	12	28,6	112	0	0,0	0,000	42
**H6548**	chr5	76590466-76590467	G	A	GTT->ATT	**IQGAP2**	Val234Ile; Val184Ile	rs147531108	0,006	71	21	29,6	151	0	0,0	0,000	40
**H6548**	chr7	128947297-128947298	A	-C^ACTCTGCAGCCGCCCACT		**IRF5**	Pro156; Thr168; Thr184	rs199508964	0,474	148	40	27,0	91	0	0,0	-2,000	23
**H6548**	chr17	3729541-3729542	G	A	CGG->TGG	**ITGAE**	Arg950Trp	rs1716	0,315	101	26	25,7	163	0	0,0	0,000	39
**H6548**	chr16	31362135-31362136	C	T	CCC->CTC	**ITGAX**	Pro33Leu; Pro383Leu	rs138501712	0,001	141	56	39,7	322	0	0,0	0,000	33
**H6548**	chr3	52786994-52786995	A	G	CAG->CGG	**ITIH1**	Gln595Arg; Gln453Arg; Gln307Arg;	rs1042779	0,399	105	27	25,7	194	0	0,0	0,000	14
**H6548**	chr3	52807755-52807756	G	A	ATG->ATA	**ITIH3**	Met757Ile; Met565Ile	rs148156289	0,029	81	22	27,2	209	0	0,0	0,000	24
**H6548**	chr10	7576722-7576723	T	G	ACC->CCC	**ITIH5**	Thr356Pro; Thr570Pro	rs2275069	0,545	266	68	25,6	287	0	0,0	0,000	58
**H6548**	chr1	226736940-226736941	C	T	CGC->CAC	**ITPKB**	Arg173His	rs3754415	0,208	388	105	27,1	311	0	0,0	0,000	30

**H6548**	chr2	24208314-24208315	C	T	GCC->ACC	**ITSN2**	Ala1534Thr; Ala1507Thr	rs2303291	0,194	97	33	34,0	178	0	0,0	0,000	33
**H6548**	chr5	134525735-134525736	G	T	TTG->TTT	**JADE2**	Leu14Phe	rs75304543	0,152	224	69	30,8	165	0	0,0	0,000	0
**H6548**	chr14	105149310-105149311	C	T	GAC->AAC	**JAG2**	Asp538Asn; Asp500Asn	rs9972231	0,138	173	60	34,7	161	0	0,0	0,000	37
**H6548**	chr19	11192783-11192784	C	-G^C		**KANK2**	Gly432	rs35706290	0,466	140	66	47,1	54	5	9,3	0,000	56
**H6548**	chr1	15112493-15112494	G	A	GCA->ACA	**KAZN**	Ala706Thr; Ala153Thr	rs10803354	0,478	78	27	34,6	124	0	0,0	0,000	6
**H6548**	chr3	127923970-127923971	G	C	GTA->CTA	**KBTBD12**	Val304Leu	rs61268888	0,159	131	40	30,5	228	0	0,0	0,000	0
**H6548**	chr19	49070867-49070868	G	C	CCG->CGG	**KCNA7**	Pro189Arg	rs1611775	0,374	25	8	32,0	72	1	1,4	0,000	5
**H6548**	chr16	84237136-84237137	G	A	CCC->TCC	**KCNG4**	Pro117Ser	rs3803641	0,056	269	97	36,1	278	0	0,0	0,000	0
**H6548**	chr12	49557593-49557594	C	T	CGT->TGT	**KCNH3**	Arg965Cys; Arg905Cys	rs59261129	0,066	256	83	32,4	140	0	0,0	0,000	9
**H6548**	chr2	232768749-232768750	G	A	ACA->ATA	**KCNJ13**	Thr175Ile; Thr95Ile	rs1801251	0,355	84	28	33,3	119	0	0,0	0,000	9
**H6548**	chr17	21703530-21703531	A	G	ATC->GTC	**KCNJ18**	Ile249Val		0,000	370	218	58,9	373	0	0,0	0,000	0
**H6548**	chr14	88185617-88185618	C	T	GCT->ACT	**KCNK10**	Ala517Thr; Ala512Thr	rs17762463	0,203	169	87	51,5	209	1	0,5	0,000	3
**H6548**	chr19	38327235-38327236	G	A	GTG->ATG	**KCNK6**	Val259Met; Val125Met	rs34989303	0,170	177	49	27,7	193	0	0,0	0,000	3

**H6548**	chr3	179242978-179242979	T	-C^T		**KCNMB3**	Lys255; Lys251; Lys233; Lys253	rs143962239	0,068	64	20	31,3	134	0	0,0	0,000	11
**H6548**	chr1	40831155-40831156	T	G	CAT->CAG	**KCNQ4**	His455Gln; His401Gln	rs34287852	0,197	148	57	38,5	186	0	0,0	0,000	4
**H6548**	chr6	36479197-36479198	G	C	AGT->ACT	**KCTD20**	Ser171Thr; Ser26Thr	rs2239808	0,240	142	67	47,2	185	0	0,0	0,000	0
**H6548**	chr5	138419118-138419119	G	A	AGC->AAC	**KDM3B**	Ser1201Asn; Ser857Asn; Ser233Asn	rs7706614	0,044	50	14	28,0	104	1	1,0	0,000	55
**H6548**	chr11	94998594-94998595	G	A	CGA->CAA	**KDM4D**	Arg408Gln	rs3740853	0,289	147	37	25,2	297	0	0,0	0,000	44
**H6548**	chr7	140097630-140097631	G	T	CGT->AGT	**KDM7A**	Arg644Ser	rs6950119	0,322	58	19	32,8	87	0	0,0	0,000	41
**H6548**	chr4	55106778-55106779	A	G	TGT->CGT	**KDR**	Cys482Arg	rs34231037	0,022	47	18	38,3	103	0	0,0	0,000	33
**H6548**	chr6	73363728-73363729	C	A	CAG->AAG	**KHDC3L**	Gln175Lys	rs747602045	0,000	344	103	29,9	239	1	0,4	0,000	59
**H6548**	chr17	28628311-28628312	A	C	GTT->GGT	**KIAA0100**	Val1516Gly	rs12602520	0,100	52	14	26,9	94	0	0,0	0,000	0
**H6548**	chrX	119096545-119096546	C	T	CGC->CAC	**KIAA1210**	Arg405His	rs145929840	0,009	45	29	64,4	107	0	0,0	0,000	0
**H6548**	chr18	37222066-37222067	C	T	GCG->GTG	**KIAA1328**	Ala384Val; Ala525Val	rs755417719	0,000	51	13	25,5	113	0	0,0	0,000	0
**H6548**	chr1	32770049-32770050	A	G	ATG->GTG	**KIAA1522**	Met291Val; Met243Val; Met232Val	rs12730560	0,143	453	136	30,0	204	0	0,0	0,000	0
**H6548**	chr7	138917670-138917671	G	A	CCG->CTG	**KIAA1549**	Pro652Leu	rs2774960	0,352	84	21	25,0	124	0	0,0	0,000	83

**H6548**	chr1	180936311-180936312	G	T	TTG->TTT	**KIAA1614**	Leu801Phe; Leu422Phe	rs3795504	0,434	263	77	29,3	376	3	0,8	0,000	0
**H6548**	chr22	25029314-25029315	A	G	AAG->AGG	**KIAA1671**	Lys439Arg	rs17667531	0,228	287	94	32,8	410	0	0,0	0,000	0
**H6548**	chr19	18257387-18257388	C	T	CGC->CAC	**KIAA1683**	Arg1299His; Arg1112His; Arg1066His	rs562355836	0,000	274	76	27,7	234	0	0,0	0,000	0
**H6548**	chr1	200553437-200553438	G	C	CCA->GCA	**KIF14**	Pro1142Ala; Pro1633Ala	rs12120084	0,316	99	35	35,4	148	0	0,0	0,000	63
**H6548**	chr3	44830012-44830013	A	T	ACA->TCA	**KIF15**	Thr996Ser; Thr899Ser; Thr44Ser	rs11710339	0,146	84	23	27,4	124	0	0,0	0,000	40
**H6548**	chr17	74354262-74354263	G	A	GCG->ACG	**KIF19**	Ala804Thr	rs2271534	0,114	316	93	29,4	178	0	0,0	0,000	0
**H6548**	chr10	89719422-89719423	G	A	TGT->TAT	**KIF20B**	Cys480Tyr	rs141485845	0,000	48	14	29,2	86	0	0,0	0,000	44
**H6548**	chrX	70341929-70341930	T	G	TTG->TGG	**KIF4A**	Leu422Trp	rs1199457	0,071	76	37	48,7	117	0	0,0	0,000	44
**H6548**	chr19	54767093-54767094	G	A	GAG->AAG	**KIR2DL1**	Glu291Lys	rs11665796	0,029	96	27	28,1	241	0	0,0	0,000	32
**H6548**	chr19	54821683-54821684	G	C	GGG->CGG	**KIR3DL1**	Gly259Arg; Gly164Arg	rs1049215	0,127	88	45	51,1	117	0	0,0	0,000	29
**H6548**	chr19	54853864-54853865	G	T	GAG->GAT	**KIR3DL2**	Glu158Asp	rs1048270	0,277	200	55	27,5	475	0	0,0	0,000	35
**H6548**	chr19	54726136-54726137	G	A	CGT->CAT	**KIR3DL3**	Arg52His	rs113988102	0,316	63	21	33,3	165	1	0,6	0,000	16
**H6548**	chr13	33054055-33054056	G	C	TGC->TCC	**KL**	Cys63Ser; Cys370Ser	rs9527025	0,143	124	32	25,8	215	0	0,0	0,000	16

**H6548**	chr14	103563040-103563041	C	G	CCT->GCT	**KLC1**	Pro14Ala	rs2274268	0,292	185	71	38,4	116	0	0,0	0,000	18
**H6548**	chr6	43061549-43061550	G	A	CGC->CAC	**KLC4**	Arg72His; Arg90His	rs11558979	0,024	77	23	29,9	108	0	0,0	0,000	0
**H6548**	chr2	23643037-23643038	G	C	AGG->AGC	**KLHL29**	Arg399Ser	rs527176	0,508	83	53	63,9	91	0	0,0	0,000	0
**H6548**	chr14	20429516-20429517	T	C	GAG->GGG	**KLHL33**	Glu345Gly	rs1953225	0,222	113	57	50,4	194	1	0,5	0,000	0
**H6548**	chr3	183494237-183494238	C	T	TCG->TCA; CGA->CAA	**KLHL6**	Ser397Ser; Arg316Gln	rs2256061	0,178	76	22	29,0	143	0	0,0	0,000	66
**H6548**	chr19	51015979-51015980	A	G	CTG->CCG	**KLK10**	Leu149Pro	rs2075690	0,570	166	95	57,2	163	0	0,0	0,000	70
**H6548**	chr1	46551088-46551089	C	T	GTC->ATC	**KNCN**	Val43Ile	rs61997191	0,044	106	30	28,3	86	0	0,0	-2,000	0
**H6548**	chr3	186743734-186743735	C	T	CGA->TGA	**KNG1**	Arg412*; Arg376*	rs76438938	0,031	73	20	27,4	104	0	0,0	0,000	23
**H6548**	chr1	152759629-152759630	A	T	CAA->CAT	**KPRP**	Gln14His	rs17612167	0,223	48	13	27,1	159	0	0,0	0,000	0
**H6548**	chr19	10553955-10553956	A	G	TCC->CCC	**KRI1**	Ser328Pro; Ser705Pro; Ser709Pro	rs3087689	0,312	144	43	29,9	126	1	0,8	0,000	0
**H6548**	chr17	41518388-41518389	T	C	ACC->GCC; CAA->CAG	**KRT15**	Thr147Ala; Gln9Gln	rs1050784	0,688	71	23	32,4	177	0	0,0	0,000	45
**H6548**	chr17	40780406-40780407	C	T	GGT->AGT	**KRT27**	Gly193Ser	rs17558560	0,403	101	29	28,7	174	0	0,0	0,000	0
**H6548**	chr12	52795911-52795912	C	G	GGC->GCC	**KRT3**	Gly44Ala	rs28721426	0,147	237	79	33,3	183	0	0,0	0,000	26

**H6548**	chr17	41467110-41467111	T	C	CAG->CGG	**KRT32**	Gln72Arg	rs3744786	0,349	136	106	77,9	140	0	0,0	0,000	0
**H6548**	chr12	52473475-52473476	C	T	GGA->AGA	**KRT6C**	Gly88Arg	rs411107	0,241	38	11	29,0	39	0	0,0	0,006	41
**H6548**	chr12	52552713-52552714	C	T	GAG->AAG	**KRT71**	Glu122Lys	rs665470	0,210	215	62	28,8	282	1	0,4	0,000	17
**H6548**	chr12	52587842-52587843	A	T	GAT->GAA	**KRT72**	Asp366Glu; Asp324Glu; Asp178Glu	rs7310138	0,184	261	86	33,0	263	0	0,0	0,000	0
**H6548**	chr12	52848794-52848795	C	T	GGG->AGG	**KRT78**	Gly46Arg	rs2253798	0,098	60	22	36,7	86	0	0,0	0,000	0
**H6548**	chr12	52823165-52823166	T	C	AAG->AGG	**KRT79**	Lys177Arg; Lys406Arg	rs73102423	0,040	144	42	29,2	225	0	0,0	0,000	0
**H6548**	chr12	52291311-52291312	C	G	GGA->CGA	**KRT81**	Gly52Arg	rs2071588	0,359	219	111	50,7	72	0	0,0	-2,000	48
**H6548**	chr21	44550876-44550877	C	-A^CTTGCAGCAGACAGG		**KRTAP10-2**	Lys194		0,000	307	79	25,7	312	0	0,0	0,000	0
**H6548**	chr21	44558708-44558709	T	C	ACG->GCG	**KRTAP10-3**	Thr3Ala	rs452472	0,285	378	258	68,3	283	0	0,0	0,000	0
**H6548**	chr21	44580489-44580490	G	T	CCC->CAC	**KRTAP10-5**	Pro30His	rs149326833	0,029	417	129	30,9	307	0	0,0	0,000	0
**H6548**	chr21	44591805-44591806	C	T	GCC->ACC	**KRTAP10-6**	Ala227Thr	rs58757665	0,068	305	104	34,1	230	0	0,0	0,000	0
**H6548**	chr21	44601419-44601420	G	A	GTG->ATG	**KRTAP10-7**	Val267Met	rs75428839	0,046	426	132	31,0	379	0	0,0	0,000	0
**H6548**	chr17	41034700-41034701	G	C	CAG->GAG	**KRTAP1-3**	Gln41Glu	rs78298475	1,000	106	33	31,1	190	18	9,5	0,000	0

**H6548**	chr21	30430449-30430450	G	A	GCC->ACC	**KRTAP13-4**	Ala59Thr	rs2226548	0,569	103	53	51,5	205	0	0,0	0,000	0
**H6548**	chr21	30440453-30440454	C	A	CTG->ATG	**KRTAP15-1**	Leu43Met	rs2832873	0,569	107	63	58,9	217	0	0,0	0,000	0
**H6548**	chr21	30496967-30496968	T	C	TAC->TGC	**KRTAP19-4**	Tyr48Cys	rs2298437	0,585	101	49	48,5	214	0	0,0	0,000	0
**H6548**	chr21	30541662-30541663	G	-A^G		**KRTAP19-6**	Phe57	rs5843453	0,730	126	73	57,9	244	0	0,0	0,000	0
**H6548**	chr17	41118194-41118195	G	A	CGC->TGC	**KRTAP4-11**	Arg41Cys	rs182578725	0,000	525	191	36,4	393	0	0,0	0,000	0
**H6548**	chr17	41149703-41149704	G	A	CGC->TGC	**KRTAP4-5**	Arg22Cys	rs1497383	0,477	574	377	65,7	407	2	0,5	0,000	0
**H6548**	chr17	41084408-41084409	C	G	ACC->AGC	**KRTAP4-7**	Thr68Ser	rs11650484	0,617	302	202	66,9	268	1	0,4	0,000	0
**H6548**	chr17	41105680-41105681	C	T	GCG->GTG	**KRTAP4-9**	Ala98Val	rs7207685	0,673	257	160	62,3	200	2	1,0	0,000	0
**H6548**	chr11	1607717-1607718	G	A	TCC->TTC	**KRTAP5-3**	Ser223Phe	rs117085626	0,062	176	50	28,4	360	0	0,0	0,000	0
**H6548**	chr11	1630355-1630356	C	-C^CTGCTGCCAGTCCAGCTG		**KRTAP5-5**	Ser172		0,000	100	34	34,0	98	1	1,0	0,000	0
**H6548**	chr11	71527406-71527407	G	C	TGT->TCT	**KRTAP5-7**	Cys36Ser	rs147685834	0,221	235	107	45,5	148	0	0,0	0,000	0
**H6548**	chr11	71548944-71548945	C	G	TGC->TGG	**KRTAP5-9**	Cys96Trp	rs200091258	0,002	217	78	35,9	333	0	0,0	0,000	0
**H6548**	chr19	18568568-18568569	C	G	CCT->GCT	**KXD1**	Pro157Ala	rs7648	0,526	163	54	33,1	149	0	0,0	0,000	0

**H6548**	chr2	143040619-143040620	A	G	AAA->GAA	**KYNU**	Lys412Glu	rs9013	0,166	84	21	25,0	157	0	0,0	0,000	22
**H6548**	chr19	54510566-54510567	T	A	TGA->AGA; TGA->AGA	**LAIR2**	*153Arg; *136Arg; His121Gln	rs62131570	0,042	28	11	39,3	51	0	0,0	0,000	0
**H6548**	chr6	129250184-129250185	G	A	CGT->CAT	**LAMA2**	Arg619His	rs3816665	0,186	136	37	27,2	163	0	0,0	0,000	13
**H6548**	chr1	209626884-209626885	C	T	GTG->ATG	**LAMB3**	Val527Met	rs2076349	0,133	181	47	26,0	106	0	0,0	0,000	27
**H6548**	chr9	131087620-131087621	G	A	CGG->CAG	**LAMC3**	Arg1459Gln; Arg147Gln	rs4740412	0,228	204	66	32,4	208	0	0,0	0,000	21
**H6548**	chr17	38914404-38914405	A	G	CAC->CGC; TCA->TCG	**LASP1**	His24Arg; Ser146Ser; Ser90Ser	rs525989	0,646	114	82	71,9	79	0	0,0	-2,000	74
**H6548**	chr1	152805431-152805432	C	T	TGC->TAC	**LCE1C**	Cys16Tyr	rs2006940	0,169	219	58	26,5	197	0	0,0	0,000	21
**H6548**	chr1	152787512-152787513	G	A	GGA->AGA	**LCE1E**	Gly72Arg	rs148840667	0,005	215	77	35,8	209	1	0,5	0,000	0
**H6548**	chr1	152776614-152776615	C	T	CGG->TGG	**LCE1F**	Arg82Trp	rs41268480	0,166	314	103	32,8	217	0	0,0	0,000	0
**H6548**	chr1	152511652-152511653	G	A	TGT->TAT	**LCE5A**	Cys40Tyr	rs2105117	0,607	203	140	69,0	259	0	0,0	0,000	0
**H6548**	chr15	43330066-43330067	C	A	AGG->AGT	**LCMT2**	Arg141Ser	rs3742970	0,084	471	144	30,6	293	0	0,0	0,000	0
**H6548**	chr9	136984717-136984718	G	A	GCC->ACC	**LCNL1**	Ala68Thr	rs17578859	0,289	288	97	33,7	241	0	0,0	0,000	0
**H6548**	chr13	46130934-46130935	G	C	CCG->GCG	**LCP1**	Pro113Ala; Pro141Ala; Pro544Ala	rs17067725	0,002	130	37	28,5	202	0	0,0	0,000	30

**H6548**	chr1	65610268-65610269	G	C	AAG->AAC	**LEPR**	Lys656Asn	rs1805094	0,157	52	13	25,0	129	0	0,0	0,000	3
**H6548**	chr12	6839303-6839304	T	C	ATA->ACA	**LEPREL2**	Ile685Thr; Ile492Thr	rs1129649	0,394	67	19	28,4	96	0	0,0	0,000	0
**H6548**	chr4	1841596-1841597	C	T	CGC->CAC	**LETM1**	Arg115His	rs116753949	0,043	75	22	29,3	162	0	0,0	0,000	21
**H6548**	chr19	39707283-39707284	T	C	TGT->CGT	**LGALS14**	Cys96Arg; Cys67Arg	rs4830	0,642	76	26	34,2	169	0	0,0	0,000	0
**H6548**	chr14	55145120-55145121	A	T	CAA->CAT	**LGALS3**	Gln201His	rs11125	0,066	116	42	36,2	239	0	0,0	0,000	47
**H6548**	chr1	236543561-236543562	G	C	AGG->AGC	**LGALS8**	Arg226Ser; Arg184Ser; Arg167Ser	rs2243525	0,717	123	49	39,8	271	0	0,0	0,000	40
**H6548**	chr1	236524481-236524482	A	G	CTG->CCG	**LGALS8-AS1**	Leu6Pro	rs3820564	0,085	163	51	31,3	231	0	0,0	0,000	0
**H6548**	chr4	25030512-25030513	C	T	GGC->AGC	**LGI2**	Gly61Ser	rs74901868	0,251	244	76	31,2	178	0	0,0	0,000	13
**H6548**	chr1	202214179-202214180	C	T	TCA->TTA	**LGR6**	Ser9Leu	rs10920362	0,496	320	214	66,9	143	0	0,0	0,000	40
**H6548**	chr1	180274388-180274389	A	G	AAT->AGT	**LHX4**	Asn328Ser	rs7536561	0,483	108	53	49,1	248	0	0,0	0,000	12
**H6548**	chr19	54239947-54239948	T	G	CAC->CCC	**LILRA6**	His282Pro; His421Pro; His322Pro	rs199826565	0,126	206	72	35,0	127	1	0,8	0,000	0
**H6548**	chr19	54631631-54631632	C	T	CCC->CTC	**LILRB1**	Pro68Leu	rs1061679	0,605	170	65	38,2	471	1	0,2	0,000	30
**H6548**	chr22	31225805-31225806	G	A	GGC->AGC	**LIMK2**	Gly35Ser	rs5997917	0,165	104	46	44,2	222	0	0,0	0,000	33

**H6548**	chr6	33593118-33593119	A	G	CTG->CCG	**LINC00336**	Leu73Pro	rs210162	0,344	150	59	39,3	138	0	0,0	0,000	0
**H6548**	chr17	81305009-81305010	G	A	CGT->TGT	**LINC00482**	Arg119Cys	rs2048058	0,102	190	68	35,8	163	0	0,0	0,000	0
**H6548**	chr12	90946475-90946476	T	-C^T		**LINC00615**	Phe37	rs397849978	0,007	78	20	25,6	55	0	0,0	0,000	0
**H6548**	chr1	3067398-3067399	G	A	GCC->GTC	**LINC00982**	Ala68Val	rs2297828	0,279	338	89	26,3	263	0	0,0	0,000	0
**H6548**	chr15	58541793-58541794	G	A	GTG->ATG	**LIPC**	Val95Met	rs6078	0,077	97	39	40,2	74	0	0,0	-2,000	8
**H6548**	chr10	88669894-88669895	A	G	ACT->GCT	**LIPF**	Thr138Ala;<br>Thr171Ala;<br>Thr128Ala;	rs814628	0,176	94	27	28,7	280	0	0,0	0,000	9
**H6548**	chr3	197975041-197975042	G	T	GAG->GAT	**LMLN**	Glu54Asp;<br>Glu106Asp	rs7373165	0,138	74	20	27,0	103	0	0,0	0,000	13
**H6548**	chr10	47922812-47922813	G	A	GTG->ATG	**LOC100996758**	Val276Met	rs79871698	0,141	90	24	26,7	113	0	0,0	0,000	0
**H6548**	chr2	131517829-131517830	C	A	CTG->ATG	**LOC150776**	Leu168Met	rs2277877	0,058	222	58	26,1	281	0	0,0	0,000	0
**H6548**	chr16	74332523-74332524	A	T	TGT->AGT	**LOC283922**	Cys316Ser	rs560368989	0,002	35	9	25,7	120	0	0,0	0,000	0
**H6548**	chr2	87049687-87049688	T	C	GAT->GGT	**LOC285074**	Asp60Gly;<br>Asp21Gly	rs34148650	0,392	40	13	32,5	48	0	0,0	0,001	0
**H6548**	chr11	49576654-49576655	T	C	TTC->TCC	**LOC440040**	Phe107Ser	rs3750925	0,530	169	44	26,0	480	0	0,0	0,000	0
**H6548**	chr15	94855885-94855886	G	A	GGT->GAT	**LOC440311**	Gly138Asp	rs28639753	0,082	274	92	33,6	206	2	1,0	0,000	0

**H6548**	chr12	9294464-9294465	C	T	CGC->TGC	**LOC642846**	Arg117Cys	rs200332681	0,003	76	29	38,2	97	4	4,1	0,000	0
**H6548**	chr18	46483634-46483635	G	A	GCC->GTC	**LOXHD1**	Ala337Val; Ala891Val; Ala987Val;	rs1377016	0,332	76	35	46,1	151	0	0,0	0,000	8
**H6548**	chr10	98261122-98261123	C	T	CGG->CAG	**LOXL4**	Arg154Gln	rs33995374	0,117	170	50	29,4	181	1	0,6	0,000	47
**H6548**	chr20	41357444-41357445	A	C	CAA->CAC	**LPIN3**	Gln679His; Gln680His	rs12625565	0,292	93	49	52,7	164	0	0,0	0,000	11
**H6548**	chr19	11362681-11362682	C	G	ACC->AGC	**LPPR2**	Thr253Ser; Thr278Ser; Thr85Ser	rs11540666	0,070	274	87	31,8	167	0	0,0	0,000	0
**H6548**	chr11	66858762-66858763	T	C	GTG->GCG	**LRFN4**	Val340Ala	rs3741194	0,217	351	109	31,1	251	0	0,0	0,000	0
**H6548**	chr4	109869754-109869755	A	T	ATG->TTG	**LRIT3**	Met336Leu; Met153Leu	rs764205	0,563	83	56	67,5	115	0	0,0	0,000	0
**H6548**	chr12	25090180-25090181	G	C	TGT->TCT	**LRMP**	Cys197Ser; Cys144Ser	rs1908946	0,537	72	36	50,0	94	0	0,0	0,000	44
**H6548**	chr2	140485348-140485349	T	C	CAA->CGA	**LRP1B**	Gln3140Arg	rs34488772	0,047	62	17	27,4	118	0	0,0	0,000	56
**H6548**	chr1	53326978-53326979	A	C	GAT->GAG	**LRP8**	Asp46Glu	rs3820198	0,418	146	57	39,0	169	0	0,0	0,000	14
**H6548**	chr10	48914063-48914064	C	T	CGC->CAC	**LRRC18**	Arg31His	rs17772611	0,119	75	24	32,0	129	0	0,0	0,000	0
**H6548**	chr10	27249639-27249640	G	A	CCT->CTT	**LRRC37A6P**	Pro275Leu	rs11015624	0,177	109	32	29,4	223	0	0,0	0,000	0
**H6548**	chr17	30631960-30631961	C	A	CTC->ATC	**LRRC37BP1**	Leu55Ile	rs200334483	0,001	148	41	27,7	240	1	0,4	0,000	0

**H6548**	chr15	71012885-71012886	G	A	CGT->CAT	**LRRC49**	Arg549His; Arg515His; Arg564His;	rs150157186	0,003	109	33	30,3	181	0	0,0	0,000	12
**H6548**	chr1	89713972-89713973	A	G	AAT->AGT	**LRRC8C**	Asn468Ser	rs12032393	0,157	65	30	46,2	102	0	0,0	0,000	18
**H6548**	chr1	204617918-204617919	G	A	CCA->TCA	**LRRN2**	Pro692Ser	rs11588857	0,198	66	28	42,4	116	0	0,0	0,000	0
**H6548**	chr20	6052378-6052379	T	C	ACC->GCC	**LRRN4**	Thr141Ala	rs1884643	0,378	267	125	46,8	103	0	0,0	0,000	0
**H6548**	chr2	80302830-80302831	T	C	AAC->AGC	**LRRTM1**	Asn330Ser	rs6733871	0,273	280	88	31,4	291	0	0,0	0,000	4
**H6548**	chr12	1831347-1831348	G	A	GAC->AAC	**LRTM2**	Asp161Asn	rs41276696	0,041	140	37	26,4	221	0	0,0	0,000	0
**H6548**	chr14	74555567-74555568	G	T	CCA->CAA	**LTBP2**	Pro319Gln	rs2304707	0,066	88	26	29,6	153	0	0,0	0,000	22
**H6548**	chr21	28969568-28969569	A	G	TTA->TCA	**LTN1**	Leu449Ser	rs2254796	0,597	54	18	33,3	84	0	0,0	0,000	2
**H6548**	chr19	13101028-13101029	C	T	CGA->CAA	**LYL1**	Arg48Gln	rs117072928	0,079	169	44	26,0	60	0	0,0	-2,000	77
**H6548**	chr20	14085629-14085630	C	T	ACT->ATT	**MACROD2**	Thr58Ile	rs2990505	0,238	36	9	25,0	61	0	0,0	0,008	31
**H6548**	chrX	153182482-153182483	A	G	ACC->GCC	**MAGEA1**	Thr32Ala		0,000	69	34	49,3	177	0	0,0	0,000	87
**H6548**	chrX	151924180-151924181	G	A	GCC->ACC	**MAGEA4**	Ala173Thr; Ala201Thr	rs1047251	0,520	44	18	40,9	123	0	0,0	0,000	84
**H6548**	chrX	152767127-152767128	C	A	GTG->TTG	**MAGEA6**	Val175Leu		0,000	101	28	27,7	179	0	0,0	0,000	0

**H6548**	chrX	27821499-27821500	G	A	CGG->CAG	**MAGEB10**	Arg65Gln	rs12557898	0,379	62	43	69,4	73	0	0,0	0,000	0
**H6548**	chrX	141906230-141906231	T	C	TTC->TCC	**MAGEC1**	Phe276Ser	rs75148863	0,054	50	23	46,0	85	0	0,0	0,000	83
**H6548**	chr10	19531325-19531326	A	T	GAT->GTT	**MALRD1**	Asp1818Val; Asp12Val	rs41276112	0,024	71	19	26,8	119	1	0,8	0,000	0
**H6548**	chr9	137086548-137086549	T	A	ACC->TCC	**MAN1B1-AS1**	Thr54Ser	rs144872844	0,005	122	32	26,2	73	0	0,0	-2,000	0
**H6548**	chr15	90916244-90916245	G	A	GTG->ATG	**MAN2A2**	Val995Met; Val657Met; Val574Met	rs12909056	0,099	114	31	27,2	152	0	0,0	0,000	28
**H6548**	chr6	95586967-95586968	C	T	CGC->TGC	**MANEA**	Arg177Cys	rs62619206	0,037	49	13	26,5	153	0	0,0	0,000	19
**H6548**	chr15	43525880-43525881	G	A	GCC->ACC	**MAP1A**	Ala1470Thr	rs62020612	0,166	86	25	29,1	105	0	0,0	0,000	19
**H6548**	chr3	47916505-47916506	C	G	GAA->CAA	**MAP4**	Glu441Gln; Glu418Gln; Glu458Gln	rs2230169	0,050	98	25	25,5	207	0	0,0	0,000	16
**H6548**	chr11	75667628-75667629	A	C	ATT->ATG	**MAP6**	Ile247Met	rs12225010	0,549	55	20	36,4	30	0	0,0	0,004	11
**H6548**	chr6	136361033-136361034	G	A	CGG->TGG	**MAP7**	Arg543Trp; Arg580Trp; Arg588Trp;	rs2076190	0,827	149	60	40,3	79	0	0,0	-2,000	25
**H6548**	chrX	20052927-20052928	T	C	AAT->AGT	**MAP7D2**	Asn75Ser; Asn138Ser; Asn182Ser;	rs34519770	0,283	116	74	63,8	110	0	0,0	0,000	0
**H6548**	chrX	136230874-136230875	T	G	GAA->GCA	**MAP7D3**	Glu467Ala; Glu502Ala; Glu484Ala;	rs1055497	0,555	87	54	62,1	154	0	0,0	0,000	0
**H6548**	chr22	50255128-50255129	C	T	TGG->TGA	**MAPK12**	Trp183*	rs74798979	0,029	140	116	82,9	133	0	0,0	0,000	54

**H6548**	chr17	45990015-45990016	T	C	TAC->CAC	**MAPT**	Tyr441His	rs2258689	0,277	36	21	58,3	48	0	0,0	0,000	8
**H6548**	chr19	8421999-8422000	G	A	GCC->ACC	**MARCH2**	Ala54Thr	rs1133893	0,271	165	45	27,3	185	1	0,5	0,000	20
**H6548**	chr2	159748424-159748425	G	A	GGT->AGT	**MARCH7**	Gly379Ser; Gly323Ser; Gly341Ser	rs13024801	0,068	69	25	36,2	129	0	0,0	0,000	25
**H6548**	chr10	45463432-45463433	A	C	TTG->TGG	**MARCH8**	Leu269Trp	rs2291429	0,234	234	73	31,2	274	2	0,7	0,000	24
**H6548**	chr16	71626500-71626501	G	A	CGG->CAG	**MARVELD3**	Arg91Gln	rs61753635	0,231	208	92	44,2	98	0	0,0	0,000	35
**H6548**	chr1	46035686-46035687	A	G	AAG->AGG	**MAST2**	Lys1673Arg; Lys1672Arg	rs34070850	0,053	101	26	25,7	245	0	0,0	0,000	24
**H6548**	chr10	52771481-52771482	G	A	CGT->TGT	**MBL2**	Arg52Cys	rs5030737	0,056	31	9	29,0	50	0	0,0	0,002	15
**H6548**	chr7	100127522-100127523	C	G	TCC->TGC	**MBLAC1**	Ser43Cys	rs78225095	0,062	129	38	29,5	74	0	0,0	-2,000	0
**H6548**	chr5	90473829-90473830	G	A	CCC->TCC	**MBLAC2**	Pro155Ser	rs3763072	0,451	466	171	36,7	501	0	0,0	0,000	0
**H6548**	chr6	31529137-31529138	G	A	GAG->AAG	**MCCD1**	Glu42Lys	rs2259435	0,181	116	36	31,0	133	1	0,8	0,000	0
**H6548**	chr3	183180131-183180132	A	G	ATG->ACG	**MCF2L2**	Met1015Thr	rs35070271	0,182	75	25	33,3	72	0	0,0	0,000	0
**H6548**	chr8	66874581-66874582	C	T	ACG->ATG	**MCMDC2**	Thr94Met; Thr25Met	rs116671744	0,040	36	10	27,8	88	0	0,0	0,000	0
**H6548**	chr8	6643022-6643023	C	T	CCT->TCT	**MCPH1**	Pro828Ser	rs1057091	0,290	39	12	30,8	83	0	0,0	0,000	29

**H6548**	chr6	30708219-30708220	C	T	GGA->AGA	**MDC1**	Gly787Arg	rs148637924	0,001	95	26	27,4	219	0	0,0	0,000	61
**H6548**	chr3	151372635-151372636	G	A	CGA->CAA	**MED12L**	Arg1210Gln; Arg1070Gln; Arg373Gln	rs3732765	0,302	96	47	49,0	175	0	0,0	0,000	0
**H6548**	chr16	3243921-3243922	A	T	GAT->GAA	**MEFV**	Asp424Glu	rs1231123	0,391	85	24	28,2	167	0	0,0	0,000	10
**H6548**	chr1	3514626-3514627	C	T	TGC->TAC	**MEGF6**	Cys154Tyr; Cys259Tyr	rs61746170	0,001	166	53	31,9	88	0	0,0	-2,000	0
**H6548**	chr10	14966493-14966494	A	C	AAA->ACA	**MEIG1**	Lys9Thr	rs4750568	0,658	56	17	30,4	89	0	0,0	0,000	8
**H6548**	chr2	66568773-66568774	T	C	TGG->CGG	**MEIS1**	Trp378Arg	rs112723259	0,027	157	49	31,2	253	0	0,0	0,000	72
**H6548**	chr17	15787613-15787614	G	C	GAG->GAC	**MEIS3P1**	Glu248Asp	rs533242	0,130	192	65	33,9	269	0	0,0	0,000	0
**H6548**	chr15	89751074-89751075	C	G	GCG->CCG	**MESP1**	Ala53Pro	rs6496598	0,516	166	81	48,8	116	1	0,9	0,000	13
**H6548**	chr11	62666700-62666701	G	A	GGC->AGC	**METTL12**	Gly125Ser	rs11231181	0,232	121	33	27,3	237	0	0,0	0,000	0
**H6548**	chr7	142052414-142052415	A	C	GAA->GCA	**MGAM**	Glu976Ala	rs116536012	0,049	25	11	44,0	44	0	0,0	0,000	11
**H6548**	chr17	76882176-76882177	G	A	GTC->ATC	**MGAT5B**	Val70Ile; Val81Ile	rs571264	0,267	245	83	33,9	152	0	0,0	0,000	30
**H6548**	chr10	129766905-129766906	A	G	AAG->AGG	**MGMT**	Lys209Arg	rs2308327	0,093	184	65	35,3	204	0	0,0	0,000	86
**H6548**	chr12	14882146-14882147	T	C	ACC->GCC	**MGP**	Thr127Ala; Thr102Ala	rs4236	0,380	111	34	30,6	141	0	0,0	0,000	11

**H6548**	chr6	31412153-31412154	G	A	CGA->CAA	**MICA**	Arg177Gln; Arg136Gln; Arg274Gln	rs1063635	0,503	51	20	39,2	154	0	0,0	0,000	15
**H6548**	chr1	12022276-12022277	A	T	AAA->AAT	**MIIP**	Lys99Asn	rs11553925	0,202	172	63	36,6	204	0	0,0	0,000	53
**H6548**	chr22	46097971-46097972	C	T	CGT->TGT	**MIRLET7BHG**	Arg16Cys	rs3747243	0,287	56	19	33,9	77	0	0,0	0,000	0
**H6548**	chr10	128113591-128113592	C	G	GAG->GAC	**MKI67**	Glu497Asp; Glu137Asp; Glu72Asp	rs11016076	0,190	56	20	35,7	85	0	0,0	0,000	76
**H6548**	chr19	2041032-2041033	T	C	ACC->GCC	**MKNK2**	Thr117Ala	rs2287003	0,611	246	87	35,4	170	0	0,0	0,000	57
**H6548**	chr14	75047124-75047125	G	A	CCT->CTT	**MLH3**	Pro844Leu	rs175080	0,411	76	24	31,6	159	0	0,0	0,000	55
**H6548**	chr12	122133441-122133442	A	G	GAG->GGG	**MLXIP**	Glu396Gly; Glu147Gly; Glu3Gly	rs7978353	0,428	93	26	28,0	123	0	0,0	0,000	36
**H6548**	chr4	145655265-145655266	G	C	CAG->CAC	**MMAA**	Gln363His	rs2270655	0,066	92	24	26,1	142	0	0,0	0,000	1
**H6548**	chr1	2595306-2595307	A	G	ATG->ACG	**MMEL1**	Met518Thr	rs3748816	0,419	246	83	33,7	265	1	0,4	0,000	11
**H6548**	chr11	102779657-102779658	C	T	GGA->AGA	**MMP10**	Gly65Arg	rs17293607	0,113	108	55	50,9	137	0	0,0	0,000	36
**H6548**	chr12	131840693-131840694	G	A	GCC->ACC	**MMP17**	Ala182Thr; Ala98Thr	rs6598163	0,474	636	171	26,9	462	1	0,2	0,000	47
**H6548**	chr11	102530679-102530680	A	C	TGT->TGG	**MMP7**	Cys7Trp	rs55701929	0,014	164	42	25,6	238	1	0,4	0,000	61
**H6548**	chr4	89935786-89935787	A	-T^AG		**MMRN1**	Arg703; Arg445		0,000	93	27	29,0	185	0	0,0	0,000	18

**H6548**	chr6	29666225-29666226	G	C	GTT->CTT	**MOG**	Val171Leu; Val55Leu; Val101Leu	rs2857766	0,220	64	17	26,6	78	0	0,0	0,000	11
**H6548**	chr2	222694369-222694370	T	C	TCA->CCA	**MOGAT1**	Ser163Pro; Ser123Pro	rs1868024	0,214	58	27	46,6	101	0	0,0	0,000	9
**H6548**	chr13	19650061-19650062	G	T	GAT->TAT	**MPHOSPH8**	Asp460Tyr; Asp157Tyr	rs75390100	0,034	72	21	29,2	95	0	0,0	0,000	0
**H6548**	chr19	18193889-18193890	A	G	ATG->GTG	**MPV17L2**	Met72Val; Met8Val	rs874628	0,232	84	23	27,4	161	0	0,0	0,000	0
**H6548**	chr11	3222136-3222137	G	T	GGG->GTG	**MRGPRG-AS1**	Gly142Val	rs12280457	0,644	45	37	82,2	41	0	0,0	0,000	0
**H6548**	chr18	50819653-50819654	G	T	ACC->AAC	**MRO**	Thr23Asn	rs3813089	0,176	76	26	34,2	203	0	0,0	0,000	9
**H6548**	chr8	144239148-144239149	G	A	GAC->AAC	**MROH1**	Asp521Asn		0,000	133	41	30,8	74	0	0,0	-2,000	0
**H6548**	chr2	233823664-233823665	G	A	GTG->ATG	**MROH2A**	Val1380Met	rs17868361	0,143	207	66	31,9	154	0	0,0	0,000	0
**H6548**	chr8	141466510-141466511	G	A	CCG->CTG	**MROH5**	Pro792Leu	rs28504323	0,162	205	54	26,3	200	0	0,0	0,000	0
**H6548**	chr1	54653470-54653471	A	G	CAT->CGT	**MROH7**	His182Arg	rs11206407	0,325	88	22	25,0	164	0	0,0	0,000	0
**H6548**	chr20	37141243-37141244	G	T	ACC->AAC	**MROH8**	Thr469Asn; Thr434Asn	rs1780680	0,446	67	31	46,3	148	0	0,0	0,000	0
**H6548**	chr11	68892651-68892652	C	G	CGC->CCC	**MRPL21**	Arg264Pro	rs1611798	0,500	54	26	48,2	28	0	0,0	0,000	0
**H6548**	chr6	24423595-24423596	C	T	CCT->TCT	**MRS2**	Pro415Ser; Pro412Ser; Pro362Ser	rs35261004	0,013	54	22	40,7	97	0	0,0	0,000	5

**H6548**	chr11	10626447-10626448	G	A	CTC->TTC	**MRVI1**	Leu288Phe; Leu296Phe	rs35468145	0,038	193	65	33,7	218	0	0,0	0,000	25
**H6548**	chr11	60763790-60763791	A	G	AGT->GGT	**MS4A15**	Ser20Gly	rs12363342	0,333	41	17	41,5	68	0	0,0	0,000	0
**H6548**	chr11	60292336-60292337	A	G	AAA->GAA	**MS4A4A**	Lys33Glu; Lys52Glu	rs10750931	0,144	99	34	34,3	198	0	0,0	0,000	18
**H6548**	chr11	60179778-60179779	C	T	GCC->ACC	**MS4A6A**	Ala112Thr	rs583791	0,579	41	23	56,1	87	0	0,0	0,000	11
**H6548**	chr2	47512411-47512412	A	G	CAG->CGG	**MSH2**	Gln915Arg	rs2303424	0,461	67	30	44,8	119	0	0,0	0,000	81
**H6548**	chr2	47800509-47800510	A	G	ATA->GTA	**MSH6**	Ile843Val; Ile541Val; Ile713Val		0,000	55	15	27,3	151	0	0,0	0,000	79
**H6548**	chr2	233867028-233867029	G	T	GCC->GAC	**MSL3P1**	Ala56Asp	rs213544	0,070	221	56	25,3	410	1	0,2	0,000	0
**H6548**	chr18	8784371-8784372	A	T	GAA->GAT	**MTCL1**	Glu258Asp; Glu420Asp	rs918272	0,008	484	158	32,6	369	0	0,0	0,000	0
**H6548**	chr16	86532275-86532276	G	C	TCC->TGC	**MTHFSD**	Ser133Cys; Ser295Cys; Ser296Cys;	rs3751802	0,227	355	117	33,0	207	0	0,0	0,000	0
**H6548**	chr1	149930849-149930850	G	A	CCT->CTT	**MTMR11**	Pro397Leu; Pro469Leu	rs376116759	0,000	68	18	26,5	63	0	0,0	0,000	0
**H6548**	chr6	152994559-152994560	C	T	GTC->ATC	**MTRF1L**	Val214Ile; Val178Ile; Val72Ile	rs3192723	0,218	118	33	28,0	207	0	0,0	0,000	0
**H6548**	chr4	99594864-99594865	C	G	CAC->CAG	**MTTP**	His297Gln; His324Gln	rs2306985	0,408	110	34	30,9	173	0	0,0	0,000	22
**H6548**	chr8	17723596-17723597	T	C	ATT->GTT	**MTUS1**	Ile842Val	rs73206217	0,044	66	19	28,8	79	0	0,0	0,000	76

**H6548**	chr7	101000745-101000746	C	A	CCT->ACT	**MUC12**	Pro3395Thr	rs140825627	0,136	34	14	41,2	224	0	0,0	0,000	43
**H6548**	chr19	8964785-8964786	G	C	TCA->TGA	**MUC16**	Ser3995*		0,000	103	27	26,2	260	0	0,0	0,000	74
**H6548**	chr7	101034348-101034349	C	T	CCT->CTT	**MUC17**	Pro978Leu	rs78990442	0,097	317	82	25,9	671	0	0,0	0,000	47
**H6548**	chr12	40490863-40490864	C	T	CCT->TCT	**MUC19**	Pro5971Ser		0,000	179	54	30,2	696	0	0,0	0,000	15
**H6548**	chr11	1103986-1103987	C	T	CTC->TTC	**MUC2**	Leu3140Phe		0,000	187	55	29,4	121	0	0,0	0,000	60
**H6548**	chr3	195725933-195725934	T	C	CTC->CCC	**MUC20**	Leu444Pro	rs138659995	0,147	131	69	52,7	216	0	0,0	0,000	31
**H6548**	chr6	31026088-31026089	A	T	ACT->TCT	**MUC22**	Thr220Ser; Thr223Ser	rs62399430	0,117	171	45	26,3	517	0	0,0	0,000	0
**H6548**	chr3	195784028-195784029	G	T	GAC->GAA	**MUC4**	Asp2517Glu	rs201192572	0,085	38	13	34,2	74	0	0,0	0,000	58
**H6548**	chr11	1244195-1244196	T	G	CTG->CGG	**MUC5B**	Leu2439Arg	rs764578318	0,000	123	43	35,0	113	0	0,0	0,000	24
**H6548**	chr9	110695518-110695519	A	G	AGC->GGC	**MUSK**	Ser159Gly	rs35176182	0,197	34	10	29,4	122	0	0,0	0,000	13
**H6548**	chr2	33727702-33727703	G	T	CCC->ACC	**MYADML**	Pro25Thr	rs75672894	0,023	365	116	31,8	395	0	0,0	0,000	0
**H6548**	chr17	81941056-81941057	G	A	CGG->TGG	**MYADML2**	Arg229Trp	rs62077244	0,042	379	111	29,3	350	0	0,0	0,000	0
**H6548**	chr8	66566195-66566196	T	G	GAA->GAC	**MYBL1**	Glu666Asp	rs61729530	0,012	41	13	31,7	70	0	0,0	0,000	61

**H6548**	chr19	50454330-50454331	G	A	TGG->TAG	**MYBPC2**	Trp659*	rs201432361	0,000	114	34	29,8	189	0	0,0	0,000	0
**H6548**	chr17	10395231-10395232	C	T	ATG->ATA	**MYH8**	Met1621Ile		0,000	53	26	49,1	74	0	0,0	0,000	21
**H6548**	chr22	25835344-25835345	G	C	TGG->TCG	**MYO18B**	Trp1037Ser; Trp918Ser; Trp919Ser;	rs17704912	0,047	73	19	26,0	180	0	0,0	0,000	60
**H6548**	chr2	127637301-127637302	C	T	CCG->CTG	**MYO7B**	Pro323Leu	rs56272236	0,535	162	109	67,3	196	4	2,0	0,000	6
**H6548**	chr19	17205972-17205973	T	C	GTT->GCT	**MYO9B**	Val1693Ala	rs7248508	0,525	322	187	58,1	114	0	0,0	0,000	12
**H6548**	chr11	17720292-17720293	C	T	CCC->TCC	**MYOD1**	Pro171Ser	rs148704904	0,000	555	149	26,9	255	0	0,0	0,000	34
**H6548**	chr10	93478205-93478206	A	G	CTT->CCT	**MYOF**	Leu3Pro	rs3938769	0,791	24	6	25,0	99	0	0,0	0,000	34
**H6548**	chr18	3126812-3126813	A	G	ATT->ACT	**MYOM1**	Ile960Thr; Ile864Thr	rs1071600	0,202	59	15	25,4	129	0	0,0	0,000	4
**H6548**	chr1	24068320-24068321	A	G	ATC->ACC	**MYOM3**	Ile723Thr; Ile1066Thr	rs12145360	0,165	102	30	29,4	122	0	0,0	0,000	0
**H6548**	chr2	131484157-131484158	T	C	CAC->CGC	**MZT2A**	His127Arg	rs75021268	0,105	231	70	30,3	160	0	0,0	0,000	0
**H6548**	chr13	32442905-32442906	T	C	AAT->AGT	**N4BP2L2**	Asn427Ser; Asn457Ser; Asn375Ser;	rs3742318	0,243	150	44	29,3	183	0	0,0	0,000	0
**H6548**	chr21	28883024-28883025	C	A	GCA->TCA	**N6AMT1**	Ala61Ser	rs61735765	0,134	34	9	26,5	73	0	0,0	0,004	21
**H6548**	chr16	3485115-3485116	T	C	GTG->GCG	**NAA60**	Val28Ala	rs4785936	0,916	221	161	72,9	259	0	0,0	0,000	0

**H6548**	chr13	101142965-101142966	A	C	AAT->AAG	**NALCN**	Asn459Lys	rs673213	0,518	71	27	38,0	112	0	0,0	0,000	7
**H6548**	chr15	35084481-35084482	G	A	ACC->ATC	**NANOG**	Thr210Ile	rs9944179	0,140	25	7	28,0	67	0	0,0	0,004	44
**H6548**	chr8	143574806-143574807	G	C	ACG->AGG	**NAPRT1**	Thr578Arg	rs78452615	0,030	241	82	34,0	244	0	0,0	0,000	62
**H6548**	chr3	46995265-46995266	C	G	CGC->GGC	**NBEAL2**	Arg511Gly; Arg477Gly; Arg72Gly	rs11720139	0,419	396	273	68,9	369	1	0,3	0,000	12
**H6548**	chr1	16565697-16565698	A	T	CTG->CAG	**NBPF1**	Leu1000Gln	rs779418724	0,036	77	20	26,0	166	0	0,0	0,000	51
**H6548**	chr1	148122792-148122793	C	T	GAA->AAA	**NBPF11**	Glu93Lys; Glu168Lys; Glu222Lys;	rs200675471	0,095	132	37	28,0	333	0	0,0	0,000	0
**H6548**	chr1	145590938-145590939	T	C	AAA->AGA	**NBPF20**	Lys41Arg		0,000	128	35	27,3	197	0	0,0	0,000	0
**H6548**	chr4	17828366-17828367	G	C	ATG->ATC	**NCAPG**	Met581Ile; Met90Ile	rs3795243	0,129	77	23	29,9	143	0	0,0	0,000	27
**H6548**	chr7	74779321-74779322	A	G	AGC->GGC	**NCF1**	Ser99Gly		0,000	261	71	27,2	360	3	0,8	0,000	13
**H6548**	chr2	132785000-132785001	C	T	GTG->ATG	**NCKAP5**	Val604Met	rs61995744	0,217	117	36	30,8	240	1	0,4	0,000	0
**H6548**	chr9	137205864-137205865	C	G	CGC->GGC	**NDOR1**	Arg30Gly	rs113809617	0,139	295	75	25,4	161	0	0,0	0,000	31
**H6548**	chr19	5893046-5893047	G	A	GCC->GTC	**NDUFA11**	Ala186Val	rs12980262	0,046	186	60	32,3	220	0	0,0	0,000	21
**H6548**	chr15	41396967-41396968	C	A	CGC->CTC	**NDUFAF1**	Arg31Leu	rs3204853	0,197	36	13	36,1	88	0	0,0	0,000	23

**H6548**	chr11	78079608-78079609	G	C	CTA->GTA	**NDUFC2-KCTD14**	Leu46Val	rs8875	0,205	63	22	34,9	46	0	0,0	0,000	0
**H6548**	chr2	151527006-151527007	C	T	GAC->AAC	**NEB**	Asp90Asn; Asp2051Asn; Asp7321Asn;	rs35625617	0,025	48	13	27,1	67	0	0,0	0,000	8
**H6548**	chr10	20850459-20850460	T	C	ATG->GTG	**NEBL**	Met351Val	rs4025981	0,064	41	17	41,5	78	0	0,0	0,000	23
**H6548**	chr16	84001840-84001841	C	G	CTG->GTG	**NECAB2**	Leu353Val; Leu270Val	rs2271298	0,390	143	54	37,8	193	0	0,0	0,000	5
**H6548**	chr3	27291328-27291329	A	G	TTG->TCG	**NEK10**	Leu513Ser	rs10510592	0,263	66	39	59,1	139	0	0,0	0,000	62
**H6548**	chr18	79451763-79451764	T	C	CTG->CCG; TCT->TCC	**NFATC1**	Leu589Pro; Ser617Ser; Ser604Ser;	rs15350	0,196	255	94	36,9	258	0	0,0	0,000	25
**H6548**	chr4	47899458-47899459	G	A	CCG->CTG	**NFXL1**	Pro246Leu	rs12651301	0,353	43	22	51,2	77	0	0,0	0,000	0
**H6548**	chr6	138430491-138430492	G	C	CCT->GCT	**NHSL1**	Pro1285Ala; Pro1289Ala	rs61747081	0,012	171	53	31,0	220	0	0,0	0,000	0
**H6548**	chr14	52042123-52042124	C	A	GAG->GAT	**NID2**	Glu602Asp	rs61747585	0,038	74	22	29,7	181	0	0,0	0,000	39
**H6548**	chr14	50792868-50792869	T	C	GAA->GGA	**NIN**	Glu93Gly; Glu55Gly	rs372334376	0,000	94	24	25,5	212	0	0,0	0,000	12
**H6548**	chr15	22866845-22866846	A	-T^AAGA		**NIPA2**	*361; *342	rs368460716	-1,000	78	24	30,8	136	0	0,0	0,000	3
**H6548**	chr5	36985200-36985201	A	G	AAT->AGT	**NIPBL**	Asn674Ser; Asn553Ser	rs3822471	0,132	86	24	27,9	93	0	0,0	0,000	13,99
**H6548**	chr9	104768870-104768871	G	C	GCT->CCT	**NIPSNAP3B**	Ala94Pro	rs10761084	0,197	46	12	26,1	86	0	0,0	0,000	0

**H6548**	chr3	52488574-52488575	C	T	GCG->GTG	**NISCH**	Ala1028Val; Ala517Val	rs61736838	0,006	218	62	28,4	166	1	0,6	0,000	33
**H6548**	chr20	63248816-63248817	T	G	AAG->CAG	**NKAIN4**	Lys29Gln; Lys91Gln	rs1129659	0,413	174	48	27,6	227	1	0,4	0,000	0
**H6548**	chr16	57026300-57026301	C	T	CCC->CTC	**NLRC5**	Pro453Leu; Pro258Leu	rs9938543	0,090	106	29	27,4	251	0	0,0	0,000	34
**H6548**	chr19	55809296-55809297	G	A	CCG->CTG	**NLRP11**	Pro339Leu; Pro438Leu	rs12461110	0,313	54	15	27,8	129	0	0,0	0,000	0
**H6548**	chr19	54982788-54982789	G	A	AGG->AAG	**NLRP2**	Arg364Lys; Arg341Lys; Arg342Lys;	rs4306647	0,100	198	72	36,4	208	0	0,0	0,000	26
**H6548**	chr19	56027599-56027600	G	A	CGT->CAT	**NLRP5**	Arg456His; Arg437His	rs200829045	0,001	258	72	27,9	271	0	0,0	0,000	6
**H6548**	chr11	119172946-119172947	C	T	CCC->TCC	**NLRX1**	Pro63Ser	rs643423	0,579	87	39	44,8	152	0	0,0	0,000	26
**H6548**	chr5	152395502-152395503	C	G	AGT->ACT	**NMUR2**	Ser298Thr	rs4958535	0,172	83	24	28,9	130	2	1,5	0,000	8
**H6548**	chr16	69742473-69742474	T	A	TAC->TTC	**NOB1**	Tyr366Phe; Tyr306Phe	rs1075935	0,046	172	47	27,3	316	0	0,0	0,000	45
**H6548**	chr10	94346482-94346483	G	C	CCA->CGA	**NOC3L**	Pro444Arg; Pro229Arg	rs11187895	0,080	77	22	28,6	81	0	0,0	0,000	21
**H6548**	chr7	30452620-30452621	C	T	GAG->AAG; CCG->CCA	**NOD1**	Glu266Lys; Pro220Pro	rs2075820	0,280	224	78	34,8	214	0	0,0	0,000	23
**H6548**	chr1	6554474-6554475	G	A	CGG->TGG	**NOL9**	Arg10Trp	rs4908923	0,839	130	72	55,4	83	0	0,0	-2,000	0
**H6548**	chr14	24302078-24302079	G	A	AGT->AAT	**NOP9**	Ser308Asn	rs4280164	0,182	76	19	25,0	141	0	0,0	0,000	0

**H6548**	chr9	136523133-136523134	C	-G^CCA		**NOTCH1**	Gly153		0,000	291	94	32,3	138	1	0,7	0,000	51
**H6548**	chr6	32220862-32220863	T	C	GAC->GGC	**NOTCH4**	Asp272Gly	rs520692	0,304	83	33	39,8	242	0	0,0	0,000	44
**H6548**	chr15	24677082-24677083	C	G	CAG->GAG	**NPAP1**	Gln406Glu	rs3742950	0,404	142	39	27,5	219	0	0,0	0,000	0
**H6548**	chr14	33799960-33799961	G	C	GCG->CCG	**NPAS3**	Ala552Pro; Ala539Pro; Ala522Pro;	rs12434716	0,141	537	160	29,8	423	0	0,0	0,000	10
**H6548**	chr11	108161948-108161949	G	A	CCC->CTC	**NPAT**	Pro102Leu; Pro1046Leu	rs117925274	0,005	55	15	27,3	149	0	0,0	0,000	47
**H6548**	chr1	153689946-153689947	G	A	GAG->AAG	**NPR1**	Glu967Lys; Glu713Lys; Glu445Lys	rs35479618	0,008	201	54	26,9	78	0	0,0	-2,000	2
**H6548**	chr7	25228314-25228315	T	C	GAC->GGC	**NPVF**	Asp42Gly	rs877834	0,232	47	13	27,7	58	0	0,0	0,002	4
**H6548**	chr10	113629606-113629607	G	A	GCC->GTC	**NRAP**	Ala674Val; Ala639Val	rs2286735	0,309	68	23	33,8	149	0	0,0	0,000	13
**H6548**	chr8	143837801-143837802	T	C	GAG->GGG	**NRBP2**	Glu57Gly	rs147343613	0,001	232	78	33,6	145	0	0,0	0,000	25
**H6548**	chr3	196661227-196661228	G	C	GCG->CCG	**NRROS**	Ala529Pro	rs62623661	0,087	196	55	28,1	266	0	0,0	0,000	0
**H6548**	chr1	212791752-212791753	G	A	TCT->TTT	**NSL1**	Ser4Phe	rs17856201	0,157	72	22	30,6	97	0	0,0	0,000	25
**H6548**	chr6	85489524-85489525	T	C	ATG->ACG	**NT5E**	Met379Thr	rs2229524	0,094	53	15	28,3	58	0	0,0	0,000	41
**H6548**	chr19	49056286-49056287	A	C	TGT->TGG	**NTF4**	Cys224Trp	rs185485728	0,012	207	66	31,9	153	0	0,0	0,000	11

**H6548**	chr16	2472349-2472350	G	A	GCC->ACC	**NTN3**	Ala217Thr	rs373454751	-1,000	298	99	33,2	215	1	0,5	0,000	0
**H6548**	chr12	95738116-95738117	A	G	TAC->CAC	**NTN4**	Tyr205His; Tyr168His	rs17288108	0,124	63	16	25,4	120	0	0,0	0,000	53
**H6548**	chr1	156881589-156881590	G	A	CGG->CAG	**NTRK1**	Arg780Gln	rs35669708	0,006	182	65	35,7	81	0	0,0	-2,000	54
**H6548**	chr8	22109790-22109791	C	T	AGG->AAG	**NUDT18**	Arg20Lys	rs7844781	0,447	256	150	58,6	287	0	0,0	0,000	0
**H6548**	chr1	163343748-163343749	C	T	TCG->TTG	**NUF2**	Ser229Leu	rs11802875	0,174	38	13	34,2	45	0	0,0	0,001	38
**H6548**	chr8	28067686-28067687	T	C	AGC->GGC	**NUGGC**	Ser180Gly	rs4732620	0,571	86	44	51,2	184	0	0,0	0,000	0
**H6548**	chr1	229487590-229487591	T	C	CAG->CGG	**NUP133**	Gln406Arg	rs1065674	0,236	36	14	38,9	48	0	0,0	0,000	11
**H6548**	chr11	47840304-47840305	A	G	TGT->CGT	**NUP160**	Cys200Arg	rs74873109	0,083	134	42	31,3	263	0	0,0	0,000	9
**H6548**	chr10	87365147-87365148	C	T	CCG->CTG	**NUTM2D**	Pro488Leu	rs28605659	0,339	44	37	84,1	14	0	0,0	0,000	0
**H6548**	chr9	94320416-94320417	G	A	CCT->TCT	**NUTM2F**	Pro387Ser	rs10993163	0,118	133	46	34,6	175	1	0,6	0,000	0
**H6548**	chr11	114705903-114705904	A	T	AAT->ATT	**NXPE2**	Asn351Ile	rs1356428	0,352	52	19	36,5	94	0	0,0	0,000	0
**H6548**	chr11	114571380-114571381	A	G	TAT->CAT	**NXPE4**	Tyr398His; Tyr114His	rs550897	0,545	73	43	58,9	98	0	0,0	0,000	0
**H6548**	chr12	112948973-112948974	C	G	AGC->AGG	**OAS3**	Ser381Arg	rs2285933	0,268	100	34	34,0	204	0	0,0	0,000	23

**H6548**	chr9	135547945-135547946	G	A	CGC->CAC; CGC->CAC	**OBP2A**	Arg118His; Ala73Ala	rs72766542	0,036	152	52	34,2	217	0	0,0	0,000	0
**H6548**	chr2	219557860-219557861	G	A	CCC->CTC	**OBSL1**	Pro242Leu; Pro150Leu; Pro1251Leu	rs145485683	0,010	253	98	38,7	196	0	0,0	0,000	12
**H6548**	chrX	13753456-13753457	A	G	GAA->GGA	**OFD1**	Glu128Gly	rs3815049	0,338	235	218	92,8	89	0	0,0	-2,000	12
**H6548**	chr1	162023401-162023402	T	C	TAC->TGC	**OLFML2B**	Tyr10Cys	rs12130792	0,077	152	56	36,8	217	0	0,0	0,000	0
**H6548**	chr12	10160848-10160849	C	G	AAG->AAC	**OLR1**	Lys167Asn	rs11053646	0,117	91	27	29,7	121	0	0,0	0,000	15
**H6548**	chr3	193618886-193618887	C	T	GCG->GTG	**OPA1**	Ala210Val; Ala174Val; Ala192Val;	rs34307082	0,022	66	21	31,8	108	0	0,0	0,000	10
**H6548**	chr10	86662358-86662359	C	T	ACC->ATC	**OPN4**	Thr405Ile; Thr394Ile; Thr64Ile	rs1079610	0,730	60	34	56,7	78	0	0,0	0,000	4
**H6548**	chr6	29440535-29440536	C	A	CCG->CAG	**OR10C1**	Pro174Gln	rs2074466	0,159	109	30	27,5	229	1	0,4	0,000	0
**H6548**	chr1	159440093-159440094	G	A	ATG->ATA	**OR10J1**	Met112Ile	rs12118628	0,136	86	26	30,2	204	0	0,0	0,000	0
**H6548**	chr14	20197480-20197481	G	A	GGC->GAC	**OR11G2**	Gly49Asp	rs140668031	0,041	30	10	33,3	73	0	0,0	0,001	0
**H6548**	chr22	15528557-15528558	G	A	GCC->ACC	**OR11H1**	Ala134Thr	rs201956705	0,016	33	11	33,3	115	0	0,0	0,000	0
**H6548**	chr6	29397173-29397174	G	A	GTA->ATA	**OR12D2**	Val159Ile	rs2073151	0,383	70	37	52,9	161	0	0,0	0,000	0
**H6548**	chr9	104605559-104605560	A	T	CTT->CAT	**OR13C2**	Leu23His	rs10991326	0,275	108	37	34,3	160	0	0,0	0,000	0

**H6548**	chr9	104598847-104598848	C	T	TGT->TAT	**OR13C5**	Cys189Tyr	rs4117966	0,269	178	45	25,3	446	0	0,0	0,000	0
**H6548**	chr1	247672476-247672477	C	G	GTA->CTA	**OR13G1**	Val189Leu	rs56096718	0,037	80	25	31,3	212	0	0,0	0,000	0
**H6548**	chr9	35870003-35870004	T	C	CAC->CGC	**OR13J1**	His133Arg	rs7044405	0,620	177	104	58,8	331	0	0,0	0,000	0
**H6548**	chr17	3216472-3216473	C	T	CCT->TCT	**OR1A1**	Pro285Ser	rs769427	0,418	67	18	26,9	135	0	0,0	0,000	6
**H6548**	chr9	122527241-122527242	G	A	CCT->TCT	**OR1N1**	Pro18Ser	rs10818708	0,571	65	23	35,4	100	0	0,0	0,000	0
**H6548**	chr1	247451314-247451315	C	T	GGC->GAC	**OR2B11**	Gly223Asp	rs4925663	0,401	179	48	26,8	287	0	0,0	0,000	0
**H6548**	chr11	6921494-6921495	G	C	TGG->TCG	**OR2D3**	Trp165Ser	rs10839659	0,332	84	32	38,1	136	0	0,0	0,000	0
**H6548**	chr9	111327601-111327602	C	T	GGA->AGA	**OR2K2**	Gly278Arg	rs61748723	0,089	84	23	27,4	158	0	0,0	0,000	0
**H6548**	chr1	248122138-248122139	G	T	GAC->TAC	**OR2M1P**	Asp2Tyr	rs6587422	0,090	79	42	53,2	211	0	0,0	0,000	0
**H6548**	chr1	248324465-248324466	A	G	TTT->CTT	**OR2M7**	Phe35Leu	rs7555424	0,777	164	52	31,7	407	0	0,0	0,000	0
**H6548**	chr1	248406067-248406068	A	G	CAT->CGT	**OR2T1**	His25Arg	rs28599722	0,459	82	25	30,5	217	1	0,5	0,000	0
**H6548**	chr1	248295414-248295415	C	G	AGG->ACG	**OR2T12**	Arg55Thr	rs12745228	0,711	101	26	25,7	221	0	0,0	0,000	0
**H6548**	chr1	248650750-248650751	T	A	AAG->ATG	**OR2T27**	Lys45Met	rs28533004	0,545	104	29	27,9	106	0	0,0	0,000	0

**H6548**	chr1	248362336-248362337	A	-C^A		**OR2T4**	Ile253	rs34079073	0,470	143	47	32,9	349	0	0,0	0,000	0
**H6548**	chr1	248388206-248388207	A	G	TAT->TGT	**OR2T6**	Tyr200Cys	rs768498819	0,000	115	36	31,3	270	0	0,0	0,000	0
**H6548**	chr17	3421515-3421516	A	G	AAG->GAG	**OR3A3**	Lys317Glu	rs227787	0,293	117	30	25,6	223	0	0,0	0,000	0
**H6548**	chr11	55344107-55344108	A	T	AAG->ATG	**OR4A16**	Lys303Met	rs10896659	0,480	44	11	25,0	79	0	0,0	0,000	0
**H6548**	chr11	55572175-55572176	C	T	CAG->TAG	**OR4C16**	Gln17*	rs1459101	0,279	102	33	32,4	152	0	0,0	0,000	0
**H6548**	chr11	54603088-54603089	C	T	GAC->AAC	**OR4C46**	Asp304Asn	rs140000110	0,006	64	24	37,5	62	0	0,0	0,000	0
**H6548**	chr11	55665563-55665564	T	C	ATC->ACC	**OR4C6**	Ile133Thr	rs11230600	0,278	67	23	34,3	165	0	0,0	0,000	0
**H6548**	chr17	58155672-58155673	C	A	CTA->ATA	**OR4D1**	Leu174Ile	rs7218964	0,217	148	39	26,4	308	0	0,0	0,000	0
**H6548**	chr14	21665433-21665434	A	G	ATG->GTG	**OR4E2**	Met118Val	rs2874103	0,244	95	27	28,4	190	0	0,0	0,000	0
**H6548**	chr14	20117424-20117425	C	G	CTC->GTC	**OR4K17**	Leu7Val	rs61743111	0,010	25	7	28,0	58	0	0,0	0,007	0
**H6548**	chr11	55638545-55638546	C	G	TAC->TAG	**OR4P4**	Tyr63*	rs76160133	0,206	91	36	39,6	196	0	0,0	0,000	0
**H6548**	chr14	19747829-19747830	A	G	ACA->GCA	**OR4Q3**	Thr135Ala	rs17210864	0,145	141	69	48,9	436	1	0,2	0,000	0
**H6548**	chr11	4955141-4955142	C	T	TGT->TAT	**OR51A2**	Cys191Tyr	rs147155775	0,005	81	37	45,7	64	0	0,0	0,000	0

**H6548**	chr11	4907390-4907391	G	A	GAA->AAA	**OR51A7**	Glu8Lys	rs11034596	0,132	28	9	32,1	71	1	1,4	0,000	0
**H6548**	chr11	5343511-5343512	C	T	GGC->AGC	**OR51B5**	Gly5Ser	rs11036913	0,396	62	32	51,6	106	0	0,0	0,000	0
**H6548**	chr11	4923998-4923999	G	A	TCA->TTA	**OR51G1**	Ser114Leu	rs10836954	0,064	97	47	48,5	184	0	0,0	0,000	0
**H6548**	chr11	5454274-5454275	C	T	CGC->TGC	**OR51I2**	Arg263Cys	rs75620804	0,017	49	30	61,2	162	0	0,0	0,000	0
**H6548**	chr11	5131726-5131727	T	C	ATT->GTT	**OR52A5**	Ile306Val	rs74051326	0,014	71	24	33,8	147	0	0,0	0,000	0
**H6548**	chr11	4368174-4368175	G	-A^G		**OR52B4**	Leu41	rs11310407	0,354	116	35	30,2	195	0	0,0	0,000	0
**H6548**	chr11	5581698-5581699	C	A	CTT->ATT	**OR52B6**	Leu275Ile	rs74053516	0,034	98	33	33,7	170	0	0,0	0,000	0
**H6548**	chr11	5058837-5058838	G	A	CGC->TGC	**OR52E2**	Arg264Cys	rs2500052	0,500	31	8	25,8	76	0	0,0	0,005	0
**H6548**	chr11	5884974-5884975	G	A	CGC->CAC	**OR52E4**	Arg228His	rs4757987	0,393	90	32	35,6	172	0	0,0	0,000	0
**H6548**	chr11	5841782-5841783	T	C	ATT->GTT	**OR52E6**	Ile39Val	rs4362173	0,370	94	27	28,7	212	0	0,0	0,000	0
**H6548**	chr11	5545258-5545259	C	T	GCA->ACA	**OR52H1**	Ala89Thr	rs10769054	0,507	87	26	29,9	154	0	0,0	0,000	0
**H6548**	chr11	5047431-5047432	C	T	CGA->TGA	**OR52J3**	Arg303*	rs57026471	0,137	50	16	32,0	105	0	0,0	0,000	0
**H6548**	chr11	5788442-5788443	C	T	TGT->TAT	**OR52N1**	Cys125Tyr	rs10769224	0,301	108	30	27,8	211	0	0,0	0,000	0

**H6548**	chr11	5755253-5755254	A	T	AGA->TGA	**OR52N4**	Arg172*	rs4910844	0,232	122	33	27,1	281	0	0,0	0,000	0
**H6548**	chr11	5178259-5178260	T	G	AGA->AGC	**OR52Z1**	Arg98Ser	rs74051402	0,002	74	20	27,0	172	0	0,0	0,000	0
**H6548**	chr11	5968184-5968185	C	T	GTG->ATG	**OR56A5**	Val104Met	rs7114672	0,403	91	26	28,6	238	0	0,0	0,000	0
**H6548**	chr3	98088099-98088100	T	C	TAA->CAA	**OR5AC2**	*310Gln	rs80220955	0,187	36	14	38,9	105	0	0,0	0,000	0
**H6548**	chr11	56989188-56989189	G	A	ATG->ATA	**OR5AK2**	Met92Ile	rs2853083	0,638	78	28	35,9	135	0	0,0	0,000	0
**H6548**	chr11	56641533-56641534	A	T	GAT->GAA	**OR5AP2**	Asp302Glu	rs61747872	0,045	52	21	40,4	95	0	0,0	0,000	0
**H6548**	chr11	56663739-56663740	C	T	CAG->TAG	**OR5AR1**	Gln19*	rs11228710	0,686	122	70	57,4	154	0	0,0	0,000	0
**H6548**	chr11	7849122-7849123	A	G	AGG->GGG	**OR5E1P**	Arg25Gly	rs61759825	0,215	63	16	25,4	154	0	0,0	0,000	0
**H6548**	chr3	98133384-98133385	T	A	TCT->ACT	**OR5H1**	Ser230Thr	rs9849637	0,177	57	17	29,8	112	0	0,0	0,000	0
**H6548**	chr3	98282932-98282933	G	C	GAG->CAG	**OR5H2**	Glu16Gln	rs72487753	0,148	44	18	40,9	68	0	0,0	0,000	0
**H6548**	chr11	55935534-55935535	G	A	CCG->CTG	**OR5I1**	Pro289Leu	rs61995929	0,040	67	17	25,4	101	0	0,0	0,000	0
**H6548**	chr11	56612657-56612658	C	G	AGC->ACC	**OR5M1**	Ser282Thr	rs4939078	0,380	120	45	37,5	232	0	0,0	0,000	0
**H6548**	chr11	56470364-56470365	C	A	GTG->TTG	**OR5M3**	Val45Leu	rs755067452	0,000	60	27	45,0	108	0	0,0	0,000	0

**H6548**	chr11	7796308-7796309	C	T	GTC->ATC	**OR5P2**	Val212Ile	rs78460198	0,254	99	43	43,4	140	0	0,0	0,000	0
**H6548**	chr11	56418032-56418033	G	C	GCC->GGC	**OR5R1**	Ala67Gly		0,000	75	19	25,3	135	0	0,0	0,000	0
**H6548**	chr11	56276127-56276128	A	G	AGC->GGC	**OR5T1**	Ser164Gly	rs12360890	0,433	104	55	52,9	254	0	0,0	0,000	0
**H6548**	chr12	55492704-55492705	T	A	TTT->ATT	**OR6C68**	Phe110Ile	rs7304753	0,225	73	38	52,1	254	0	0,0	0,000	0
**H6548**	chr12	55469439-55469440	C	G	AAG->AAC	**OR6C70**	Lys233Asn	rs60683621	0,225	105	58	55,2	187	0	0,0	0,000	0
**H6548**	chr1	158755020-158755021	G	A	GGA->GAA	**OR6K6**	Gly69Glu	rs114533687	0,003	104	26	25,0	228	0	0,0	0,000	0
**H6548**	chr11	123805679-123805680	G	A	CGT->TGT	**OR6M1**	Arg224Cys	rs76301014	0,159	89	25	28,1	174	0	0,0	0,000	0
**H6548**	chr19	14841425-14841426	A	G	ATG->ACG	**OR7A10**	Met151Thr	rs12972670	0,145	102	32	31,4	246	0	0,0	0,000	0
**H6548**	chr19	14799397-14799398	C	G	GTG->CTG	**OR7C1**	Val247Leu	rs73004304	0,180	101	26	25,7	171	0	0,0	0,000	0
**H6548**	chr11	3391792-3391793	C	T	CGG->CAG	**OR7E12P**	Arg13Gln	rs61877985	0,020	34	9	26,5	42	0	0,0	0,010	0
**H6548**	chr11	124424806-124424807	T	C	GAG->GGG	**OR8B4**	Glu22Gly	rs10750270	0,328	73	27	37,0	100	0	0,0	0,000	0
**H6548**	chr11	56291058-56291059	C	T	GGT->AGT	**OR8H1**	Gly2Ser	rs11600896	0,116	65	33	50,8	173	0	0,0	0,000	0
**H6548**	chr11	56376115-56376116	C	T	CGC->TGC	**OR8U1**	Arg165Cys	rs17150411	0,332	87	47	54,0	180	0	0,0	0,000	0

**H6548**	chr11	56743146-56743147	A	G	GTA->GCA	**OR9G4**	Val222Ala	rs513873	0,251	95	24	25,3	156	2	1,3	0,000	0
**H6548**	chr1	52388506-52388507	A	G	TCC->CCC	**ORC1**	Ser440Pro	rs61756136	0,001	69	23	33,3	124	0	0,0	0,000	16
**H6548**	chr2	178324231-178324232	G	A	CGG->CAG	**OSBPL6**	Arg53Gln; Arg32Gln	rs35032920	0,091	51	20	39,2	108	0	0,0	0,000	0
**H6548**	chr17	47816452-47816453	C	T	GCC->ACC	**OSBPL7**	Ala320Thr	rs191850189	0,003	100	37	37,0	109	0	0,0	0,000	0
**H6548**	chr12	80353428-80353429	C	G	CTA->GTA	**OTOGL**	Leu1829Val	rs7312569	0,220	74	22	29,7	118	0	0,0	0,000	14
**H6548**	chr20	16748492-16748493	T	C	CTC->CCC	**OTOR**	Leu31Pro	rs6135876	0,196	125	58	46,4	217	0	0,0	0,000	32
**H6548**	chr11	7706324-7706325	T	C	ACT->GCT	**OVCH2**	Thr24Ala	rs12289558	0,301	53	19	35,9	105	0	0,0	0,000	0
**H6548**	chr1	111414474-111414475	C	G	GAA->CAA	**OVGP1**	Glu638Gln; Glu324Gln; Glu676Gln	rs7825	0,517	30	12	40,0	66	0	0,0	0,000	22
**H6548**	chr14	22769895-22769896	G	A	CGA->CAA	**OXA1L**	Arg242Gln; Arg166Gln	rs745756054	0,000	97	26	26,8	196	0	0,0	0,000	0
**H6548**	chr11	73235158-73235159	C	T	CGC->TGC	**P2RY2**	Arg334Cys	rs1626154	0,149	308	96	31,2	250	0	0,0	0,000	16
**H6548**	chrX	70259091-70259092	T	G	AAC->ACC	**P2RY4**	Asn178Thr	rs1152187	0,253	128	72	56,3	242	1	0,4	0,000	0
**H6548**	chr19	39386313-39386314	T	C	AGG->GGG	**PAF1**	Arg425Gly	rs748482850	0,000	137	36	26,3	174	0	0,0	0,000	27
**H6548**	chr6	10709398-10709399	G	A	GTA->ATA	**PAK1IP1**	Val376Ile	rs72821574	0,012	42	11	26,2	51	0	0,0	0,008	36

**H6548**	chr15	40265862-40265863	A	G	ATG->GTG	**PAK6**	Met76Val	rs2412504	0,114	265	100	37,7	255	0	0,0	0,000	53
**H6548**	chr19	14053714-14053715	G	C	CCT->GCT	**PALM3**	Pro638Ala	rs61749577	0,021	414	120	29,0	224	0	0,0	0,000	0
**H6548**	chr14	73252125-73252126	G	A	GCG->ACG	**PAPLN**	Ala291Thr; Ala318Thr	rs145176574	0,000	286	83	29,0	137	0	0,0	0,000	0
**H6548**	chr2	205124453-205124454	C	G	GAC->GAG	**PARD3B**	Asp431Glu		0,000	45	12	26,7	139	0	0,0	0,000	25
**H6548**	chr3	122699902-122699903	G	A	AGG->AAG	**PARP14**	Arg450Lys; Arg314Lys; Arg167Lys	rs11719086	0,193	176	46	26,1	221	0	0,0	0,000	44
**H6548**	chr13	24455079-24455080	C	T	GCC->ACC	**PARP4**	Ala899Thr	rs2275660	0,739	70	32	45,7	141	0	0,0	0,000	61
**H6548**	chr1	154946603-154946604	C	T	GGT->GAT	**PBXIP1**	Gly357Asp; Gly328Asp; Gly202Asp	rs2061690	0,498	216	152	70,4	181	0	0,0	0,000	63
**H6548**	chr5	140788484-140788485	A	G	TAT->TGT	**PCDHA1**	Tyr732Cys	rs2240696	0,525	416	125	30,1	239	0	0,0	0,000	0
**H6548**	chr5	140802062-140802063	T	C	ATT->ACT	**PCDHA3**	Ile289Thr	rs3733709	0,104	98	37	37,8	197	0	0,0	0,000	0
**H6548**	chr5	140807736-140807737	C	T	CCT->TCT	**PCDHA4**	Pro184Ser	rs3822346	0,547	152	50	32,9	260	1	0,4	0,000	7
**H6548**	chr5	140822756-140822757	C	G	CCA->GCA	**PCDHA5**	Pro328Ala	rs782646318	0,000	145	53	36,6	267	0	0,0	0,000	0
**H6548**	chr5	140842571-140842572	A	C	AGA->AGC	**PCDHA8**	Arg417Ser	rs3733705	0,405	479	190	39,7	525	1	0,2	0,000	0
**H6548**	chr5	141341386-141341387	T	C	TTT->CTT	**PCDHGA2**	Phe806Leu	rs66823521	0,089	149	54	36,2	181	0	0,0	0,000	0

**H6548**	chr5	141344267-141344268	C	T	CCG->TCG	**PCDHGA3**	Pro79Ser	rs11575947	0,087	54	16	29,6	123	0	0,0	0,000	0
**H6548**	chr2	119546243-119546244	A	T	GAA->GTA	**PCDP1**	Glu38Val	rs2579624	0,537	21	7	33,3	49	0	0,0	0,002	7
**H6548**	chr20	57563564-57563565	A	G	ATT->GTT	**PCK1**	Ile267Val	rs8192708	0,091	65	21	32,3	150	0	0,0	0,000	11
**H6548**	chr7	82953529-82953530	C	T	GTT->ATT	**PCLO**	Val2475Ile	rs10954696	0,360	82	22	26,8	146	0	0,0	0,000	12
**H6548**	chr8	17972461-17972462	C	T	CGC->TGC	**PCM1**	Arg1240Cys; Arg1241Cys; Arg1279Cys;		0,000	107	34	31,8	144	0	0,0	0,000	36
**H6548**	chr21	46366608-46366609	A	G	ACC->GCC	**PCNT**	Thr879Ala; Thr761Ala	rs2839227	0,209	197	50	25,4	190	0	0,0	0,000	19
**H6548**	chr1	232984445-232984446	G	A	GCC->GTC	**PCNXL2**	Ala2091Val	rs113070999	0,033	212	61	28,8	180	0	0,0	0,000	0
**H6548**	chr11	65618734-65618735	C	G	TCC->TGC	**PCNXL3**	Ser458Cys	rs1193851	0,279	311	122	39,2	341	0	0,0	0,000	0
**H6548**	chr19	1489817-1489818	C	T	CGC->CAC	**PCSK4**	Arg90His	rs12972390	0,030	262	69	26,3	293	0	0,0	0,000	13
**H6548**	chr12	20369317-20369318	G	A	GAC->AAC	**PDE3A**	Asp12Asn	rs12305038	0,374	254	84	33,1	136	0	0,0	0,000	14
**H6548**	chr10	93622015-93622016	T	A	TCA->ACA	**PDE6C**	Ser270Thr	rs701865	0,375	78	44	56,4	163	0	0,0	0,000	6
**H6548**	chr16	286915-286916	C	T	CCC->TCC	**PDIA2**	Pro502Ser; Pro347Ser	rs1048786	0,154	132	54	40,9	124	0	0,0	0,000	12
**H6548**	chr1	147178427-147178428	G	A	ATG->ATA	**PDIA3P1**	Met105Ile	rs72708526	0,036	80	28	35,0	143	0	0,0	0,000	0

**H6548**	chr11	119188694-119188695	G	A	CGG->CAG	**PDZD3**	Arg372Gln; Arg359Gln; Arg358Gln;	rs1815811	0,584	130	83	63,9	163	1	0,6	0,000	0
**H6548**	chr10	101010756-101010757	T	C	CAT->CGT	**PDZD7**	His711Arg	rs34616847	0,028	95	31	32,6	104	1	1,0	0,000	7
**H6548**	chr3	73384217-73384218	G	A	GCG->GTG	**PDZRN3**	Ala76Val; Ala500Val; Ala503Val;	rs3205537	0,321	311	113	36,3	289	0	0,0	0,000	16
**H6548**	chrX	77969381-77969382	C	T	CGC->CAC	**PGAM4**	Arg86His	rs62621209	0,044	68	44	64,7	92	0	0,0	0,000	0
**H6548**	chr1	153304946-153304947	C	T	GGC->AGC	**PGLYRP3**	Gly126Ser	rs843971	0,425	101	50	49,5	151	0	0,0	0,000	8
**H6548**	chr1	63631760-63631761	C	T	CGT->TGT	**PGM1**	Arg221Cys; Arg24Cys; Arg239Cys	rs1126728	0,243	74	21	28,4	127	0	0,0	0,000	8
**H6548**	chr6	83170447-83170448	C	T	GAT->AAT	**PGM3**	Asp466Asn; Asp385Asn; Asp494Asn	rs473267	0,281	79	20	25,3	164	0	0,0	0,000	10
**H6548**	chr12	55412427-55412428	G	A	CCG->CTG	**PHC1**	Pro295Leu; Pro770Leu		0,000	126	34	27,0	400	1	0,3	0,000	28
**H6548**	chr16	71649814-71649815	A	G	TTA->TCA	**PHLPP2**	Leu949Ser; Leu1016Ser	rs61733127	0,120	84	21	25,0	234	0	0,0	0,000	66
**H6548**	chr19	4013193-4013194	C	T	CCG->CTG	**PIAS4**	Pro100Leu	rs2289867	0,000	381	111	29,1	263	0	0,0	0,000	48
**H6548**	chr13	72795503-72795504	A	G	ATT->GTT	**PIBF1**	Ile167Val	rs1372000	0,264	57	19	33,3	81	0	0,0	0,000	32
**H6548**	chr16	88733730-88733731	C	T	GGC->AGC	**PIEZO1**	Gly319Ser; Gly782Ser	rs200970763	0,003	246	70	28,5	104	0	0,0	0,000	15
**H6548**	chr18	10681713-10681714	C	T	GTT->ATT	**PIEZO2**	Val255Ile; Val2463Ile	rs3748428	0,130	92	28	30,4	161	0	0,0	0,000	12

**H6548**	chr16	583353-583354	G	A	TGT->TAT	**PIGQ**	Cys668Tyr; Cys238Tyr	rs710925	0,492	228	76	33,3	227	1	0,4	0,000	7
**H6548**	chr10	96709935-96709936	C	T	GAG->AAG	**PIK3AP1**	Glu21Lys	rs17112076	0,157	170	50	29,4	195	0	0,0	0,000	43
**H6548**	chr12	18496122-18496123	C	T	CCG->CTG	**PIK3C2G**	Pro952Leu; Pro730Leu; Pro911Leu	rs12312266	0,358	40	13	32,5	89	0	0,0	0,000	34
**H6548**	chr1	20650506-20650507	A	C	AAT->ACT	**PINK1**	Asn521Thr; Asn214Thr	rs1043424	0,298	47	15	31,9	135	0	0,0	0,000	15
**H6548**	chr11	67494939-67494940	C	T	CGG->CAG	**PITPNM1**	Arg165Gln; Arg883Gln; Arg882Gln	rs144939807	0,008	394	148	37,6	247	0	0,0	0,000	7
**H6548**	chr10	3149637-3149638	G	C	TTC->TTG	**PITRM1**	Phe618Leu; Phe586Leu; Phe610Leu;	rs41305673	0,037	100	34	34,0	175	0	0,0	0,000	5
**H6548**	chr5	135028827-135028828	C	G	GGC->GCC	**PITX1**	Gly299Ala	rs479632	0,301	379	185	48,8	230	0	0,0	0,000	18
**H6548**	chr12	130357092-130357093	G	A	AGA->AAA	**PIWIL1**	Arg527Lys	rs1106042	0,080	60	18	30,0	136	0	0,0	0,000	32
**H6548**	chr22	24749785-24749786	G	A	CCA->TCA	**PIWIL3**	Pro266Ser; Pro375Ser	rs1475853	0,030	75	22	29,3	104	0	0,0	0,000	44
**H6548**	chr16	2110971-2110972	A	G	TGG->CGG	**PKD1**	Trp1399Arg	rs116092985	0,066	235	88	37,5	161	0	0,0	0,000	20
**H6548**	chr16	81220311-81220312	A	G	GTT->GCT	**PKD1L2**	Val20Ala	rs9924530	0,567	94	50	53,2	191	1	0,5	0,000	11
**H6548**	chr16	71934023-71934024	C	T	CGT->CAT	**PKD1L3**	Arg1572His	rs17358402	0,194	203	67	33,0	375	0	0,0	0,000	7
**H6548**	chr4	88007815-88007816	G	C	CGG->CCG	**PKD2**	Arg28Pro	rs1805044	0,145	103	64	62,1	37	0	0,0	0,000	17

**H6548**	chr22	46261434-46261435	G	T	CCC->ACC	**PKDREJ**	Pro630Thr		0,000	88	27	30,7	128	0	0,0	0,000	0
**H6548**	chr8	109420643-109420644	A	G	TAC->TGC	**PKHD1L1**	Tyr884Cys	rs186716306	0,002	41	14	34,2	111	0	0,0	0,000	25
**H6548**	chr1	19920382-19920383	C	T	CGC->CAC	**PLA2G2E**	Arg118His	rs61729970	0,094	183	47	25,7	217	1	0,5	0,000	0
**H6548**	chr19	48099786-48099787	G	A	CGA->TGA	**PLA2G4C**	Arg46*; Arg111*; Arg121*	rs143994362	0,000	54	20	37,0	95	0	0,0	0,000	27
**H6548**	chr19	43648947-43648948	A	G	CTC->CCC	**PLAUR**	Leu317Pro; Leu272Pro	rs4760	0,120	54	14	25,9	123	0	0,0	0,000	51
**H6548**	chr2	28591165-28591166	A	G	ATG->GTG	**PLB1**	Met218Val; Met396Val; Met708Val;	rs11681826	0,247	79	26	32,9	228	0	0,0	0,000	5
**H6548**	chr20	41168824-41168825	T	C	ATC->ACC	**PLCG1**	Ile137Thr; Ile813Thr; Ile439Thr	rs753381	0,620	86	33	38,4	169	0	0,0	0,000	42
**H6548**	chr12	18688180-18688181	G	A	TCA->TTA	**PLCZ1**	Ser282Leu; Ser241Leu; Ser396Leu;	rs10505830	0,031	36	14	38,9	82	0	0,0	0,000	5
**H6548**	chr17	4817173-4817174	C	T	ACA->ATA	**PLD2**	Thr434Ile; Thr577Ile	rs1052748	0,387	94	41	43,6	207	0	0,0	0,000	29
**H6548**	chr19	39425117-39425118	C	G	CCA->GCA	**PLEKHG2**	Pro1329Ala; Pro1107Ala	rs31728	0,555	256	135	52,7	118	0	0,0	0,000	15
**H6548**	chr1	15727156-15727157	G	A	CGG->CAG	**PLEKHM2**	Arg362Gln	rs370612427	0,000	251	89	35,5	165	0	0,0	0,000	0
**H6548**	chr19	4510837-4510838	G	A	GCG->GTG	**PLIN4**	Ala51Val; Ala1027Val	rs145519622	0,010	239	70	29,3	429	0	0,0	0,000	13
**H6548**	chr22	50283978-50283979	T	C	AAC->GAC	**PLXNB2**	Asn759Asp	rs79966207	0,124	385	162	42,1	156	0	0,0	0,000	17

**H6548**	chr3	129585953-129585954	G	A	CCA->TCA	**PLXND1**	Pro617Ser	rs2285372	0,052	308	97	31,5	310	0	0,0	0,000	31
**H6548**	chr18	59900466-59900467	G	A	GGA->GAA	**PMAIP1**	Gly33Glu	rs78800940	0,156	189	52	27,5	158	0	0,0	0,000	80
**H6548**	chr15	74035799-74035800	G	T	GGT->GTT	**PML**	Gly780Val; Gly732Val; Gly209Val	rs743581	0,360	116	32	27,6	217	0	0,0	0,000	59
**H6548**	chr7	103311675-103311676	G	T	GAG->GAT	**PMPCB**	Glu396Asp; Glu291Asp; Glu271Asp	rs3087615	0,090	82	25	30,5	151	1	0,7	0,000	0
**H6548**	chr2	68157964-68157965	A	G	AGG->GGG	**PNO1**	Arg11Gly	rs2044693	0,709	213	145	68,1	303	0	0,0	0,000	13
**H6548**	chr22	43891069-43891070	G	A	CTC->TTC	**PNPLA5**	Leu140Phe	rs2071883	0,194	208	103	49,5	170	0	0,0	0,000	14
**H6548**	chr7	108472434-108472435	A	T	ATG->AAG	**PNPLA8**	Met772Lys; Met672Lys; Met707Lys	rs558273792	0,000	75	25	33,3	125	0	0,0	0,000	11
**H6548**	chr2	55684984-55684985	T	C	ATT->GTT	**PNPT1**	Ile121Val	rs782572	0,425	67	18	26,9	139	0	0,0	0,000	32
**H6548**	chr5	75707852-75707853	T	C	CAT->CGT	**POC5**	His11Arg; His36Arg	rs2307111	0,560	55	17	30,9	99	0	0,0	0,000	16
**H6548**	chr7	131508979-131508980	C	T	GTC->ATC	**PODXL**	Val358Ile; Val326Ile	rs3212298	0,064	63	19	30,2	151	0	0,0	0,000	54
**H6548**	chr2	74958728-74958729	G	T	GGA->GTA	**POLE4**	Gly17Val	rs12366	0,396	281	133	47,3	201	1	0,5	0,000	0
**H6548**	chr18	54280889-54280890	A	G	ATA->ATG	**POLI**	Ile193Met; Ile261Met; Ile158Met	rs3218784	0,018	26	11	42,3	58	0	0,0	0,000	15
**H6548**	chr4	2198638-2198639	G	A	CCG->TCG	**POLN**	Pro265Ser	rs140869775	0,002	82	26	31,7	155	0	0,0	0,000	38

**H6548**	chr10	77986122-77986123	T	C	ATC->GTC	**POLR3A**	Ile980Val	rs146253630	0,006	60	19	31,7	126	0	0,0	0,000	7
**H6548**	chr7	72926404-72926405	A	G	AAC->AGC	**POM121**	Asn263Ser	rs367944164	0,001	119	30	25,2	247	0	0,0	0,000	22
**H6548**	chr6	27312072-27312073	T	C	CAG->CGG	**POM121L2**	Gln33Arg	rs2235233	0,234	280	87	31,1	449	0	0,0	0,000	0
**H6548**	chr22	20691108-20691109	C	T	GCG->GTG	**POM121L4P**	Ala360Val	rs12158743	0,125	44	15	34,1	132	0	0,0	0,000	0
**H6548**	chr14	19413461-19413462	G	C	CCT->CGT	**POTEG**	Pro434Arg	rs199722113	0,823	46	29	63,0	78	0	0,0	0,000	0
**H6548**	chr19	10113849-10113850	C	T	GCC->GCT; CCC->CTC	**PPAN-P2RY11**	Ala499Ala; Pro520Leu	rs3745600	0,417	516	159	30,8	275	0	0,0	0,000	0
**H6548**	chr9	4662393-4662394	A	G	AGC->GGC	**PPAPDC2**	Ser7Gly	rs34250374	0,165	73	23	31,5	23	0	0,0	0,011	0
**H6548**	chr1	203039203-203039204	G	C	GAC->CAC	**PPFIA4**	Asp213His		0,000	79	25	31,7	108	0	0,0	0,000	0
**H6548**	chr2	169636592-169636593	T	A	GAT->GAA	**PPIG**	Asp445Glu; Asp430Glu; Asp438Glu;	rs1050354	0,573	65	35	53,9	54	0	0,0	0,000	12
**H6548**	chr2	44244269-44244270	T	G	TCT->GCT	**PPM1B**	Ser393Ala	rs12613956	0,214	46	23	50,0	51	0	0,0	0,000	29
**H6548**	chr1	112712833-112712834	C	A	TTG->TTT	**PPM1J**	Leu213Phe	rs34611728	0,097	38	10	26,3	83	0	0,0	0,000	0
**H6548**	chr9	135485810-135485811	T	C	ATG->ACG	**PPP1R26**	Met434Thr	rs1808998	0,343	266	100	37,6	239	1	0,4	0,000	0
**H6548**	chr14	64564815-64564816	C	T	ACT->ATT	**PPP1R36**	Thr83Ile	rs6573560	0,572	90	24	26,7	204	0	0,0	0,000	0

**H6548**	chr7	95250103-95250104	G	A	GAA->AAA	**PPP1R9A**	Glu749Lys; Glu727Lys		0,000	44	13	29,6	60	0	0,0	0,001	32
**H6548**	chr3	136001697-136001698	A	G	GAT->GGT	**PPP2R3A**	Asp67Gly	rs9814557	0,250	74	25	33,8	158	0	0,0	0,000	38
**H6548**	chr22	50439766-50439767	G	A	AGG->AAG	**PPP6R2**	Arg732Lys; Arg706Lys; Arg705Lys;	rs13057311	0,243	222	72	32,4	145	0	0,0	0,000	0
**H6548**	chr17	48724645-48724646	C	G	ACG->AGG	**PRAC2**	Thr79Arg; Thr129Arg	rs2271891	0,463	97	28	28,9	197	0	0,0	0,000	0
**H6548**	chr1	12827689-12827690	C	T	GGA->GAA	**PRAMEF11**	Gly103Glu	rs2994114	0,224	209	92	44,0	427	0	0,0	0,000	0
**H6548**	chr17	76545046-76545047	A	G	ATG->GTG	**PRCD**	Met224Val	rs4232870	0,506	96	38	39,6	35	0	0,0	0,000	5
**H6548**	chr11	82853251-82853252	T	G	GAA->GAC	**PRCP**	Glu133Asp; Glu112Asp	rs2229437	0,157	69	19	27,5	122	0	0,0	0,000	10
**H6548**	chr8	70068502-70068503	T	C	AAA->GAA	**PRDM14**	Lys244Glu	rs3750228	0,166	39	12	30,8	96	0	0,0	0,000	49
**H6548**	chr21	41878736-41878737	C	T	GGT->GAT	**PRDM15**	Gly124Asp	rs143393748	0,101	243	74	30,5	171	0	0,0	0,000	0
**H6548**	chr10	70600630-70600631	G	A	GCG->GTG	**PRF1**	Ala91Val	rs35947132	0,030	173	59	34,1	129	1	0,8	0,000	34
**H6548**	chr1	186304861-186304862	C	T	CGG->TGG	**PRG4**	Arg180Trp; Arg139Trp	rs2273779	0,302	46	16	34,8	83	0	0,0	0,000	6
**H6548**	chr8	47798393-47798394	A	G	ATT->ACT	**PRKDC**	Ile3434Thr	rs7830743	0,140	31	12	38,7	67	0	0,0	0,000	60
**H6548**	chr16	11273337-11273338	G	C	CAC->CAG	**PRM3**	His86Gln	rs35598356	0,148	276	185	67,0	141	0	0,0	0,000	30

**H6548**	chr20	4699731-4699732	A	G	AAC->AGC	**PRNP**	Asn171Ser; Asn141Ser	rs16990018	0,005	238	73	30,7	279	0	0,0	0,000	5
**H6548**	chr5	139393427-139393428	A	G	TCC->CCC	**PROB1**	Ser552Pro		0,000	233	75	32,2	395	1	0,3	0,000	0
**H6548**	chr2	68645938-68645939	A	G	AGC->GGC	**PROKR1**	Ser40Gly	rs7570797	0,077	137	35	25,6	326	0	0,0	0,000	27
**H6548**	chr22	31715001-31715002	G	A	CCT->CTT	**PRR14L**	Pro745Leu; Pro946Leu	rs117343825	0,011	68	17	25,0	133	0	0,0	0,000	0
**H6548**	chr7	29566699-29566700	C	A	CCC->CAC	**PRR15**	Pro124His	rs112093295	0,113	83	36	43,4	85	0	0,0	0,000	12
**H6548**	chr7	128359590-128359591	C	T	CGG->CAG	**PRRT4**	Arg134Gln	rs56272205	0,165	165	129	78,2	144	0	0,0	0,000	0
**H6548**	chr4	151291333-151291334	C	T	CGT->TGT	**PRSS48**	Arg290Cys; Arg147Cys	rs13126069	0,413	104	32	30,8	221	0	0,0	0,000	0
**H6548**	chr4	7434758-7434759	C	A	GCC->TCC	**PSAPL1**	Ala41Ser	rs11548325	0,179	235	179	76,2	187	0	0,0	0,000	0
**H6548**	chr8	142682338-142682339	T	G	ATT->AGT	**PSCA**	Ile122Ser	rs1045547	0,444	129	38	29,5	160	0	0,0	0,000	75
**H6548**	chr19	42729131-42729132	T	C	AAA->GAA	**PSG3**	Lys412Glu	rs17420676	0,010	120	32	26,7	309	0	0,0	0,000	3
**H6548**	chr19	43205484-43205485	C	G	GTC->CTC	**PSG4**	Val18Leu	rs141508635	0,149	62	27	43,6	226	0	0,0	0,000	5
**H6548**	chr14	35278711-35278712	C	T	CGG->TGG	**PSMA6**	Arg5Trp	rs17103147	0,113	51	22	43,1	123	0	0,0	0,000	19
**H6548**	chr2	53892887-53892888	A	T	TCA->ACA	**PSME4**	Ser1257Thr; Ser1371Thr; Ser746Thr;	rs805408	0,322	90	25	27,8	122	0	0,0	0,000	0

**H6548**	chr6	31138723-31138724	C	-T^C		**PSORS1C1**	Pro38	rs9278990	0,031	119	50	42,0	207	0	0,0	0,000	7
**H6548**	chr1	11519446-11519447	G	C	GGC->GCC	**PTCHD2**	Gly661Ala	rs2072993	0,164	133	38	28,6	193	0	0,0	0,000	51
**H6548**	chr10	27403349-27403350	A	C	TGC->GGC	**PTCHD3**	Cys407Gly	rs2484180	0,400	102	26	25,5	114	0	0,0	0,000	0
**H6548**	chr9	128128780-128128781	G	A	GGG->GAG	**PTGES2-AS1**	Gly46Glu	rs12377832	0,146	365	95	26,0	431	0	0,0	0,000	0
**H6548**	chr1	116986835-116986836	G	A	GTC->ATC	**PTGFRN**	Val837Ile	rs10801922	0,491	110	34	30,9	178	0	0,0	0,000	64
**H6548**	chr3	46903509-46903510	G	A	GAG->AAG	**PTH1R**	Glu546Lys	rs77048718	0,016	153	46	30,1	136	0	0,0	0,000	17
**H6548**	chr6	43132798-43132799	T	G	CTG->CGG	**PTK7**	Leu447Arg	rs78422682	0,087	81	27	33,3	197	0	0,0	0,000	56
**H6548**	chr14	88479062-88479063	C	G	GGG->CGG	**PTPN21**	Gly586Arg; Gly790Arg	rs3825676	0,021	386	102	26,4	189	0	0,0	0,000	42
**H6548**	chr12	70590114-70590115	A	C	GAT->GAG	**PTPRB**	Asp415Glu; Asp633Glu; Asp632Glu;	rs2165627	0,105	91	24	26,4	242	0	0,0	0,000	40
**H6548**	chr12	80542246-80542247	A	G	AAT->GAT	**PTPRQ**	Asn1202Asp	rs17713202	0,133	46	12	26,1	99	0	0,0	0,000	18
**H6548**	chr7	121873506-121873507	T	G	ATC->AGC	**PTPRZ1**	Ile3Ser	rs740965	0,165	129	34	26,4	75	0	0,0	-2,000	34
**H6548**	chr8	66767958-66767959	A	G	ATC->ACC	**PTTG3P**	Ile16Thr	rs6472285	0,086	66	19	28,8	123	0	0,0	0,000	0
**H6548**	chr7	100219961-100219962	G	T	GAG->TAG	**PVRIG**	Glu18*	rs7786505	0,286	240	67	27,9	266	0	0,0	0,000	0

**H6548**	chr8	51319926-51319927	G	T	GAC->GAA	**PXDNL**	Asp1452Glu	rs1052704	0,271	85	23	27,1	121	0	0,0	0,000	0
**H6548**	chr6	36426038-36426039	A	G	GTT->GCT	**PXT1**	Val15Ala	rs664370	0,403	58	34	58,6	89	0	0,0	0,000	36
**H6548**	chr8	143607118-143607119	C	T	CGG->CAG	**PYCRL**	Arg69Gln	rs11549789	0,078	73	21	28,8	54	0	0,0	0,000	0
**H6548**	chr17	43953162-43953163	G	C	ACG->AGG	**PYY**	Thr72Arg	rs1058046	0,625	357	254	71,2	387	0	0,0	0,000	8
**H6548**	chr12	9194195-9194196	G	C	CTC->GTC	**PZP**	Leu379Val; Leu248Val	rs12230214	0,265	76	27	35,5	91	0	0,0	0,000	10
**H6548**	chr4	121329578-121329579	A	G	TTG->TCG	**QRFPR**	Leu344Ser	rs2302310	0,251	58	22	37,9	159	0	0,0	0,000	7
**H6548**	chr6	106640355-106640356	C	T	GCG->GTG	**QRSL1**	Ala11Val	rs36016898	0,071	44	22	50,0	80	0	0,0	0,000	0
**H6548**	chr9	136223839-136223840	G	A	CTT->TTT	**QSOX2**	Leu200Phe		0,000	91	26	28,6	154	0	0,0	0,000	0
**H6548**	chr20	44337200-44337201	T	G	TTC->TGC	**R3HDML**	Phe15Cys	rs11699901	0,100	213	100	47,0	188	0	0,0	0,000	0
**H6548**	chr2	129980589-129980590	G	A	GCT->ACT	**RAB6C**	Ala159Thr	rs4662674	0,649	144	40	27,8	417	0	0,0	0,000	55
**H6548**	chr11	67393489-67393490	A	G	ATG->GTG	**RAD9A**	Met1Val	rs2066496	0,109	162	51	31,5	167	0	0,0	0,000	57
**H6548**	chr6	149890866-149890867	C	T	CGC->CAC	**RAET1E**	Arg12His	rs9383583	0,196	78	24	30,8	105	0	0,0	0,000	55
**H6548**	chr6	150025395-150025396	T	C	AGG->GGG	**RAET1L**	Arg26Gly	rs1543547	0,423	66	22	33,3	88	0	0,0	0,000	53

**H6548**	chr11	36575762-36575763	A	G	AAG->AGG	**RAG1**	Lys820Arg	rs2227973	0,189	72	23	31,9	165	0	0,0	0,000	22
**H6548**	chr6	13696812-13696813	C	G	GTA->CTA	**RANBP9**	Val219Leu		0,000	51	13	25,5	79	0	0,0	0,000	25
**H6548**	chr7	102595321-102595322	T	C	ATG->GTG	**RASA4B**	Met352Val	rs144395384	0,096	89	24	27,0	117	0	0,0	0,000	0
**H6548**	chr10	44982643-44982644	A	G	AGA->GGA; AGA->GGA	**RASSF4**	Arg88Gly; Arg119Gly; Arg18Gly;	rs870957	0,210	76	25	32,9	134	0	0,0	0,000	64
**H6548**	chr7	5072922-5072923	A	C	GAG->GCG	**RBAK-RBAKDN**	Glu146Ala	rs1130329	0,354	103	37	35,9	57	0	0,0	0,000	0
**H6548**	chr21	14227144-14227145	A	G	CAC->CGC	**RBM11**	His233Arg; His119Arg	rs7280643	0,106	53	14	26,4	98	0	0,0	0,000	0
**H6548**	chr2	237817072-237817073	G	C	GAT->CAT	**RBM44**	Asp53His	rs13393001	0,009	73	21	28,8	143	0	0,0	0,000	0
**H6548**	chr2	160493313-160493314	T	-A^TAG		**RBMS1**	Tyr17		0,000	261	68	26,1	308	0	0,0	0,000	19
**H6548**	chr19	10021323-10021324	G	A	ATG->ATA	**RDH8**	Met222Ile	rs77833898	0,101	146	45	30,8	213	0	0,0	0,000	6
**H6548**	chrX	153941597-153941598	G	A	GAC->GAT; ACC->ATC	**RENBP**	Asp275Asp; Thr248Ile	rs143192047	0,000	37	19	51,4	60	0	0,0	0,000	4
**H6548**	chr19	55761990-55761991	A	G	AAG->AGG	**RFPL4A**	Lys64Arg	rs202102526	0,004	37	11	29,7	74	0	0,0	0,000	0
**H6548**	chr19	13972948-13972949	T	C	ACC->GCC	**RFX1**	Thr370Ala	rs2305780	0,501	133	63	47,4	95	0	0,0	0,000	32
**H6548**	chr15	56095761-56095762	G	A	CCA->TCA	**RFX7**	Pro470Ser; Pro656Ser	rs201488404	0,000	78	20	25,6	188	0	0,0	0,000	0

**H6548**	chr19	11397500-11397501	G	A	CGT->TGT	**RGL3**	Arg379Cys; Arg621Cys; Arg615Cys	rs2291516	0,169	245	76	31,0	202	0	0,0	0,000	12
**H6548**	chr15	93073745-93073746	A	G	CTG->CCG	**RGMA**	Leu4Pro	rs4598860	0,599	232	86	37,1	293	0	0,0	0,000	20
**H6548**	chr16	270759-270760	C	T	GTG->ATG	**RGS11**	Val330Met; Val351Met; Val340Met;	rs9806942	0,124	200	73	36,5	214	0	0,0	0,000	4
**H6548**	chr16	669591-669592	G	A	GCC->ACC	**RHOT2**	Ala88Thr	rs11557858	0,184	180	60	33,3	209	0	0,0	0,000	24
**H6548**	chr5	38955693-38955694	G	A	TCC->TTC	**RICTOR**	Ser837Phe; Ser552Phe	rs2043112	0,392	73	21	28,8	121	0	0,0	0,000	50
**H6548**	chr8	104094653-104094654	T	G	ATA->AGA	**RIMS2**	Ile1024Arg	rs9969594	0,284	174	49	28,2	129	0	0,0	0,000	15
**H6548**	chr20	19960774-19960775	C	T	CCA->TCA	**RIN2**	Pro192Ser; Pro143Ser	rs45488002	0,016	65	23	35,4	106	0	0,0	0,000	16
**H6548**	chr14	92651883-92651884	C	T	CGC->TGC	**RIN3**	Arg279Cys; Arg204Cys	rs117068593	0,137	71	25	35,2	57	0	0,0	0,000	9
**H6548**	chr19	38869956-38869957	C	T	CGA->CAA	**RINL**	Arg329Gln; Arg443Gln	rs113728915	0,124	204	68	33,3	244	0	0,0	0,000	0
**H6548**	chr5	97167818-97167819	C	T	GGG->AGG	**RIOK2**	Gly349Arg	rs160632	0,585	85	47	55,3	142	0	0,0	0,000	23
**H6548**	chr1	40240053-40240054	A	T	GAA->GAT	**RLF**	Glu1784Asp; Glu1475Asp	rs10889205	0,176	68	17	25,0	158	0	0,0	0,000	16
**H6548**	chr15	40754684-40754685	C	A	CAG->CAT	**RMDN3**	Gln33His	rs11558807	0,037	120	38	31,7	182	0	0,0	0,000	0
**H6548**	chr2	86623854-86623855	C	T	CGG->CAG	**RNF103**	Arg3Gln	rs3810829	0,032	113	32	28,3	70	0	0,0	-2,000	0

**H6548**	chr3	49703461-49703462	A	G	AAG->GAG	**RNF123**	Lys596Glu; Lys258Glu	rs35726701	0,015	145	37	25,5	190	0	0,0	0,000	38
**H6548**	chr3	196472332-196472333	G	T	CCA->CAA	**RNF168**	Pro401Gln; Pro234Gln	rs3796129	0,535	107	37	34,6	193	0	0,0	0,000	65
**H6548**	chr17	80325125-80325126	G	A	GCG->ACG	**RNF213**	Ala1041Thr; Ala772Thr	rs61359568	0,046	139	38	27,3	220	0	0,0	0,000	15
**H6548**	chr9	137228679-137228680	C	G	ACA->AGA	**RNF224**	Thr22Arg	rs6606565	0,507	87	23	26,4	50	0	0,0	0,000	0
**H6548**	chr12	121417914-121417915	C	G	CGA->GGA	**RNF34**	Arg214Gly		0,000	44	11	25,0	74	0	0,0	0,000	27
**H6548**	chr17	58358747-58358748	C	T	CGC->CAC	**RNF43**	Arg302His; Arg343His; Arg216His	rs34523089	0,158	240	87	36,3	263	0	0,0	0,000	80
**H6548**	chr9	91724038-91724039	C	T	GTC->ATC	**ROR2**	Val819Ile	rs10761129	0,712	350	258	73,7	323	0	0,0	0,000	29
**H6548**	chr1	151830972-151830973	G	T	CAC->AAC	**RORC**	His53Asn	rs80223568	0,347	56	25	44,6	84	1	1,2	0,000	19
**H6548**	chr8	54628889-54628890	G	A	GCA->ACA	**RP1**	Ala1670Thr	rs446227	0,253	143	48	33,6	201	0	0,0	0,000	16
**H6548**	chr17	81380541-81380542	T	C	AAT->GAT	**RP11-1055B8.3**	Asn16Asp	rs61745998	0,196	113	62	54,9	148	0	0,0	0,000	0
**H6548**	chr9	88647606-88647607	T	A	AAT->TAT	**RP13-60M5.2**	Asn41Tyr	rs28429833	0,171	68	38	55,9	127	0	0,0	0,000	0
**H6548**	chr8	10608971-10608972	G	A	GCC->GTC	**RP1L1**	Ala1709Val	rs13267180	0,380	130	39	30,0	173	0	0,0	0,000	9
**H6548**	chrX	46860062-46860063	C	T	CGG->TGG	**RP2**	Arg282Trp	rs1805147	0,019	205	153	74,6	112	0	0,0	0,000	10

**H6548**	chr16	89561664-89561665	G	A	GCC->ACC; GCC->ACC	**RPL13**	Ala112Thr; Ala93Thr; Gly73Asp	rs9930567	0,160	153	39	25,5	231	0	0,0	0,000	32
**H6548**	chr4	108620446-108620447	T	A	ACC->TCC	**RPL34-AS1**	Thr4Ser	rs3796936	0,348	53	21	39,6	158	0	0,0	0,000	0
**H6548**	chr1	96679457-96679458	T	C	AAG->GAG	**RPL7**	Lys55Glu	rs3748786	0,318	27	13	48,2	32	0	0,0	0,000	16
**H6548**	chr11	61637631-61637632	T	G	CTT->CGT	**RPLP0P2**	Leu318Arg	rs12281961	0,237	57	16	28,1	149	0	0,0	0,000	0
**H6548**	chr11	67433340-67433341	C	T	CCG->CTG	**RPS6KB2**	Pro267Leu	rs55987642	0,043	212	67	31,6	219	0	0,0	0,000	65
**H6548**	chr20	17615491-17615492	T	A	GAG->GTG	**RRBP1**	Glu70Val; Glu267Val; Glu671Val;		0,000	175	58	33,1	190	0	0,0	0,000	38
**H6548**	chr6	7231609-7231610	G	A	GAC->AAC	**RREB1**	Asp1171Asn	rs9379084	0,106	253	148	58,5	144	0	0,0	0,000	63
**H6548**	chr10	97366191-97366192	C	T	GGC->AGC	**RRP12**	Gly160Ser; Gly262Ser; Gly863Ser;	rs2275580	0,529	103	49	47,6	156	0	0,0	0,000	26
**H6548**	chr7	5943431-5943432	C	T	ATG->ATA	**RSPH10B**	Met550Ile	rs148485394	0,281	52	16	30,8	167	0	0,0	0,000	0
**H6548**	chr6	116629570-116629571	G	A	CGC->CAC	**RSPH4A**	Arg556His	rs6927567	0,213	90	23	25,6	118	0	0,0	0,000	5
**H6548**	chr19	12825878-12825879	C	T	GTG->ATG	**RTBDN**	Val173Met; Val167Met; Val179Met;	rs148952767	0,006	191	71	37,2	237	0	0,0	0,000	0
**H6548**	chr10	62198352-62198353	T	C	CAT->CGT	**RTKN2**	His264Arg; His116Arg; His462Arg	rs3125734	0,632	59	26	44,1	97	0	0,0	0,000	42
**H6548**	chr3	187199962-187199963	C	G	CAG->GAG	**RTP1**	Gln229Glu	rs6764714	0,474	322	99	30,8	172	0	0,0	0,000	5

**H6548**	chr3	187698930-187698931	T	C	CAG->CGG	**RTP2**	Gln82Arg	rs11707167	0,596	209	104	49,8	170	2	1,2	0,000	7
**H6548**	chr3	187371134-187371135	G	A	AGC->AAC	**RTP4**	Ser168Asn	rs35224605	0,023	90	25	27,8	174	0	0,0	0,000	14
**H6548**	chr8	92011072-92011073	C	T	GAA->AAA	**RUNX1T1**	Glu126Lys; Glu136Lys; Glu163Lys;		0,000	91	28	30,8	194	1	0,5	0,000	83
**H6548**	chr11	18106011-18106012	C	+CGG		**SAAL1**	Pro10	rs148650821	0,753	268	86	32,1	150	0	0,0	0,000	0
**H6548**	chr20	51791195-51791196	A	C	TTT->TTG	**SALL4**	Phe429Leu	rs143818932	0,001	35	15	42,9	69	0	0,0	0,000	50
**H6548**	chr7	93104452-93104453	C	A	GTG->TTG	**SAMD9**	Val549Leu	rs10279499	0,091	62	18	29,0	123	1	0,8	0,000	50
**H6548**	chr11	9858290-9858291	C	T	GAA->AAA	**SBF2**	Glu435Lys; Glu679Lys	rs7102464	0,073	98	25	25,5	157	0	0,0	0,000	22
**H6548**	chr9	125055996-125055997	C	T	GCT->ACT	**SCAI**	Ala60Thr; Ala37Thr	rs589292	0,253	84	22	26,2	152	0	0,0	0,000	8
**H6548**	chr12	124863716-124863717	C	T	GGC->AGC	**SCARB1**	Gly2Ser	rs4238001	0,097	264	134	50,8	154	0	0,0	0,000	14
**H6548**	chr13	77602666-77602667	G	A	GAA->AAA	**SCEL**	Glu331Lys; Glu311Lys; Glu309Lys	rs201091090	0,001	63	18	28,6	98	0	0,0	0,000	29
**H6548**	chr4	53145559-53145560	G	A	TCC->TTC	**SCFD2**	Ser445Phe		0,000	54	20	37,0	119	0	0,0	0,000	25
**H6548**	chr3	38603928-38603929	T	C	CAC->CGC	**SCN5A**	His558Arg; His424Arg	rs1805124	0,222	205	69	33,7	252	0	0,0	0,000	4
**H6548**	chr8	143810913-143810914	G	A	CCC->CTC	**SCRIB**	Pro422Leu	rs6558394	0,596	361	130	36,0	300	0	0,0	0,000	69

**H6548**	chr1	169854576-169854577	T	C	CAA->CGA	**SCYL3**	Gln213Arg; Gln621Arg; Gln567Arg	rs4656197	0,645	114	52	45,6	209	0	0,0	0,000	0
**H6548**	chr4	75957562-75957563	G	C	TCC->TGC	**SDAD1**	Ser478Cys; Ser575Cys; Ser538Cys	rs2242471	0,339	80	30	37,5	148	0	0,0	0,000	42
**H6548**	chr20	1312602-1312603	C	T	GTG->ATG	**SDCBP2**	Val97Met; Val182Met	rs2273959	0,393	190	98	51,6	414	0	0,0	0,000	0
**H6548**	chr3	195671233-195671234	T	C	ATC->ACC	**SDHAP2**	Ile55Thr	rs9871062	0,388	28	7	25,0	104	9	8,7	0,000	0
**H6548**	chr10	119898887-119898888	C	T	CCT->TCT	**SEC23IP**	Pro209Ser	rs148955366	0,009	88	23	26,1	153	0	0,0	0,000	21
**H6548**	chr15	49000996-49000997	C	A	GTA->TTA	**SECISBP2L**	Val710Leu; Val665Leu; Val472Leu	rs11854184	0,151	72	31	43,1	97	0	0,0	0,000	0
**H6548**	chr18	12984144-12984145	C	A	ACT->AAT	**SEH1L**	Thr342Asn	rs6505776	0,681	86	34	39,5	121	0	0,0	0,000	0
**H6548**	chr1	151366753-151366754	C	T	CGA->CAA	**SELENBP1**	Arg253Gln; Arg211Gln; Arg149Gln;	rs148694055	0,001	105	27	25,7	202	0	0,0	0,000	53
**H6548**	chr1	169707344-169707345	A	G	TTT->CTT	**SELL**	Phe146Leu; Phe206Leu	rs1131498	0,208	54	14	25,9	103	0	0,0	0,000	10
**H6548**	chr22	50208506-50208507	G	C	GCT->CCT	**SELO**	Ala55Pro	rs5771102	0,623	137	76	55,5	120	0	0,0	0,000	0
**H6548**	chr1	169597074-169597075	C	T	GAC->AAC	**SELP**	Asp438Asn; Asp603Asn	rs6127	0,643	55	21	38,2	166	0	0,0	0,000	18
**H6548**	chr17	77402415-77402416	C	T	CCG->CTG	**SEPT9**	Pro145Leu; Pro126Leu; Pro127Leu;	rs34587622	0,091	429	128	29,8	224	0	0,0	0,000	65
**H6548**	chr1	31425791-31425792	C	T	CGT->TGT; GGC->GGT	**SERINC2**	Arg137Cys; Gly172Gly; Gly167Gly;	rs35976845	0,067	119	32	26,9	106	0	0,0	0,000	21

**H6548**	chr14	94378505-94378506	T	G	GAA->GAC	**SERPINA1**	Glu400Asp	rs1303	0,275	86	42	48,8	158	0	0,0	0,000	20
**H6548**	chr14	94643495-94643496	G	A	CGT->CAT	**SERPINA13P**	Arg261His	rs17826595	0,125	79	20	25,3	182	0	0,0	0,000	0
**H6548**	chr14	94309883-94309884	A	C	TCG->GCG	**SERPINA6**	Ser246Ala	rs2228541	0,528	97	53	54,6	306	0	0,0	0,000	8
**H6548**	chr14	94469772-94469773	G	A	CCG->CTG	**SERPINA9**	Pro41Leu	rs45438398	0,093	100	37	37,0	206	0	0,0	0,000	62
**H6548**	chr18	63716119-63716120	C	T	ACG->ATG	**SERPINB11**	Thr148Met	rs17071550	0,182	81	28	34,6	131	0	0,0	0,000	35
**H6548**	chr18	63655777-63655778	C	G	GGA->GCA	**SERPINB3**	Gly351Ala; Gly299Ala	rs3180227	0,161	137	37	27,0	218	0	0,0	0,000	64
**H6548**	chr3	9475684-9475685	C	T	ACA->ATA	**SETD5**	Thr1308Ile; Thr1210Ile; Thr1197Ile;	rs11542009	0,075	135	53	39,3	253	0	0,0	0,000	30
**H6548**	chr22	31615238-31615239	T	C	CTT->CCT	**SFI1**	Leu1087Pro; Leu1056Pro; Leu1005Pro;	rs12171042	0,350	177	92	52,0	87	0	0,0	-2,000	6
**H6548**	chr10	104123006-104123007	A	G	GAC->GGC	**SFR1**	Asp6Gly; Asp19Gly; Asp81Gly	rs10786783	0,176	73	19	26,0	114	0	0,0	0,000	7
**H6548**	chr10	79558906-79558907	C	G	GCT->CCT	**SFTPA2**	Ala91Pro	rs17886395	0,187	75	19	25,3	103	0	0,0	0,000	23
**H6548**	chr8	22164419-22164420	C	T	GCG->GTG	**SFTPC**	Ala246Val	rs1126931	0,166	69	21	30,4	78	0	0,0	0,000	15
**H6548**	chr2	200572324-200572325	A	G	AAC->AGC	**SGOL2**	Asn660Ser	rs17532665	0,110	70	48	68,6	119	0	0,0	0,000	22
**H6548**	chr17	80210801-80210802	C	T	GTG->ATG	**SGSH**	Val387Met; Val184Met	rs62620232	0,013	517	189	36,6	396	0	0,0	0,000	0

**H6548**	chr7	102285203-102285204	C	G	CCT->GCT	**SH2B2**	Pro16Ala	rs803074	0,411	92	27	29,4	171	0	0,0	0,000	19
**H6548**	chr5	172339729-172339730	T	C	ACG->GCG	**SH3PXD2B**	Thr459Ala		0,000	321	121	37,7	210	0	0,0	0,000	11
**H6548**	chr22	50679151-50679152	T	C	ATC->ACC	**SHANK3**	Ile245Thr	rs9616915	0,502	397	164	41,3	319	0	0,0	0,000	6
**H6548**	chr8	144099583-144099584	C	T	GCA->ACA	**SHARPIN**	Ala232Thr	rs201668898	0,000	227	82	36,1	224	0	0,0	0,000	31
**H6548**	chr1	182953079-182953080	G	T	AGC->AGA	**SHCBP1L**	Ser18Arg	rs78487548	0,166	239	83	34,7	122	0	0,0	0,000	0
**H6548**	chr11	406075-406076	G	-C^GA		**SIGIRR**	Arg448		0,000	226	60	26,6	158	0	0,0	0,000	25
**H6548**	chr19	49959040-49959041	G	C	GCA->GGA	**SIGLEC11**	Ala365Gly	rs62113133	0,218	160	44	27,5	287	0	0,0	0,000	24
**H6548**	chr19	49972001-49972002	G	C	GAG->CAG	**SIGLEC16**	Glu62Gln	rs12463036	0,196	430	120	27,9	422	0	0,0	0,000	0
**H6548**	chr19	51627671-51627672	G	A	CGG->TGG	**SIGLEC5**	Arg358Trp	rs8108074	0,173	78	34	43,6	117	0	0,0	0,000	20
**H6548**	chr19	51125271-51125272	A	G	AAG->GAG	**SIGLEC9**	Lys100Glu	rs2075803	0,556	122	36	29,5	411	0	0,0	0,000	32
**H6548**	chr1	232428415-232428416	T	C	AGA->GGA	**SIPA1L2**	Arg543Gly; Arg1469Gly	rs34110867	0,050	30	12	40,0	67	0	0,0	0,000	13
**H6548**	chr20	1566158-1566159	G	A	GCC->GTC	**SIRPB1**	Ala398Val; Ala181Val	rs62623705	0,149	89	39	43,8	206	1	0,5	0,000	28
**H6548**	chr13	21161858-21161859	T	C	ACA->GCA	**SKA3**	Thr254Ala; Thr172Ala	rs17345690	0,128	69	39	56,5	110	0	0,0	0,000	41

**H6548**	chr6	31961236-31961237	A	C	ATG->CTG	**SKIV2L**	Met214Leu; Met56Leu; Met21Leu	rs437179	0,772	64	40	62,5	151	0	0,0	0,000	13
**H6548**	chr6	25776720-25776721	G	A	GCC->ACC	**SLC17A4**	Ala318Thr; Ala372Thr; Ala142Thr;	rs11754288	0,363	119	46	38,7	184	1	0,5	0,000	11
**H6548**	chr1	53088081-53088082	T	C	CAG->CGG; CAG->CGG	**SLC1A7**	Gln190Arg; Gln537Arg; Gln596Arg;	rs1288401	0,586	132	54	40,9	103	0	0,0	0,000	6
**H6548**	chr6	160139848-160139849	A	-C^ATG		**SLC22A1**	Met420	rs202220802	0,153	76	26	34,2	156	0	0,0	0,000	30
**H6548**	chr1	116037309-116037310	C	T	CGG->TGG	**SLC22A15**	Arg365Trp	rs767605411	0,000	104	26	25,0	142	1	0,7	0,000	0
**H6548**	chr11	65214381-65214382	G	T	ATG->ATT	**SLC22A20**	Met146Ile	rs239259	0,379	105	66	62,9	103	0	0,0	0,000	0
**H6548**	chr20	19280978-19280979	G	A	GTA->ATA	**SLC24A3**	Val55Ile	rs1569767	0,439	73	20	27,4	130	0	0,0	0,000	12
**H6548**	chr19	6427469-6427470	G	T	ACG->AAG	**SLC25A41**	Thr219Lys; Thr81Lys	rs191002996	0,010	112	33	29,5	100	0	0,0	0,000	0
**H6548**	chr5	149981657-149981658	A	T	ACT->TCT	**SLC26A2**	Thr689Ser	rs3776070	0,167	88	49	55,7	115	0	0,0	0,000	5
**H6548**	chr8	91351836-91351837	G	A	GTC->ATC	**SLC26A7**	Val390Ile; Val89Ile	rs371678026	0,000	64	26	40,6	148	0	0,0	0,000	4
**H6548**	chr6	35955468-35955469	T	C	ATT->GTT	**SLC26A8**	Ile221Val; Ile534Val; Ile639Val	rs2295852	0,348	66	26	39,4	168	0	0,0	0,000	3
**H6548**	chr5	128966191-128966192	T	G	TTG->GTG	**SLC27A6**	Leu19Val	rs2526247	0,296	47	19	40,4	143	0	0,0	0,000	13
**H6548**	chr12	7814361-7814362	C	T	GGG->GAG	**SLC2A14**	Gly506Glu; Gly483Glu; Gly397Glu;	rs10845981	0,263	42	25	59,5	79	0	0,0	0,000	25

**H6548**	chr9	127403693-127403694	T	C	ATC->ACC	**SLC2A8**	Ile253Thr; Ile90Thr	rs34064803	0,045	159	56	35,2	260	0	0,0	0,000	10
**H6548**	chr4	9920542-9920543	C	T	GTT->ATT	**SLC2A9**	Val282Ile; Val253Ile	rs16890979	0,240	136	37	27,2	256	0	0,0	0,000	18
**H6548**	chr5	177386244-177386245	G	A	CGC->CAC	**SLC34A1**	Arg95His	rs145798898	0,003	298	127	42,6	345	2	0,6	0,000	5
**H6548**	chr18	11610332-11610333	G	T	TTG->TTT	**SLC35G4**	Leu246Phe	rs62084101	0,386	35	11	31,4	56	0	0,0	0,002	0
**H6548**	chr17	7482134-7482135	G	A	GCT->ACT	**SLC35G6**	Ala51Thr	rs3760422	0,183	226	71	31,4	186	0	0,0	0,000	0
**H6548**	chr21	42565847-42565848	A	T	AGC->TGC	**SLC37A1**	Ser415Cys	rs117821416	0,006	81	28	34,6	159	0	0,0	0,000	8
**H6548**	chr10	17953381-17953382	A	G	AGC->GGC	**SLC39A12**	Ser36Gly	rs10764176	0,276	118	43	36,4	264	0	0,0	0,000	12
**H6548**	chr11	47410151-47410152	A	G	ACT->GCT	**SLC39A13**	Thr20Ala	rs61897432	0,110	199	71	35,7	139	0	0,0	0,000	0
**H6548**	chr6	31871531-31871532	C	T	GTT->ATT	**SLC44A4**	Val111Ile; Val187Ile; Val145Ile	rs2242665	0,599	113	51	45,1	173	0	0,0	0,000	24
**H6548**	chr5	1244309-1244310	C	T	CCG->CTG	**SLC6A18**	Pro478Leu	rs4073918	0,715	123	68	55,3	174	0	0,0	0,000	5
**H6548**	chr11	20601428-20601429	G	A	GGC->AGC	**SLC6A5**	Gly102Ser	rs1443547	0,407	366	111	30,3	220	0	0,0	0,000	2
**H6548**	chr19	33211352-33211353	A	G	TGG->CGG	**SLC7A10**	Trp35Arg	rs61759827	0,121	122	53	43,4	204	0	0,0	0,000	8
**H6548**	chr3	112182251-112182252	T	C	ATG->GTG	**SLC9C1**	Met107Val; Met844Val; Met796Val	rs74840030	0,046	104	33	31,7	96	0	0,0	0,000	0

**H6548**	chr5	102296056-102296057	T	C	AAT->AGT	**SLCO4C1**	Asn69Ser	rs76381196	0,038	231	61	26,4	274	0	0,0	0,000	18
**H6548**	chr8	69832576-69832577	G	A	CTC->TTC	**SLCO5A1**	Leu33Phe	rs3750266	0,451	248	159	64,1	235	0	0,0	0,000	26
**H6548**	chr17	35353791-35353792	C	A	CGC->CTC	**SLFN11**	Arg489Leu	rs9898983	0,270	24	10	41,7	65	0	0,0	0,000	73
**H6548**	chr17	35554698-35554699	G	A	CCC->TCC	**SLFN14**	Pro356Ser	rs321613	0,589	51	15	29,4	47	0	0,0	0,001	0
**H6548**	chr1	41020572-41020573	C	T	GCA->ACA	**SLFNL1**	Ala30Thr	rs1138293	0,151	187	60	32,1	228	0	0,0	0,000	0
**H6548**	chr10	97059559-97059560	A	G	TGC->CGC	**SLIT1**	Cys339Arg	rs2817676	0,677	114	30	26,3	179	0	0,0	0,000	25
**H6548**	chr9	2191308-2191309	C	G	GAC->GAG	**SMARCA2**	Asp1546Glu;<br>Asp1470Glu;<br>Asp1528Glu;	rs2296212	0,171	71	26	36,6	89	0	0,0	0,000	44
**H6548**	chr12	14806456-14806457	T	C	AAA->AGA	**SMCO3**	Lys75Arg	rs2241221	0,158	76	27	35,5	98	0	0,0	0,000	0
**H6548**	chr17	17423289-17423290	C	T	GCA->GTA	**SMCR9**	Ala140Val	rs7225976	0,474	91	26	28,6	224	1	0,4	0,000	0
**H6548**	chr17	2299880-2299881	C	G	CGA->CCA	**SMG6**	Arg291Pro;<br>Arg260Pro	rs1885986	0,289	311	200	64,3	238	0	0,0	0,000	13
**H6548**	chr22	31095345-31095346	C	T	GCG->GTG	**SMTN**	Ala559Val;<br>Ala644Val;<br>Ala615Val;	rs5997872	0,150	51	13	25,5	81	2	2,5	0,000	20
**H6548**	chr17	4592965-4592966	C	A	TTC->TTA	**SMTNL2**	Phe31Leu;<br>Phe175Leu	rs145126598	0,002	235	66	28,1	355	1	0,3	0,000	0
**H6548**	chr10	86962640-86962641	A	T	GAG->GTG	**SNCG**	Glu110Val	rs9864	0,236	84	21	25,0	133	0	0,0	0,000	69

**H6548**	chr2	241071914-241071915	C	A	CCA->CAA	**SNED1**	Pro112Gln	rs6746124	0,564	299	119	39,8	156	0	0,0	0,000	0
**H6548**	chr6	7590390-7590391	C	T	CCC->CTC	**SNRNP48**	Pro45Leu	rs2757594	0,349	206	69	33,5	173	2	1,2	0,000	0
**H6548**	chr20	45840650-45840651	G	A	GCC->ACC; TCG->TCA	**SNX21**	Ala154Thr; Ser151Ser; Ser157Ser;	rs4638862	0,474	273	93	34,1	223	0	0,0	0,000	0
**H6548**	chr16	12524755-12524756	G	A	GTC->ATC	**SNX29**	Val745Ile	rs556538290	0,000	100	34	34,0	199	0	0,0	0,000	37
**H6548**	chr12	53103617-53103618	A	G	GAA->GGA	**SOAT2**	Glu14Gly	rs9658625	0,113	244	72	29,5	122	0	0,0	0,000	10
**H6548**	chr17	38395936-38395937	A	G	AAA->AGA	**SOCS7**	Lys572Arg; Lys538Arg	rs55687042	0,031	43	16	37,2	93	0	0,0	0,000	34
**H6548**	chr8	22571194-22571195	A	G	ACC->GCC	**SORBS3**	Thr573Ala; Thr231Ala	rs1047030	0,181	112	28	25,0	41	0	0,0	0,000	18
**H6548**	chr4	7728429-7728430	G	A	GTG->ATG	**SORCS2**	Val984Met; Val812Met	rs750057469	0,000	89	27	30,3	122	0	0,0	0,000	14
**H6548**	chr2	230212396-230212397	G	A	GCG->GTG	**SP110**	Ala206Val; Ala212Val	rs28930679	0,209	52	13	25,0	98	0	0,0	0,000	30
**H6548**	chr2	230393434-230393435	C	T	CCA->TCA	**SP140L**	Pro317Ser; Pro377Ser; Pro352Ser	rs7590429	0,461	63	21	33,3	110	0	0,0	0,000	0
**H6548**	chr2	173956021-173956022	T	C	ACA->GCA	**SP3**	Thr111Ala; Thr164Ala; Thr161Ala	rs1047640	0,132	112	33	29,5	195	0	0,0	0,000	22
**H6548**	chrX	141241571-141241572	A	T	CTC->CAC	**SPANXE**	Leu80His	rs139422549	0,001	23	16	69,6	17	0	0,0	0,000	0
**H6548**	chrX	143720597-143720598	T	-A^T		**SPANXN2**	Asn24	rs782748787	0,008	40	17	42,5	82	0	0,0	0,000	0

**H6548**	chr1	84544210-84544211	A	G	AAA->GAA	**SPATA1**	Lys243Glu	rs12143652	0,317	87	39	44,8	161	0	0,0	0,000	0
**H6548**	chr3	173117291-173117292	C	T	GGG->GAG	**SPATA16**	Gly147Glu	rs16846616	0,129	100	39	39,0	168	0	0,0	0,000	3
**H6548**	chr11	133844626-133844627	G	A	GCT->GTT	**SPATA19**	Ala50Val	rs2282602	0,381	98	55	56,1	138	0	0,0	0,000	26
**H6548**	chr16	89696854-89696855	T	C	AGC->GGC	**SPATA2L**	Ser142Gly	rs462769	0,395	215	71	33,0	200	0	0,0	0,000	0
**H6548**	chr9	42189006-42189007	C	T	CCT->CTT	**SPATA31A6**	Pro1102Leu	rs10907643	0,512	99	50	50,5	323	0	0,0	0,000	0
**H6548**	chr4	176184858-176184859	T	-T^TCTC		**SPATA4**	Glu280	rs28381989	0,090	68	22	32,4	125	0	0,0	0,000	5
**H6548**	chr14	88396184-88396185	G	A	GTG->ATG	**SPATA7**	Val74Met; Val42Met	rs3179969	0,350	22	10	45,5	74	0	0,0	0,000	10
**H6548**	chr3	52706165-52706166	C	G	CCC->GCC	**SPCS1**	Pro41Ala	rs6617	0,422	175	49	28,0	136	0	0,0	0,000	0
**H6548**	chr5	35659136-35659137	G	A	AGA->AAA	**SPEF2**	Arg366Lys	rs16902381	0,053	76	22	29,0	92	0	0,0	0,000	6
**H6548**	chr2	219488809-219488810	C	A	CCC->ACC	**SPEG**	Pro2687Thr	rs13026308	0,149	133	45	33,8	183	0	0,0	0,000	6
**H6548**	chr5	148120327-148120328	G	T	GAG->GAT	**SPINK5**	Glu806Asp; Glu825Asp	rs2303070	0,157	48	24	50,0	61	0	0,0	0,000	12
**H6548**	chr4	1170719-1170720	C	T	TGG->TGA	**SPON2**	Trp223*	rs13140868	0,082	349	118	33,8	276	0	0,0	0,000	61
**H6548**	chr17	45845813-45845814	G	A	CGC->CAC	**SPPL2C**	Arg303His	rs242944	0,620	266	251	94,4	43	0	0,0	-2,000	0

**H6548**	chr5	80320458-80320459	A	G	AAT->GAT	**SPZ1**	Asn82Asp	rs139471643	0,011	65	26	40,0	55	0	0,0	0,000	42
**H6548**	chr5	122020493-122020494	T	A	TTT->TTA	**SRFBP1**	Phe253Leu	rs61734326	0,061	95	33	34,7	114	0	0,0	0,000	0
**H6548**	chr12	64091372-64091373	G	A	GTC->ATC	**SRGAP1**	Val512Ile	rs74691643	0,025	52	14	26,9	77	0	0,0	0,000	41
**H6548**	chrX	38164778-38164779	T	C	ATT->GTT	**SRPX**	Ile215Val;<br>Ile195Val;<br>Ile156Val	rs747875312	0,000	58	30	51,7	61	0	0,0	0,000	49
**H6548**	chr17	29632884-29632885	G	A	TCG->TTG	**SSH2**	Ser770Leu;<br>Ser743Leu	rs2289629	0,274	71	40	56,3	168	0	0,0	0,000	37
**H6548**	chr7	149787709-149787710	C	T	GCG->GTG	**SSPO**	Ala1207Val	rs62490656	0,259	267	108	40,5	206	2	1,0	0,000	8
**H6548**	chr16	1065494-1065495	T	C	AAC->GAC	**SSTR5-AS1**	Asn339Asp	rs213659	0,260	154	62	40,3	119	0	0,0	0,000	0
**H6548**	chrX	52645463-52645464	G	T	GAC->GAA	**SSX7**	Asp182Glu	rs112382781	0,099	26	16	61,5	41	0	0,0	0,000	0
**H6548**	chrX	48303669-48303670	A	G	TGT->CGT	**SSX9**	Cys72Arg	rs4598385	0,466	123	89	72,4	184	0	0,0	0,000	0
**H6548**	chr3	52501749-52501750	C	T	CAT->TAT	**STAB1**	His110Tyr	rs138055453	0,013	109	37	33,9	103	0	0,0	0,000	34
**H6548**	chr7	100180661-100180662	A	C	ACC->CCC	**STAG3**	Thr36Pro	rs2272343	0,177	85	24	28,2	140	2	1,4	0,000	24
**H6548**	chr7	22492640-22492641	A	G	ATA->ACA	**STEAP1B**	Ile210Thr;<br>Ile229Thr	rs16881817	0,305	157	40	25,5	315	0	0,0	0,000	0
**H6548**	chr7	90225131-90225132	T	G	TTT->TGT	**STEAP2**	Phe59Cys;<br>Phe17Cys	rs194520	0,463	130	82	63,1	246	0	0,0	0,000	72

**H6548**	chr7	23772180-23772181	G	T	AGT->ATT	**STK31**	Ser623Ile; Ser600Ile; Ser587Ile	rs10247878	0,120	44	26	59,1	67	0	0,0	0,000	32
**H6548**	chr10	132331648-132331649	G	A	CAC->TAC	**STK32C**	His30Tyr	rs7100410	0,216	222	69	31,1	167	0	0,0	0,000	0
**H6548**	chr2	218697951-218697952	G	A	GGT->GAT	**STK36**	Gly1003Asp; Gly982Asp; Gly48Asp	rs1863704	0,310	89	37	41,6	188	0	0,0	0,000	29
**H6548**	chr1	36341879-36341880	C	T	GCC->ACC	**STK40**	Ala395Thr; Ala400Thr	rs3795498	0,211	270	145	53,7	166	0	0,0	0,000	24
**H6548**	chr1	24383914-24383915	A	G	ATG->ACG	**STPG1**	Met1Thr; Met93Thr; Met46Thr	rs11538189	0,107	54	17	31,5	81	0	0,0	0,000	0
**H6548**	chr15	43605300-43605301	T	C	CAC->CGC	**STRC**	His1298Arg; His525Arg	rs2920780	0,246	96	25	26,0	172	0	0,0	0,000	10
**H6548**	chr19	7640565-7640566	G	A	GCG->ACG	**STXBP2**	Ala189Thr	rs186799855	0,014	93	27	29,0	132	0	0,0	0,000	26
**H6548**	chr20	47736891-47736892	C	T	GCG->ACG	**SULF2**	Ala76Thr	rs56218501	0,140	225	63	28,0	279	1	0,4	0,000	49
**H6548**	chr2	108258741-108258742	G	A	GGG->AGG	**SULT1C3**	Gly179Arg	rs2219078	0,283	80	20	25,0	131	0	0,0	0,000	0
**H6548**	chr7	842030-842031	C	T	CAC->TAC	**SUN1**	His118Tyr; His139Tyr; His68Tyr	rs6461378	0,469	235	80	34,0	331	0	0,0	0,000	16
**H6548**	chr9	112124288-112124289	C	T	GGC->GAC	**SUSD1**	Gly285Asp	rs146879704	0,002	129	37	28,7	144	0	0,0	0,000	0
**H6548**	chr9	93077973-93077974	A	G	AAG->GAG	**SUSD3**	Lys123Glu; Lys136Glu; Lys73Glu	rs1131773	0,190	86	29	33,7	171	0	0,0	0,000	0
**H6548**	chr5	76295884-76295885	C	G	ACT->AGT	**SV2C**	Thr482Ser	rs2270927	0,142	54	19	35,2	80	0	0,0	0,000	9

**H6548**	chr9	110407350-110407351	G	A	GCA->GTA	**SVEP1**	Ala676Val; Ala2750Val	rs7030192	0,363	93	29	31,2	190	0	0,0	0,000	28
**H6548**	chr1	185202736-185202737	A	G	CAC->CGC	**SWT1**	His536Arg	rs6698109	0,317	50	18	36,0	112	0	0,0	0,000	0
**H6548**	chr14	63941926-63941927	A	G	ATA->GTA	**SYNE2**	Ile94Val	rs146748294	0,001	89	34	38,2	163	0	0,0	0,000	17
**H6548**	chr17	37596343-37596344	G	C	GCA->GGA	**SYNRG**	Ala40Gly; Ala73Gly	rs12944821	0,194	62	19	30,7	101	0	0,0	0,000	6
**H6548**	chr1	210094548-210094549	G	-T^GAA		**SYT14**	Glu269; Glu224; Glu186	rs144713062	0,117	78	24	30,8	139	0	0,0	0,000	0
**H6548**	chr4	68062818-68062819	G	A	ACG->ATG	**SYT14L**	Thr97Met	rs3819257	0,240	76	42	55,3	212	2	0,9	0,000	0
**H6548**	chr11	1835113-1835114	C	A	CAC->CAA	**SYT8**	His3Gln; His17Gln	rs57344881	0,066	128	47	36,7	95	0	0,0	0,000	0
**H6548**	chr11	85709357-85709358	G	A	GCG->GTG	**SYTL2**	Ala100Val; Ala60Val; Ala89Val;	rs61740616	0,024	79	24	30,4	146	0	0,0	0,000	18
**H6548**	chr8	38827407-38827408	C	T	CAT->TAT	**TACC1**	His370Tyr	rs781532579	0,000	72	26	36,1	145	0	0,0	0,000	70
**H6548**	chr10	122084656-122084657	G	T	GAG->GAT	**TACC2**	Glu719Asp	rs41288002	0,036	89	29	32,6	241	0	0,0	0,000	67
**H6548**	chr9	32634468-32634469	T	C	ATG->GTG	**TAF1L**	Met371Val	rs17219559	0,070	113	32	28,3	265	0	0,0	0,000	45
**H6548**	chr8	119788865-119788866	G	A	GCA->GTA	**TAF2**	Ala536Val	rs745511347	0,000	46	13	28,3	83	0	0,0	0,000	14
**H6548**	chr1	234467575-234467576	A	T	TCC->ACC	**TARBP1**	Ser392Thr	rs60079444	0,108	68	17	25,0	125	0	0,0	0,000	34

**H6548**	chr7	38260125-38260126	T	C	AGA->GGA	**TARP**	Arg100Gly; Arg300Gly; Arg304Gly	rs138027161	0,440	86	52	60,5	93	2	2,2	0,000	0
**H6548**	chr1	18854520-18854521	G	C	CGC->GGC	**TAS1R2**	Arg317Gly	rs34447754	0,287	369	275	74,5	327	0	0,0	0,000	0
**H6548**	chr12	10997640-10997641	T	C	AAA->GAA	**TAS2R20**	Lys79Glu	rs7135018	0,226	53	14	26,4	98	0	0,0	0,000	0
**H6548**	chr7	143478251-143478252	C	T	CCA->CTA	**TAS2R41**	Pro127Leu	rs10278721	0,260	72	26	36,1	200	0	0,0	0,000	0
**H6548**	chr12	11061545-11061546	C	T	TGG->TAG	**TAS2R46**	Trp250*	rs2708381	0,226	179	58	32,4	329	1	0,3	0,000	12
**H6548**	chr16	71576372-71576373	G	A	CCC->TCC	**TAT**	Pro15Ser	rs74344827	0,151	106	31	29,3	181	0	0,0	0,000	19
**H6548**	chr7	27785235-27785236	C	G	TCC->TGC	**TAX1BP1**	Ser229Cys; Ser72Cys		0,000	40	10	25,0	68	0	0,0	0,004	71
**H6548**	chr11	67409426-67409427	A	G	TCA->TCG; AGA->GGA	**TBC1D10C**	Ser338Ser; Arg274Gly	rs11227788	0,187	125	36	28,8	55	0	0,0	-2,000	16
**H6548**	chrX	48560270-48560271	G	A	GCT->ACT	**TBC1D25**	Ala455Thr; Ala397Thr; Ala201Thr;	rs235836	0,488	155	107	69,0	174	0	0,0	0,000	0
**H6548**	chr6	121256223-121256224	T	C	ATA->GTA	**TBC1D32**	Ile599Val; Ile118Val	rs7745023	0,438	76	21	27,6	129	0	0,0	0,000	0
**H6548**	chr16	1976889-1976890	T	C	TCC->CCC	**TBL3**	Ser457Pro; Ser343Pro; Ser324Pro	rs17605	0,072	157	53	33,8	196	0	0,0	0,000	15
**H6548**	chr14	55436997-55436998	C	A	AGG->AGT	**TBPL2**	Arg57Ser	rs45612333	0,069	33	16	48,5	69	0	0,0	0,000	24
**H6548**	chr18	47034728-47034729	C	G	CGG->CCG	**TCEB3B**	Arg179Pro	rs2571028	0,561	480	228	47,5	194	0	0,0	0,000	0

**H6548**	chr1	152107512-152107513	T	G	AAA->CAA	**TCHH**	Lys1902Gln	rs1131471	0,512	194	50	25,8	310	1	0,3	0,000	19
**H6548**	chr11	68041800-68041801	C	T	CGG->TGG	**TCIRG1**	Arg56Trp	rs36027301	0,062	71	22	31,0	110	0	0,0	0,000	13
**H6548**	chr5	150396668-150396669	C	T	GCA->GTA	**TCOF1**	Ala1390Val; Ala1352Val; Ala1353Val;	rs15251	0,289	80	29	36,3	146	0	0,0	0,000	10
**H6548**	chr6	35120603-35120604	C	G	GGA->GCA	**TCP11**	Gly191Ala; Gly190Ala; Gly261Ala;	rs2234045	0,137	121	34	28,1	204	0	0,0	0,000	10
**H6548**	chrX	102463309-102463310	C	-G^C		**TCP11X2**	Ala154; Ala164	rs782116629	-1,000	74	27	36,5	133	0	0,0	0,000	0
**H6548**	chr14	21869211-21869212	G	A	GGA->AGA	**TCRA**	Gly62Arg	rs876093	0,640	117	58	49,6	150	0	0,0	0,000	
**H6548**	chr13	60528441-60528442	A	-G^ATA		**TDRD3**	Asp138; Asp313; Asp406; Asp312	rs148917045	0,181	58	16	27,6	106	0	0,0	0,000	35
**H6548**	chr7	98217693-98217694	C	T	GGG->AGG	**TECPR1**	Gly1128Arg		0,000	107	48	44,9	60	0	0,0	0,000	10
**H6548**	chr9	27197487-27197488	G	T	GTG->TTG	**TEK**	Val534Leu; Val600Leu; Val557Leu;	rs35030851	0,045	88	22	25,0	126	2	1,6	0,000	20
**H6548**	chr16	1495446-1495447	A	G	CAG->CGG	**TELO2**	Gln146Arg	rs2235624	0,601	242	123	50,8	98	2	2,0	0,000	45
**H6548**	chr12	53061153-53061154	G	A	GGG->GAG	**TENC1**	Gly959Glu; Gly1093Glu; Gly1083Glu;	rs139881378	0,001	224	72	32,1	295	0	0,0	0,000	51
**H6548**	chr5	168262244-168262245	G	A	AGC->AAC	**TENM2**	Ser2578Asn; Ser2348Asn; Ser1951Asn;	rs3208478	0,004	143	46	32,2	155	0	0,0	0,000	0
**H6548**	chr14	20384657-20384658	G	A	CGT->TGT	**TEP1**	Arg405Cys; Arg1055Cys; Arg947Cys	rs1760903	0,536	111	28	25,2	208	0	0,0	0,000	56

**H6548**	chr1	45345192-45345193	G	A	CGT->TGT	**TESK2**	Arg426Cys; Arg455Cys; Arg372Cys;	rs17853159	0,051	135	36	26,7	299	0	0,0	0,000	26
**H6548**	chr10	68572822-68572823	A	G	GAC->GGC	**TET1**	Asp162Gly	rs10823229	0,326	58	15	25,9	133	0	0,0	0,000	44
**H6548**	chr4	105235005-105235006	G	A	GGT->GAT	**TET2**	Gly355Asp; Gly376Asp	rs61744960	0,026	76	20	26,3	103	1	1,0	0,000	70
**H6548**	chr2	74047553-74047554	C	T	ACC->ATC	**TET3**	Thr411Ile; Thr546Ile	rs183678709	0,002	93	25	26,9	130	0	0,0	0,000	35
**H6548**	chr8	30844478-30844479	A	C	GAT->GAG	**TEX15**	Asp1513Glu	rs61732457	0,072	167	55	32,9	252	0	0,0	0,000	19
**H6548**	chr17	82362172-82362173	G	A	CGG->CAG	**TEX19**	Arg8Gln	rs143841873	0,000	50	17	34,0	139	0	0,0	0,000	21
**H6548**	chr3	51703948-51703949	G	A	GGG->GAG	**TEX264**	Gly292Glu; Gly112Glu; Gly218Glu	rs11553574	0,116	119	36	30,3	147	0	0,0	0,000	0
**H6548**	chr1	246565948-246565949	C	T	GCG->ACG	**TFB2M**	Ala64Thr	rs143880306	0,005	104	28	26,9	195	0	0,0	0,000	9
**H6548**	chr6	41691150-41691151	C	T	CAG->CAA; CAG->CAA	**TFEB**	Gln88Gln; Gln21Gln; Gln35Gln; Ala65Thr	rs1015149	0,520	179	122	68,2	117	0	0,0	0,000	38
**H6548**	chr18	3457608-3457609	C	T	CCG->CTG	**TGIF1**	Pro143Leu; Pro177Leu; Pro166Leu;	rs2229333	0,063	60	20	33,3	120	0	0,0	0,000	37
**H6548**	chr20	2317143-2317144	G	A	AGC->AAC	**TGM3**	Ser249Asn	rs214814	0,112	57	15	26,3	130	0	0,0	0,000	30
**H6548**	chr3	44907097-44907098	G	A	GTA->ATA	**TGM4**	Val409Ile	rs9876921	0,555	110	29	26,4	212	0	0,0	0,000	32
**H6548**	chr11	2169720-2169721	C	T	GTG->ATG	**TH**	Val108Met; Val112Met; Val81Met;	rs6356	0,425	271	73	26,9	162	0	0,0	0,000	23

**H6548**	chr4	82917108-82917109	G	T	TTG->TTT	**THAP9**	Leu155Phe; Leu299Phe; Leu16Phe	rs897945	0,487	142	86	60,6	204	0	0,0	0,000	0
**H6548**	chr2	137663425-137663426	A	G	AAA->AGA	**THSD7B**	Lys1471Arg	rs10206850	0,623	80	29	36,3	155	0	0,0	0,000	0
**H6548**	chr19	3728610-3728611	G	A	CGC->CAC	**TJP3**	Arg19His; Arg28His; Arg38His	rs2067019	0,267	104	32	30,8	310	1	0,3	0,000	19
**H6548**	chr4	163471964-163471965	T	A	CAA->CAT	**TKTL2**	Gln590His	rs11735477	0,171	114	29	25,4	206	1	0,5	0,000	0
**H6548**	chrX	12885539-12885540	A	T	CAA->CTA	**TLR7**	Gln11Leu	rs179008	0,177	212	198	93,4	67	0	0,0	-2,000	29
**H6548**	chr3	52221727-52221728	C	T	CGA->CAA	**TLR9**	Arg863Gln; Arg960Gln	rs5743845	0,010	368	116	31,5	318	0	0,0	0,000	30
**H6548**	chr15	83112846-83112847	T	G	CTG->CGG	**TM6SF1**	Leu48Arg	rs79470022	0,045	94	27	28,7	157	0	0,0	0,000	0
**H6548**	chr20	32141815-32141816	G	A	CGA->CAA	**TM9SF4**	Arg76Gln; Arg150Gln; Arg57Gln;	rs117822199	0,007	113	32	28,3	263	0	0,0	0,000	48
**H6548**	chr15	81332686-81332687	C	T	AGA->AAA	**TMC3**	Arg1011Lys	rs8031046	0,085	153	49	32,0	322	0	0,0	0,000	0
**H6548**	chr19	54160471-54160472	C	G	GAG->CAG	**TMC4**	Glu403Gln; Glu689Gln; Glu683Gln	rs36657	0,498	77	36	46,8	157	0	0,0	0,000	0
**H6548**	chr17	78124728-78124729	G	A	CCG->CTG	**TMC6**	Pro229Leu; Pro62Leu; Pro2Leu	rs749326103	0,000	312	80	25,6	107	0	0,0	0,000	41
**H6548**	chr17	55720778-55720779	A	G	TTT->CTT	**TMEM100**	Phe98Leu	rs141365411	0,000	104	26	25,0	210	0	0,0	0,000	38
**H6548**	chr12	47966200-47966201	C	T	TCC->TTC	**TMEM106C**	Ser175Phe	rs2286025	0,152	76	27	35,5	122	1	0,8	0,000	0

**H6548**	chr12	128415127-128415128	A	G	CAC->CGC	**TMEM132C**	His161Arg	rs11059681	0,481	256	169	66,0	300	0	0,0	0,000	0
**H6548**	chr5	73123789-73123790	C	A	AAC->AAA	**TMEM171**	Asn139Lys	rs636926	0,328	143	43	30,1	307	0	0,0	0,000	0
**H6548**	chr4	948217-948218	G	A	CGA->CAA	**TMEM175**	Arg160Gln	rs2290403	0,329	151	47	31,1	147	0	0,0	0,000	21
**H6548**	chr7	150793995-150793996	T	G	AGT->CGT	**TMEM176B**	Ser94Arg	rs3173833	0,558	82	23	28,1	183	0	0,0	0,000	32
**H6548**	chr14	104594294-104594295	T	C	CAT->CGT	**TMEM179**	His208Arg	rs3803315	0,396	121	74	61,2	201	0	0,0	0,000	0
**H6548**	chr3	149982731-149982732	T	C	ATG->GTG	**TMEM183B**	Met193Val	rs7630407	0,404	175	74	42,3	314	0	0,0	0,000	0
**H6548**	chr12	63780026-63780027	G	A	GCT->ACT	**TMEM5**	Ala23Thr	rs61935924	0,088	445	141	31,7	435	0	0,0	0,000	11
**H6548**	chr1	1918483-1918484	C	A	GCA->TCA	**TMEM52**	Ala25Ser	rs72634869	0,078	230	84	36,5	188	0	0,0	0,000	0
**H6548**	chr1	156285664-156285665	G	A	GTG->ATG	**TMEM79**	Val147Met	rs6684514	0,266	472	162	34,3	316	0	0,0	0,000	27
**H6548**	chr4	67963390-67963391	C	T	ATG->ATA	**TMPRSS11A**	Met1Ile	rs977728	0,207	55	28	50,9	81	0	0,0	0,000	63
**H6548**	chr12	50844032-50844033	G	A	GCT->ACT	**TMPRSS12**	Ala127Thr	rs861204	0,329	64	43	67,2	107	0	0,0	0,000	0
**H6548**	chr20	7982393-7982394	C	T	GGA->AGA	**TMX4**	Gly303Arg	rs2076015	0,175	138	68	49,3	201	0	0,0	0,000	0
**H6548**	chr9	115052756-115052757	G	A	ACT->ATT	**TNC**	Thr1249Ile	rs62578424	0,126	122	34	27,9	173	0	0,0	0,000	16

**H6548**	chr18	62354527-62354528	C	T	CAC->TAC	**TNFRSF11A**	His141Tyr	rs35211496	0,118	434	215	49,5	233	0	0,0	0,000	58
**H6548**	chr1	2559765-2559766	C	T	CGT->TGT	**TNFRSF14**	Arg253Cys	rs2234161	0,658	264	82	31,1	196	0	0,0	0,000	82
**H6548**	chr5	151056860-151056861	C	T	GGC->AGC	**TNIP1**	Gly125Ser;<br>Gly178Ser	rs52817781	0,028	364	106	29,1	311	0	0,0	0,000	41
**H6548**	chr3	195868078-195868079	G	A	CCG->CTG	**TNK2**	Pro132Leu;<br>Pro250Leu;<br>Pro757Leu;	rs56260729	0,223	513	146	28,5	221	2	0,9	0,000	53
**H6548**	chr2	217818415-217818416	G	A	CCC->TCC	**TNS1**	Pro1189Ser;<br>Pro1181Ser;<br>Pro1202Ser	rs34291329	0,077	250	90	36,0	210	0	0,0	0,000	28
**H6548**	chr6	32052734-32052735	T	C	AAG->AGG	**TNXB**	Lys3015Arg	rs17207895	0,022	337	86	25,5	324	0	0,0	0,000	18
**H6548**	chr8	144436291-144436292	G	A	GCC->GTC	**TONSL**	Ala714Val	rs7830832	0,459	194	63	32,5	181	0	0,0	0,000	37
**H6548**	chr22	21964009-21964010	C	T	GAA->AAA	**TOP3B**	Glu373Lys	rs752506133	0,000	166	62	37,4	200	0	0,0	0,000	30
**H6548**	chr9	129818621-129818622	C	G	GAT->CAT	**TOR1A**	Asp216His	rs1801968	0,121	96	32	33,3	227	0	0,0	0,000	3
**H6548**	chr15	43475575-43475576	G	C	GAC->GAG	**TP53BP1**	Asp353Glu;<br>Asp358Glu	rs560191	0,380	74	42	56,8	113	0	0,0	0,000	88
**H6548**	chr2	1456231-1456232	G	T	GCT->TCT	**TPO**	Ala257Ser	rs4927611	0,379	98	32	32,7	113	0	0,0	0,000	22
**H6548**	chr9	137199558-137199559	G	T	CCC->ACC	**TPRN**	Pro385Thr	rs761993178	0,000	249	87	34,9	159	0	0,0	0,000	0
**H6548**	chr19	47802393-47802394	G	A	CCG->CTG	**TPRX1**	Pro206Leu	rs62130758	0,305	25	11	44,0	29	0	0,0	0,001	0

**H6548**	chr16	1241595-1241596	C	G	AAC->AAG	**TPSAB1**	Asn132Lys; Asn139Lys; Asn68Lys	rs144979264	0,445	20	12	60,0	38	0	0,0	0,000	15
**H6548**	chr16	1222749-1222750	G	A	TCC->TTC	**TPSG1**	Ser138Phe	rs113856625	0,052	74	31	41,9	51	0	0,0	0,000	0
**H6548**	chr16	3671771-3671772	G	C	GAC->GAG	**TRAP1**	Asp186Glu; Asp395Glu; Asp342Glu	rs1136948	0,151	258	69	26,7	304	0	0,0	0,000	54
**H6548**	chr5	65629725-65629726	T	A	TCC->ACC	**TRAPPC13**	Ser47Thr	rs17830337	0,050	109	28	25,7	159	0	0,0	0,000	0
**H6548**	chr18	31917583-31917584	A	T	TTA->ATA	**TRAPPC8**	Leu146Ile; Leu92Ile	rs201756494	0,001	60	15	25,0	151	0	0,0	0,000	0
**H6548**	chr11	118659901-118659902	T	C	ACA->GCA	**TREH**	Thr389Ala; Thr358Ala; Thr266Ala	rs2276065	0,249	224	62	27,7	279	0	0,0	0,000	12
**H6548**	chr7	28956940-28956941	C	T	GGC->GAC	**TRIL**	Gly369Asp	rs3735562	0,313	247	89	36,0	147	0	0,0	0,000	12
**H6548**	chr6	30171921-30171922	G	A	AGC->AAC	**TRIM15**	Ser324Asn	rs929156	0,276	267	84	31,5	213	0	0,0	0,000	45
**H6548**	chr17	18727681-18727682	G	A	AGG->AAG	**TRIM16L**	Arg96Lys; Arg42Lys; Arg144Lys	rs8075739	0,349	99	39	39,4	205	0	0,0	0,000	0
**H6548**	chr6	30147764-30147765	G	A	GAG->AAG	**TRIM40**	Glu244Lys; Glu215Lys	rs757259	0,209	65	24	36,9	89	0	0,0	0,000	0
**H6548**	chr11	55885687-55885688	G	A	AGC->AAC	**TRIM51**	Ser87Asn	rs10897403	0,420	93	40	43,0	181	0	0,0	0,000	0
**H6548**	chr4	165041337-165041338	A	T	GAA->GAT	**TRIM60**	Glu422Asp	rs77293967	0,011	87	22	25,3	155	0	0,0	0,000	0
**H6548**	chr17	75892057-75892058	C	T	GGG->GAG	**TRIM65**	Gly291Glu	rs762217267	0,000	184	59	32,1	245	0	0,0	0,000	20

**H6548**	chr11	8620480-8620481	G	A	CGG->TGG	**TRIM66**	Arg1037Trp	rs138444298	0,004	77	22	28,6	167	0	0,0	0,000	0
**H6548**	chr15	44756452-44756453	C	T	GCT->GTT	**TRIM69**	Ala190Val; Ala31Val	rs3759880	0,125	44	16	36,4	91	0	0,0	0,000	34
**H6548**	chr5	181204827-181204828	G	C	CAG->GAG	**TRIM7**	Gln95Glu	rs2770945	0,144	166	56	33,7	64	0	0,0	-2,000	19
**H6548**	chrX	54925030-54925031	C	T	CCA->CTA	**TRO**	Pro483Leu; Pro86Leu; Pro14Leu	rs45448595	0,062	40	14	35,0	112	0	0,0	0,000	17
**H6548**	chr8	72071805-72071806	C	G	AGA->ACA	**TRPA1**	Arg58Thr	rs16937976	0,135	46	12	26,1	72	0	0,0	0,000	9
**H6548**	chr15	31002450-31002451	C	T	GTT->ATT	**TRPM1**	Val1302Ile; Val1417Ile; Val1395Ile;	rs3784588	0,079	102	26	25,5	256	0	0,0	0,000	23
**H6548**	chr19	49167949-49167950	G	A	GCA->ACA	**TRPM4**	Ala101Thr	rs113984787	0,021	175	51	29,1	231	0	0,0	0,000	13
**H6548**	chr17	3592170-3592171	C	T	CCG->CCA; CCG->CCA	**TRPV1**	Pro60Pro; Arg35Gln	rs56095209	0,002	127	41	32,3	156	0	0,0	0,000	10
**H6548**	chr9	132911516-132911517	A	G	ATG->ACG	**TSC1**	Met322Thr; Met271Thr; Met201Thr	rs1073123	0,129	59	31	52,5	124	0	0,0	0,000	81
**H6548**	chr13	44574120-44574121	G	A	CCT->TCT	**TSC22D1**	Pro652Ser	rs9525983	0,090	142	36	25,4	380	0	0,0	0,000	54
**H6548**	chr17	75524277-75524278	C	G	CCA->GCA	**TSEN54**	Pro483Ala	rs62088470	0,058	65	19	29,2	159	2	1,3	0,000	6
**H6548**	chr12	57791986-57791987	A	T	AAC->ATC	**TSFM**	Asn169Ile		0,000	43	14	32,6	37	0	0,0	0,004	16
**H6548**	chr18	75286943-75286944	G	A	GCG->ACG	**TSHZ1**	Ala468Thr	rs33930274	0,032	153	45	29,4	147	0	0,0	0,000	10

**H6548**	chr11	76796647-76796648	T	A	GAT->GAA	**TSKU**	Asp344Glu	rs1149621	0,809	154	88	57,1	86	0	0,0	-2,000	0
**H6548**	chr8	142344358-142344359	T	C	ACT->GCT	**TSNARE1**	Thr118Ala	rs10100935	0,456	342	137	40,1	262	0	0,0	0,000	0
**H6548**	chr17	81645606-81645607	T	C	TAC->CAC	**TSPAN10**	Tyr256His; Tyr218His	rs34379910	0,055	200	57	28,5	209	0	0,0	0,000	39
**H6548**	chrX	38563056-38563057	G	A	GAT->AAT	**TSPAN7**	Asp15Asn	rs372929587	0,001	88	56	63,6	94	0	0,0	0,000	23
**H6548**	chr6	116279646-116279647	G	A	CCA->TCA	**TSPYL1**	Pro62Ser	rs3828743	0,325	226	73	32,3	298	0	0,0	0,000	12
**H6548**	chr11	2403310-2403311	C	G	CAC->CAG	**TSSC4**	His162Gln; His226Gln	rs2234279	0,065	250	91	36,4	240	0	0,0	0,000	49
**H6548**	chr15	42878594-42878595	A	G	CTG->CCG	**TTBK2**	Leu8Pro	rs6493068	0,384	114	35	30,7	110	0	0,0	0,000	18
**H6548**	chr2	165941135-165941136	C	T	GTG->ATG	**TTC21B**	Val201Met	rs1432273	0,657	108	27	25,0	196	0	0,0	0,000	16
**H6548**	chr1	54801123-54801124	G	C	CTC->GTC	**TTC22**	Leu14Val	rs671108	0,197	325	115	35,4	253	0	0,0	0,000	0
**H6548**	chr2	32736790-32736791	A	G	TAT->TGT	**TTC27**	Tyr476Cys; Tyr426Cys	rs2273660	0,117	172	44	25,6	290	1	0,3	0,000	0
**H6548**	chr21	37121972-37121973	C	G	CTA->GTA	**TTC3**	Leu353Val; Leu43Val; Leu118Val	rs61999340	0,049	87	32	36,8	87	0	0,0	-2,000	16
**H6548**	chr5	95525413-95525414	G	C	CTA->GTA	**TTC37**	Leu437Val; Leu389Val	rs17084873	0,195	69	22	31,9	114	0	0,0	0,000	5
**H6548**	chr10	132836204-132836205	C	T	GGC->AGC	**TTC40**	Gly2184Ser	rs76581191	0,011	155	48	31,0	124	0	0,0	0,000	0

**H6548**	chr14	37804748-37804749	G	A	GCA->ACA	**TTC6**	Ala97Thr	rs17768654	0,038	112	31	27,7	174	0	0,0	0,000	0
**H6548**	chr1	1184996-1184997	T	A	ATG->AAG	**TTLL10**	Met357Lys; Met430Lys	rs116321663	0,018	158	59	37,3	253	0	0,0	0,000	0
**H6548**	chr2	218737775-218737776	G	C	GAG->CAG	**TTLL4**	Glu34Gln	rs3731877	0,437	104	43	41,4	212	0	0,0	0,000	7
**H6548**	chr2	178556966-178556967	A	G	ATC->ACC	**TTN**	Ile29396Thr; Ile27755Thr; Ile26828Thr;	rs9808377	0,357	94	46	48,9	202	0	0,0	0,000	11
**H6548**	chr17	59886175-59886176	A	G	ATG->ACG	**TUBD1**	Met76Thr; Met41Thr	rs1292053	0,448	62	18	29,0	68	0	0,0	0,000	0
**H6548**	chr22	50240190-50240191	G	C	TTC->TTG	**TUBGCP6**	Phe306Leu	rs17248329	0,021	42	15	35,7	63	0	0,0	0,000	31
**H6548**	chrX	16829641-16829642	A	G	ATT->GTT	**TXLNG**	Ile246Val; Ile114Val	rs5969783	0,022	109	89	81,7	75	0	0,0	-2,000	0
**H6548**	chr22	19880732-19880733	G	-A^G		**TXNRD2**	Pro7	rs34827823	0,150	132	54	40,9	128	0	0,0	0,000	33
**H6548**	chr22	50525806-50525807	G	A	TCG->TTG	**TYMP**	Ser476Leu; Ser471Leu	rs11479	0,140	336	110	32,7	215	0	0,0	0,000	34
**H6548**	chr21	42409584-42409585	G	A	GTG->ATG	**UBASH3A**	Val111Met	rs141421753	0,000	90	25	27,8	149	0	0,0	0,000	9
**H6548**	chr17	76391373-76391374	C	T	GCC->ACC	**UBE2O**	Ala754Thr; Ala1150Thr	rs34070229	0,010	296	99	33,5	260	0	0,0	0,000	41
**H6548**	chr20	3110295-3110296	A	G	CCT->CCC; CTG->CCG	**UBOX5**	Pro477Pro; Leu479Pro; Leu425Pro	rs34205880	0,063	117	34	29,1	142	0	0,0	0,000	0
**H6548**	chr10	5373990-5373991	A	G	AGA->GGA	**UCN3**	Arg91Gly	rs10904481	0,379	177	51	28,8	204	0	0,0	0,000	11

**H6548**	chr11	18578735-18578736	T	C	ATG->GTG	**UEVLD**	Met1Val; Met39Val	rs61752314	0,021	97	30	30,9	197	0	0,0	0,000	0
**H6548**	chr2	233682322-233682323	A	G	AAT->AGT	**UGT1A7**	Asn129Ser	rs56385016	0,008	70	18	25,7	169	0	0,0	0,000	34
**H6548**	chr4	69647054-69647055	A	G	TTA->TCA	**UGT2A1**	Leu197Ser	rs41292307	0,136	128	37	28,9	191	0	0,0	0,000	14
**H6548**	chr19	4954442-4954443	G	A	GCG->ACG	**UHRF1**	Ala638Thr; Ala253Thr; Ala651Thr	rs17883331	0,166	316	85	26,9	265	0	0,0	0,000	57
**H6548**	chr5	176955993-176955994	G	A	CCA->CTA	**UIMC1**	Pro433Leu; Pro65Leu	rs3733876	0,141	56	16	28,6	118	0	0,0	0,000	56
**H6548**	chr6	150068981-150068982	C	T	GCC->ACC	**ULBP3**	Ala29Thr	rs113233347	0,111	155	60	38,7	167	1	0,6	0,000	61
**H6548**	chr3	41954643-41954644	T	C	AAA->AGA	**ULK4**	Lys39Arg	rs2272007	0,783	103	30	29,1	184	2	1,1	0,000	28
**H6548**	chr17	75832821-75832822	G	A	ACG->ATG	**UNC13D**	Thr514Met	rs112267124	0,225	90	27	30,0	157	0	0,0	0,000	25
**H6548**	chr1	215838099-215838100	C	T	GAA->AAA	**USH2A**	Glu3088Lys	rs56056328	0,006	45	16	35,6	50	0	0,0	0,000	3
**H6548**	chr1	62450618-62450619	A	G	ATT->GTT	**USP1**	Ile666Val	rs141001844	0,003	86	31	36,1	159	0	0,0	0,000	41
**H6548**	chr8	12137447-12137448	T	C	AAA->AGA	**USP17L2**	Lys438Arg	rs12543578	0,455	119	39	32,8	119	0	0,0	0,000	66
**H6548**	chrX	133027130-133027131	G	A	CTT->TTT	**USP26**	Leu364Phe	rs35397110	0,038	31	21	67,7	59	0	0,0	0,000	16
**H6548**	chr6	99446209-99446210	C	G	AGA->ACA	**USP45**	Arg201Thr; Arg521Thr	rs41288947	0,274	78	21	26,9	142	0	0,0	0,000	9

**H6548**	chr15	50477322-50477323	G	A	GCT->ACT	**USP8**	Ala348Thr; Ala49Thr; Ala271Thr	rs61733869	0,025	61	19	31,2	61	0	0,0	0,000	42
**H6548**	chr13	52029759-52029760	G	A	CGC->CAC	**UTP14C**	Arg319His	rs17402034	0,065	91	31	34,1	158	0	0,0	0,000	0
**H6548**	chr6	31795066-31795067	G	T	CCC->ACC	**VARS**	Pro51Thr	rs2753960	0,453	192	100	52,1	116	0	0,0	0,000	4
**H6548**	chr6	30925650-30925651	G	A	GCA->ACA	**VARS2**	Ala965Thr; Ala825Thr; Ala995Thr;	rs2252863	0,325	120	33	27,5	177	0	0,0	0,000	19
**H6548**	chr12	47879111-47879112	A	G	ATG->ACG	**VDR**	Met1Thr; Met51Thr	rs2228570	0,639	53	28	52,8	86	0	0,0	0,000	30
**H6548**	chr6	132724672-132724673	G	A	ACA->ATA	**VNN3**	Thr87Ile; Thr121Ile	rs2294758	0,365	65	22	33,9	104	0	0,0	0,000	27
**H6548**	chr8	99193012-99193013	C	T	TCC->TTC	**VPS13B**	Ser824Phe; Ser295Phe	rs145419141	0,000	71	19	26,8	95	0	0,0	0,000	10
**H6548**	chr15	91000530-91000531	C	T	GGT->AGT	**VPS33B**	Gly423Ser; Gly514Ser; Gly487Ser	rs11073964	0,571	86	22	25,6	121	0	0,0	0,000	12
**H6548**	chr3	184936320-184936321	A	C	ATT->CTT	**VPS8**	Ile990Leu; Ile900Leu; Ile419Leu	rs146067504	0,004	27	9	33,3	83	0	0,0	0,000	11
**H6548**	chr14	74356939-74356940	C	T	CTC->TTC	**VRTN**	Leu53Phe	rs2232032	0,002	242	70	28,9	255	0	0,0	0,000	0
**H6548**	chr16	22132996-22132997	C	T	ACA->ATA	**VWA3A**	Thr665Ile; Thr657Ile	rs1105929	0,409	101	38	37,6	196	0	0,0	0,000	0
**H6548**	chr1	20332825-20332826	A	G	ATC->GTC	**VWA5B1**	Ile252Val; Ile545Val; Ile440Val;	rs199681702	0,002	91	25	27,5	235	0	0,0	0,000	0
**H6548**	chr7	49775793-49775794	C	G	GCG->GGG	**VWC2**	Ala120Gly	rs769604	0,523	410	114	27,8	225	0	0,0	0,000	0

**H6548**	chr12	6064266-6064267	C	T	GTC->ATC	**VWF**	Val471Ile	rs1800377	0,119	131	62	47,3	179	0	0,0	0,000	10
**H6548**	chr22	42020051-42020052	A	G	GAT->GGT	**WBP2NL**	Asp121Gly	rs133335	0,502	109	51	46,8	192	0	0,0	0,000	8
**H6548**	chr7	75073649-75073650	C	T	GGG->AGG	**WBSCR16**	Gly30Arg	rs6955671	0,509	163	42	25,8	66	0	0,0	-2,000	0
**H6548**	chr4	84691740-84691741	T	C	ATT->GTT	**WDFY3**	Ile3032Val; Ile643Val	rs17368018	0,007	64	17	26,6	100	0	0,0	0,000	21
**H6548**	chr10	48901849-48901850	A	C	ACC->CCC	**WDFY4**	Thr2525Pro	rs35423873	0,147	80	21	26,3	178	0	0,0	0,000	27
**H6548**	chrX	48600713-48600714	C	G	CCA->GCA	**WDR13**	Pro185Ala	rs57467079	0,394	50	36	72,0	62	0	0,0	0,000	12
**H6548**	chr4	176179492-176179493	G	A	GCT->ACT	**WDR17**	Ala1295Thr; Ala1256Thr; Ala506Thr	rs11736872	0,253	67	18	26,9	123	0	0,0	0,000	0
**H6548**	chr12	121954131-121954132	C	T	CTT->TTT	**WDR66**	Leu445Phe	rs11043265	0,144	72	23	31,9	125	0	0,0	0,000	32
**H6548**	chr15	53615750-53615751	G	A	CTC->TTC	**WDR72**	Leu819Phe	rs17730281	0,255	96	29	30,2	190	0	0,0	0,000	13
**H6548**	chr1	66822361-66822362	C	T	CGG->CAG	**WDR78**	Arg545Gln; Arg832Gln	rs482082	0,501	58	32	55,2	68	0	0,0	0,000	0
**H6548**	chr17	1726913-1726914	C	T	CCG->CTG	**WDR81**	Pro652Leu	rs57207396	0,254	199	53	26,6	246	0	0,0	0,000	7
**H6548**	chr16	666272-666273	T	C	TGC->CGC	**WDR90**	Cys1555Arg; Cys170Arg; Cys154Arg	rs11866949	0,207	280	88	31,4	365	1	0,3	0,000	0
**H6548**	chr2	68134726-68134727	C	T	GCC->ACC	**WDR92**	Ala180Thr; Ala281Thr	rs138784630	0,012	78	24	30,8	105	0	0,0	0,000	0

**H6548**	chr15	89716913-89716914	T	A	TCC->ACC	**WDR93**	Ser254Thr	rs7163367	0,391	37	10	27,0	67	0	0,0	0,002	0
**H6548**	chr20	45555858-45555859	A	G	ATG->ACG	**WFDC8**	Met96Thr	rs2272955	0,075	51	13	25,5	120	0	0,0	0,000	0
**H6548**	chr4	6301469-6301470	G	A	GCC->ACC	**WFS1**	Ala559Thr; Ala377Thr	rs55814513	0,004	300	79	26,3	359	0	0,0	0,000	6
**H6548**	chr12	862124-862125	C	T	ACA->ATA	**WNK1**	Thr665Ile; Thr112Ile	rs2286007	0,064	51	13	25,5	99	0	0,0	0,000	13
**H6548**	chr8	31081187-31081188	G	A	ATG->ATA	**WRN**	Met387Ile	rs1800391	0,074	113	29	25,7	215	0	0,0	0,000	48
**H6548**	chr4	183269079-183269080	G	T	GCC->TCC	**WWC2**	Ala773Ser; Ala568Ser; Ala455Ser;	rs11941467	0,107	68	17	25,0	106	1	0,9	0,000	0
**H6548**	chrX	123900660-123900661	A	C	CAA->CCA	**XIAP**	Gln423Pro; Gln49Pro	rs5956583	0,332	52	28	53,9	82	0	0,0	0,000	69
**H6548**	chr3	39186551-39186552	T	G	CAC->CCC	**XIRP1**	His965Pro	rs11711871	0,167	181	65	35,9	241	0	0,0	0,000	5
**H6548**	chr14	103699415-103699416	G	A	ACG->ATG	**XRCC3**	Thr241Met	rs861539	0,304	118	85	72,0	95	0	0,0	0,000	70
**H6548**	chr17	50354962-50354963	G	C	AGG->ACG	**XYLT2**	Arg305Thr	rs12451299	0,205	170	49	28,8	200	1	0,5	0,000	8
**H6548**	chr12	32755302-32755303	C	A	GGT->GTT	**YARS2**	Gly191Val	rs11539445	0,126	244	72	29,5	232	0	0,0	0,000	16
**H6548**	chr18	751738-751739	T	G	AAG->CAG	**YES1**	Lys113Gln	rs117449313	0,005	23	10	43,5	50	0	0,0	0,000	46
**H6548**	chr5	113553672-113553673	G	C	GAC->CAC	**YTHDC2**	Asp651His; Asp351His	rs75714066	0,064	89	26	29,2	160	0	0,0	0,000	32

**H6548**	chr14	21894903-21894904	C	G	GCG->GGG	**Z77830**	Ala72Gly	rs12890675	0,383	145	38	26,2	316	0	0,0	0,000	0
**H6548**	chr7	100773853-100773854	G	-T^G		**ZAN**	Cys1923		0,000	73	20	27,4	157	0	0,0	0,000	14
**H6548**	chr11	114064107-114064108	G	A	GAC->AAC	**ZBTB16**	Asp270Asn		0,000	194	56	28,9	216	0	0,0	0,000	79
**H6548**	chr3	114350759-114350760	C	T	GAG->AAG	**ZBTB20**	Glu440Lys; Glu367Lys	rs138924453	0,006	270	95	35,2	307	0	0,0	0,000	23
**H6548**	chr21	41993443-41993444	T	G	AAG->CAG	**ZBTB21**	Lys218Gln	rs871546	0,220	115	40	34,8	214	0	0,0	0,000	0
**H6548**	chr6	33315988-33315989	T	C	ACA->GCA	**ZBTB22**	Thr310Ala	rs3130100	0,546	117	32	27,4	198	1	0,5	0,000	0
**H6548**	chr16	11779301-11779302	T	C	CAT->CGT	**ZC3H7A**	His57Arg	rs16958654	0,060	68	18	26,5	162	0	0,0	0,000	0
**H6548**	chr7	139047750-139047751	G	A	ACT->ATT	**ZC3HAV1**	Thr851Ile	rs3735007	0,507	112	55	49,1	192	0	0,0	0,000	31
**H6548**	chr7	139028729-139028730	C	T	ATG->ATA	**ZC3HAV1L**	Met244Ile	rs17856272	0,075	31	11	35,5	100	0	0,0	0,000	0
**H6548**	chr1	52475172-52475173	C	A	GAC->TAC	**ZCCHC11**	Asp796Tyr; Asp555Tyr	rs12127732	0,009	101	33	32,7	194	1	0,5	0,000	52
**H6548**	chrX	112455211-112455212	G	T	GAC->TAC	**ZCCHC16**	Asp162Tyr	rs7474140	0,153	50	25	50,0	115	0	0,0	0,000	0
**H6548**	chrX	78658071-78658072	G	A	CCT->TCT	**ZCCHC5**	Pro117Ser	rs4077512	0,115	59	20	33,9	100	0	0,0	0,000	0
**H6548**	chr9	86322936-86322937	T	-A^TTT		**ZCCHC6**	Asn938; Asn815	rs58050565	0,546	91	48	52,8	133	0	0,0	0,000	35

**H6548**	chr3	28478925-28478926	T	A	CTA->CAA	**ZCWPW2**	Leu202Gln; Leu7Gln	rs1563656	0,436	45	30	66,7	86	0	0,0	0,000	0
**H6548**	chr2	206305006-206305007	G	A	AGA->AAA	**ZDBF2**	Arg160Lys; Arg158Lys	rs10932150	0,413	95	28	29,5	148	0	0,0	0,000	18
**H6548**	chr10	45648509-45648510	T	G	AAG->ACG	**ZFAND4**	Lys44Thr; Lys118Thr	rs17854567	0,132	83	26	31,3	151	0	0,0	0,000	0
**H6548**	chr14	23525307-23525308	A	G	GTT->GCT	**ZFHX2**	Val497Ala; Val1545Ala	rs4982766	0,235	128	38	29,7	250	0	0,0	0,000	0
**H6548**	chr16	72797211-72797212	G	C	CTG->GTG	**ZFHX3**	Leu1824Val; Leu910Val	rs79037957	0,011	105	27	25,7	229	0	0,0	0,000	36
**H6548**	chr19	3831766-3831767	G	A	TCG->TTG	**ZFR2**	Ser164Leu	rs2240235	0,389	194	65	33,5	134	0	0,0	0,000	0
**H6548**	chr20	41203987-41203988	T	C	AAT->AGT	**ZHX3**	Asn310Ser	rs17265513	0,136	120	49	40,8	359	0	0,0	0,000	34
**H6548**	chr3	44570266-44570267	A	G	GAT->GGT	**ZKSCAN7**	Asp170Gly; Asp386Gly; Asp235Gly	rs149725884	0,003	125	48	38,4	173	0	0,0	0,000	0
**H6548**	chr10	79293717-79293718	G	A	CGT->CAT	**ZMIZ1**	Arg314His	rs1250560	0,436	203	54	26,6	194	0	0,0	0,000	0
**H6548**	chr7	64979322-64979323	C	T	TGT->TAT	**ZNF117**	Cys83Tyr	rs3807069	0,374	62	17	27,4	130	0	0,0	0,000	0
**H6548**	chr20	18315431-18315432	G	A	GGG->GAG	**ZNF133**	Gly131Glu; Gly194Glu; Gly99Glu;	rs2228273	0,097	101	30	29,7	210	0	0,0	0,000	34
**H6548**	chr2	218643648-218643649	A	T	CTT->CAT	**ZNF142**	Leu917His; Leu793His; Leu956His	rs3770213	0,315	130	59	45,4	243	0	0,0	0,000	0
**H6548**	chr19	53069982-53069983	C	T	AGC->AAC	**ZNF160**	Ser184Asn	rs74897323	0,008	92	27	29,4	214	0	0,0	0,000	0

**H6548**	chr7	149239786-149239787	G	T	GAG->GAT	**ZNF212**	Glu3Asp	rs11548535	0,196	189	58	30,7	65	0	0,0	-2,000	0
**H6548**	chr11	7001128-7001129	A	C	ATA->AGA	**ZNF214**	Ile185Arg	rs2239734	0,105	71	20	28,2	160	0	0,0	0,000	0
**H6548**	chr11	6932626-6932627	A	G	ATG->GTG	**ZNF215**	Met119Val	rs11041108	0,327	70	24	34,3	142	0	0,0	0,000	19
**H6548**	chr19	44031845-44031846	G	T	GTT->TTT	**ZNF222**	Val4Phe; Val58Phe; Val98Phe	rs7258517	0,577	85	41	48,2	119	0	0,0	0,000	0
**H6548**	chr19	44066239-44066240	C	A	CTA->ATA	**ZNF223**	Leu138Ile	rs6509138	0,541	101	53	52,5	247	0	0,0	0,000	0
**H6548**	chr10	37952138-37952139	A	C	AAT->AAG	**ZNF25**	Asn453Lys; Asn417Lys	rs1208606	0,059	89	24	27,0	113	0	0,0	0,000	0
**H6548**	chr19	24127229-24127230	A	T	GAA->GAT	**ZNF254**	Glu369Asp; Glu325Asp; Glu410Asp;	rs61737180	0,149	105	27	25,7	118	0	0,0	0,000	0
**H6548**	chr12	133203722-133203723	G	T	TTG->TTT	**ZNF268**	Leu518Phe; Leu679Phe; Leu596Phe	rs36127550	0,207	79	20	25,3	116	0	0,0	0,000	42
**H6548**	chr19	44085846-44085847	C	-T^CTC		**ZNF284**	Phe123	rs139900131	0,568	83	34	41,0	166	2	1,2	0,000	0
**H6548**	chr17	18662109-18662110	G	A	CCG->TCG	**ZNF286B**	Pro466Ser	rs9912852	0,337	139	79	56,8	150	0	0,0	0,000	0
**H6548**	chr19	9156615-9156616	G	A	GTC->ATC; ACG->ACA	**ZNF317**	Val52Ile; Thr10Thr	rs35822846	0,073	91	23	25,3	153	0	0,0	0,000	0
**H6548**	chr6	43340122-43340123	G	A	ACC->ATC	**ZNF318**	Thr1292Ile	rs10948072	0,124	79	22	27,9	147	0	0,0	0,000	0
**H6548**	chr10	38056536-38056537	G	C	GAT->CAT	**ZNF33A**	Asp693His; Asp826His; Asp811His;	rs10508862	0,058	89	30	33,7	124	0	0,0	0,000	0

**H6548**	chr10	62655423-62655424	A	G	ACC->GCC	**ZNF365**	Thr62Ala	rs7076156	0,797	140	53	37,9	261	0	0,0	0,000	45
**H6548**	chr18	74631224-74631225	A	G	AAT->AGT	**ZNF407**	Asn68Ser; Asn69Ser	rs3794942	0,063	99	26	26,3	191	0	0,0	0,000	21
**H6548**	chr10	30857685-30857686	T	C	ATG->GTG	**ZNF438**	Met1Val	rs16932010	0,157	37	12	32,4	70	0	0,0	0,000	0
**H6548**	chr19	11868348-11868349	T	C	TTG->TCG	**ZNF439**	Leu291Ser; Leu427Ser	rs10500209	0,201	155	41	26,5	186	1	0,5	0,000	0
**H6548**	chr19	12430399-12430400	G	T	CCA->CAA	**ZNF443**	Pro591Gln	rs7256321	0,324	112	36	32,1	177	0	0,0	0,000	0
**H6548**	chr5	178964858-178964859	G	A	TGT->TAT	**ZNF454**	Cys152Tyr	rs6867221	0,306	85	26	30,6	128	0	0,0	0,000	0
**H6548**	chr16	88435562-88435563	T	A	CTG->CAG	**ZNF469**	Leu2670Gln	rs3812956	0,304	204	66	32,4	188	0	0,0	0,000	2
**H6548**	chr7	57119993-57119994	A	G	TTT->TCT	**ZNF479**	Phe474Ser	rs200382632	0,020	115	37	32,2	177	1	0,6	0,000	0
**H6548**	chr10	47368614-47368615	G	A	GCG->GTG	**ZNF488**	Ala72Val		0,000	350	105	30,0	193	0	0,0	0,000	0
**H6548**	chr19	58357113-58357114	G	T	CAC->CAA	**ZNF497**	His174Gln	rs12609654	0,411	392	133	33,9	241	0	0,0	0,000	0
**H6548**	chr20	63966312-63966313	C	T	GTG->ATG	**ZNF512B**	Val288Met	rs45486695	0,157	251	64	25,5	516	0	0,0	0,000	11
**H6548**	chr8	144807456-144807457	G	A	GTG->ATG	**ZNF517**	Val181Met; Val87Met	rs62531506	0,137	339	118	34,8	213	0	0,0	0,000	0
**H6548**	chr19	2917543-2917544	A	G	TAT->TGT	**ZNF57**	Tyr308Cys; Tyr276Cys	rs138288250	0,002	68	17	25,0	103	1	1,0	0,000	0

**H6548**	chr19	51877313-51877314	G	A	GCA->GTA	**ZNF577**	Ala84Val; Ala77Val	rs17856123	0,025	100	30	30,0	168	0	0,0	0,000	0
**H6548**	chr19	52511498-52511499	G	A	TGT->TAT	**ZNF578**	Cys373Tyr	rs61739625	0,005	149	45	30,2	287	0	0,0	0,000	0
**H6548**	chr3	48241204-48241205	A	G	ACT->GCT	**ZNF589**	Thr12Ala	rs9847953	0,265	246	91	37,0	168	0	0,0	0,000	28
**H6548**	chr19	52767130-52767131	A	G	TGT->CGT	**ZNF600**	Cys209Arg	rs1820128	0,817	124	38	30,7	282	1	0,4	0,000	0
**H6548**	chr19	57980258-57980259	T	C	AGC->GGC	**ZNF606**	Ser141Gly; Ser51Gly	rs11673029	0,137	99	28	28,3	194	1	0,5	0,000	0
**H6548**	chr19	52016354-52016355	C	T	GTT->ATT	**ZNF614**	Val415Ile; Val118Ile	rs8104890	0,338	98	28	28,6	176	0	0,0	0,000	0
**H6548**	chr19	51994491-51994492	T	C	CAG->CGG	**ZNF615**	Gln206Arg; Gln195Arg; Gln202Arg;	rs77230420	0,019	71	27	38,0	150	0	0,0	0,000	0
**H6548**	chrX	48060475-48060476	C	T	AGT->AAT	**ZNF630**	Ser71Asn; Ser57Asn	rs41297342	0,026	89	64	71,9	55	0	0,0	0,000	0
**H6548**	chr2	71427044-71427045	G	A	GTG->ATG	**ZNF638**	Val1726Met; Val823Met; Val95Met	rs1804020	0,285	104	26	25,0	168	1	0,6	0,000	0
**H6548**	chr16	31077303-31077304	A	G	GAG->GGG	**ZNF646**	Glu327Gly	rs749670	0,382	119	41	34,5	294	0	0,0	0,000	0
**H6548**	chr12	124012316-124012317	T	G	GTC->GGC	**ZNF664**	Val58Gly	rs80197353	0,079	83	22	26,5	115	0	0,0	0,000	11
**H6548**	chr1	247103971-247103972	C	A	GAG->GAT	**ZNF669**	Glu76Asp	rs4925692	0,380	93	29	31,2	32	0	0,0	0,000	0
**H6548**	chr19	22181430-22181431	C	T	GAA->AAA	**ZNF676**	Glu96Lys	rs12986319	0,113	77	23	29,9	134	0	0,0	0,000	0

**H6548**	chr19	23744113-23744114	T	C	AAA->AGA	**ZNF681**	Lys479Arg; Lys410Arg	rs1852432	0,094	178	47	26,4	179	0	0,0	0,000	0
**H6548**	chr4	161922-161923	A	G	CAT->CGT; CAT->CGT	**ZNF718**	His381Arg; His413Arg; Ile193Val	rs7440274	0,196	79	24	30,4	105	1	1,0	0,000	0
**H6548**	chr19	22976044-22976045	T	A	AAA->ATA	**ZNF728**	Lys248Ile; Lys431Ile	rs1433111	0,129	197	104	52,8	232	0	0,0	0,000	0
**H6548**	chr7	64219720-64219721	C	A	CAC->AAC	**ZNF735P**	His224Asn	rs4320434	0,289	102	28	27,5	152	1	0,7	0,000	0
**H6548**	chr19	57445459-57445460	T	G	ATA->AGA	**ZNF749**	Ile771Arg	rs7246856	0,671	69	28	40,6	107	0	0,0	0,000	0
**H6548**	chr19	53456313-53456314	G	C	GAG->CAG	**ZNF761**	Glu603Gln; Glu549Gln	rs2617726	0,424	222	110	49,6	317	1	0,3	0,000	0
**H6548**	chr16	89223174-89223175	G	A	GAC->AAC	**ZNF778**	Asp46Asn	rs147281237	0,002	151	43	28,5	213	0	0,0	0,000	0
**H6548**	chr19	12113356-12113357	T	C	TAT->CAT	**ZNF788**	Tyr604His	rs8109273	0,141	76	26	34,2	131	0	0,0	0,000	0
**H6548**	chr19	12391972-12391973	G	A	ACG->ATG	**ZNF799**	Thr142Met	rs12974755	0,127	166	44	26,5	356	1	0,3	0,000	0
**H6548**	chr7	89334881-89334882	T	A	TTT->ATT	**ZNF804B**	Phe634Ile	rs801840	0,350	73	23	31,5	141	0	0,0	0,000	0
**H6548**	chr19	52553699-52553700	G	A	GAT->AAT	**ZNF808**	Asp262Asn	rs329965	0,254	112	30	26,8	176	0	0,0	0,000	0
**H6548**	chr20	59194084-59194085	G	C	TTG->TTC	**ZNF831**	Leu1022Phe	rs55786258	0,205	124	37	29,8	179	0	0,0	0,000	0
**H6548**	chr19	52067460-52067461	C	G	GTT->CTT	**ZNF841**	Val141Leu; Val25Leu	rs16983412	0,212	71	19	26,8	180	0	0,0	0,000	0

**H6548**	chr19	12075795-12075796	A	G	AAA->GAA	**ZNF844**	Lys226Glu; Lys69Glu	rs7259684	0,108	53	23	43,4	134	0	0,0	0,000	0
**H6548**	chr3	31990469-31990470	A	G	CAT->CGT	**ZNF860**	His464Arg	rs1808125	0,098	110	40	36,4	328	1	0,3	0,000	0
**H6548**	chr7	149864233-149864234	G	A	GAG->AAG	**ZNF862**	Glu1154Lys	rs62621237	0,044	23	10	43,5	29	0	0,0	0,001	0
**H6548**	chr19	12043983-12043984	G	C	CAC->GAC	**ZNF878**	His473Asp	rs67102109	0,073	83	21	25,3	153	0	0,0	0,000	0
**H6548**	chr7	65398478-65398479	C	T	GCA->GTA	**ZNF92**	Ala46Val; Ala122Val; Ala90Val;	rs10265083	0,234	86	32	37,2	156	0	0,0	0,000	0
**H6548**	chr19	19934122-19934123	G	A	GTT->ATT	**ZNF93**	Val390Ile	rs62135329	0,031	164	44	26,8	227	2	0,9	0,000	0
**H6548**	chr19	22392015-22392016	T	C	AAA->GAA	**ZNF98**	Lys407Glu	rs201005223	0,515	52	26	50,0	53	0	0,0	0,000	0
**H6548**	chr19	22758388-22758389	T	A	AAA->ATA	**ZNF99**	Lys507Ile	rs11667397	0,172	176	53	30,1	284	0	0,0	0,000	0
**H6548**	chr19	5455966-5455967	T	C	GTG->GCG	**ZNRF4**	Val159Ala	rs8107825	0,531	527	141	26,8	213	0	0,0	0,000	0
**H6548**	chr7	76425054-76425055	G	A	GGA->AGA	**ZP3**	Gly31Arg	rs2286428	0,240	103	37	35,9	248	0	0,0	0,000	4
**H6548**	chr15	43369603-43369604	T	C	AGA->GGA	**ZSCAN29**	Arg104Gly; Arg103Gly	rs3809482	0,389	87	54	62,1	199	1	0,5	0,000	0
**H6548**	chr20	45877333-45877334	T	C	GTC->GCC	**ZSWIM3**	Val253Ala; Val259Ala	rs2903808	0,604	149	40	26,9	276	0	0,0	0,000	0
**H6548**	chr6	116652018-116652019	T	C	AAC->GAC	**ZUFSP**	Asn379Asp; Asn183Asp	rs4946188	0,356	53	15	28,3	71	0	0,0	0,000	0

Supplementary Table S3. Germline DQB1 variants detected in 8 ALK+ ALCL patients treated with crizotinib, and association with response. The three variants (Leu58/Val102/Gly235) are shared by alleles DQB1*06:02 and DQB1*06:03. All three relapsed patients were homozygous for these variants.

Patient ID	AA58	AA102	AA235	response
3953	Leu / Leu	Val / Val	Gly / Gly	Relapsed
10752	Leu / Leu	Val / Val	Gly / Gly	Relapsed
9667	Leu / Leu	Val / Val	Gly / Gly	Relapsed
K18	His / Phe	Val / Val	Gly / Gly	Non-relapsed
H6548	His / Phe	Val / Ile	Gly / Arg	Non-relapsed
E2013	His / Phe	Val / Ile	Gly / Arg	Non-relapsed
687	His / Phe	Val / Val	Gly / Arg	Non-relapsed
I2010	Leu / Leu	Val / Ile	Gly / Gly	Non-relapsed

Supplementary Table S4. Functional effect of somatic alterations in FAT cadherins, detected in ALCL patients, assessed by the indicated tools.

Patient ID		Protein	Variant	PolyPhen-2	Provean
ALK+	3953	**FAT4**	G2959S	Damaging	Deleterious
ALK+	6548	**FAT4**	Q453L	Benign	Neutral
ALK+	B4	**FAT4**	E2165*	Truncating	Truncating
ALK-	Neg2	**FAT4**	G2346R	Damaging	Deleterious
ALK-	Neg2	**FAT4**	N615D	Damaging	Deleterious
ALK-	Neg3	**FAT4**	S77I	Benign	Neutral
ALK+	10752	**FAT1**	N1487I	Damaging	Deleterious
ALK+	H6548	**FAT1**	A131V	Damaging	Neutral
ALK+	3953	**FAT1**	deletion	--	--
ALK+	20871	**FAT1**	N1487I	Damaging	Deleterious
ALK-	Neg1	**FAT3**	L290I	Benign	nd
ALK-	Neg1	**FAT3**	E323*	Truncating	Truncating
ALK+	H6548	**FAT3**	S3812G	Benign	nd
ALK+	H6548	**DCHS1**	T1949M	Benign	nd
ALK+	GPS 56-57	**DCHS1**	H2819Y	Damaging	nd
